# Discovery, Structure–Activity Relationship, and Functional Characterization of a Chromenopyrrole Series as Orthosteric Antagonists of GPR84

**DOI:** 10.1021/acs.jmedchem.5c03367

**Published:** 2026-03-23

**Authors:** Michael A. Malone, Ruijing Yin, Yueming Li, Laura Jenkins, Abdul-Akim Guseinov, Sara Marsango, Mark Huggett, Margaret Huggett, Anna Boyle, Angus Morrison, Irina G. Tikhonova, Graeme Milligan, Andrew G. Jamieson

**Affiliations:** a School of Chemistry, The Advanced Research Centre, 3526 University of Glasgow, 11 Chapel Lane, Glasgow G11 6EW, U.K.; b Centre for Translational Pharmacology, The Advanced Research Centre, 3526 University of Glasgow, 11 Chapel Lane, Glasgow G11 6EW, U.K.; c School of Pharmacy, Queen’s University Belfast, Belfast BT9 7BL, U.K.; d 523043 BioAscent Discovery Ltd, Bo’Ness Road, Newhouse, Lanarkshire ML1 5UH, U.K.

## Abstract

GPR84 is a proinflammatory G-protein-coupled receptor implicated in autoimmune and fibrotic disorders. Although orthosteric antagonists have been reported, their physicochemical limitations have hindered development. Here, we describe the discovery and optimization of a chromenopyrrole scaffold as a new class of orthosteric GPR84 antagonists. Guided by molecular modeling and iterative SAR, we identified ligands that competitively inhibit agonist binding, confirmed by Schild analysis and radioligand displacement. Structural refinement defined key steric and hydrophobic features required for high-affinity binding, culminating in the isolation of a single active enantiomer, **42E2** (p*A*_2_ = 8.41, p*K*_i_ = 8.16). This chemotype displays improved drug-like properties relative to earlier series and strong selectivity over related free fatty acid receptors. Preliminary pharmacokinetic studies indicate favorable solubility and plasma protein binding, although metabolic stability remains to be optimized. These results expand the chemical space for GPR84 modulation and provide a foundation for therapeutic development and mechanistic investigation.

## Graphical abstract

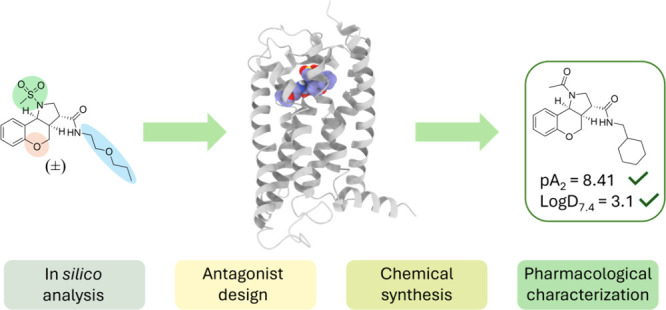

## 1 Introduction

G-protein-coupled receptors (GPCRs) represent the largest and most versatile family of transmembrane signaling proteins, orchestrating a vast array of physiological processes and serving as the targets for more than one-third of approved therapeutics. Free fatty acid receptors (FFARs), a subclass of GPCRs, have emerged as critical mediators of metabolic and immunological homeostasis, linking nutrient sensing to hormonal and inflammatory responses. Selective modulation of FFARs therefore offers a powerful strategy to reduce hyperglycemia, improve insulin sensitivity, and combat metabolic syndrome.^1^

GPR84 is a putative FFAR, acting on the G_i_ pathway, with medium-chain fatty acids (MCFAs) displaying agonism with potency in the micromolar range.^2,3^ However, due to the modest potency of these ligands, it remains uncertain whether MCFAs are the true endogenous regulators.^4^ Upregulation of GPR84 is observed in a range of immune cells in response to proinflammatory stimuli.^5^ In addition to increasing cytokine production from neutrophils and macrophages, activation of the receptor enhances chemotaxis in macrophages, monocytes, and neutrophils.^3,6–9^ Pharmacological evaluation of targeting GPR84 in selected tissue types has also been widely reported, and multiple synthetic agonists of GPR84 have been developed.^10–13^ Understanding the mode of binding of orthosteric agonists has been provided by a series of cryo-EM structures of GPR84–G_i_ complexes, including with 6-OAU, LY237, and the MCFA 3-OH lauric acid.^14,15^

Antagonism of GPR84, on the other hand, has been shown to reduce interstitial fibrosis following abdominal aorta trauma in rats and is also documented to suppress renal disease progression in mouse models.^16,17^ Such characteristics of GPR84 more generally suggest that antagonism may have therapeutic potential in the treatment of autoimmune diseases. Despite this, only three antagonist series have been reported. Galapagos developed the antagonist GLPG1205 (Figure 1), which reached phase 2 clinical trials as a potential treatment for ulcerative colitis.^18^ Mutagenesis studies showed that GLPG1205-mediated inhibition of GPR84 was not prevented by alteration of Arg^172^, a key residue for agonist function in the orthosteric binding pocket. In concert with detailed pharmacological studies, GLPG1205 was shown to act at a distinct allosteric binding site, functioning as a negative allosteric modulator.^18^ In addition, Kalita et al. have reported PET imaging agents based on the allosteric Galapagos series.^19^ The lead compound, ^11^C-MGX-11S (Figure 1), showed promising selectivity and effective blood–brain barrier permeability in mouse models.^19^ Due to the upregulation of GPR84 in proinflammatory settings, ^11^C-MGX-11S showcases the potential of GPR84 ligands as novel neuroinflammation imaging tools.^19^

**Figure 1:**
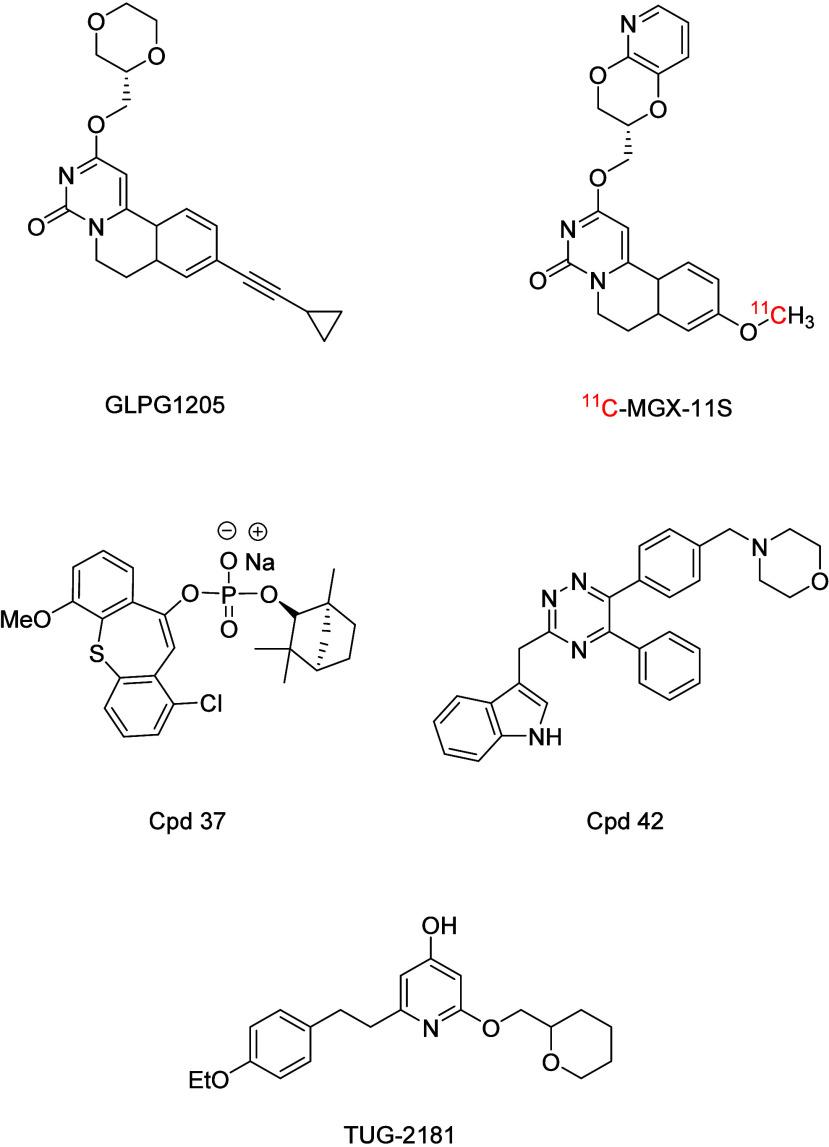
Allosteric antagonist GLPG1205. Radio-labeled allosteric binder ^11^C-MGX-11S. Allosteric phosphodiester antagonist **cpd 37**. Orthosteric 1,2,4-triazine antagonist **cpd 42**. Orthosteric inverse agonist TUG-2181.

Chen et al. reported the identification of phosphodiester-based GPR84 antagonists.^20^ These showed inhibition of 6-OAU-stimulated GPR84 activity in an apparently noncompetitive manner, strongly suggesting allosteric binding of these phosphodiesters.^20^ Desymmetrizing the phosphodiester scaffold led to improved polarity and the overall pharmacokinetic profile of the series lead.^21^ The most encouraging candidate was a (−)-fenchol analog (**Cpd 37**) (Figure 1) with a logD of 2.8 and a pIC_50_ = 8.28 in an acute lung injury mouse model.^21^ Incorporating the phosphodiester scaffold, Nan and co-workers also developed a fluorescent probe capable of GPR84 bioimaging.^22^

Our group previously reported the first GPR84 orthosteric antagonist series, based on a 1,2,4-triazine scaffold.^23^ The most potent derivative of the series was a 6-substituted morpholino derivative with a pIC_50_ of 8.28 (**Cpd 42**) (Figure 1).^24^ A radio-labeled analog from this series, [^3^H]­140, has provided a means to characterize the binding affinity of orthosteric ligands of GPR84.^23^ However, the polyaromatic and lipophilic nature of these compounds results in poor pharmacokinetic profiles, leaving a requirement for further development of alternative GPR84 orthosteric antagonists.^23,24^

Recently, Ulven and co-workers synthetically altered the agonist LY237 to develop inverse agonists (e.g., TUG-2181 with an EC_50_ = 8.0 nM). The inclusion of the cyclic ether at the 2-position of the pyridine ring was proposed to facilitate the observed functional switch within this series.^25^

Herein, we report the discovery and validation of a novel series of orthosteric antagonists targeting GPR84. Through an integrated structure-based drug design approach guided by molecular modeling, we identified and pharmacologically characterized a ligand series that defines a new chemical space for selective GPR84 modulation.

## 2 Results

### 2.1 Initial Discovery of a Chromenopyrrole-Based GPR84 Antagonist

A high-throughput screen to detect compounds able to prevent GPR84-mediated inhibition of cAMP production was conducted in collaboration with the European Lead Factory.^23^ Two clusters were identified, including the previously described 1,2,4-triazine series and a racemic chromenopyrrole GPR84 antagonist.^23,24^ Preliminary investigations surrounding the chromenopyrrole scaffold led to compound **1** (Figure 2a), the starting point for exploring and optimizing this chemotype’s activity. Subsequent orthogonal assays designed to measure inhibition of GPR84 agonist-promoted binding of [^35^S]­GTPγS confirmed the GPR84 antagonist behavior of this compound with a measured IC_50_ of 4.4 μM against an EC_80_ concentration of a GPR84 orthosteric agonist. Furthermore, Schild analysis demonstrated that increasing concentrations of chromenopyrrole **1** caused a surmountable rightward shift in the concentration–response curve for the orthosteric agonist OX04539.^13,26^ This is consistent with chromenopyrrole **1** acting as an orthosteric antagonist of GPR84, with a p*A*_2_ estimated at 6.2 (Figure 2b). The high degree of sp^3^ character, combined with the intrinsic chirality of the tricyclic core, imparts desirable drug-like properties to these compounds.^27^ Moreover, chromenopyrrole scaffolds are widely represented in nature and drug discovery, featuring prominently in bioactive molecules with reported anti-HIV-1 and antibacterial activities.^28^

**Figure 2:**
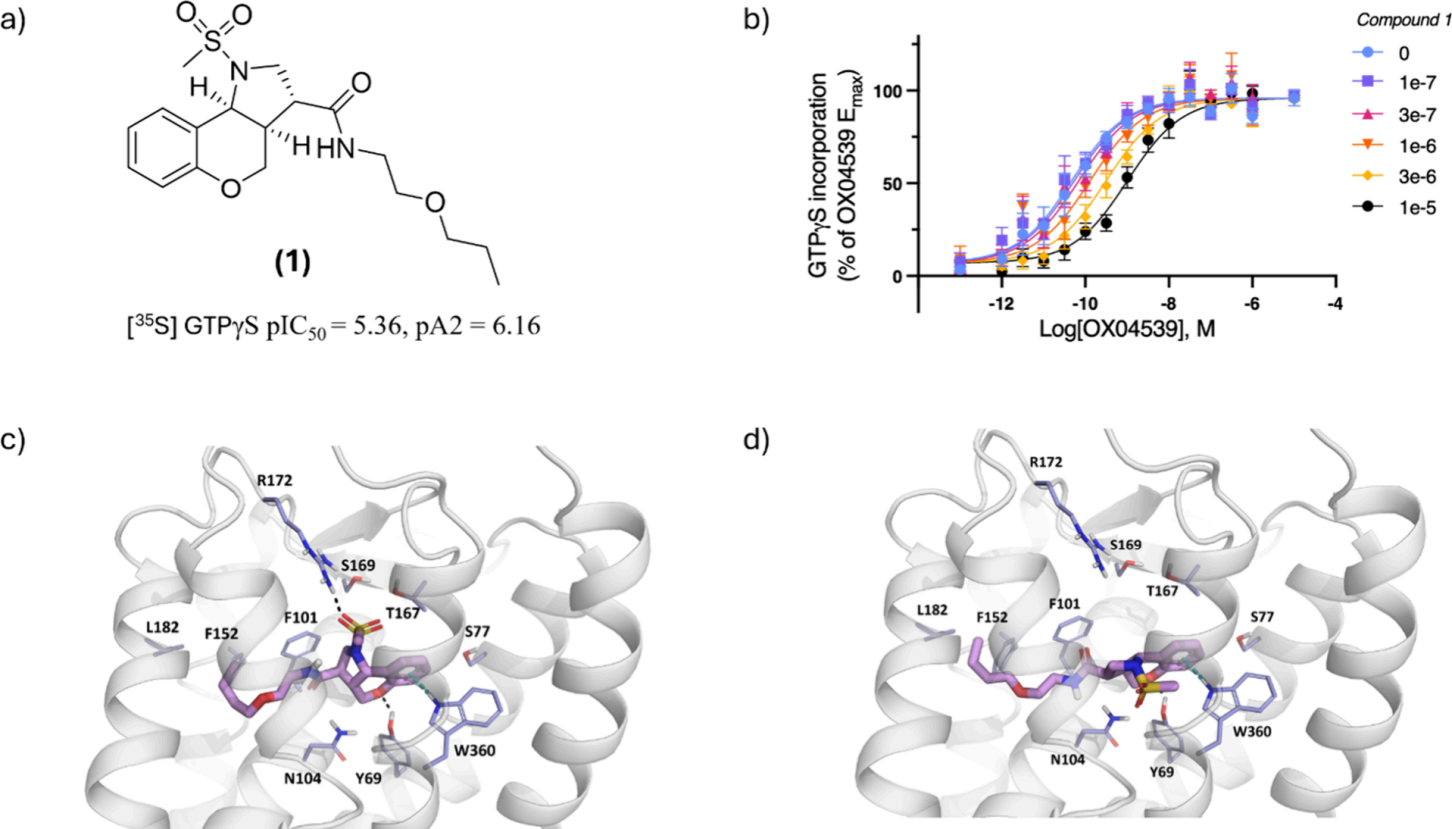
(a) Structure of the chromenopyrrole **1** scaffold, which was pharmacologically characterized in these studies. (b) Increasing concentrations of compound **1** promote surmountable increases in the concentration of the orthosteric agonist OX04539 required to promote activation of the G-protein G_i_ measured by binding of [^35^S]­GTPγS. Schild analysis indicated compound **1** to be a competitive antagonist with estimated p*A*_2_ = 6.2. (c) Docking-predicted interaction of **1** (enantiomer *S*,*S*,*R*) with an antagonist-optimized 3D model derived from the 6-OAU-bound cryo-EM structure of human GPR84. (d) Predicted binding pose of **1** (enantiomer *R*,*R*,*S*) with the homology model of human GPR84.

### 2.2 *In Silico* Docking

No inactive-state or antagonist-bound structures of GPR84 are currently available. To facilitate structure-based design of the chromenopyrrole antagonist series, we developed a model based on the 6-OAU-bound cryo-EM structure of a human GPR84–G_i_ complex (PDB: 8G05).^15^

In this agonist-bound structure, 6-OAU forms five key hydrogen bonds within the binding pocket, resulting in a contracted orthosteric site that limits accommodation of bulkier antagonist scaffolds. To address this constraint, the structure was relaxed in the absence of agonist using short implicit solvent molecular dynamics simulations followed by energy minimization. In parallel, the AlphaFold-predicted model of the inactive GPR84 state was also employed for docking studies.

As compound **1** was identified as a racemic mixture, we investigated the binding modes of both enantiomers in each model. Docking results indicated a substantially more favorable fit for the (*S*,*S*,*R*) configuration within the orthosteric site (Figure 2c). *In silico* analysis revealed that the aliphatic tail of the chromenopyrrole was oriented toward the hydrophobic residues Leu182^5.42^, Phe152^4.57^, and Phe101^3.53^, positioning the lipophilic ether moiety deep within the hydrophobic pocket. Additionally, potential N*H*–π interactions were observed between the tricyclic core scaffold and Trp360^7.43^, with hydrogen bonding between Tyr69^2.53^ and the chromenopyrrole cyclic ether. The mesylate moiety of compound **1** was positioned to form a hydrogen bond with Arg172^ECL2^, located near the upper region of the orthosteric binding site.

In contrast, the (*R*,*R*,*S*) enantiomer (Figure 2d) failed to establish key interactions with these polar residues and exhibited a steric clash between its aliphatic tail and the hydrophobic wall of the binding pocket.

### 2.3 Structure–Activity Relationship

Retaining the sulfonamide headgroup of the tricyclic core, initial structure–activity relationship (SAR) studies focused on modifications to the lipophilic tail of chromenopyrrole **1** to define the optimal acyclic chain length and substitution pattern at R_1_ (Table 1). A corresponding series of analogs were synthesized and evaluated. Exchange of the ether moiety to give the *n*-hexane analog (**2**) resulted in a loss of ability to inhibit GPR84 in the [^35^S]­GTPγS binding assay, indicating a potential hydrogen bond interaction between the ether and Leu182^5.42^. Terminal fluorination (**3**) yielded no antagonist activity. The isopropylethyl, isobutyl, 2,2-dimethylpropyl, and 2-ethylbutyl analogs (**4**–**7**) were all inactive. Cyclopropylmethyl and cyclobutylmethyl derivatives (**8**–**12**) also showed no antagonist activity, suggesting that small cycloalkyl substituents are not well tolerated. In contrast, cyclopentylmethyl chromenopyrrole **13** (IC_50_ = 3.2 μM) showed comparable activity to **1** (IC_50_ = 4.4 μM). Increasing the ring size to cyclohexylmethyl (**14**) or cycloheptanemethyl (**15**) resulted in some 10-fold improvement in potency to 0.6 μM for the racemic mixtures. The cyclooctanemethyl analog (**16**) also showed improved activity relative to the initial compound (IC_50_ = 1.0 μM), with the adamantyl compound (**17**) displaying comparable potency to **1**. The relative activity of analogs **13**–**16** compared to **2**–**12** suggests favorable van der Waals interactions with the hydrophobic wall of the orthosteric binding pocket, establishing an optimal size constraint for this region. The cyclohexyl analog **14** was investigated further due to the promising IC_50_ and favorable cLogP value.

**Table 1:**
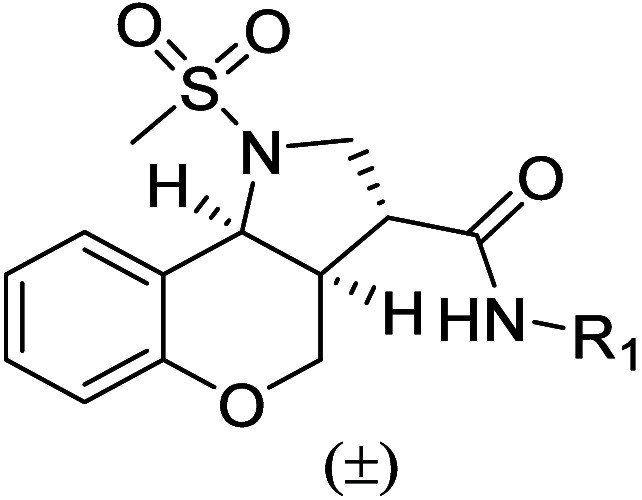

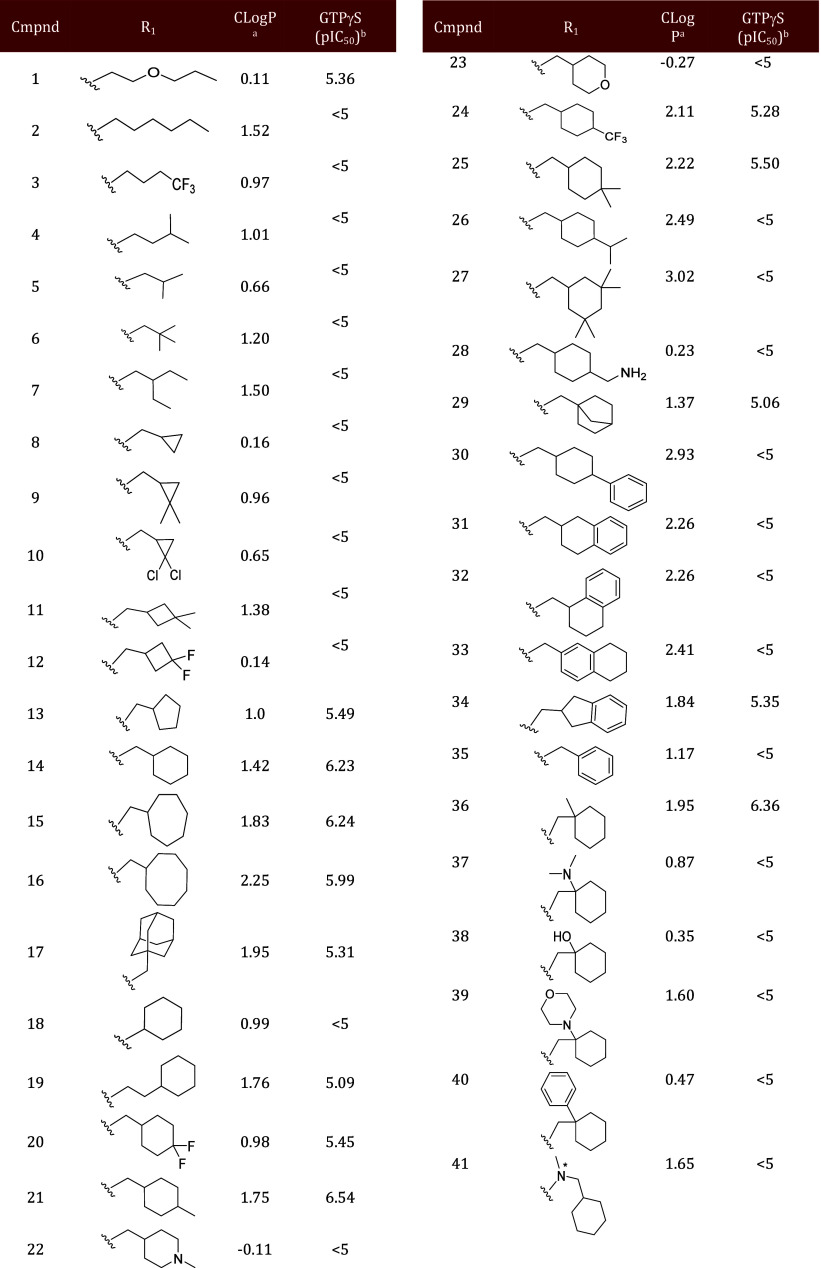
ClogP and GTPγS pIC_50_ of Compounds **1**–**41**

a Logarithm of the partition coefficient for *n*-octanol/water (CLogP) was calculated using ChemDraw2025.

b Negative log of the IC_50_ in molar (pIC_50_); a guanosine 5′-*O*-[gammathio]­triphosphate ([^35^S]­GTPγS) assay following 2-HTP promoted G-protein recruitment of human GPR84 (*n* = 3). *denotes nitrogen of NHR_1_ in the general structure of Table 1.

Initially, the methylene linker was removed, resulting in loss of activity (**18**). Furthermore, the inclusion of an ethyl spacer **19** reduced activity (8.7 μM) relative to **14** suggesting a requirement of a methylene spacer to ensure optimal orientation of the cyclic aliphatic tail within the orthosteric binding pocket. Therefore, the inclusion of a methyl group was deemed essential for investigations of the cyclohexyl group.

Geminal difluorination (**20**) of the cyclohexyl ring resulted in a decrease of cLogP and comparable activity (3.5 μM) to **1**. Methylation (**21**) of the cyclohexyl ring resulted in a slight increase in cLogP, and improved IC_50_ (0.3 μM) was observed, suggesting beneficial hydrophobic interactions within the lipophilic pocket. The importance of maintaining a hydrophobic environment was further demonstrated by the loss of activity observed for the 4-amino and 4-oxa cyclohexyl analogs **22** and **23**. Additionally, substitution of the methyl group to trifluoromethyl derivative **24** resulted in a reduction in antagonistic properties (IC_50_ = 5.2 μM). Dimethylation of the cyclohexyl’s 4-position **25** resulted in a 12-fold drop in potency, now comparable to **1**, indicating steric clash. This constraint was further confirmed by the loss of activity for the more highly substituted analogs **26**–**29**.

Aromatic substitution of the cyclohexyl group was not well tolerated in the GPR84 antagonist [^35^S]­GTPγS assay, with loss of activity observed (**30**–**33**). However, the indanylmethyl analog **34**, featuring a cyclopentyl ring fused to benzene, displayed modest activity (IC_50_ = 4.5 μM) comparable to the initial hit **1**. Replacing the cyclohexyl group with benzene (**35**) resulted in no activity. Methylation of the cyclohexyl position (**36**) displayed comparable potency to **14**. More sterically demanding modifications of the cyclohexyl position (**37**–**40**) led to complete loss of activity, suggesting steric clash with the hydrophobic wall of the orthosteric binding pocket. Notably, the tertiary amide analog **41** was inactive, suggesting that the secondary amide NH may participate in a hydrogen-bonding interaction, potentially with Asn104^3.36^.

The SAR exploration of the lipophilic tail revealed that steric balance is critical for this orthosteric antagonist series. Pharmacological evaluation of analogs **1**–**41** demonstrated that cyclic substituents generally provide superior antagonist affinity compared to their acyclic counterparts, with the cyclohexylmethyl group emerging as the optimal substituent. Subsequent investigations focused on altering both the secondary amine of the tricyclic core, as well as functionalization of cyclic R_1_ groups. Thus, a series of analogs bearing either acetyl or sulfonamide substitutions at R_2_ was synthesized (Table 2). Initially, due to the potency of cyclohexyl-containing compound **14**, an acetylated analog was generated. Pleasingly, the racemic mixture **42** resulted in a pIC_50_ = 6.63 in the GPR84 [^35^S]­GTPγS assay. Sulfonamide **43** and acetylated **44** tetrahydrofuran analogs recorded moderate activity (pIC_50_ = 4.99 and 5.60, respectively). Similarly, mesylated R_2_ showed greater antagonism than the relevant acetyl tetrahydropyran (**44** and **45**) and 1,4-dioxane (**46**–**47**) analogs. However, all oxygen-containing heterocycles exhibited reduced activity compared to the parent cyclohexyl analogs, further suggesting that the hydrophobic nature of the cyclohexyl ring is important for optimal binding. Conversely to antagonist **31**, fusion of the saturated heterocyclic ring with an aromatic ring (**49** and **50**) produced moderate potencies (IC_50_ = 2.1 and 0.4 μM, respectively), suggesting that electron donation from the dioxane ring enhances the aromatic interactions of the R_2_ group with Phe101^3.53^ and Phe152^4.57^. To further investigate the electronic effects of aromatic lipophilic tails, a series of halogenated analogs (**51**–**58**) were synthesized. Fluorinated compounds **51**–**54** showed no activity in the GPR84 [^35^S]­GTPγS assay. Interestingly, *m*-Cl benzyl chromenopyrroles (**55** and **56**) showed no GPR84 antagonistic properties, while *p*-Cl antagonists **57** and **58** displayed IC_50_s of 1.8 μM and 234 nM respectively.

**Table 2:**
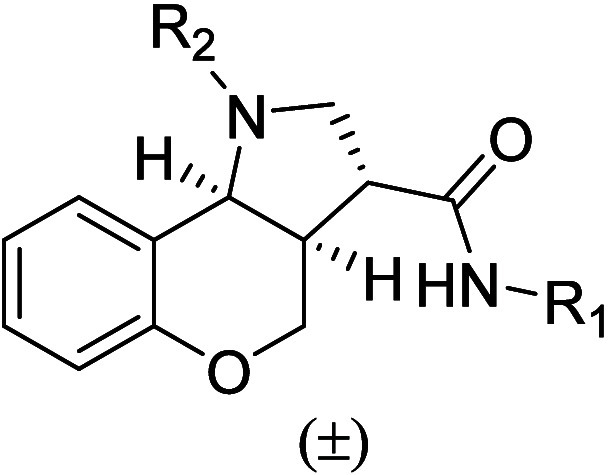

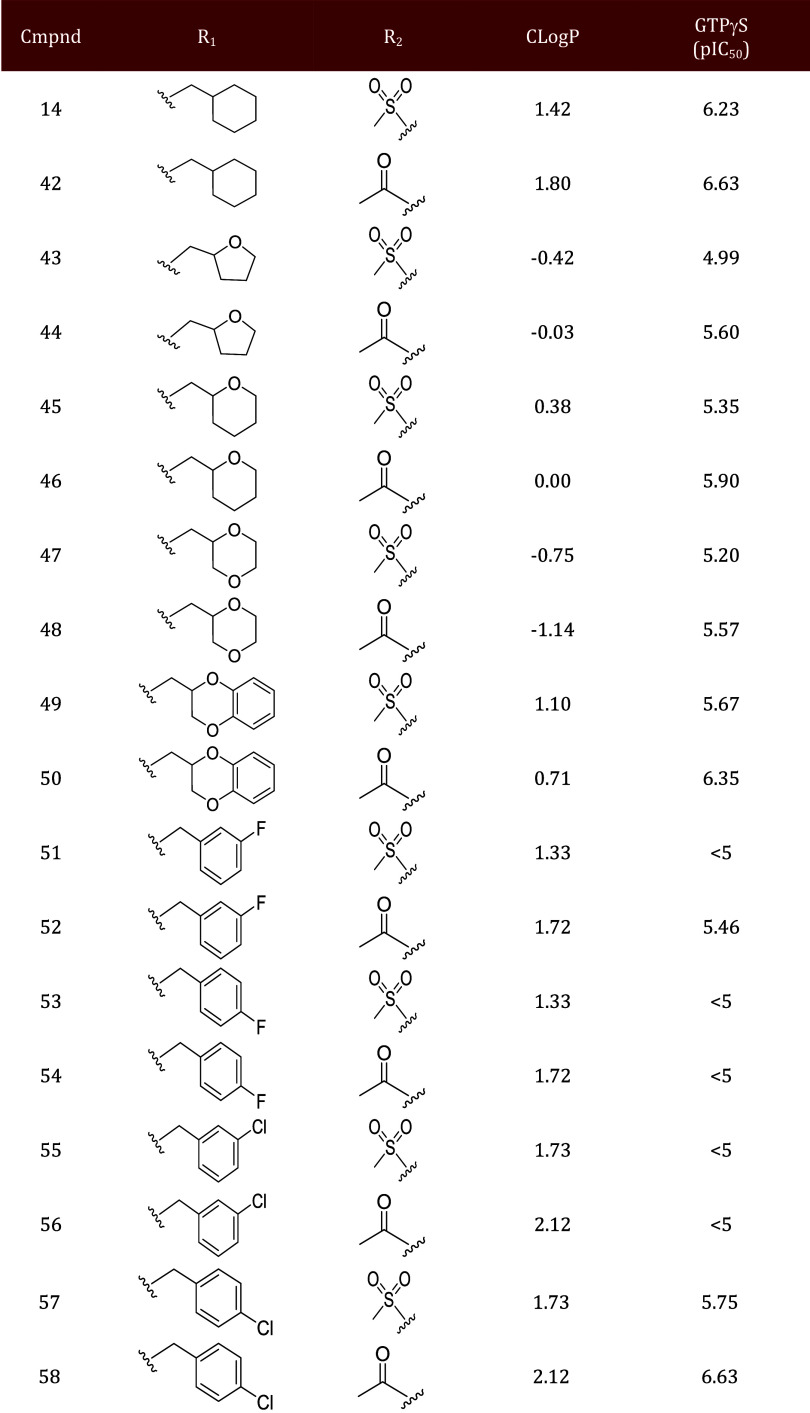
ClogP and GTPγS pIC_50_ of Compounds **42**–**58**

a Logarithm of the partition coefficient for *n*-octanol/water (CLogP) was calculated using ChemDraw2025.

b Negative log of the IC_50_ in molar (pIC_50_); a guanosine 5′-*O*-[gammathio]­triphosphate ([^35^S]­GTPγS) assay following agonist 2-HTP promoted G-protein recruitment of human GPR84 (*n* = 3).

Explorations surrounding the lipophilic tail of the tricyclic core demonstrated the cyclohexyl group as the most favored substituent. Thus, maintaining the cyclohexyl group, investigations surrounding functionalization of the amino portion of the chromenopyrrole were performed (Table 3). Initially, the free secondary amine **59** was assessed, showing reduced activity (IC_50_ = 2.5 μM) compared to the acetylated analog **42**. Methylation (**60**) resulted in no activity, and *p*-methoxy benzyl (**61**) functionalization gave comparable activity to hit compound **1**. Both the propionamide **62** and cyclopropanecarboxamide **63** analogs displayed promising antagonism of GPR84 (IC_50_ = 150 and 250 nM, respectively). The reduced potency of phenyl **64** relative to acetyl **42** is most likely attributable to steric hindrance. However, sulfonamide cyclopropane **65** displayed a 10-fold drop in potency compared to cyclopropane amide **63**, suggesting that amide functionalization of the chromenopyrrole interacts favorably at the top of the pocket. This is further evidenced by acidic sulfonamide isosteres **66** and **67** (IC_50_ = 8.3 and 3.4 μM, respectively).

**Table 3:**
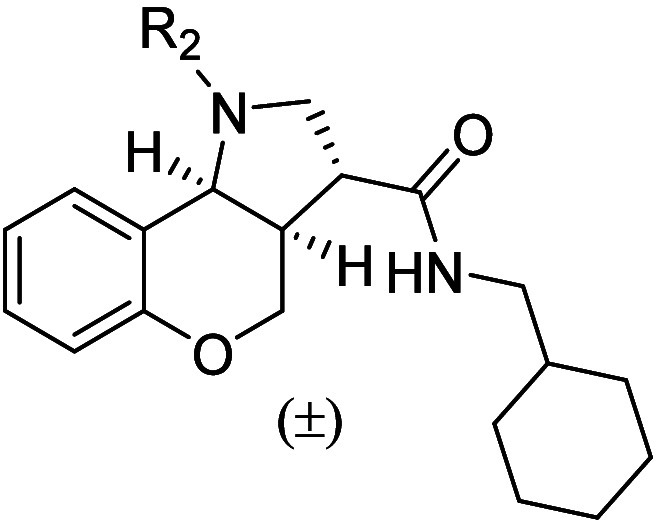

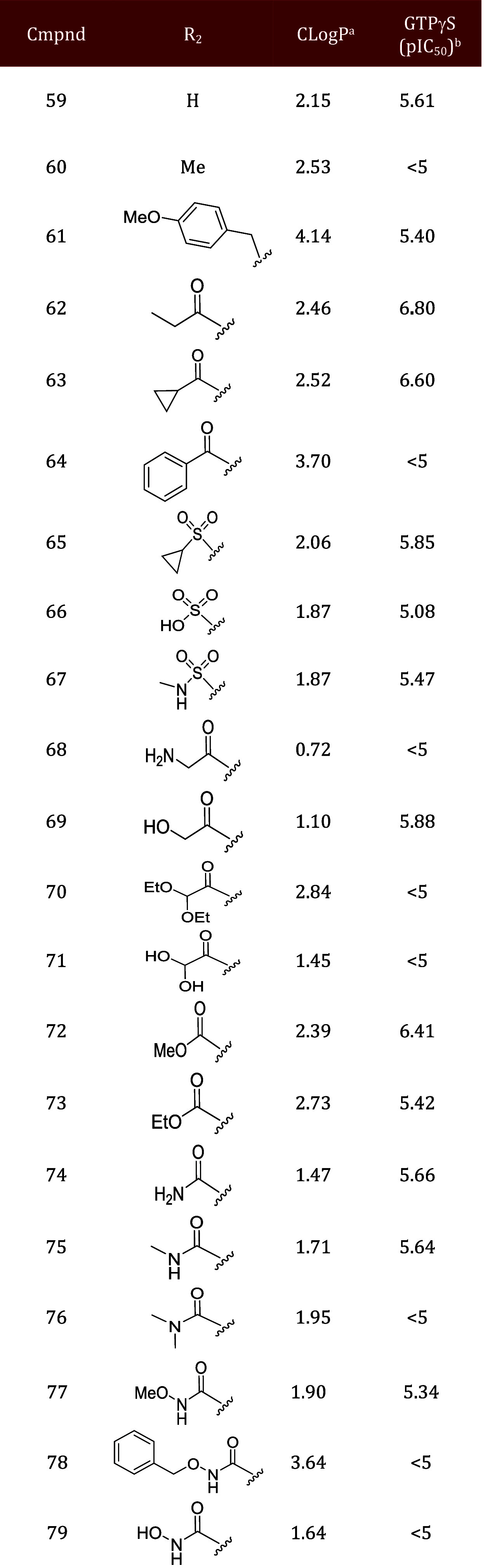
ClogP and GTPγS pIC_50_ of Compounds **59**–**79**

a Logarithm of the partition coefficient for *n*-octanol/water (CLogP) was calculated using ChemDraw2025.

b Negative log of the IC_50_ in molar (pIC_50_); a guanosine 5′-*O*-[gammathio]­triphosphate ([^35^S]­GTPγS) assay following 2-HTP promoted G-protein recruitment of human GPR84 (*n* = 3).

Glycine analog **68** showed no activity, hypothesized to be a consequence of unfavorable electrostatic interactions with Arg172^ECL2^. Molecular modeling of alcohol **69** indicated strong interactions between the hydroxy and Arg172^ECL2^. However, this was not supported experimentally (IC_50_ = 1.3 μM), indicating that the specific orientation of the polar moiety is critical for antagonistic activity. Additionally, **70** and **71** displayed no activity in the GPR84 [^35^S]­GTPγS assay. The introduction of a carbamate moiety was then explored. The racemic methoxycarbamate **72** displayed a promising IC_50_ of 390 nM, proving to be more active than the ethoxy counterpart **73** (IC_50_ = 3.8 μM). Urea and oxy-urea moieties were investigated in attempts to promote hydrogen bonding with Thr167^ECL2^, Ser169^ECL2^, and Arg172^ECL2^. Comparable activity was observed for primary **74** and secondary **75** urea analogs (IC_50_ = 2.2 and 2.3 μM, respectively). Fully substituted tertiary urea **76** demonstrated no antagonism of GPR84. Additionally, *N*-methoxyurea **77** resulted in potency comparable to initial compound **1**. *N*-Benzoyloxyurea and hydroxycarbamide **78** and **79** were inactive in the [^35^S]­GTPγS assay. The importance of the cyclic ether was investigated by exchanging the oxygen with an amino group (Table 4). Secondary amine **80** displayed a 45-fold drop in potency compared to **14**, suggesting that hydrogen bonding acceptors are favored at this position. Additionally, functionalization of the internal amine (**81** and **82**) failed to improve the potency of the hit compound. Activation of GPR84 can also result in recruitment of arrestin adaptor proteins.^29,30^ Comparisons of the antagonistic activity of a subset of ligands in an arrestin-3 recruitment assay and the GTPγS binding assay showed a strong positive correlation (Figure 3), indicating a lack of bias in their activity.

**Table 4:**
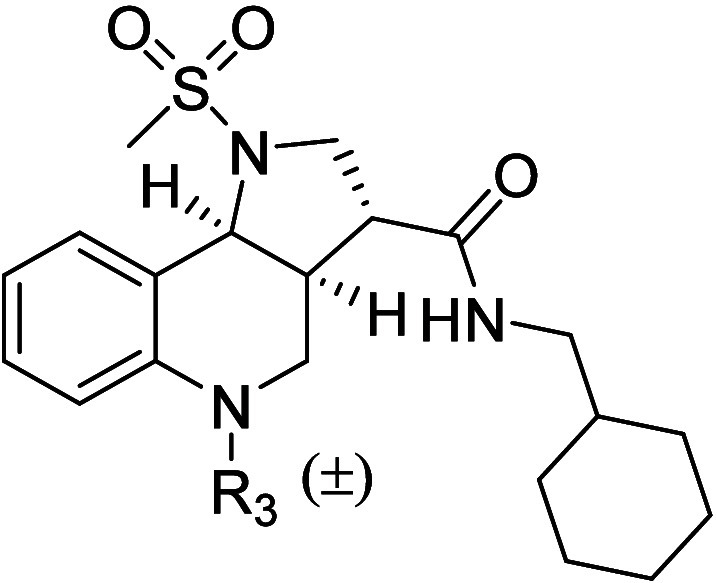
ClogP and GTPγS pIC_50_ of Compounds **80**–**82**

cmpnd	R_3_	CLogP^a^	GTPγS (pIC_50_)^b^
80	H	1.04	5.50
81	Me	1.83	5.76
82	Ac	0.69	<5

a Logarithm of the partition coefficient for *n*-octanol/water (CLogP) was calculated using ChemDraw2025.

b Negative log of the IC_50_ in molar (pIC_50_); a guanosine 5′-*O*-[gammathio]­triphosphate ([^35^S]­GTPγS) assay following 2-HTP promoted G-protein recruitment of human GPR84 (*n* = 3).

**Figure 3:**
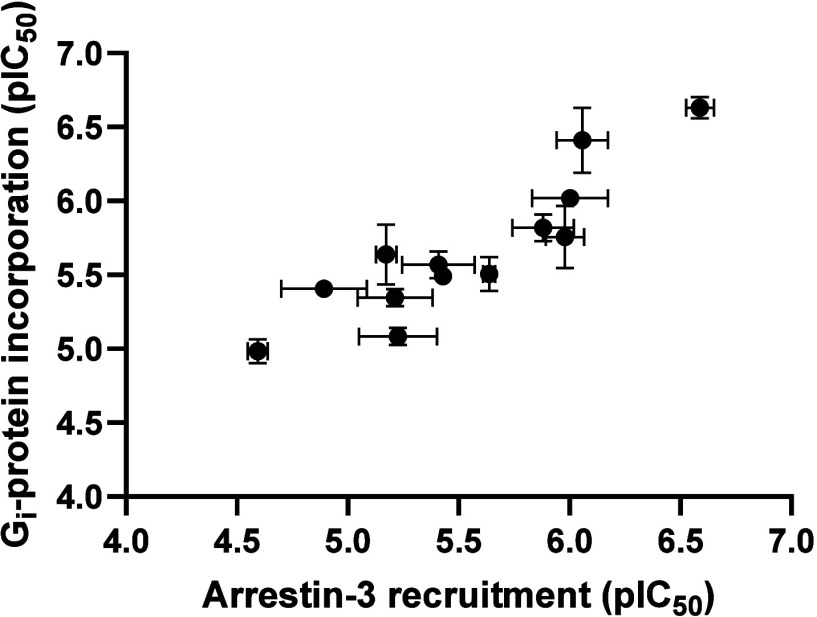
Correlation plot of calculated G-protein inhibition vs arrestin-3 recruitment for a subset of ligands, measured following the 2-HTP activation of GPR84.

### 2.4 GPR84 Antagonist Activity Resides in a Single Enantiomer

The five most potent racemic mixtures (**42**, **58**, **62**, **63**, and **72**) were advanced for more in-depth pharmacological evaluation (Table 5). The racemic mixtures were separated using chiral supercritical fluid chromatography (SFC) to yield two distinct enantiomers. Notably, the activity of these ligands to antagonize agonist-promoted binding of [^35^S]­GTPγS resided entirely in one enantiomer, **E2**, with the other displaying a complete lack of activity (Figure 4a), consistent with the molecular modeling of the two enantiomers into the orthosteric pocket (Figure 2c,d). The affinity for antagonism of GPR84 by **42E2**, **58E2**, **62E2**, **63E2**, and **72E2** was evaluated via Schild analysis of agonist-promoted binding of [^35^S]­GTPγS (Figure 4b,c). As for initial hit (**1**), such characterizations demonstrated their competitive binding nature with agonist OX04539. **42E2** was identified as the ligand with the highest affinity (p*A*_2_ = 8.41). Competition binding studies against [^3^H]­140 confirmed the high affinity of **42E2** (p*K*_i_ = 8.16) with **58E2**, **62E2**, **63E2**, and **72E2** ranging from 7.27 to 8.15 (Figure 4c,d). Subsequently, in combination with docking investigations, the single active enantiomer E2 is suggested to be the *S*,*S*,*R* enantiomer.

**Table 5:**
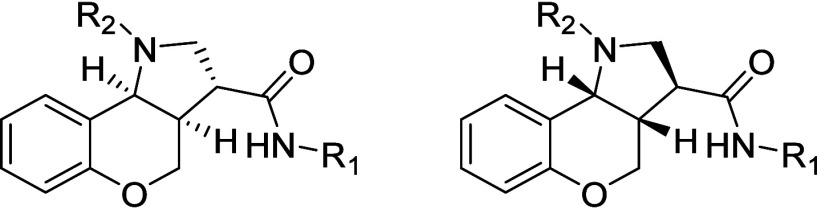

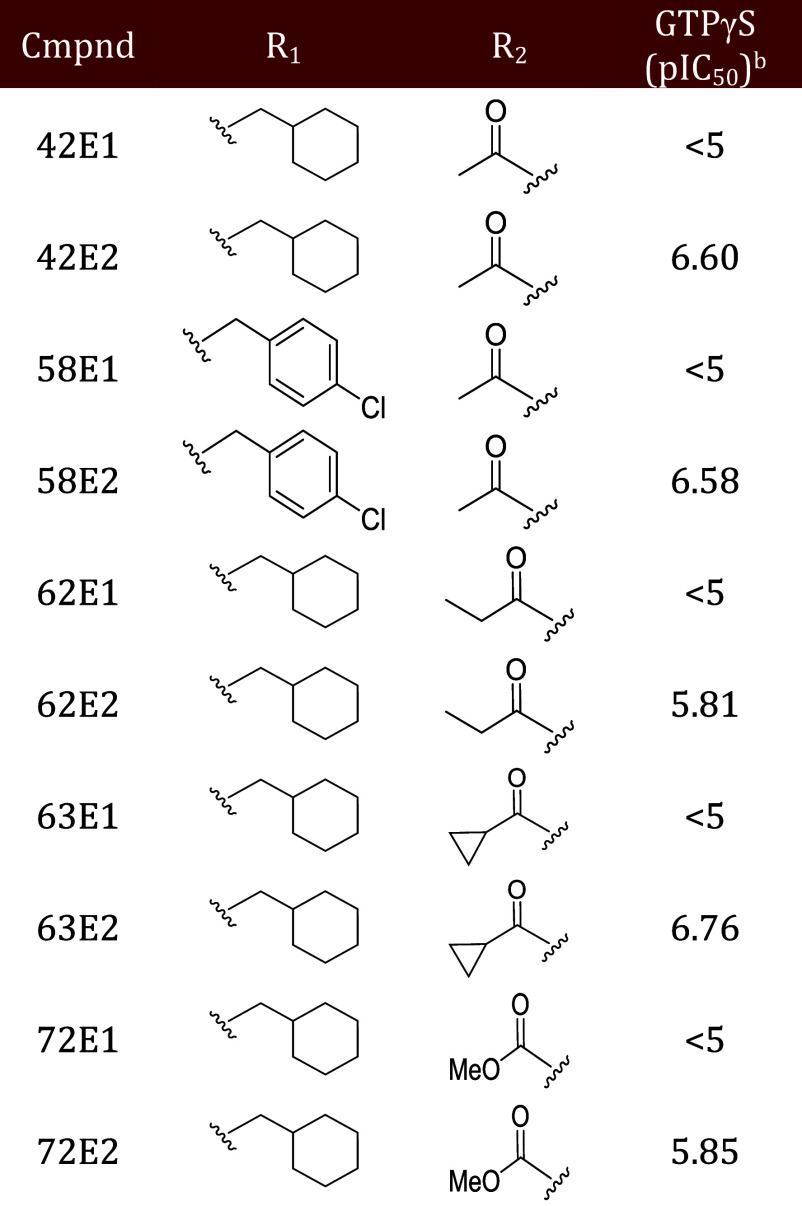
ClogP and GTPγS pIC_50_ of Separated Enantiomers, Compounds **42**, **58**, **62**, **63**, and **72**

a Logarithm of the partition coefficient for *n*-octanol/water (CLogP) was calculated using ChemDraw2025.

b Negative log of the IC_50_ in molar (pIC_50_); a guanosine 5′-*O*-[gammathio]­triphosphate ([^35^S]­GTPγS) assay following 2-HTP promoted G-protein recruitment of human GPR84 (*n* = 3).

**Figure 4:**
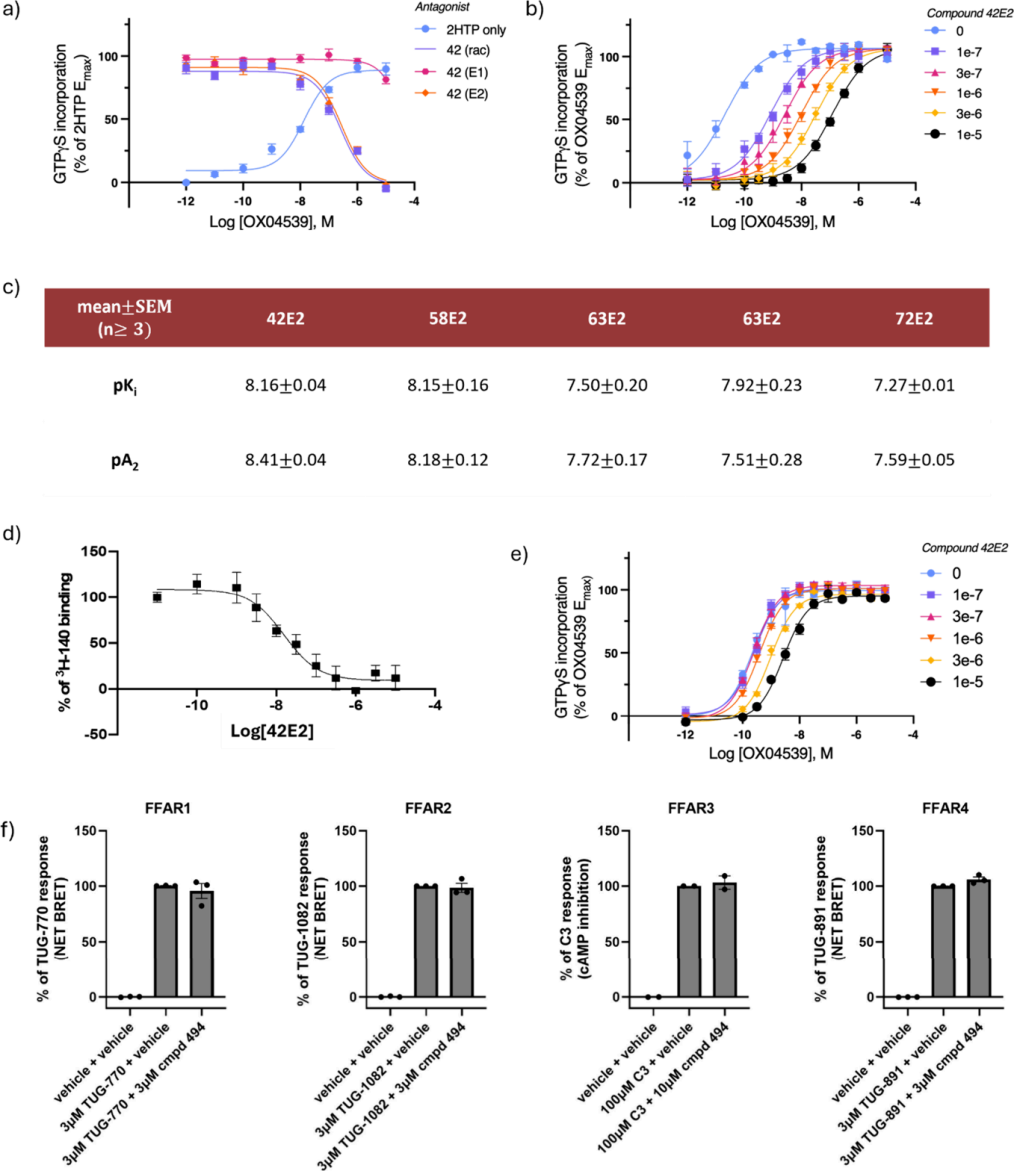
(a) As exemplified by **42**, only the E2 enantiomers displayed antagonism of GPR84 (Schild coefficients ranging between 1.1–1.2 ± 0.01–0.14). (b) Schild analysis of compound **42E2** at human GPR84. (c) p*K*_i_ and p*A*_2_ measurements of the enantiomers isolated from the chromenopyrrole cluster of human GPR84 (*n* = 3). (d) Competition binding assay of **42E2** against the orthosteric antagonist [^3^H]­140 of human GPR84 (*n* = 3). (e) Schild analysis of compound **42E2** at mouse GPR84 (*n* = 3). (f) Screening of **42E2** against FFAR1–4.

Antagonists of the 1,2,4-triazine series have very limited affinity at the murine ortholog of GPR84.^23^ This is due to the conversion of two key residues in mouse GPR84 anticipated to eliminate key H-bond interactions that restrict the shape of the binding pocket. However, **42E2** displayed moderate antagonism at this ortholog, p*A*_2_ = 6.4, offering potential investigations of this series in mGPR84 (Figure 4e).

Although GPR84 is activated by MCFAs, it is not closely related to other members of the FFAR family. Consistent with this, compound 42E2 did not exhibit any significant inhibition of FFAR1, FFAR2, FFAR3, or FFAR4, demonstrating the marked selectivity of this series for GPR84 (Figure 4f).

### 2.5 Drug Metabolism and Pharmacokinetics (DMPK)

The most potent enantiomer, **42E2**, was selected for *in vitro* DMPK evaluation (Table 6). **42E2** exhibited a favorable logD_7.4_ and excellent solubility at physiologically relevant pH. Furthermore, **42E2** displayed an excellent plasma protein binding profile with 23% unbound. However, its relatively short hepatocyte half-life and elevated clearance rates indicate limited metabolic stability. The rapid turnover observed in mouse microsomes suggests metabolism via a predominant P1 pathway.

**Table 6:** *In Vitro* DMPK Properties of Compound **42E2** ^a^

**property**	**42E2**
solubility	286 μM at pH 7.4
logD_7.4_	3.1
mouse PPB	% bound = 77
	% unbound = 23
% remaining MLM (30 min)	3.5
*t*_1/2_ (min)	2
CL_int_ (μL/min/kg)	8376

a Solubility in phosphate buffer pH 7.4 (μM); logD_7.4_ (distribution coefficient); mouse plasma protein binding (mouse PPB); percentage remaining of compound in mouse liver microsomes; half-life of compound in mouse hepatocytes; intrinsic clearance level (CL_int_) in mouse hepatocytes.

## 3 Discussion

Systematic modification of the chromenopyrrole tail revealed a strong dependence on steric and hydrophobic interactions within the orthosteric pocket. Initial analogs (**2**–**12**) bearing linear or small cycloalkyl substituents were inactive, highlighting the intolerance of minimal hydrophobic bulk. Activity emerged with cyclopentylmethyl (**13**, pIC_50_ = 5.49) and improved markedly with cyclohexylmethyl (**14**, pIC_50_ 6.23) and cycloheptylmethyl (**15**, pIC_50_ 6.24), suggesting an optimal lipophilic volume. Larger or highly substituted rings (**25**–**29**) reduced potency, consistent with steric clash. Incorporation of polar functionalities (**22**, **23**) abolished activity, reinforcing the requirement for a nonpolar environment. Electronic effects were also probed via heterocyclic and aromatic substitutions. Oxygen-containing rings (**43**–**47**) and fused benzodioxane derivatives (**49**, **50**) were tolerated only moderately (pIC_50_ ≤ 6.35), indicating that increased polarity compromises binding despite potential π-interactions. Halogenated benzyl analogues (**51**–**58**) revealed positional sensitivity: ortho- and meta-chloro derivatives (**55**, **56**) were inactive, whereas para-chloro substitution restored potency (**57**, pIC_50_ = 5.75; **58**, pIC_50_ 6.63), suggesting electronic modulation of aromatic interactions with Phe101^3.53^ and Phe152^4.57^.

Overall, SAR trends indicate that optimal antagonism requires a balance of hydrophobic bulk and minimal polarity at the tail region. Cyclohexylmethyl emerged as the preferred substituent, while para-halogenation of aromatic tails offers an alternative strategy for enhancing potency. These findings align with docking predictions, which position the lipophilic tail deep within a hydrophobic cleft bordered by Leu182^5.42^, Phe152^4.57^, and Phe101^3.53^ residues, rationalizing the observed steric and electronic constraints.

GPR84, a member of the FFAR subfamily of GPCRs, shares structural similarities with other family members, notably the positioning of extracellular loop 2 (ECL2) extending over the receptor’s orthosteric pocket. FFARs typically contain one or more gatekeeper arginine residues, able to recruit the acidic moiety of fatty acids. This mechanism is essential for the binding of short-chain fatty acids to FFAR2 and FFAR3. In the case of GPR84, MCFA binding is proposed to be facilitated via Arg^172^. Strategically targeting the FFAR gatekeeper residue through replacement of the native acidic moiety with bioisosteres has proven challenging. The size, geometry, and orientation of the carboxylate mimic critically influence a ligand’s ability to achieve high-affinity binding. This factor is hypothesized to contribute to the antagonistic activity observed for compounds **59**–**79**. The orthosteric pocket of GPR84, as revealed by the 6-OAU-bound cryo-EM structure, forms a narrow and well-defined binding cavity. Consequently, Arg^172^ may not play a major role in ligand recruitment for the chromenopyrrole antagonists. Indeed, substitution of Arg^172^ to alanine did not diminish ligand affinity (Supplementary Figure SI-1). These findings suggest that the chromenopyrrole series may access the orthosteric site via an alternative entry route, likely through the transmembrane domains.^15^ As a result, highly polar functionalities are less well tolerated, and an optimal balance between polarity, to enable key binding interactions, and lipophilicity, to facilitate transmembrane tunneling, is required for effective receptor engagement.

As all compounds in the initial SAR series were racemic, the five most potent analogs (**42**, **58**, **62**, **63**, and **72**) were advanced for separation by SFC purification. Detailed pharmacological characterization revealed that the antagonistic activity resided exclusively in a single enantiomer. In combination with molecular modeling, these findings strongly suggest that the active configuration corresponds to the (*S*,*S*,*R*) enantiomer.

The [^35^S]­GTPγS binding assay was selected because it provides a direct, proximal measure of ligand-dependent G-protein activation and thus minimizes signal amplification and pathway bias associated with distal functional readouts. Consistent with this rationale, inhibition of agonist-induced GPR84–arrestin-3 recruitment showed a strong correlation between arrestin IC_50_ values and potencies obtained in the [^35^S]­GTPγS assay. This concordance across mechanistically distinct pathways supports the conclusion that the compounds act as bona fide GPR84 antagonists.

Given its high affinity and potency (p*A*_2_ = 8.41 and p*K*_i_ = 8.16), **42E2** was selected for in-depth pharmacological profiling. The orthosteric nature of **42E2** was confirmed using a competitive [^3^H]-140 binding assay. Evaluation of this analog in the mouse ortholog revealed moderate activity, in contrast to the previously reported orthosteric 1,2,4-triazine antagonist series. Furthermore, **42E2** exhibited no detectable off-target activity against FFAR1–4. Pharmacokinetic profiling demonstrated favorable solubility at physiological pH (7.4) and acceptable plasma protein binding in mouse plasma. However, **42E2** displayed a short half-life in mouse hepatocytes, indicating limited metabolic stability. Moreover, the rapid turnover observed in mouse liver microsomes suggests metabolism via a Phase I pathway. Cytochrome P450 enzymes are the primary mediators of Phase I metabolism, typically targeting inactivated CH bonds.

The tricyclic chromenopyrrole scaffold contains several sp^3^-hybridized CH’s; however, the presence of chromenopyrrole-based therapeutics employed in the clinic indicate that the core scaffold is not inherently metabolically labile. Therefore, it is proposed that the cyclohexyl methylene substituent is the principal contributor to the reduced metabolic stability observed for compound **42E2**.

## 4 Chemistry

A six-step synthesis was undertaken to access pharmacologically active compounds. For amide derivatives **1**–**58**, an initial Williamson ether synthesis using salicylic aldehyde and methyl (*E*)-4-bromobut-2-enoate was performed (Scheme 1). A 1,3-dipolar cycloaddition, employing a Dean and Stark apparatus, afforded the racemic core scaffold bearing a DMB-protected amine and an ethyl ester at the 3-position (**85**). The DMB-protecting group was removed under acidic conditions using TFA. Acetylation or mesylation of the secondary amine was achieved under standard basic conditions. Ester hydrolysis afforded the corresponding carboxylic acids. Late-stage diversification to achieve compounds **1**–**58** was accessed via T3P-mediated amide couplings with the appropriate amine. Compounds **58**–**79** were similarly synthesized via a racemic 1,3-dipolar cycloaddition, differing in the use of more acid-resistant PMB-Gly-OEt. Following formation of the tricyclic core, ester hydrolysis was carried out, with subsequent T3P-mediated amide coupling of cyclohexylamine. TFA-mediated removal of the PMB group afforded the free amine.

**Scheme 1:**
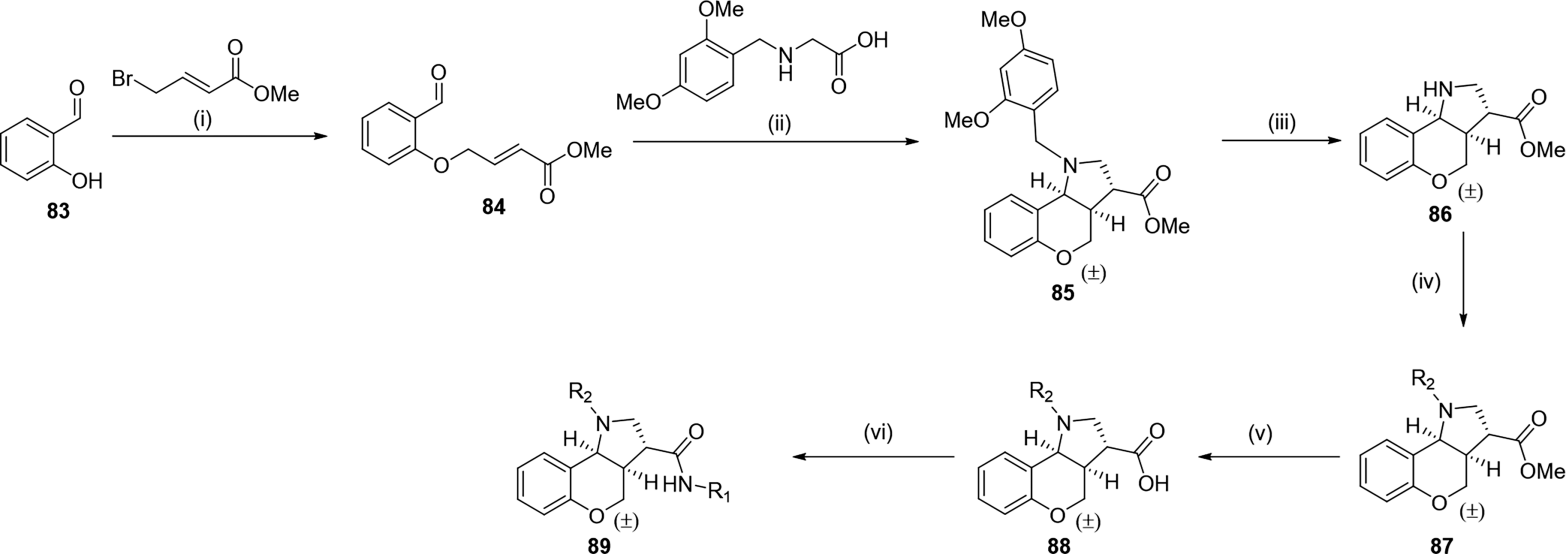
Generalized Scheme of Chromenopyrrole Scaffold Synthesis^a^

a (i) K_2_CO_3_, acetone, r.t., 16 h; (ii) Et_3_N, toluene, reflux, 16 h; (iii) TFA, reflux, 3 h; (iv) R_1_-Cl, DIPEA, CH_2_Cl_2_, 0–25 °C, 4–16 h; (v) 2 M NaOH, MeOH, r.t., 4–16 h; (vi) H_2_N-R_2_, DIPEA, T3P, CH_2_Cl_2_, r.t., 16 h.

Compounds **62**–**68** and **72**–**73** were obtained from the corresponding acetyl or sulfonyl chlorides (Scheme 2). Notably, dropwise addition of the sulfonic acid chloride and methyl sulfamoyl chloride was required to achieve compounds **66** and **67**. Compounds **68**–**71** were prepared via HATU-mediated amide coupling of the corresponding acids (Scheme 3). Boc removal was achieved under standard TFA conditions, affording the free amine **68**. CsF was used as an efficient fluoride source for TBDPS removal, allowing for compound **69**. Treatment of compound **70** with HCl liberated the free diol **71**.

**Scheme 2:**
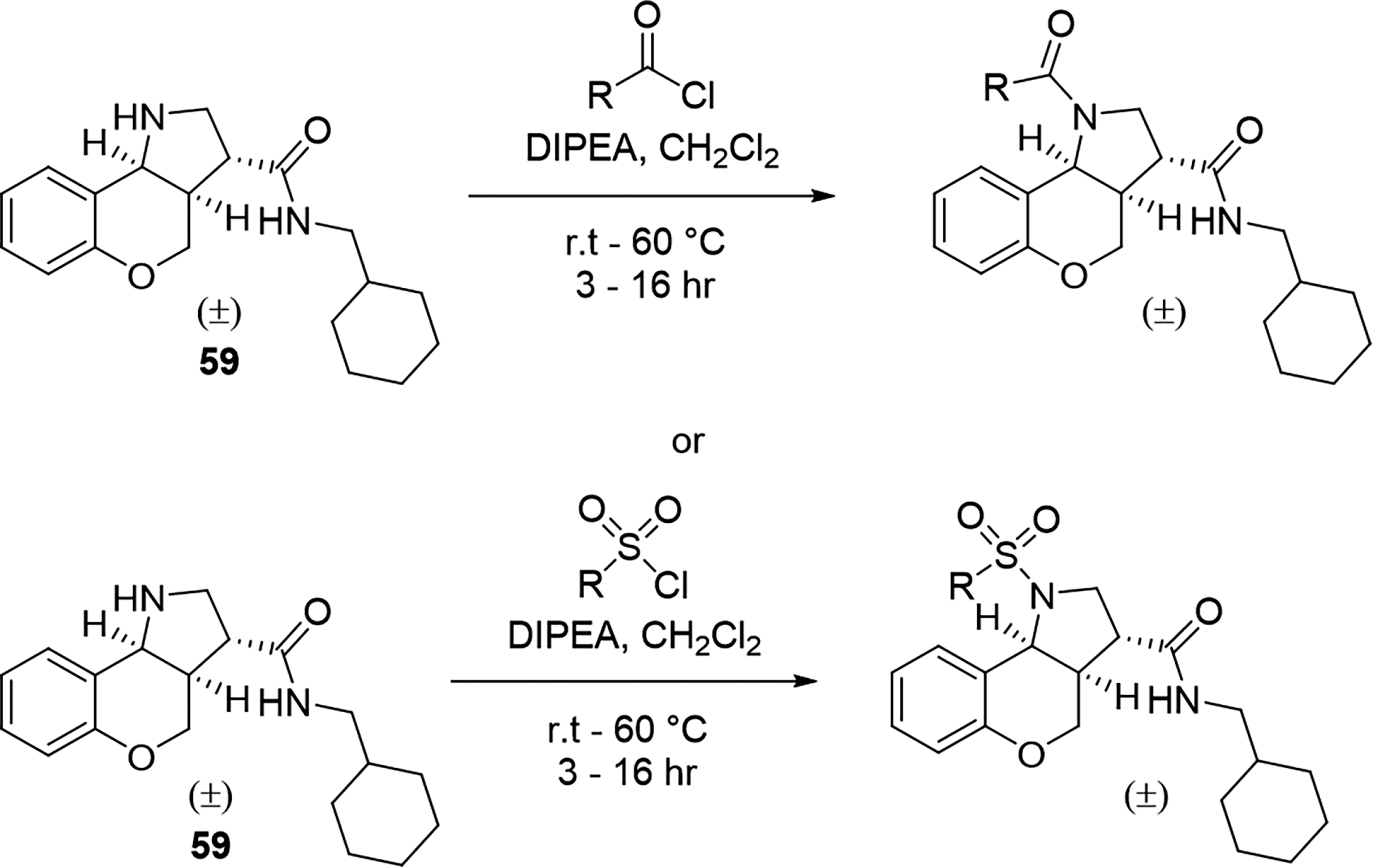
Generalized Scheme for the Synthesis of Compounds **42**–**58** and **62**–**67**

**Scheme 3:**
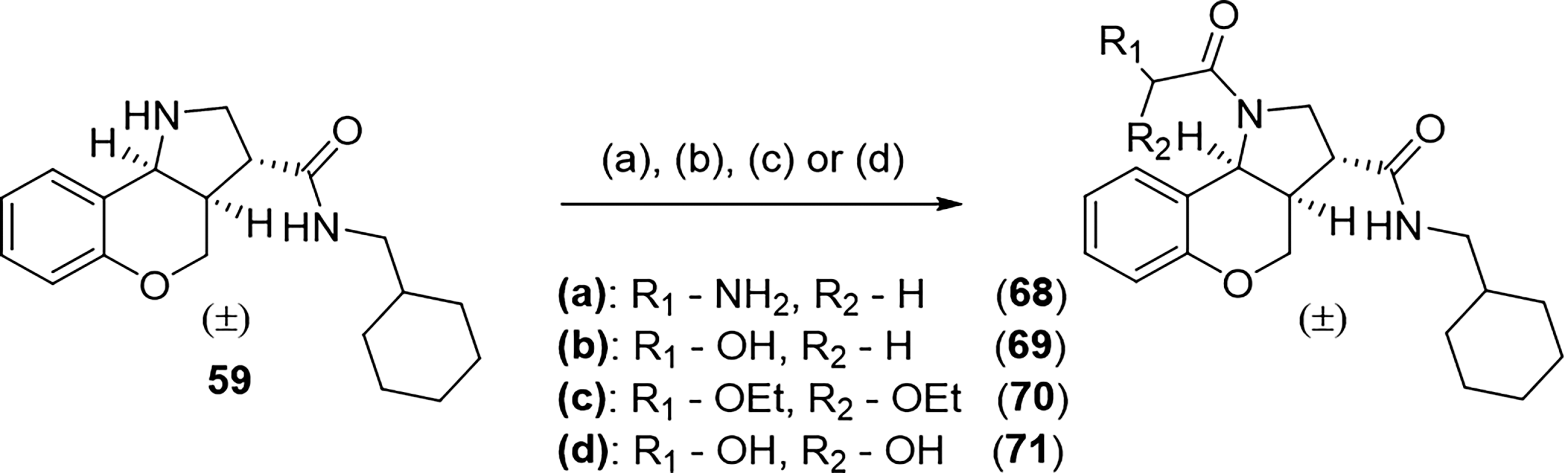
Reaction Conditions to Form Analogues **68**–**71** ^a^

a (a) *N*(Boc)-Glycine, HATU, DIPEA, DMF, then TFA:CH_2_Cl_2_; (b) TBDPS-glycolic acid, HATU, DIPEA, DMF, then CsF, DMF; (c) 2,2-diethoxy acetic acid, HATU, DIPEA, DMF; (d) (i) 2,2-diethoxy acetic acid, HATU, DIPEA, DMF; (ii) HCl, CH_2_Cl_2_.

A radical copper-mediated functionalization of the deprotected core scaffold allowed for the synthesis of urea analogues **74**–**76** (Scheme 4). A DMAP-promoted nucleophilic attack of phenyl *N*-methoxycarbamate gave the methoxy urea, compound **77**. A one-pot CDI-mediated coupling generated the asymmetrical urea **78**. Reduction of **78**, utilizing Pd­(OH)_2_, under standard hydrogenation conditions afforded compound **79**. To obtain analogues **80**–**82**, a seven-step synthesis was performed (Scheme 5). Capping of 2-aminobenzaldehyde, employing TFAA under basic conditions, provided the trifluoroacetyl (**91**). Basic conditions then facilitated a nucleophilic attack on the appropriate alkyl halide to give the desired tertiary amine (**92**). A 1,3-dipolar cycloaddition, using a Dean and Stark apparatus, generated the core DMB-protected tricyclic scaffold (**93**). TFA-mediated DMB removal and subsequent mesylation generated the sulfonamide intermediate (**95**). Global deprotection via 2 M NaOH in MeOH gave the acid. T3P-mediated amide coupling gave compound **80**. Methylation of **80** utilizing MeI provided **81**. Acetylation of **80** was achieved employing acetyl chloride under basic conditions, affording **82**.

**Scheme 4:**
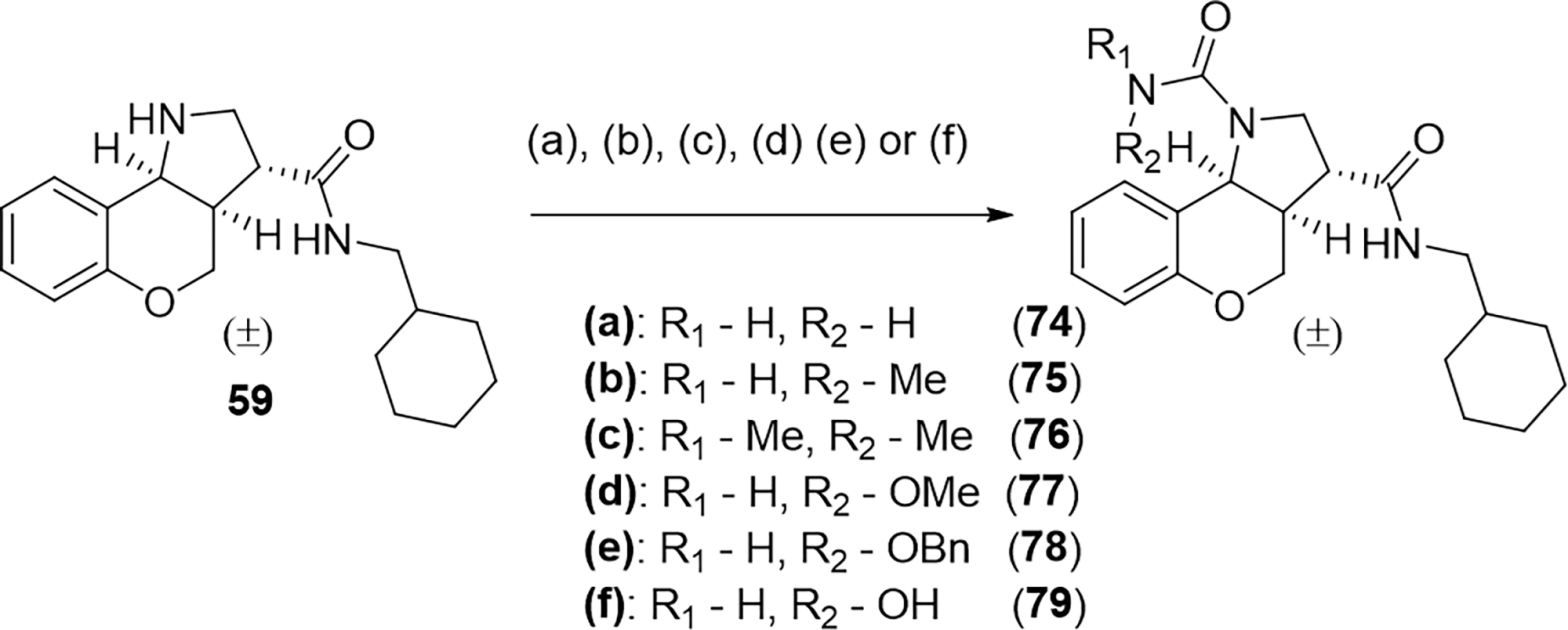
Reaction Conditions to Form Analogues **74**–**79** ^a^

a (a) Formamide, TBHP in dioxane, CuBr_2_; (b) NMF, TBHP in dioxane, CuBr_2_; (c) DMF, TBHP in dioxane, CuBr_2_; (d) phenyl *N*-methoxycarbamate, DMAP, Et_3_N, DMF; (e) *N*-benzyloxyamine, CDI, DIPEA, CH_2_Cl_2_; (f) (i) *N*-benzyloxyamine, CDI, DIPEA, CH_2_Cl_2_; (ii) Pd­(OH)_2_/H_2_, MeOH.

**Scheme 5:**
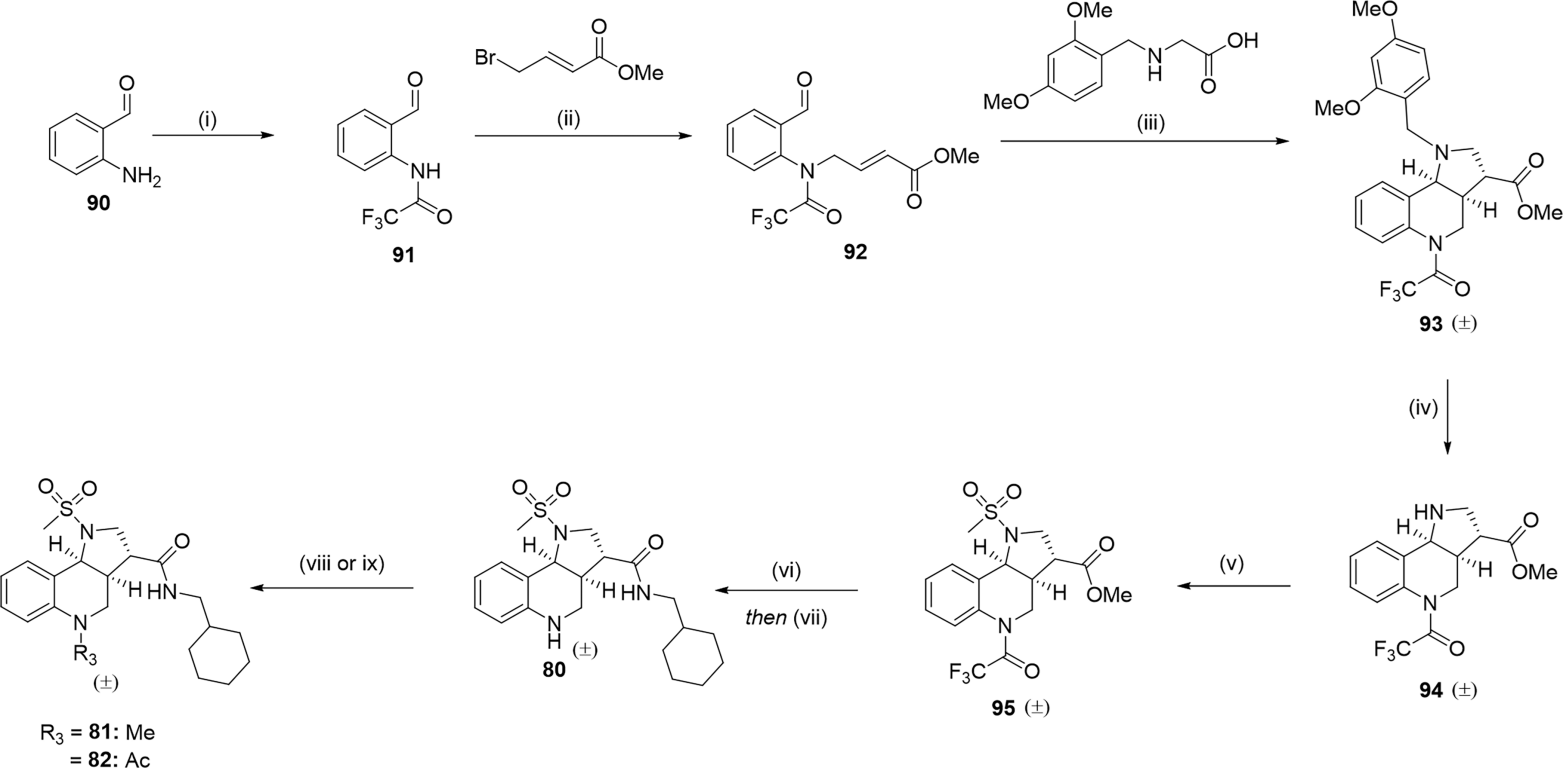
Generalized Scheme of *N*-Chromenopyrrole Scaffold Synthesis **80**–**82** ^a^

a (i) TFAA, pyridine, CH_2_Cl_2_, r.t., 4 h; (ii) NaH, DMF, 60 °C, 4 h; (iii) toluene, reflux, 6 h; (iv) TFA, reflux, 2 h; (v) MsCl, DIPEA, CH_2_Cl_2_, 0–25 °C, 1 h; (vi) 2 M NaOH, MeOH, r.t., 4 h; (vii) DIPEA, T3P, CH_2_Cl_2_, r.t., 2 h; (viii) formaldehyde, acetic acid, STAB, MeOH, r.t., 1 h; (ix) AcCl, DIPEA, CH_2_Cl_2_, r.t., 16 h.

## 5 Conclusions

Selective small-molecule antagonists targeting GPR84 offer a promising route for the treatment of inflammatory disorders. The availability of potent, well-characterized antagonists to probe the structure and function of GPR84 will be essential to fully elucidate its therapeutic potential. We report here the identification of a racemic chromenopyrrole orthosteric antagonist series. Following hit identification, pharmacological characterization of compound **1**, guided by molecular modeling, enabled a structure-based exploration of this chemotype. Molecular design focused on three areas of modification: substitution of the secondary amine, functionalization of the chromenopyrrole acid moiety, and alteration of the cyclic ether within the core scaffold. The five most potent antagonists were advanced to chiral SFC purification. Comprehensive pharmacological evaluation identified E2 as the sole active enantiomer, with molecular modeling suggesting a (*S*,*S*,*R*) stereochemical configuration. Lead enantiomer, **42E2**, was identified with p*A*_2_ = 8.41 and p*K*_i_ = 8.16 and was subsequently advanced to *in vitro* pharmacokinetic profiling. Compound **42E2** exhibited optimal solubility at pH 7.4, excellent mouse plasma protein binding, and a balanced logD_7.4_. However, short hepatocyte half-life indicates the need for further optimization to improve metabolic stability within this series. Collectively, these studies provide a robust platform for the continued development of selective GPR84 antagonists and offer valuable insights into the structural requirements for high-affinity orthosteric inhibition.

## 6 Experimental Section

### 6.1 General Information

All starting materials were purchased commercially and used without further purification unless stated otherwise. All solvents were reagent grade and used as received. Anhydrous solvents were obtained by passage through a solvent filtration system (PureSolv) and were transferred via a syringe. Celite was purchased from Acros Organics or Fisher. Hydrophobic frits and SCX columns were purchased from Telos or Biotage. Telos columns were purchased from Telos. Daicel OJ-H columns and Daicel AD-H columns were purchased from Daicel. Reacti-Vials were purchased from Thermo Fisher. Thin-layer chromatography was performed on silica gel 60 F254 aluminum plates from Merck. Readings of the TLC plates were obtained via the use of a UV lamp (254 nm) or treatment with potassium permanganate. Chromatographic purifications were performed using prepacked Telos silica columns using a Biotage Isolera Purification system (Uppsala, Sweden). The deuterated solvent was obtained from Cambridge Isotopes. Proton nuclear magnetic resonance spectra (^1^H) were recorded on AVANCE III 400 Bruker 400 MHz. Chemical shifts are expressed in parts per million (ppm) and are referenced to the deuterated NMR solvent ^1^H (CDCl_3_, δ 7.26, CD_3_OD, δ 3.31). The following abbreviations were used to describe signal splitting from the recorded ^1^H NMR spectra: s = singlet, d = doublet, t = triplet, q = quartet, m = multiplet. Coupling constants (J values) were measured in hertz (Hz). Accurate mass measurements were obtained via high-resolution mass spectrometry (HRMS) performed on a Bruker microTOF-Q II using positive mode electrospray ionization (ESI+). Analyses by liquid chromatography–mass spectrometry (LC-MS) were performed on a Thermo Scientific LCQ Fleet Ion Trap Mass Spectrometer (ESI+), where buffer A = 0.1% TFA in H_2_O and buffer B = 0.1% TFA in MeCN. HPLC analysis was performed on a Shimadzu RP-HPLC system equipped with Shimadzu LC-20AT pumps, a Shimadzu SIL20A autosampler, and a SPS-20A UV–vis detector using a Phenomenex Aeris column (5 mm C18, 100 Å, 150 × 10 mm) at a flow rate of 1 mL/min. Reactions under microwave radiation were performed on Biotage Initiator Sixty under standard operating conditions. The purity of all final compounds was >95%, as determined by liquid chromatography mass spectrometry (LC-MS). The purity of all biologically evaluated compounds was greater than 95%.

### 6.2 Biological Procedures

#### 6.2.1 [^35^S]­GTPγS Incorporation Assay

The GTPγS functional assay was performed according to the published procedures.^23^ A brief description of the binding assay is given below. Flp-ln *T*REx 293 cells (lnvitrogen) were maintained in Dulbecco’s modified Eagle’s medium without sodium pyruvate (lnvitrogen), supplemented with 10% (w/v) fetal calf serum, 1% penicillin/streptomycin mixture, and 10 μg/mL blasticidin at 37 °C in a 5% CO_2_ humidified atmosphere. To generate Flp-ln *T*REx 293 cells capable of inducibly expressing the GPR84 receptor constructs, the cells were transfected with a mixture containing the desired cDNA in pcDNA5/FRT/TO vector and pOG44 vector (1:9) using 1 mg/mL polyethylenimine (PEI) (molecular weight (MW) 25,000). Cells were grown until 60–80% confluent then transfected with 8 μg of required plasmid DNA and PEI (ratio 1:6 DNA/PEl) and diluted in 150 mM NaCl, pH 7.4. After incubation at room temperature for 10 min, the mixture was added to cells. After 48 h, the medium was changed to a medium supplemented with 200 μg/mL hygromycin B to initiate the selection of stably transfect cells. After isolation of resistant cells, expression of the appropriate construct from the Flp-ln *T*REx locus was induced by treatment with up to 100 ng/mL doxycycline for 24 h. Membrane proteins were generated from Flp-In T-REx HEK293 cells treated with 100 ng/mL doxycycline to induce expression of the receptor construct of interest. The cells were washed with ice-cold phosphate-buffered saline, removed from dishes by scraping, and centrifuged at 3000 rpm for 5 min at 4 °C. Pellets were resuspended in TE buffer (10 mM Tris-HCl, 0.1 mM ethylenediaminetetraacetic acid (EDTA); pH 7.5) containing a protease inhibitor mixture (Roche Applied Science West Sussex, U.K.) and homogenized with a 5 mL hand-held homogenizer. This material was centrifuged at 1500 rpm for 5 min at 4 °C, and the supernatant was further centrifuged at 50,000 rpm for 45 min at 4 °C. The resulting pellet was resuspended in TE buffer, and the protein content was assessed using a bicinchoninic acid (BCA) protein assay kit (Pierce, Fisher Scientific, Loughborough, U.K.). The following assay can be used for the determination of GPR84 activation. The guanosine 5′-*O*-[γ-thio]­triphosphate ([^35^S]­GTPγS) functional assay measures the level of G-protein activation following agonist occupation of a GPCR, by determining the binding of the poorly hydrolyzable analogue [^35^S]­GTPγS to Gα subunits. Initially, 5 μg of generated membrane protein was preincubated for 15 min at 25 °C in assay buffer (20 mM *N*-(2-hydroxyethyl)­piperazine-*N*′-ethanesulfonic acid (HEPES), 5 mM MgCl2; 160 mM NaCl; 1 μM GDP; 0.05% fatty acid-free bovine serum albumin; pH 7.5) containing the indicated ligand agonist concentrations. To assess inhibition of agonist stimulation, membrane preparations were preincubated with antagonist compound for 15 min at room temperature prior to the addition of agonist. The reaction was then initiated with the addition of [^35^S]­GTPγS (50 nCi per tube), and the reaction was terminated after 45 min incubation at 30 °C by rapid filtration through GF/C glass filters using a 24-well Brandel cell harvester (Alpha Biotech, Glasgow, U.K.). Unbound radioligand was removed from filters by three washes with ice-cold phosphate-buffered saline (pH 7.4), and filters were dried for 2–3 h at room temperature. The dried filters were added to 3 mL of Ultima GoldTM XR (PerkinElmer Life Sciences, Beaconsfield, U.K.), and [^35^S]­GTPγS binding was determined by liquid scintillation spectrometry.

#### 6.2.2 Bioluminescence Resonance Energy Transfer (BRET) Arrestin Recruitment Assays

HEK293T cells were transiently cotransfected with a receptor-eYFP fusion construct and arrestin-2 or arrestin-3 fused to nanoluciferase, in a ratio of 100:1. As a control, cells were transfected with a 100:1 ratio of empty plasmid and the appropriate arrestin isoform fused to nanoluciferase. After 24 h, cells were seeded at 50,000 cells per well in poly-d-lysine-coated white 96-well plates and incubated at 37 °C overnight. After 24 h, cells were washed twice with Hank’s balanced salt solution (HBSS), pH 7.4, and then preincubated for 5 min with 10 μL/well of antagonist compounds before addition of 10 μL/well of the nanoluciferase substrate coelenterazine-h (Nanolight Technologies) at a final concentration of 5 μM. Cells were incubated in the dark for a further 10 min at 37 °C, after which 10 μL/well of the agonist compound was added, and the cells were incubated for another 5 min. Fifteen min after coelenterazine-h addition, reading of emission signals was performed on a PHERAstar FS plate reader (BMG Labtech) at 475 and 535 nm, representing nanoluciferase and eYFP emission signals, respectively. The net bioluminescence resonance energy transfer (BRET) ratio (mBRET) was calculated as follows: [(signal 535 nm/signal 475 nm) – (signal nanoluc luciferase only 535 nm/signal nanoluc luciferase only 475 nm)] × 1000.

#### 6.2.3 HTRF-Based cAMP Inhibition Assays

cAMP experiments were performed using Flp-In T-REx293 cells induced to express the receptor construct of interest. Experiments were carried out using a homogeneous time-resolved FRET-based detection kit (Revvity) according to the manufacturer’s protocol. For the assay, cells were plated at 5000 cells/well in low-volume 384-well plates. The ability of agonists to inhibit 1 μM forskolin-induced cAMP production was assessed following a preincubation for 15 min with antagonist compounds and then a further 1 h incubation with agonist compounds. Reactions were stopped according to the manufacturer’s instructions, and the output was measured with a PHERAstar FS plate reader (BMG Labtech).

#### 6.2.4 Competition Binding Assays

Competition binding experiments using approximate *K*_d_ concentrations of [^3^H]­140 were conducted in binding buffer (phosphate-buffered saline (PBS) with 0.5% fatty acid-free bovine serum albumin; pH 7.4) in a total assay volume of 500 μL in 96 deep-well blocks. Binding was initiated by the addition of 5 μg membrane protein generated from Flp-In T-REx cells induced to express the receptor construct of interest with 100 ng/mL doxycycline. Nonspecific binding of the radioligand was determined in the presence of 10 μM compound 837. All assays were performed at 25 °C for 1 h before termination by the addition of ice-cold PBS and vacuum filtration through GF/C glassfibre filter-bottom 96-well microplates using a UniFilter FilterMate Harvester (Revvity). Each well was washed three times with ice-cold PBS, and filters were allowed to dry for 2–3 h before addition of 50 μL of Ultima Gold XR. Radioactivity was quantified by liquid scintillation spectrometry.

#### 6.2.5 Data Analysis

All data are presented as means ± SEM of at least three independent experiments. Data analysis and curve fitting were carried out using the GraphPad Prism software package version 10 (GraphPad, San Diego). For functional assays, the concentration–response data were plotted on a log axis, with the untreated vehicle control plotted at 1 log unit lower than the lowest ligand concentration, and fitted to a three-parameter sigmoidal curve with the Hill slope constrained to equal 1. In the case of inhibition experiments with antagonists, an equivalent analysis was followed to fit an inverse sigmoidal curve. Antagonism experiments carried out with multiple defined concentrations of antagonist were fit to a global Gaddum/Schild EC50 shift equation to estimate p*A*_2_ values for the antagonist.

### 6.3 Molecular Docking

A homology model for structure-based design of the chromenopyrrole antagonist series was developed based on the 6-OAU-bound cryo-EM structure of the human GPR84–Gi complex (PDB: 8G05). To reduce computational cost, all G-protein subunits were removed from the cryo-EM structure as they are distant from the orthosteric binding site. Intracellular loop 3 (ICL3), which is unresolved in the cryo-EM structure and located far from the binding pocket, was modeled as a 10-residue loop. The agonist-bound structure was relaxed in the absence of 6-OAU using short (1000 steps) implicit solvent molecular dynamics simulations of the MacroModel module (Schrödinger Suite 2020-1), followed by energy minimization to expand the binding pocket and accommodate bulkier antagonist scaffolds.

The AlphaFold-predicted structure of human GPR84 was obtained from the AlphaFold Protein Structure Database (https://alphafold.ebi.ac.uk/, UniProt ID: Q9NQS5). This model, representing an inactive receptor conformation, was used in parallel with the modified homology model for docking studies.

Both receptor structures were prepared using the Protein Preparation Wizard in Schrödinger Suite 2020-1. This protocol included assignment of bond orders, addition of hydrogen atoms, optimization of hydrogen bond networks, and restrained minimization using the OPLS_2005 force field. Ligand structures were prepared using the LigPrep module, generating appropriate ionization states at pH 7.4 ± 1.0 and tautomeric forms. All possible stereoisomers were generated for racemic compounds. Docking calculations were performed using Glide (Schrödinger Suite 2020-1) in standard precision (SP) mode. The receptor grid was centered on the orthosteric binding site using key residues Tyr69^2.53^, Phe101^3.53^, Arg172^ECL2^, Phe335^7.35^, and Trp360^7.43^ to define the grid center. To accommodate binding of bulky antagonist analogs, three sets of receptor grids were generated with van der Waals radius scaling factors of 1.0, 0.9, and 0.8 for receptor atoms. The default Glide SP docking protocol was used, with no constraints applied. Up to 10 poses per ligand were retained for analysis. Docking poses were evaluated based on Glide docking scores and visual inspection of predicted binding modes. The most frequently observed binding mode across structural analogs was selected as the most probable binding pose for the series. Selected protein–ligand complexes were subjected to restrained energy minimization using the MacroModel module (Schrödinger Suite 2020-1) with the OPLS_2005 force field. Minimization was performed using the default protocol in an implicit solvent (generalized Born/surface area continuum solvation model) with heavy atoms of the receptor constrained to allow relaxation of side-chain conformations in the binding site. Three-dimensional representations of protein–ligand complexes were generated using Maestro 2020-1 (Schrödinger, LLC).

### 6.4 Chemistry Procedures

#### 6.4.1 Method A: The General Method for Amine Functionalization

Methyl-rac-(3*R*,3*aR*,9*bS*)-1,2,3,3*a*,4,9*b*-hexahydrochromeno­[4,3-*b*]­pyrrole-3-carboxylate (8.2 mmol, 1.0 equiv) and DIPEA (16.4 mmol, 2.0 equiv) were dissolved in dry CH_2_Cl_2_ (30.0 mL) under N_2_ gas. The solution was cooled in an ice bath. The required chloride (9.1 mmol, 1.1 equiv) was added dropwise. The mixture was left stirring in the ice bath for 30 min followed by 2–16 h at room temperature under N_2_ gas. CH_2_Cl_2_ (50 mL) was added to the reaction mixture and washed with NaHCO_3_ (50 mL). The aqueous layer was back extracted with CH_2_Cl_2_ (50 mL). The organic layers were combined, dried over Na_2_SO_4_, filtered, and concentrated *in* *vacuo.* The residue was then purified by flash column chromatography (0–10% MeOH in CH_2_Cl_2_) to give the desired product.

#### 6.4.2 Method B: The General Method for Ester Hydrolysis

The required chromeno­[4,3-*b*]­pyrrole-3-carboxylate (7.1 mmol, 1.0 equiv) was suspended in a solution of MeOH (20 mL) and 2 M NaOH (14.2 mmol, 2.0 equiv) and stirred at room temperature for 2 h. The solution was neutralized with 5 M HCl and concentrated down. The residue was partitioned between H_2_O (100 mL) and EtOAc (3 × 50 mL). The organics were combined, dried over Na_2_SO_4_, filtered, and concentrated down *in* *vacuo*, affording the desired product.

#### 6.4.3 Method C: The General Method for Amide Coupling

The corresponding chromeno­[4,3-*b*]­pyrrole-3-carboxylic acid (0.033 mmol, 1.0 equiv) was mixed in CH_2_Cl_2_ (1.0 mL), the T3P (50.0%, 0.10 mmol, 3.0 equiv) was added, and the resulting solution was stirred for 30 min. The required amine (0.09 mmol, 2.7 equiv) and DIPEA (0.20 mmol, 6.0 equiv) in CH_2_Cl_2_ (0.5 mL) were added, and the solution was stirred overnight. The mixture was partitioned between H_2_O (5.0 mL) and CH_2_Cl_2_ (5.0 mL) and passed through a hydrophobic frit. The organics were concentrated URP and then purified by prep HPLC (XBridge column, 0.1% NH_4_OH modifier).

#### 6.4.4 Method D: The General Method for Urea Formation **74**–**76**

A solution of chromeno­[4,3-*b*]­pyrrole-3-carboxylic acid (50 mg, 0.2 mmol, 1 equiv) and CuBr_2_ (2 mg, 0.01 mmol, 0.05 equiv) in 0.4 mL of the corresponding formamide solution was stirred at room temperature. To the same solution, a 5–6 M TBHP solution in decane (0.17 mL, 0.3 mmol, 1.5 equiv) was added dropwise and stirred for 1 h. The crude mixture was then partitioned between EtOAc (5 mL) and H_2_O (5 mL) and washed 3x with EtOAc (3 × 10 mL). The organic layers were combined, dried over Na_2_SO_4_, filtered, and concentrated *in vacuo*. The residues were prepared by flash column chromatography (7% MeOH in CH_2_Cl_2_).

### 6.5 Characterization Data

#### 6.5.1 Rac-(3*R*,3*aR*,9*bS*)-1-methylsulfonyl-*N*-(2-propoxyethyl)-3,3*a*,4,9*b*-tetrahydro-2*H*-chromeno­[4,3-*b*]­pyrrole-3-carboxamide (**1**)

Prepared according to method C. The residue was purified by prep HPLC to afford a white solid, yield 45.5 mg (71%). ^1^H NMR (400 MHz, CDCl_3_) δ 7.69–7.79 (m, 1 H), 7.14–7.25 (m, 1 H), 6.96–7.08 (m, 1 H), 6.75–6.88 (m, 1 H), 6.00–6.23 (m, 1 H), 5.07–5.23 (m, 1 H), 4.15–4.28 (m, 2 H), 3.70–3.79 (m, 1 H), 3.56–3.63 (m, 1 H), 3.53 (s, 4 H), 3.43 (s, 2 H), 3.10–3.20 (m, 1 H), 3.05 (s, 3 H), 2.86–2.95 (m, 1 H), 1.61 (s, 4 H), 0.95 (s, 3 H), *m*/*z* calcd for C_18_H_26_N_2_O_5_S [M+H^+^] 382.2, found 383.0.

#### 6.5.2 Rac-(3*R*,3*aR*,9*bS*)-*N*-methylsulfonyl-3,3*a*,4,9*b*-tetrahydro-2*H*-chromeno­[4,3-*b*]­pyrrole-3-carboxamide (**2**)

Prepared according to method C. The residue was purified by prep HPLC to afford a white solid, yield 18.0 mg (68%). ^1^H NMR (400 MHz, DMSO-*d*_6_) δ 8.11–8.28 (m, 1H), 7.49–7.63 (m, 1H), 7.12–7.24 (m, 1H), 6.92–7.06 (m, 1H), 6.67–6.86 (m, 1H), 5.08–5.22 (m, 1H), 4.01–4.21 (m, 2H), 3.35–3.48 (m, 2H), 3.10 (s, 6H), 2.76–2.86 (m, 1H), 1.41 (t, J = 6.53 Hz, 2H), 1.21–1.33 (m, 6H), 0.86 (t, J = 6.78 Hz, 3H), *m*/*z* calcd for C_19_H_28_N_2_O_4_S [M+H^+^] 380.2, found 381.2.

#### 6.5.3 Rac-(3*R*,3*aR*,9*bS*)-1-methylsulfonyl-*N*-(4,4,4-trifluorobutyl)-3,3*a*,4,9*b*-tetrahydro-2*H*-chromeno­[4,3-*b*]­pyrrole-3-carboxamide (**3**)

Prepared according to method C. The residue was purified by prep HPLC to afford a white solid, yield 20 mg (73%). ^1^H NMR (400 MHz, CDCl_3_) δ 7.63–7.78 (m, 1 H), 7.14–7.24 (m, 1 H), 6.95–7.08 (m, 1 H), 6.72–6.86 (m, 1H), 5.79–6.04 (m, 1H), 5.08–5.21 (m, 1H), 4.11–4.27 (m, 2H), 3.66–3.77 (m, 1H), 3.51–3.61 (m, 1H), 3.41 (d, J = 6.78 Hz, 2H), 3.08–3.19 (m, 1H), 3.04 (s, 3H), 2.84–2.95 (m, 1H), 2.08–2.24 (m, 2H), 1.85 (s, 2H), *m*/*z* calcd for C_17_H_21_F_3_N_2_O_4_S [M+H^+^] 406.1, found 405.0.

#### 6.5.4 Rac-(3*R*,3*aR*,9*bS*)-*N*-isopentyl-1-methylsulfonyl-3,3*a*,4,9*b*-tetrahydro-2*H-*chromeno­[4,3-*b*]­pyrrole-3-carboxamide (**4**)

Prepared according to method C. The residue was purified by prep HPLC to afford a white solid, yield 18.0 mg (71.6%). ^1^H NMR (400 MHz, DMSO-*d*_6_) δ 8.10–8.26 (m, 1H), 7.50–7.62 (m, 1H), 7.12–7.25 (m, 1H), 6.91–7.04 (m, 1H), 6.72–6.85 (m, 1H), 5.06–5.19 (m, 1H), 3.99–4.21 (m, 2H), 3.35–3.48 (m, 2H), 3.10 (s, 5H), 2.76–2.86 (m, 1H), 1.50–1.62 (m, 1H), 1.26–1.36 (m, 2H), 0.87 (d, J = 6.78 Hz, 6H), *m*/*z* calcd for C_18_H_26_N_2_O_4_S [M+H^+^] 366.2, found 367.0.

#### 6.5.5 Rac-(3*R*,3*aR*,9*bS*)-*N*-isobutyl-1-methylsulfonyl-3,3*a*,4,9*b*-tetrahydro-2*H*-chromeno­[4,3-*b*]­pyrrole-3-carboxamide (**5**)

Prepared according to method C. The residue was purified by prep HPLC to afford a white solid, yield 15.9 mg (67%). ^1^H NMR (400 MHz, CDCl_3_) δ 7.68–7.78 (m, 1 H), 7.14–7.25 (m, 1H), 6.96–7.07 (m, 1 H), 6.74–6.85 (m, 1 H), 5.74–5.89 (m, 1 H), 5.08–5.20 (m, 1H), 4.21 (d, J = 1.76 Hz, 2 H), 3.67–3.78 (m, 1 H), 3.52–3.63 (m, 1 H), 3.16 (s, 3 H), 3.05 (s, 3 H), 2.85–2.96 (m, 1 H), 1.74–1.93 (m, 1 H), 0.95 (d, J = 6.78 Hz, 6 H), *m*/*z* calcd for C_17_H_24_N_2_O_4_S [M+H^+^] 352.2, found 353.0.

#### 6.5.6 Rac-(3*R*,3*aR*,9*bS*)-*N*-(2,2-dimethylpropyl)-1-methylsulfonyl-3,3*a*,4,9*b*-tetrahydro-2*H*-chromeno­[4,3-*b*]­pyrrole-3-carboxamide (**6**)

Prepared according to method C. The residue was purified by prep HPLC to afford a white solid, yield 17.2 mg (70%). ^1^H NMR (400 MHz, CDCl_3_) δ 7.69–7.78 (m, 1H), 7.16–7.24 (m, 1H), 6.97–7.08 (m, 1H), 6.74–6.88 (m, 1H), 5.71–5.85 (m, 1 H), 5.10–5.20 (m, 1H), 4.22 (d, J = 1.76 Hz, 1H), 3.69–3.80 (m, 1H), 3.51–3.66 (m, 1H), 3.15 (dd, J = 6.53, 1.51 Hz, 3 H), 3.05 (s, 3H), 2.86–2.99 (m, 1H), 0.95 (s, 9 H), *m*/*z* calcd for C_18_H_26_N_2_O_4_S [M+H^+^] 366.2, found 367.0.

#### 6.5.7 Rac-(3*R*,3*aR*,9*bS*)-*N*-(2-ethylbutyl)-1-methylsulfonyl-3,3*a*,4,9*b*-tetrahydro-2*H*-chromeno­[4,3-*b*]­pyrrole-3-carboxamide (**7**)

Prepared according to method C. The residue was purified by prep HPLC to afford a white solid, yield 18.5 mg (72%). ^1^H NMR (400 MHz, CDCl_3_) δ 7.68–7.79 (m, 1H), 7.14–7.25 (m, 1H), 6.95–7.07 (m, 1H), 6.75–6.86 (m, 1H), 5.62–5.79 (m, 1 H), 5.09–5.19 (m, 1H), 4.15–4.26 (m, 2H), 3.67–3.78 (m, 1H), 3.54–3.64 (m, 1 H), 3.29 (s, 2H), 3.08–3.17 (m, 1H), 3.05 (s, 3H), 2.85–2.98 (m, 1H), 1.45–1.49 (m, 5H), 0.93 (t, J = 7.40 Hz, 6 H), *m*/*z* calcd for C_19_H_28_N_2_O_4_S [M+H^+^] 380.5, found 381.2.

#### 6.5.8 Rac-(3*R*,3*aR*,9*bS*)-*N*-(cyclopropylmethyl)-1-methylsulfonyl-3,3*a*,4,9*b*-tetrahydro-2*H*-chromeno­[4,3-*b*]­pyrrole-3-carboxamide (**8**)

Prepared according to method C. The residue was purified by prep HPLC to afford a white solid, yield 12.3 mg (52%). ^1^H NMR (400 MHz, CDCl_3_) δ 7.68–7.77 (m, 1 H), 7.14–7.23 (m, 1 H), 6.96–7.06 (m, 1 H), 6.76–6.84 (m, 1 H), 5.78–5.92 (m, 1 H), 5.08–5.17 (m, 1 H), 4.15–4.26 (m, 2 H), 3.68–3.78 (m, 1 H), 3.51–3.63 (m, 1 H), 3.07–3.23 (m, 3 H), 3.04 (s, 3 H), 2.84–2.95 (m, 1 H), 0.91–1.04 (m, 1 H), 0.49–0.60 (m, 2 H), 0.18–0.27 (m, 2 H), *m*/*z* calcd for C_17_H_22_N_3_O_4_S [M+H^+^] 350.1, found 351.0.

#### 6.5.9 Rac-(3*R*,3*aR*,9*bS*)-*N*-[92,2-dimethylcyclopropyl)­methyl]-1-methylsulfonyl-3,3*a*,4,9*b*-tetrahydro-2*H*-chromeno­[4,3-*b*]­pyrrole-3-carboxamide (**9**)

Prepared according to method C. The residue was purified by prep HPLC to afford a white solid, yield 18.0 mg (69.3%). ^1^H NMR (400 MHz, DMSO-*d*_6_) δ 8.18–8.33 (m, 1H), 7.50–7.62 (m, 1H), 7.10–7.23 (m, 1H), 6.91–7.05 (m, 1H), 6.75–6.85 (m, 1H), 5.08–5.20 (m, 1H), 4.01–4.23 (m, 2H), 3.36–3.50 (m, 2H), 3.10 (s, 6H), 2.76–2.87 (m, 1H), 0.97–1.08 (m, 6H), 0.67–0.80 (m, 1H), 0.40 (dd, J = 4.02, 8.53 Hz, 1H), 0.08 (t, J = 4.64 Hz, 1H), *m*/*z* calcd for C_19_H_26_N_2_O_4_S [M+H^+^] 378.2, found 379.4.

#### 6.5.10 Rac-(3*R*,3*aR*,9*bS*)-N-[(2,2-dichlorocyclopropyl)­methyl]-1-methylsulfonyl-3,3*a*,4,9*b*-tetrahydro-2*H*-chromeno­[4,3-*b*]­pyrrole-3-carboxamide (**10**)

Prepared according to method C. The residue was purified by prep HPLC to afford a white solid, yield 21.8 mg (77%). ^1^H NMR (400 MHz, CDCl_3_) δ 7.64–7.79 (m, 1H), 7.15–7.25 (m, 1H), 7.02 (s, 1H), 6.76–6.87 (m, 1H), 6.07–6.23 (m, 1H), 5.18 (d, J = 7.78 Hz, 1H), 4.22 (d, J = 2.01 Hz, 2H), 3.87–4.02 (m, 1H), 3.71–3.83 (m, 1H), 3.53–3.68 (m, 1H), 3.01–3.06 (m, 5H), 2.86–2.97 (m, 1H), 1.86–2.07 (m, 1H), 1.67–1.77 (m, 1H), 1.24–1.39 (m, 1H), *m*/*z* calcd for C_17_H_20_Cl_2_N_2_O_2_S [M+H^+^] 418.1, found 419.0.

#### 6.5.11 Rac-(3*R*,3*aR*,9*bS*)-*N*-[(3,3-dimethylcyclobutyl)­methyl]-1-methylsulfonyl-3,3*a*,4,9*b*-tetrahydro-2*H*-chromeno­[4,3-*b*]­pyrrole-3-carboxamide (**11**)

Prepared according to method C. The residue was purified by prep HPLC to afford a white solid, yield 18.0 mg (67%). ^1^H NMR (400 MHz, DMSO-*d*_6_) δ 8.11–8.26 (m, 1H), 7.50–7.63 (m, 1H), 7.12–7.26 (m, 1H), 6.93–7.06 (m, 1H), 6.71–6.87 (m, 1H), 5.06–5.21 (m, 1H), 4.00–4.22 (m, 2H), 3.34–3.47 (m, 2H), 3.12–3.21 (m, 1H), 3.09 (s, 4H), 2.76–2.85 (m, 1H), 2.27–2.39 (m, 1H), 1.68–1.78 (m, 2H), 1.46 (t, J = 9.91 Hz, 2H), 1.10 (s, 3H), 1.02 (s, 3H), *m*/*z* calcd for C_20_H_28_N_2_O_4_S [M+H^+^] 392.2, found 393.0.

#### 6.5.12 Rac-(3*R*,3*aR*,9*bS*)-*N*-[(3,3-difluorocyclobutyl)­methyl]-1-methylsulfonyl-3,3*a*,4,9*b*-tetrahydro-2*H*-chromeno­[4,3-*b*]­pyrrole-3-carboxamide (**12**)

Prepared according to method C. The residue was purified by prep HPLC to afford a white solid, yield 18.0 mg (66%). ^1^H NMR (400 MHz, DMSO-*d*_6_) δ 8.30–8.47 (m, 1H), 7.49–7.61 (m, 1H), 7.11–7.23 (m, 1H), 6.92–7.04 (m, 1H), 6.73–6.84 (m, 1H), 5.07–5.22 (m, 1H), 4.03–4.24 (m, 2H), 3.36–3.51 (m, 2H), 3.15–3.28 (m, 2H), 3.10 (s, 3H), 3.00–3.08 (m, 1H), 2.76–2.85 (m, 1H), 2.60 (ddd, J = 5.77, 8.53, 14.1 Hz, 2H), 2.22–2.36 (m, 3H), *m*/*z* calcd for C_18_H_22_N_2_O_4_S [M+H^+^] 400.1, found 401.4.

#### 6.5.13 Rac-(3*R*,3*aR*,9*bS*)-*N*-(cyclopentylmethyl)-1-methylsulfonyl-3,3*a*,4,9*b*-tetrahydro-2*H*-chromeno­[4,3-*b*]­pyrrole-3-carboxamide (**13**)

Prepared according to method C. The residue was purified by prep HPLC to afford a white solid, yield 17.4 mg (68%). ^1^H NMR (400 MHz, CDCl_3_) δ 7.67–7.77 (m, 1 H), 7.14–7.24 (m, 1 H), 6.96–7.06 (m, 1 H), 6.74–6.86 (m, 1 H), 5.72–5.88 (m, 1 H), 5.08–5.18 (m, 1 H), 4.21 (d, J = 1.51 Hz, 2 H), 3.68–3.79 (m, 1 H), 3.53–3.65 (m, 1 H), 3.21–3.33 (m, 2 H), 3.07–3.17 (m, 1 H), 3.05 (s, 3 H), 2.86–2.95 (m, 1 H), 1.99–2.15 (m, 1 H), 1.72–1.84 (m, 2 H), 1.61 (s, 5 H), 1.12–1.30 (m, 2 H), *m*/*z* calcd for C_19_H_26_N_2_O_4_S [M+H^+^] 378.2, found 378.8.

#### 6.5.14 Rac-(3*R*,3*aR*,9*bS*)-*N*-(cyclohexylmethyl)-1-methylsulfonyl-3,3*a*,4,9*b*-tetrahydro-2*H*-chromeno­[4,3-*b*]­pyrrole-3-carboxamide (**14**)

Prepared according to method C. The residue was purified by prep HPLC to afford a white solid, yield 32.0 mg (48%). ^1^H NMR (400 MHz, CDCl_3_) δ 7.68–7.79 (m, 1 H), 7.15–7.24 (m, 1 H), 6.97–7.06 (m, 1 H), 6.76–6.83 (m, 1 H), 5.65–5.89 (m, 1 H), 5.05–5.20 (m, 1 H), 4.21 (s, 2 H), 3.67–3.78 (m, 1 H), 3.52–3.65 (m, 1 H), 3.07–3.21 (m, 3 H), 3.05 (s, 3 H), 2.85–2.97 (m, 1 H), 1.66–1.84 (m, 5 H), 1.42–1.57 (m, 1 H), 1.11–1.34 (m, 3 H), 0.87–1.05 (m, 2 H), *m*/*z* calcd for C_20_H_28_N_2_O_4_S [M+H^+^] 392.2, found 379.4.

#### 6.5.15 Rac-(3*R*,3*aR*,9*bS*)-*N*-(cycloheptylmethyl)-1-methylsulfonyl-3,3*a*,4,9*b*-tetrahydro-2*H*-chromeno­[4,3-*b*]­pyrrole-3-carboxamide (**15**)

Prepared according to method C. The residue was purified by prep HPLC to afford a white solid, yield 8.0 mg (32%). ^1^H NMR (400 MHz, CDCl_3_) δ 7.69–7.76 (m, 1 H), 7.15–7.24 (m, 1 H), 6.94–7.07 (m, 1 H), 6.75–6.86 (m, 1 H), 5.71–5.84 (m, 1 H), 5.09–5.19 (m, 1 H), 4.21 (s, 2 H), 3.69–3.82 (m, 1 H), 3.51–3.64 (m, 1 H), 3.16–3.26 (m, 2 H), 3.05 (s, 4 H), 2.87–2.95 (m, 1 H), 1.35–1.79 (m, 13 H), 1.13–1.30 (m, 2 H), *m*/*z* calcd for C_21_H_30_N_2_O_4_S [M+H^+^] 406.2, found 408.0.

#### 6.5.16 Rac-(3*R*,3*aR*,9*bS*)-*N*-(cyclooctylmethyl)-1-methylsulfonyl-3,3*a*,4,9*b*-tetrahydro-2*H*-chromeno­[4,3-*b*]­pyrrole-3-carboxamide (**16**)

Prepared according to method C. The residue was purified by prep HPLC to afford a white solid, yield 8.2 mg (29%). ^1^H NMR (400 MHz, CDCl_3_) δ 7.69–7.78 (m, 1 H), 7.16–7.25 (m, 1 H), 6.98–7.07 (m, 1 H), 6.76–6.86 (m, 1 H), 5.72–5.84 (m, 1 H), 5.10–5.19 (m, 1 H), 4.21 (s, 2 H), 3.67–3.80 (m, 1 H), 3.51–3.64 (m, 1 H), 3.07–3.23 (m, 3 H), 3.05 (s, 3 H), 2.87–2.96 (m, 1 H), 1.40–1.80 (m, 14 H), 1.24–1.38 (m, 2 H), *m*/*z* calcd for C_22_H_32_N_2_O_4_S [M+H^+^] 420.2, found 421.2.

#### 6.5.17 Rac-(3*R*,3*aR*,9*bS*)-*N*-(1-adamantylmethyl)-1-methylsulfonyl-3,3*a*,4,9*b*-tetrahydro-2*H*-chromeno­[4,3-*b*]­pyrrole-3-carboxamide (**17**)

Prepared according to method C. The residue was purified by prep HPLC to afford a white solid, yield 10.5 mg (35%). ^1^H NMR (400 MHz, CDCl_3_) δ 7.67–7.78 (m, 1 H), 7.16–7.24 (m, 1 H), 6.98–7.06 (m, 1 H), 6.82 (s, 1 H), 5.68–5.85 (m, 1 H), 5.08–5.20 (m, 1 H), 4.23 (d, J = 1.51 Hz, 2 H), 3.68–3.81 (m, 1 H), 3.55–3.65 (m, 1 H), 3.10–3.21 (m, 1 H), 3.00–3.09 (m, 5 H), 2.88–2.97 (m, 1 H), 1.97–2.08 (m, 3 H), 1.60–1.81 (m, 7 H), 1.50 (br s, 6 H), *m*/*z* calcd for C_24_H_32_N_2_O_4_S [M+H^+^] 444.2, found 445.4.

#### 6.5.18 Rac-(3*R*,3*aR*,9*bS*)-*N*-cyclohexyl-1-methylsulfonyl-3,3*a*,4,9*b*-tetrahydro-2*H*-chromeno­[4,3-*b*]­pyrrole-3-carboxamide (**18**)

Prepared according to method C. The residue was purified by prep HPLC to afford a white solid, yield 14.4 mg (57%). ^1^H NMR (400 MHz, CDCl_3_) δ 7.77–7.66 (m, 1H), 7.24–7.11 (m, 1H), 7.05–6.95 (m, 1H), 6.84–6.77 (m, 1H), 5.73–5.56 (m, 1H), 5.19–5.07 (m, 1H), 4.20 (s, 2H), 3.73 (dd, J = 11.3, 8.3 Hz, 2H), 3.56 (dd, J = 11.3, 9.0 Hz, 1H), 3.05 (s, 4H), 2.93–2.82 (m, 1H), 2.01–1.87 (m, 2H), 1.83–1.54 (m, 4H), 1.49–1.28 (m, 2H), 1.27–1.05 (m, 3H), *m*/*z* calcd for C_19_H_26_N_2_O_4_S [M+H^+^] 378.16, found 379.0.

#### 6.5.19 Rac-(3*R*,3*aR*,9*bS*)-*N*-(2-cyclohexylethyl)-1-methylsulfonyl-3,3*a*,4,9*b*-tetrahydro-2*H*-chromeno­[4,3-*b*]­pyrrole-3-carboxamide (**19**)

Prepared according to method B. The residue was purified by prep HPLC to afford a white solid, yield 17.1 mg (63%). ^1^H NMR (400 MHz, DMSO-*d*) δ 7.65–7.76 (m, 1H), 7.14–7.22 (m, 1H), 6.95–7.05 (m, 1H), 6.74–6.83 (m, 1H), 5.63–5.77 (m, 1 H), 5.07–5.18 (m, 1H), 4.18 (s, 2H), 3.66–3.79 (m, 1H), 3.49–3.62 (m, 1H), 3.25–3.40 (m, 2H), 3.04 (s, 4H), 2.84–2.93 (m, 1H), 1.57–1.77 (m, 6H), 1.37–1.48 (m, 2H), 1.07–1.34 (m, 4H), 0.84–1.02 (m, 2H), *m*/*z* calcd for C_21_H_30_N_2_O_4_S [M+H^+^] 406.2, found 406.8.

#### 6.5.20 Rac-(3*R*,3*aR*,9*bS*)-*N*-[(4,4-difluorocyclohexyl)­methyl]-1-methylsulfonyl-3,3*a*,4,9*b*-tetrahydro-2*H*-chromeno­[4,3-*b*]­pyrrole-3-carboxamide (**20**)

Prepared according to method B. The residue was purified by prep HPLC to afford a white solid, yield 15.0 mg (52%). ^1^H NMR (400 MHz, DMSO-*d*) δ 8.22–8.34 (m, 1H), 7.50–7.61 (m, 1H), 7.11–7.25 (m, 1H), 6.91–7.05 (m, 1H), 6.72–6.86 (m, 1H), 5.07–5.19 (m, 1H), 4.00–4.21 (m, 2H), 3.35–3.48 (m, 2H), 3.10 (s, 3H), 2.92–3.08 (m, 2H), 2.75–2.86 (m, 1H), 1.91–2.08 (m, 2H), 1.66–1.89 (m, 4H), 1.47–1.63 (m, 1H), 1.07–1.24 (m, 2H), *m*/*z* calcd for C_20_H_26_F_2_N_2_O_4_S [M+H^+^] 428.2, found 429.0.

#### 6.5.21 Rac-(3*R*,3*aR*,9*bS*)-*N*-[(4-methylcyclohexyl)­methyl]-1-methylsulfonyl-3,3*a*,4,9*b*-tetrahydro-2*H*-chromeno­[4,3-*b*]­pyrrole-3-carboxamide (**21**)

Prepared according to method C. The residue was purified by prep HPLC to afford a white solid, yield 14.9 mg (55%). ^1^H NMR (400 MHz, CDCl_3_) δ 7.67–7.79 (m, 1 H), 7.13–7.25 (m, 5 H), 6.97–7.06 (m, 1 H), 6.77–6.84 (m, 1 H), 5.77–5.90 (m, 1 H), 5.09–5.21 (m, 1 H), 4.07–4.27 (m, 2 H), 3.66–3.78 (m, 1 H), 3.49–3.60 (m, 1 H), 3.45 (s, 2 H), 3.07–3.18 (m, 3 H), 3.05 (s, 3 H), 2.83–2.93 (m, 1 H), 2.73 (br s, 3 H), *m*/*z* calcd for C_21_H_30_N_2_O_4_S [M+H^+^] 406.2, found 407.4

#### 6.5.22 Rac-(3*R*,3*aR*,9*bS*)-*N*-[(1-methyl-4-piperidyl)­methyl]-1-methylsulfonyl-3,3*a*,4,9*b*-tetrahydro-2*H*-chromeno­[4,3-*b*]­pyrrole-3-carboxamide (**22**)

Prepared according to method C. The residue was purified by prep HPLC to afford a white solid, yield 12.6 mg (46%). ^1^H NMR (400 MHz, CDCl_3_) δ 7.65–7.77 (m, 1 H), 7.14–7.23 (m, 1 H), 6.96–7.06 (m, 1 H), 6.73–6.85 (m, 1 H), 5.91–6.07 (m, 1 H), 5.11–5.19 (m, 1 H), 4.13–4.25 (m, 2 H), 3.65–3.77 (m, 1 H), 3.52–3.63 (m, 1 H), 3.08–3.31 (m, 3 H), 3.04 (s, 3 H), 2.84–2.96 (m, 3 H), 2.31 (s, 3 H), 1.93–2.04 (m, 2 H), 1.65–1.75 (m, 2 H), 1.47–1.60 (m, 1 H), 1.29–1.45 (m, 2 H), *m*/*z* calcd for C_20_H_29_N_3_O_4_S [M+H^+^] 407.2, found 408.0.

#### 6.5.23 Rac-(3*R*,3*aR*,9*bS*)-1-methylsulfonyl-*N*-(tetrahydropyran-4-ylmethyl)-3,3*a*,4,9*b*-tetrahydro-2*H*-chromeno­[4,3-*b*]­pyrrole-3-carboxamide (**23**)

Prepared according to method C. The residue was purified by prep HPLC to afford a white solid, yield 19.8 mg (75%). ^1^H NMR (400 MHz, CDCl_3_) δ 7.64–7.78 (m, 1 H), 7.15–7.24 (m, 1 H), 6.98–7.06 (m, 1 H), 6.76–6.84 (m, 1 H), 5.77–5.92 (m, 1 H), 5.15 (d, J = 7.78 Hz, 1 H), 4.20 (dd, J = 4.27, 1.76 Hz, 2 H), 3.93–4.07 (m, 2 H), 3.66–3.79 (m, 1 H), 3.53–3.64 (m, 1 H), 3.39 (d, J = 1.25 Hz, 2 H), 3.24 (s, 2 H), 3.08–3.18 (m, 1 H), 3.05 (s, 3 H), 2.86–2.96 (m, 1 H), 1.70–1.87 (m, 1 H), 1.54–1.68 (m, 4 H), 1.36 (d, J = 4.52 Hz, 2 H), *m*/*z* calcd for C_19_H_26_N_2_O_5_S [M+H^+^] 394.2, found 394.8.

#### 6.5.24 Rac-(3*R*,3*aR*,9*bS*)-1-methylsulfonyl-*N*-[[4-(trifluoromethyl)­cyclohexyl]­methyl]-3,3*a*,4,9*b*-tetrahydro-2*H*-chromeno­[4,3-*b*]­pyrrole-3-carboxamide (**24**)

Prepared according to method C. The residue was purified by prep HPLC to afford a white solid, yield 25.6 mg (83%). ^1^H NMR (400 MHz, CDCl_3_) δ 7.66–7.79 (m, 1 H), 7.13–7.24 (m, 1 H), 6.96–7.08 (m, 1 H), 6.73–6.86 (m, 1 H), 5.73–5.92 (m, 1 H), 5.10–5.23 (m, 1 H), 4.13–4.28 (m, 2 H), 3.67–3.78 (m, 1 H), 3.54–3.63 (m, 1 H), 3.31–3.39 (m, 1 H), 3.18–3.24 (m, 1 H), 3.08–3.17 (m, 1 H), 3.05 (s, 3 H), 2.85–2.96 (m, 1 H), 2.07–2.21 (m, 1 H), 1.95–2.06 (m, 1 H), 1.78–1.93 (m, 1 H), 1.65–1.78 (m, 2 H),) 1.60 (d, J = 4.77 Hz, 4 H), 1.24–1.41 (m, 1 H), 0.94–1.09 (m, 1 H), *m*/*z* calcd for C_21_H_27_F_3_N_2_O_4_S [M+H^+^] 460.2, found 461.2.

#### 6.5.25 Rac-(3*R*,3*aR*,9*bS*)-*N*-[(4,4-dimethylcyclohexyl)­methyl]-1-methylsulfonyl-3,3*a*,4,9*b*-tetrahydro-2*H*-chromeno­[4,3-*b*]­pyrrole-3-carboxamide (**25**)

Prepared according to method C. The residue was purified by prep HPLC to afford a white solid, yield 10.6 mg (38%). ^1^H NMR (400 MHz, CDCl_3_) δ 7.67–7.79 (m, 1 H), 7.14–7.24 (m, 1 H), 6.96–7.09 (m, 1 H), 6.73–6.86 (m, 1 H), 5.71–5.89 (m, 1 H), 5.07–5.22 (m, 1 H), 4.14–4.28 (m, 2 H), 3.67–3.80 (m, 1 H), 3.49–3.64 (m, 1 H), 3.17–3.25 (m, 2 H), 3.07–3.16 (m, 1 H), 3.05 (s, 3 H), 2.86–2.96 (m, 1 H), 1.49–1.58 (m, 2 H), 1.34–1.47 (m, 3 H), 1.09–1.25 (m, 4 H), 0.91 (d, J = 14.56 Hz, 6 H), *m*/*z* calcd for C_22_H_32_N_2_O_4_S [M+H^+^] 420.2, found 421.2.

#### 6.5.26 Rac-(3*R*,3*aR*,9*bS*)-1-methylsulfonyl-*N*-[(4-isopropylcyclohexyl)­methyl]-13,3*a*,4,9*b*-tetrahydro-2*H*-chromeno­[4,3-*b*]­pyrrole-3-carboxamide (**26**)

Prepared according to method C. The residue was purified by prep HPLC to afford a white solid, yield 15.4 mg (53%). ^1^H NMR (400 MHz, CDCl_3_) δ 7.67–7.79 (m, 1H), 7.14–7.25 (m, 1H), 6.96–7.08 (m, 1H), 6.72–6.87 (m, 1H), 5.69–5.88 (m, 1H), 5.07–5.20 (m, 1H), 4.13–4.26 (m, 2H), 3.66–3.79 (m, 1H), 3.52–3.64 (m, 1H), 3.25–3.34 (m, 1H), 3.14–3.21 (m, 1H), 3.07–3.14 (m, 1H), 3.05 (s, 3H), 2.87–2.95 (m, 1H), 1.68–1.85 (m, 3H), 1.31–1.58 (m, 5H), 0.92–1.19 (m, 3H), 0.81–0.91 (m, 6H), *m*/*z* calcd for C_23_H_34_N_2_O_4_S [M+H^+^] 434.2, found 435.2.

#### 6.5.27 Rac-(3*R*,3*aR*,9*bS*)-1-methylsulfonyl-*N*-[(3,3,5,5-tetramethylcyclohexyl)­methyl]-3,3*a*,4,9*b*-tetrahydro-2*H*-chromeno­[4,3-*b*]­pyrrole-3-carboxamide (**27**)

Prepared according to method C. The residue was purified by prep HPLC to afford a white solid, yield 20.0 mg (65%). ^1^H NMR (400 MHz, DMSO-*d*_6_) δ 8.16–8.29 (m, 1H), 7.49–7.62 (m, 1H), 7.11–7.25 (m, 1H), 6.92–7.03 (m, 1H), 6.74–6.86 (m, 1H), 5.09–5.19 (m, 1H), 4.01–4.22 (m, 2H), 3.34–3.50 (m, 2H), 3.10 (s, 3H), 2.96–3.06 (m, 1H), 2.77–2.93 (m, 2H), 1.66–1.83 (m, 1H), 1.38 (d, J = 10.54 Hz, 2H), 1.21 (d, J = 13.55 Hz, 1H), 1.00 (d, J = 13.80 Hz, 1H), 0.95 (s, 6H), 0.87 (s, 6H), 0.67 (t, J = 12.67 Hz, 2H), *m*/*z* calcd for C_24_H_36_N_2_O_4_S [M+H^+^] 448.2, found 449.2.

#### 6.5.28 Rac-(3*R*,3*aR*,9*bS*)-*N*-[[4-(aminomethyl)­cyclohexyl]­methyl]-1-methylsulfonyl-3,3*a*,4,9*b*-tetrahydro-2*H*-chromeno­[4,3-*b*]­pyrrole-3-carboxamide (**28**)

Prepared according to method C. The residue was purified by prep HPLC to afford a white solid, yield 8.6 mg (30%). ^1^H NMR (400 MHz, CDCl_3_) δ 7.67–7.77 (m, 1 H), 7.14–7.24 (m, 1 H), 6.97–7.07 (m, 1 H), 6.75–6.85 (m, 1 H), 5.90–6.03 (m, 1 H), 5.15 (d, J = 8.03 Hz, 1 H), 4.20 (s, 2 H), 3.68–3.77 (m, 1 H), 3.53–3.63 (m, 1 H), 3.24–3.34 (m, 1 H), 3.08–3.23 (m, 2 H), 3.00–3.07 (m, 3 H), 2.86–2.95 (m, 1 H), 2.69–2.76 (m, 1 H), 2.59–2.65 (m, 1 H), 2.04–2.48 (m, 4 H), 1.68–1.91 (m, 2 H), 1.30–1.66 (m, 7 H), 0.89–1.08 (m, 1 H), *m*/*z* calcd for C_21_H_31_N_3_O_4_S [M+H^+^] 421.2, found 422.0.

#### 6.5.29 Rac-(3*R*,3*aR*,9*bS*)-1-methylsulfonyl-*N*-(norbornan-1-ylmethyl)-3,3*a*,4,9*b*-tetrahydro-2*H*-chromeno­[4,3-*b*]­pyrrole-3-carboxamide (**29**)

Prepared according to method C. The residue was purified by prep HPLC to afford a white solid, yield 18.0 mg (65%). ^1^H NMR (400 MHz, DMSO-*d*_6_) δ 8.10–8.23 (m, 1H), 7.51–7.61 (m, 1H), 7.11–7.23 (m, 1H), 6.91–7.03 (m, 1H), 6.75–6.86 (m, 1H), 5.09–5.19 (m, 1H), 4.01–4.23 (m, 2H), 3.34–3.46 (m, 2H), 3.12–3.28 (m, 2H), 3.10 (s, 2H), 2.82 (d, J = 2.26 Hz, 1H), 2.14 (br s, 1H), 1.49–1.61 (m, 2H), 1.32–1.42 (m, 2H), 1.21–1.30 (m, 2H), 1.08–1.20 (m, 4H), *m*/*z* calcd for C_21_H_28_N_2_O_4_S [M+H^+^] 404.5, found 405.0.

#### 6.5.30 Rac-(3*R*,3*aR*,9*bS*)-1-methylsulfonyl-*N*-[(4-phenylcyclohexyl)­mehyl]-3,3*a*,4,9*b*-tetrahydro-2*H*-chromeno­[4,3-*b*]­pyrrole-3-carboxamide (**30**)

Prepared according to method C. The residue was purified by prep HPLC to afford a white solid, yield 6.4 mg (20%). ^1^H NMR (400 MHz, CDCl_3_) δ 7.68–7.79 (m, 1 H), 7.29–7.35 (m, 2 H), 7.17–7.27 (m, 4 H), 6.96–7.08 (m, 1 H), 6.74–6.87 (m, 1 H), 5.70–5.89 (m, 1 H), 5.08–5.21 (m, 1 H), 4.14–4.29 (m, 2 H), 3.69–3.81 (m, 1 H), 3.54–3.66 (m, 1 H), 3.39–3.50 (m, 1 H), 3.20–3.29 (m, 1 H), 3.09–3.19 (m, 1 H), 3.06 (d, J = 3.76 Hz, 3 H), 2.87–2.98 (m, 1 H), 2.59–2.70 (m, 1 H), 2.42–2.57 (m, 1 H), 1.83–2.02 (m, 3 H), 1.64–1.80 (m, 3 H), 1.56–1.63 (m, 2 H), 1.41–1.56 (m, 1 H), 1.06–1.23 (m, 1 H), *m*/*z* calcd for C_26_H_32_N_2_O_4_S [M+H^+^] 468.2, found 469.0.

#### 6.5.31 Rac-(3*R*,3*aR*,9*bS*)-1-methylsulfonyl-*N*-tetralin-6-yl-3,3*a*,4,9*b*-tetrahydro-2*H*-chromeno­[4,3-*b*]­pyrrole-3-carboxamide (**31**)

Prepared according to method C. The residue was purified by prep HPLC to afford a white solid, yield 13.5 mg (47%). ^1^H NMR (400 MHz, CDCl_3_) δ 7.69–7.78 (m, 1 H), 7.42–7.52 (m, 1 H), 7.14–7.25 (m, 2 H), 6.97–7.09 (m, 2 H), 6.74–6.89 (m, 1 H), 5.10–5.24 (m, 1 H), 4.15–4.33 (m, 2 H), 3.74–3.87 (m, 1 H), 3.56–3.69 (m, 1 H), 3.19–3.34 (m, 1 H), 3.06 (s, 3 H), 2.93–3.02 (m, 1 H), 2.68–2.81 (m, 4 H), 1.80 (br s, 4 H), *m*/*z* calcd for C_24_H_28_N_2_O_4_S [M+H^+^] 440.2, found 441.0.

#### 6.5.32 Rac-(3*R*,3*aR*,9*bS*)-1-methylsulfonyl-*N*-(tetralin-1-ylmethyl)-3,3*a*,4,9*b*-tetrahydro-2*H*-chromeno­[4,3-*b*]­pyrrole-3-carboxamide (**32**)

Prepared according to method C. The residue was purified by prep HPLC to afford a white solid, yield 18.8 mg (63%). ^1^H NMR (400 MHz, CDCl_3_) δ 7.66–7.78 (m, 1 H), 7.08–7.26 (m, 5 H), 6.97–7.06 (m, 1 H), 6.74–6.83 (m, 1 H), 5.71–5.90 (m, 1 H), 5.08–5.18 (m, 1 H), 4.00–4.23 (m, 2 H), 3.67–3.77 (m, 1 H), 3.48–3.67 (m, 3 H), 2.99–3.17 (m, 5 H), 2.83–2.96 (m, 1 H), 2.80 (br s, 2 H), 1.84–1.99 (m, 2 H), 1.66–1.84 (m, 2 H), *m*/*z* calcd for C_24_H_28_N_2_O_4_S [M+H^+^] 440.2, found 441.2.

#### 6.5.33 Rac-(3*R*,3*aR*,9*bS*)-1-methylsulfonyl-*N*-tetralin-6-yl-3,3*a*,4,9*b*-tetrahydro-2*H*-chromeno­[4,3-*b*]­pyrrole-3-carboxamide (**33**)

Prepared according to method C. The residue was purified by prep HPLC to afford a white solid, yield 13.5 mg (47%). ^1^H NMR (400 MHz, CDCl_3_) δ 7.69–7.78 (m, 1H), 7.42–7.52 (m, 1H), 7.14–7.25 (m, 2H), 6.97–7.09 (m, 2 H), 6.74–6.89 (m, 1H), 5.10–5.24 (m, 1H), 4.15–4.33 (m, 2 H), 3.74–3.87 (m, 1H), 3.56–3.69 (m, 1H), 3.19–3.34 (m, 1 H), 3.06 (s, 3 H), 2.93–3.02 (m, 1H), 2.68–2.81 (m, 4H), 1.75–1.90 (br s, 4H), *m*/*z* calcd for C_23_H_26_N_2_O_4_S [M+H^+^] 426.2, found 427.2.

#### 6.5.34 Rac-(3*R*,3*aR*,9*bS*)-*N*-(indan-2-ylmethyl)-1-methylsulfonyl-3,3*a*,4,9*b*-tetrahydro-2*H*-chromeno­[4,3-*b*]­pyrrole-3-carboxamide (**34**)

Prepared according to method C. The residue was purified by prep HPLC to afford a white solid, yield 20.0 mg (70%). ^1^H NMR (400 MHz, CDCl_3_) δ 7.67–7.79 (m, 1 H), 7.13–7.25 (m, 5 H), 6.97–7.06 (m, 1 H), 6.77–6.84 (m, 1 H), 5.77–5.90 (m, 1 H), 5.09–5.21 (m, 1 H), 4.07–4.27 (m, 2 H), 3.66–3.78 (m, 1 H), 3.49–3.60 (m, 1 H), 3.45 (s, 2 H), 3.07–3.18 (m, 3 H), 3.05 (s, 3 H), 2.83–2.93 (m, 1 H), 2.73 (br s, 3 H), *m*/*z* calcd for C_23_H_26_O_4_S [M+H^+^] 426.2, found 427.2.

#### 6.5.35 Rac-(3*R*,3*aR*,9*bS*)-*N*-benzyl-1-methylsulfonyl-3,3*a*,4,9*b*-tetrahydro-2*H*-chromeno­[4,3-*b*]­pyrrole-3-carboxamide (**35**)

Prepared according to method C. The residue was purified by prep HPLC to afford a white solid, yield 15.9 mg (61%). ^1^H NMR (400 MHz, CDCl_3_) δ 7.64–7.79 (m, 1 H), 7.24–7.43 (m, 5 H), 7.11–7.23 (m, 1 H), 6.95–7.06 (m, 1 H), 6.79 (d, J = 0.50 Hz, 1 H), 6.01–6.23 (m, 1 H), 5.07–5.21 (m, 1 H), 4.42–4.59 (m, 2 H), 4.19 (d, J = 1.76 Hz, 2 H), 3.69–3.83 (m, 1 H), 3.51–3.65 (m, 1 H), 3.09–3.23 (m, 1 H), 3.05 (s, 3 H), 2.88–2.98 (m, 1 H), *m*/*z* calcd for C_20_H_22_N_2_O_4_S [M+H^+^] 386.2, found 387.4.

#### 6.5.36 Rac-(3*R*,3*aR*,9*bS*)-*N*-[(1-methylcyclohexyl)­methyl]-1-methylsulfonyl-3,3*a*,4,9*b*-tetrahydro-2*H*-chromeno­[4,3-*b*]­pyrrole-3-carboxamide (**36**)

Prepared according to method C. The residue was purified by prep HPLC to afford a white solid, yield 10.0 mg (37%). ^1^H NMR (400 MHz, DMSO-*d*_6_) δ 8.02–8.15 (m, 1H), 7.50–7.63 (m, 1H), 7.12–7.24 (m, 1H), 6.91–7.03 (m, 1H), 6.80 (d, J = 8.03 Hz, 1H), 5.13 (d, J = 8.03 Hz, 1H), 4.00–4.25 (m, 2H), 3.37–3.44 (m, 2H), 3.13–3.23 (m, 1H), 3.10 (s, 3H), 2.90–2.98 (m, 1H), 2.83 (dd, J = 8.16, 10.42 Hz, 1H), 1.09–1.53 (m, 9H), 0.81 (s, 3H), *m*/*z* calcd for C_21_H_30_N_2_O_4_S [M+H^+^] 406.2, found 407.0.

#### 6.5.37 Rac-(3*R*,3*aR*,9*bS*)-*N-*[[1-(dimethylamino)­cyclohexyl]­methyl]-1-methylsulfonyl-3,3*a*,4,9*b*-tetrahydro-2*H*-chromeno­[4,3-*b*]­pyrrole-3-carboxamide (**37**)

Prepared according to method C. The residue was purified by prep HPLC to afford a white solid, yield 10.0 mg (37%). ^1^H NMR (400 MHz, DMSO-*d*_6_): δ 7.89–8.01 (m, 1H), 7.51–7.63 (m, 1H), 7.12–7.25 (m, 1H), 6.93–7.03 (m, 1H), 6.74–6.85 (m, 1H), 5.06–5.19 (m, 1H), 3.98–4.23 (m, 2H), 3.36–3.42 (m, 2H), 3.17–3.27 (m, 2H), 3.12–3.16 (m, 1H), 3.10 (s, 2H), 2.79–2.88 (m, 1H), 2.22 (s, 6H), 1.13–1.66 (m, 9H), *m*/*z* calcd for C_22_H_33_N_3_O_4_S [M+H^+^] 435.2, found 436.4.

#### 6.5.38 Rac-(3*R*,3*aR*,9*bS*)-*N*-[(1-hydroxycyclohexyl)­methyl]-1-methylsulfonyl-3,3*a*,4,9*b*-tetrahydro-2*H*-chromeno­[4,3-*b*]­pyrrole-3-carboxamide (**38**)

Prepared according to method C. The residue was purified by prep HPLC to afford a white solid, yield 10.0 mg (36%). ^1^H NMR (400 MHz, DMSO-*d*_6_): δ 7.98–8.12 (m, 1H), 7.50–7.63 (m, 1H), 7.13–7.23 (m, 1H), 6.92–7.00 (m, 1H), 6.75–6.86 (m, 1H), 5.06–5.17 (m, 1H), 4.20 (s, 1H), 4.06–4.18 (m, 2H), 3.40 (s, 2H), 3.14–3.25 (m, 2H), 3.09 (s, 3H), 2.96–3.04 (m, 1H), 2.78–2.88 (m, 1H), 1.54 (d, J = 8.78 Hz, 2H), 1.39 (d, J = 9.54 Hz, 4H), 1.11–1.33 (m, 3H), *m*/*z* calcd for C_20_H_28_N_2_O_5_S [M+H^+^] 408.2, found 409.0.

#### 6.5.39 Rac-(3*R*,3*aR*,9*bS*)-1-methylsulfonyl-*N*-[(1-morpholinocyclohexyl)­methyl]-3,3*a*,4,9*b*-tetrahydro-2*H*-chromeno­[4,3-*b*]­pyrrole-3-carboxamide (**39**)

Prepared according to method C. The residue was purified by prep HPLC to afford a white solid, yield 22.0 mg (69%). ^1^H NMR (400 MHz, DMSO-*d*_6_): δ 7.90–8.04 (m, 1H), 7.50–7.61 (m, 1H), 7.10–7.22 (m, 1H), 6.91–7.03 (m, 1H), 6.73–6.86 (m, 1H), 5.09–5.21 (m, 1H), 4.05–4.26 (m, 2H), 3.48–3.59 (m, 4H), 3.37–3.44 (m, 2H), 3.14–3.24 (m, 2H), 3.10 (s, 3H), 2.79–2.88 (m, 1H), 2.55 (br s, 3H), 1.58–1.69 (m, 2H), 1.43–1.56 (m, 2H), 1.27–1.38 (m, 2H), 1.151.25 (m, 2H), *m*/*z* calcd for C_24_H_35_N_3_O_5_S [M+H^+^] 477.2, found 478.4.

#### 6.5.40 Rac-(3*R*,3*aR*,9*bS*)-1-methylsulfonyl-*N*-[(1-phenylcyclohexyl)­methyl]-3,3*a*,4,9*b*-tetrahydro-2*H*-chromeno­[4,3-*b*]­pyrrole-3-carboxamide (**40**)

Prepared according to method C. The residue was purified by prep HPLC to afford a white solid, yield 15.0 mg (48%). ^1^H NMR (400 MHz, DMSO-*d*_6_): δ 7.80–7.92 (m, 1H), 7.47–7.57 (m, 1H), 7.25–7.41 (m, 4H), 7.09–7.24 (m, 2H), 6.90–7.01 (m, 1H), 6.72–6.84 (m, 1H), 5.01–5.08 (m, 1H), 4.01–4.11 (m, 1H), 3.81–3.93 (m, 1H), 3.12–3.30 (m, 4H), 3.01 (s, 4H), 2.58–2.70 (m, 1H), 2.07 (m, 2H), 1.47–1.62 (m, 4H), 1.13–1.47 (m, 4H), *m*/*z* calcd for C_26_H_32_N_2_O_4_S [M+H^+^] 468.6, found 469.0.

#### 6.5.41 Rac-(3*R*,3*aR*,9*bS*)-*N*-(cyclohexylmethyl)-*N*-methyl-1-methylsulfonyl-3,3*a*,4,9*b*-tetrahydro-2*H*-chromeno­[4,3-*b*]­pyrrole-3-carboxamide (**41**)

Prepared according to method C. The residue was purified by prep HPLC to afford a white solid, yield 13.7 mg (50%). ^1^H NMR (400 MHz, CDCl_3_) δ 7.70–7.83 (m, 1 H), 7.21 (s, 1 H), 7.03 (s, 1 H), 6.76–6.88 (m, 1 H), 5.13 (d, J = 8.03 Hz, 1 H), 4.02–4.28 (m, 2 H), 3.49–3.76 (m, 3 H), 2.85–3.35 (m, 9 H), 1.50–1.85 (m, 7 H), 1.07–1.35 (m, 3 H), 0.73–1.06 (m, 2 H), *m*/*z* calcd for C_21_H_30_N_2_O_4_S [M+H^+^] 406.2, found 407.4.

#### 6.5.42 Rac-(3*R*,3*aR*,9*bS*)-1-acetyl-*N*-(cyclohexylmethyl)-3,3*a*,4,9*b*-tetrahydro-2*H*-chromeno­[4,3-*b*]­pyrrole-3-carboxamide (**42**)

Prepared according to method C. The residue was purified by prep HPLC to afford a white solid, yield 31.9 mg (78%). ^1^H NMR (400 MHz, CDCl_3_) δ 7.67–7.79 (m, 1 H), 7.11–7.21 (m, 1 H), 6.89–6.98 (m, 1 H), 6.73–6.83 (m, 1 H), 5.75–5.86 (m, 1 H), 5.66–5.73 (m, 1 H), 4.19 (d, J = 1.51 Hz, 2 H), 3.81–3.93 (m, 1 H), 3.59–3.74 (m, 1 H), 3.08–3.29 (m, 3 H), 2.74–2.91 (m, 1 H), 2.12 (s, 3 H), 1.66–1.85 (m, 6 H), 1.38–1.59 (m, 1 H), 1.11–1.34 (m, 3 H), 0.85–1.06 (m, 2 H), *m*/*z* calcd for C_21_H_28_N_2_O_3_ [M+H^+^] 356.2, found 357.2.

#### 6.5.43 Rac-(3*R*,3*aR*,9*bS*)-1-methylsulfonyl-*N*-[[Rac-(2*R*)-tetrahydrofuran-2-yl]­methyl]-3,3*a*,4,9*b*-tetrahydro-2*H*-chromeno­[4,3-*b*]­pyrrole-3-carboxamide (**43**)

Prepared according to method C. The residue was purified by prep HPLC to afford a white solid, yield 13.3 mg (52%). ^1^H NMR (400 MHz, CDCl_3_) δ 7.67–7.78 (m, 1 H), 7.14–7.24 (m, 1 H), 6.95–7.08 (m, 1 H), 6.74–6.85 (m, 1 H), 6.02–6.22 (m, 1 H), 5.04–5.21 (m, 1 H), 4.14–4.28 (m, 2 H), 3.93–4.04 (m, 1 H), 3.83–3.92 (m, 1 H), 3.70–3.82 (m, 2 H), 3.60 (d, J = 9.03 Hz, 2 H), 3.16 (s, 2 H), 3.05 (s, 3 H), 2.85–2.96 (m, 1 H), 1.83–2.10 (m, 3 H), 1.46–1.61 (m, 1 H), *m*/*z* calcd for C_18_H_24_N_2_O_5_S [M+H^+^] 380.1, found 382.0.

#### 6.5.44 Rac-(3*R*,3*aR*,9*bS*)-1-acetyl-*N*-[[Rac-(2*R*)-tetrahydrofuran-2-yl]­methyl]-3,3*a*,4,9*b*-tetrahydro-2*H*-chromeno­[4,3-*b*]­pyrrole-3-carboxamide (**44**)

Prepared according to method C. The residue was purified by prep HPLC to afford a white solid, yield 16.3 mg (62%). ^1^H NMR (400 MHz, CDCl_3_) δ 7.64–7.76 (m, 1 H), 7.10–7.20 (m, 1 H), 6.89–6.97 (m, 1 H), 6.75–6.84 (m, 1 H), 6.09–6.26 (m, 1 H), 5.60–5.78 (m, 1 H), 4.19 (s, 2 H), 3.94–4.05 (m, 1 H), 3.82–3.93 (m, 2 H), 3.78 (d, J = 7.03 Hz, 1 H), 3.62–3.73 (m, 2 H), 3.20 (s, 2 H), 2.71–2.87 (m, 1 H), 2.12 (s, 3 H), 1.84–2.08 (m, 3 H), 1.43–1.64 (m, 1 H), *m*/*z* calcd for C_19_H_24_N_2_O_4_ [M+H^+^] 344.2, found 345.4.

#### 6.5.45 Rac-(3*R*,3*aR*,9*bS*)-1-methylsulfonyl-*N*-[[Rac-(2*R*)-tetrahydropyran-2-yl]­methyl]-3,3*a*,4,9*b*-tetrahydro-2*H*-chromeno­[4,3-*b*]­pyrrole-3-carboxamide (**45**)

Prepared according to method C. The residue was purified by prep HPLC to afford a white solid, yield 15.2 mg (57%). ^1^H NMR (400 MHz, CDCl_3_) δ 7.66–7.77 (m, 1 H), 7.14–7.24 (m, 1 H), 6.97–7.07 (m, 1 H), 6.72–6.86 (m, 1 H), 6.04–6.26 (m, 1 H), 5.08–5.19 (m, 1 H), 4.21 (d, J = 1.51 Hz, 2 H), 3.92–4.05 (m, 1 H), 3.68–3.80 (m, 1 H), 3.52–3.66 (m, 2 H), 3.32–3.50 (m, 2 H), 3.08–3.23 (m, 2 H), 3.05 (d, J = 2.01 Hz, 3 H), 2.86–2.97 (m, 1 H), 1.81–1.96 (m, 1 H), 1.41–1.64 (m, 4 H), 1.18–1.39 (m, 1 H), *m*/*z* calcd for C_19_H_26_N_2_O_5_S [M+H^+^] 394.2, found 395.0.

#### 6.5.46 Rac-(3*R*,3*aR*,9*bS*)-1-acetyl-*N*-[[Rac-(2*R*)-tetrahydropyran-2-yl]­methyl]-3,3*a*,4,9*b*-tetrahydro-2*H*-chromeno­[4,3-*b*]­pyrrole-3-carboxamide (**46**)

Prepared according to method C. The residue was purified by prep HPLC to afford a white solid, yield 18.3 mg (67%). ^1^H NMR (400 MHz, CDCl_3_) δ 7.66–7.75 (m, 1 H), 7.16 (s, 1 H), 6.93 (s, 1 H), 6.80 (d, J = 8.28 Hz, 1 H), 6.17 (br s, 1 H), 5.70 (d, J = 7.78 Hz, 1 H), 4.20 (d, J = 6.78 Hz, 2 H), 3.94–4.08 (m, 1 H), 3.82–3.92 (m, 1 H), 3.56–3.72 (m, 2 H), 3.34–3.51 (m, 2 H), 3.01–3.23 (m, 2 H), 2.75–2.86 (m, 1 H), 2.13 (d, J = 1.00 Hz, 3 H), 1.82–1.93 (m, 1 H), 1.44–1.66 (m, 4 H), 1.19–1.41 (m, 1 H), *m*/*z* calcd for C_20_H_26_N_2_O_4_ [M+H^+^] 358.2, found 359.4.

#### 6.5.47 Rac-(3*R*,3*aR*,9*bS*)-1-methylsulfonyl-*N*-[[Rac-(2*S*)-1,4-dioxan-2-yl]­methyl]-3,3*a*,4,9*b*-tetrahydro-2*H*-chromeno­[4,3-*b*]­pyrrole-3-carboxamide (**47**)

Prepared according to method C. The residue was purified by prep HPLC to afford a white solid, yield 16. mg (61%). ^1^H NMR (400 MHz, CDCl_3_) δ 7.65–7.79 (m, 1 H), 7.14–7.24 (m, 1 H), 6.93–7.08 (m, 1 H), 6.75–6.87 (m, 1 H), 6.05–6.20 (m, 1 H), 5.10–5.22 (m, 1 H), 4.21 (s, 2 H), 3.65–3.86 (m, 6 H), 3.47–3.65 (m, 3 H), 3.28–3.38 (m, 1 H), 3.11–3.25 (m, 2 H), 3.04 (d, J = 0.75 Hz, 3 H), 2.85–2.95 (m, 1 H), *m*/*z* calcd for C_18_H_24_N_2_O_6_S [M+H^+^] 394.1, found 397.2.

#### 6.5.48 Rac-(3*R*,3*aR*,9*bS*)-1-acetyl-*N*-[[Rac-(2*S*)-1,4-dioxan-2-yl]­methyl]-3,3*a*,4,9*b*-tetrahydro-2*H*-chromeno­[4,3-*b*]­pyrrole-3-carboxamide (**48**)

Prepared according to method C. The residue was purified by prep HPLC to afford a white solid, yield 16.7 mg (61%). ^1^H NMR (400 MHz, CDCl_3_) δ 7.65–7.75 (m, 1 H), 7.12–7.20 (m, 1 H), 6.88–6.96 (m, 1 H), 6.77–6.83 (m, 1 H), 6.05–6.21 (m, 1 H), 5.60–5.77 (m, 1 H), 4.13–4.27 (m, 2 H), 3.47–3.92 (m, 10 H), 3.27–3.39 (m, 1 H), 3.13–3.26 (m, 2 H), 2.69–2.87 (m, 1 H), 2.13 (s, 3 H), *m*/*z* calcd for C_19_H_24_N_2_O_5_ [M+H^+^] 360.2, found 361.2.

#### 6.5.49 Rac-(3*R*,3*aR*,9*bS*)-1-methylsulfonyl-*N*-[[Rac-(3*S*)-2,3-dihydro-1,4-benzodioxin-3-yl]­methyl]-3,3*a*,4,9*b*-tetrahydro-2*H*-chromeno­[4,3-*b*]­pyrrole-3-carboxamide (**49**)

Prepared according to method C. The residue was purified by prep HPLC to afford a white solid, yield 17.0 mg (57%). ^1^H NMR (400 MHz, CDCl_3_) δ 7.66–7.80 (m, 1H), 7.16–7.24 (m, 1H), 6.98–7.05 (m, 2H), 6.90 (s, 3H), 6.77–6.84 (m, 1H), 6.17–6.29 (m, 1 H), 5.16 (d, J = 7.78 Hz, 1 H), 4.30 (d, J = 12.05 Hz, 3H), 4.19 (d, J = 1.76 Hz, 1 H), 3.90–4.04 (m, 1H), 3.76 (s, 2H), 3.44–3.63 (m, 2H), 3.13–3.25 (m, 1H), 3.04 (s, 3H), 2.82–2.96 (m, 1 H), *m*/*z* calcd for C_22_H_24_N_2_O_6_S [M+H^+^] 444.1, found 445.0.

#### 6.5.50 Rac-(3*R*,3*aR*,9*bS*)-1-acetyl-*N*-[[Rac-(3*S*)-2,3-dihydro-1,4-benzodioxin-3-yl]­methyl]-3,3*a*,4,9*b*-tetrahydro-2*H*-chromeno­[4,3-*b*]­pyrrole-3-carboxamide (**50**)

Prepared according to method C. The residue was purified by prep HPLC to afford a white solid, yield 20.4 mg (65%). ^1^H NMR (400 MHz, CDCl_3_) δ 7.64–7.75 (m, 1 H), 7.12–7.20 (m, 1 H), 6.85–6.98 (m, 5 H), 6.75–6.83 (m, 1 H), 6.22–6.34 (m, 1 H), 5.63–5.75 (m, 1 H), 4.33 (s, 3 H), 4.18 (d, J = 1.25 Hz, 1 H), 3.92–4.03 (m, 1 H), 3.86 (d, J = 10.04 Hz, 2 H), 3.62–3.72 (m, 1 H), 3.45–3.59 (m, 1 H), 3.15–3.32 (m, 1 H), 2.73–2.88 (m, 1 H), 2.12 (s, 3 H), *m*/*z* calcd for C_23_H_24_N_2_O_5_ [M+H^+^] 408.2, found 408.8.

#### 6.5.51 Rac-(3*R*,3*aR*,9*bS*)-*N*-(*m*-fluorobenzyl)-1-(1-methylsulfonyl)-3,3*a*,4,9*b*-tetrahydro-2*H*-chromeno­[4,3-*b*]­pyrrole-3-carboxamide (**51**)

Prepared according to method C. The residue was purified by prep HPLC to afford a white solid, yield 14 mg (54%). ^1^H NMR (400 MHz, MeOD) δ 7.64 (dt, *J* = 7.7, 1.2 Hz, 1H), 7.35 (td, *J* = 7.9, 5.9 Hz, 1H), 7.13 (td, *J* = 7.5, 5.8 Hz, 2H), 7.05–6.90 (m, 2H), 6.77 (dd, *J* = 8.2, 1.2 Hz, 1H), 5.18 (d, *J* = 7.8 Hz, 1H), 4.51–4.32 (m, 2H), 4.15 (dd, *J* = 12.1, 2.2 Hz, 1H), 3.65 (dd, *J* = 11.1, 8.5 Hz, 1H), 3.53 (dd, *J* = 11.0, 8.8 Hz, 1H), 3.32–3.23 (m, 4H), 3.08 (s, 3H), 2.91 (ddt, *J* = 10.2, 7.8, 2.2 Hz, 1H), *m*/*z* calcd for C_20_H_21_FN_2_O_4_S [M+H^+^] 404.1, found 405.2.

#### 6.5.52 Rac-(3*R*,3*aR*,9*bS*)-*N*-(*m*-fluorobenzyl)-1-(acetyl)-3,3*a*,4,9*b*-tetrahydro-2*H*-chromeno­[4,3-*b*]­pyrrole-3-carboxamide (**52**)

Prepared according to method C. The residue was purified by prep HPLC to afford a white solid, yield 5 mg (18%). ^1^H NMR (400 MHz, MeOD) δ 7.59–7.52 (m, 1H), 7.34 (td, *J* = 8.0, 5.8 Hz, 1H), 7.18–6.92 (m, 5H), 6.86 (td, *J* = 7.7, 1.3 Hz, 1H), 6.77 (dd, *J* = 8.3, 1.2 Hz, 1H), 5.63 (d, *J* = 7.8 Hz, 1H), 4.53–4.27 (m, 3H), 4.16 (qd, *J* = 12.1, 2.2 Hz, 2H), 3.77–3.68 (m, 1H), 3.44–3.32 (m, 1H), 2.83 (ddt, *J* = 10.1, 7.8, 2.1 Hz, 1H), 2.11 (s, 3H), *m*/*z* calcd for C_21_H_21_FN_2_O_3_ [M+H^+^] 368.2, found 369.0.

#### 6.5.53 Rac-(3*R*,3*aR*,9*bS*)-*N*-(*p*-fluorobenzyl)-1-(1-methylsulfonyl)-3,3*a*,4,9*b*-tetrahydro-2*H*-chromeno­[4,3-*b*]­pyrrole-3-carboxamide (**53**)

Prepared according to method C. The residue was purified by prep HPLC to afford a white solid, yield 3 mg (8%). ^1^H NMR (400 MHz, MeOD) δ 7.64 (dt, *J* = 7.7, 1.4 Hz, 1H), 7.39–7.25 (m, 3H), 7.16–7.11 (m, 1H), 7.06 (t, *J* = 8.8 Hz, 2H), 6.93 (ddd, *J* = 8.3, 7.3, 1.2 Hz, 1H), 6.77 (dd, *J* = 8.3, 1.2 Hz, 1H), 5.17 (d, *J* = 7.8 Hz, 1H), 4.39 (q, *J* = 14.8 Hz, 3H), 4.24–4.09 (m, 2H), 3.64 (dd, *J* = 11.0, 8.6 Hz, 1H), 3.26–3.18 (m, 1H), 3.08 (s, 3H), 2.91 (ddt, *J* = 10.2, 7.8, 2.2 Hz, 1H), *m*/*z* calcd for C_20_H_21_FN_2_O_4_S [M+H^+^] 404.1, found 405.1.

#### 6.5.54 Rac-(3*R*, *aR*,9*bS*)-*N*-(*p*-fluorobenzyl)-1-(acetyl)-3,3*a*,4,9*b*-tetrahydro-2*H*-chromeno­[4,3-*b*]­pyrrole-3-carboxamide (**54**)

Prepared according to method C. The residue was purified by prep HPLC to afford a white solid, yield 6 mg (55%). ^1^H NMR (400 MHz, MeOD) δ 7.55 (dt, *J* = 7.7, 1.3 Hz, 1H), 7.32 (ddd, *J* = 8.6, 5.4, 2.6 Hz, 2H), 7.14–7.01 (m, 3H), 6.86 (td, *J* = 7.5, 1.3 Hz, 1H), 6.77 (dd, *J* = 8.3, 1.2 Hz, 1H), 5.63 (d, *J* = 7.8 Hz, 1H), 4.49–4.32 (m, 3H), 4.15 (qd, *J* = 12.0, 2.1 Hz, 2H), 3.84–3.66 (m, 2H), 2.82 (ddt, *J* = 10.1, 7.8, 2.1 Hz, 1H), 2.11 (s, 3H), *m*/*z* calcd for C_21_H_21_FN_2_O_3_ [M+H^+^] 368.2, found 369.1.

#### 6.5.55 Rac-(3*R*,3*aR*,9*bS*)-*N*-(*m*-chlorobenzyl)-1-(1-methylsulfonyl)-3,3*a*,4,9*b*-tetrahydro-2*H*-chromeno­[4,3-*b*]­pyrrole-3-carboxamide (**55**)

Prepared according to method C. The residue was purified by prep HPLC to afford a white solid, yield 7 mg (54%). ^1^H NMR (400 MHz, MeOD) δ 7.64 (dt, *J* = 7.7, 1.2 Hz, 1H), 7.35 (td, *J* = 7.9, 5.9 Hz, 1H), 7.13 (td, *J* = 7.5, 5.8 Hz, 2H), 7.07–6.90 (m, 3H), 6.77 (dd, *J* = 8.2, 1.2 Hz, 1H), 5.18 (d, *J* = 7.8 Hz, 1H), 4.51–4.32 (m, 2H), 4.18 (qd, *J* = 12.1, 2.2 Hz, 2H), 3.65 (dd, *J* = 11.1, 8.5 Hz, 1H), 3.53 (dd, *J* = 11.0, 8.8 Hz, 1H), 3.29–3.20 (m, 1H), 3.08 (s, 3H), 2.91 (ddt, *J* = 10.2, 7.8, 2.2 Hz, 1H), *m*/*z* calcd for C_20_H_21_ClN_2_O_4_S [M+H^+^] 420.1, found 421.2.

#### 6.5.56 Rac-(3*R*,3*aR*,9*bS*)-*N*-(*m*-chlorobenzyl)-1-(acetyl)-3,3*a*,4,9*b*-tetrahydro-2*H*-chromeno­[4,3-*b*]­pyrrole-3-carboxamide (**56**)

Prepared according to method C. The residue was purified by prep HPLC to afford a white solid, yield 6 mg (25%). ^1^H NMR (400 MHz, MeOD) δ 7.56 (dt, *J* = 7.8, 1.4 Hz, 1H), 7.35–7.23 (m, 4H), 7.12 (td, *J* = 7.8, 1.7 Hz, 1H), 6.86 (td, *J* = 7.6, 1.4 Hz, 1H), 6.78 (dd, *J* = 8.3, 1.2 Hz, 1H), 5.64 (d, *J* = 7.8 Hz, 1H), 4.41 (dd, *J* = 7.9, 6.0 Hz, 2H), 4.17 (dd, *J* = 11.0, 2.1 Hz, 1H), 3.78–3.68 (m, 1H), 3.35 (d, *J* = 9.1 Hz, 1H), 2.88–2.72 (m, 1H), 2.12 (d, *J* = 3.7 Hz, 3H), *m*/*z* calcd for C_21_H_21_ClN_2_O_3_ [M+H^+^] 384.1, found 385.1.

#### 6.5.57 Rac-(3*R*,3*aR*,9*bS*)-*N*-(*p*-chlorobenzyl)-1-(1-methylsulfonyl)-3,3*a*,4,9*b*-tetrahydro-2*H*-chromeno­[4,3-*b*]­pyrrole-3-carboxamide (**57**)

Prepared according to method C. The residue was purified by prep HPLC to afford a white solid, yield 8 mg (40%). ^1^H NMR (400 MHz, MeOD) δ 7.64 (dt, *J* = 7.7, 1.4 Hz, 1H), 7.37–7.22 (m, 4H), 7.14 (dddd, *J* = 8.1, 7.3, 1.7, 0.7 Hz, 1H), 6.93 (ddd, *J* = 8.3, 7.3, 1.2 Hz, 1H), 6.77 (dd, *J* = 8.2, 1.2 Hz, 1H), 5.17 (d, *J* = 7.8 Hz, 1H), 4.47–4.33 (m, 2H), 4.17 (qd, *J* = 12.1, 2.1 Hz, 2H), 3.64 (dd, *J* = 11.1, 8.5 Hz, 1H), 3.51 (dd, *J* = 11.1, 8.8 Hz, 1H), 3.30–3.21 (m, 1H), 3.08 (s, 3H), 2.90 (ddt, *J* = 10.2, 7.8, 2.1 Hz, 1H), *m*/*z* calcd for C_20_H_21_ClN_2_O_4_S [M+H^+^] 420.1.

#### 6.5.58 Rac-(3*R*,3*aR*,9*bS*)-*N*-(*p*-chlorobenzyl)-1-(acetyl)-3,3*a*,4,9*b*-tetrahydro-2*H*-chromeno­[4,3-*b*]­pyrrole-3-carboxamide (**58**)

Prepared according to method C. The residue was purified by prep HPLC to afford a white solid, yield 4 mg (27%). ^1^H NMR (400 MHz, MeOD) δ 7.55 (d, *J* = 7.6 Hz, 1H), 7.38–7.26 (m, 6H), 6.86 (td, *J* = 7.5, 1.3 Hz, 2H), 6.77 (dd, *J* = 8.3, 1.2 Hz, 1H), 5.63 (d, *J* = 7.7 Hz, 2H), 4.39 (d, *J* = 9.5 Hz, 3H), 4.16 (dd, *J* = 10.7, 2.1 Hz, 2H), 3.94–3.67 (m, 3H), 3.34 (d, *J* = 9.1 Hz, 1H), 2.82 (dd, *J* = 10.7, 8.0 Hz, 1H), 2.11 (s, 3H), *m*/*z* calcd for C_21_H_21_ClN_2_O_3_ [M+H^+^] 384.1, found 385.0.

#### 6.5.59 Rac-(3*R*,3*aR*,9*bS*)-*N*-(cyclohexylmethyl)-1,2,3,3*a*,4,9*b*-hexahydrochromeno­[4,3-*b*]­pyrrole-3-carboxamide (**59**)

Prepared according to method C. The residue was purified by prep HPLC to afford a white solid, yield 26.8 mg (40%). ^1^H NMR (400 MHz, CDCl_3_) δ 7.30–7.41 (m, 1 H), 7.09–7.21 (m, 1 H), 6.92–7.02 (m, 1 H), 6.79–6.90 (m, 1 H), 5.75–5.95 (m, 1 H), 4.39 (d, J = 7.53 Hz, 1 H), 4.14 (d, J = 3.76 Hz, 1 H), 3.95 (d, J = 6.27 Hz, 1 H), 3.03–3.24 (m, 4 H), 2.78–2.92 (m, 1 H), 2.54–2.72 (m, 1 H), 2.03 (br s, 2 H), 1.58–1.83 (m, 5 H), 1.37–1.55 (m, 1 H), 1.21 (d, J = 12.30 Hz, 3 H), 0.95 (dd, J = 11.80, 2.51 Hz, 2 H), *m*/*z* calcd for C_19_H_26_N_2_O_2_ [M+H^+^] 314.2, found 315.2.

#### 6.5.60 Rac-(3*R*,3*aR*,9*bS*)-*N*-(cyclohexylmethyl)-1-methyl-3,3*a*,4,9*b*-tetrahydro-2*H*-chromeno­[4,3-*b*]­pyrrole-3-carboxamide (**60**)

Prepared according to method C. The residue was purified by prep HPLC to afford a white solid, yield 14.1 mg (68%). ^1^H NMR (400 MHz, CDCl_3_) δ 7.17–7.25 (m, 2 H), 6.88–7.00 (m, 2 H), 5.58–5.81 (m, 1 H), 3.94–4.10 (m, 2 H), 3.36–3.48 (m, 1 H), 3.20–3.28 (m, 1 H), 3.15 (s, 2 H), 2.80–2.89 (m, 1 H), 2.65–2.73 (m, 1 H), 2.55–2.64 (m, 1 H), 2.49 (s, 3 H), 1.73 (d, J = 11.29 Hz, 5 H), 1.41–1.57 (m, 1 H), 1.11–1.33 (m, 3 H), 0.86–1.05 (m, 2 H), *m*/*z* calcd for C_20_H_28_N_2_O_2_ [M+H^+^] 328.2, found 329.2.

#### 6.5.61 Rac-(3*R*,3*aR*,9*bS*)-*N*-(cyclohexylmethyl)-1-[(4-methoxyphenyl)­methyl]-3,3*a*,4,9*b*-tetrahydro-2*H*-chromeno­[4,3-*b*]­pyrrole-3-carboxamide (**61**)

Prepared according to method C. The residue was purified by prep HPLC to afford a white solid, yield 200.0 mg (62%). ^1^H NMR (400 MHz, CDCl_3_) δ 7.17–7.28 (m, 4 H), 6.88–6.99 (m, 2 H), 6.84 (d, J = 8.53 Hz, 2 H), 5.70–5.89 (m, 1 H), 4.10–4.21 (m, 2 H), 4.01–4.09 (m, 1 H), 3.80 (s, 4 H), 3.42–3.52 (m, 1 H), 3.04–3.14 (m, 2 H), 2.92–3.02 (m, 1 H), 2.83–2.91 (m, 1 H), 2.51–2.63 (m, 2 H), 1.62–1.80 (m, 5 H), 1.38–1.53 (m, 1 H), 1.10–1.35 (m, 4 H), 0.811.01 (m, 2 H), *m*/*z* calcd for C_27_H_34_N_2_O_3_ [M+H^+^] 434.3, found 435.2.

#### 6.5.62 Rac-(3*R*,3*aR*,9*bS*)-*N*-(cyclohexylmethyl)-1-propanoyl-3,3*a*,4,9*b*-tetrahydro-2*H*-chromeno­[4,3-*b*]­pyrrole-3-carboxamide (**62**)

Prepared according to method C. The residue was purified by prep HPLC to afford a white solid, yield 12.5 mg (46%). ^1^H NMR (400 MHz, CDCl_3_) δ 7.66–7.78 (m, 1 H), 7.12–7.24 (m, 1 H), 6.89–7.00 (m, 1 H), 6.75–6.87 (m, 1 H), 5.65–5.82 (m, 2 H), 4.20 (d, J = 1.25 Hz, 2 H), 3.80–3.95 (m, 1 H), 3.55–3.72 (m, 1 H), 3.04–3.29 (m, 3 H), 2.73–2.87 (m, 1 H), 2.34–2.51 (m, 1 H), 2.15–2.33 (m, 1 H), 1.64–1.89 (m, 5 H), 1.40–1.56 (m, 1 H), 1.38–1.22 (m, 6 H), 0.87–1.04 (m, 2 H), *m*/*z* calcd for C_22_H_30_N_2_O_3_ [M+H^+^] 370.2, found 371.2.

#### 6.5.63 Rac-(3*R*,3*aR*,9*bS*)-*N*-(cyclohexylmethyl)-1-(cyclopropanecarbonyl)-3,3*a*,4,9*b*-tetrahydro-2*H*-chromeno­[4,3-*b*]­pyrrole-3-carboxamide (**63**)

Prepared according to method A. The residue was purified by prep HPLC to afford a white solid, yield 10.7 mg (40%). ^1^H NMR (400 MHz, CDCl_3_) δ 7.67 (d, J = 7.78 Hz, 1 H), 7.08–7.24 (m, 1 H), 6.89–7.02 (m, 1 H), 6.75–6.88 (m, 1 H), 5.41–5.86 (m, 2 H), 4.21 (d, J = 1.51 Hz, 2 H), 3.90–4.14 (m, 1 H), 3.49–3.89 (m, 1 H), 3.21 (br s, 3 H), 2.75–3.05 (m, 1 H), 1.62–1.84 (m, 5 H), 1.43–1.56 (m, 1 H), 0.74–1.37 (m, 9 H), *m*/*z* calcd for C_23_H_30_N_2_O_3_ [M+H^+^] 382.2, found 383.4.

#### 6.5.64 Rac-(3*R*,3*aR*,9*bS*)-1-benzoyl-*N*-(cyclohexylmethyl)-3,3*a*,4,9*b*-tetrahydro-2*H*-chromeno­[4,3-*b*]­pyrrole-3-carboxamide (**64**)

Prepared according to method A. The residue was purified by prep HPLC to afford a white solid, yield 5.7 mg (22%). ^1^H NMR (400 MHz, CDCl_3_) δ 7.68–7.80 (m, 1 H), 7.42 (br s, 5 H), 7.14–7.26 (m, 1 H), 6.94–7.06 (m, 1 H), 6.75–6.91 (m, 1 H), 5.91–6.09 (m, 1 H), 5.58–5.74 (m, 1 H), 4.12–4.34 (m, 2 H), 3.56–3.80 (m, 2 H), 3.01–3.22 (m, 3 H), 2.81–2.98 (m, 1 H), 1.63–1.82 (m, 5 H), 1.35–1.53 (m, 1 H), 1.07–1.32 (m, 3 H), 0.81–1.04 (m, 2 H), *m*/*z* calcd for C_26_H_30_N_2_O_3_ [M+H^+^] 418.2, found 419.4.

#### 6.5.65 Rac-(3*R*,3*aR*,9*bS*)-*N*-(cyclohexylmethyl)-1-cyclopropylsulfonyl-3,3*a*,4,9*b*-tetrahydro-2*H*-chromeno­[4,3-*b*]­pyrrole-3-carboxamide (**65**)

Prepared according to method A. The residue was purified by prep HPLC to afford a white solid, yield 15.9 mg (48%). ^1^H NMR (400 MHz, CDCl_3_) δ 7.65–7.78 (m, 1 H), 7.13–7.23 (m, 1 H), 6.97–7.05 (m, 1 H), 6.74–6.84 (m, 1 H), 5.64–5.85 (m, 1 H), 5.20 (d, J = 7.53 Hz, 1 H), 4.23 (s, 2 H), 3.83–3.95 (m, 1 H), 3.48–3.64 (m, 1 H), 3.17 (t, J = 6.40 Hz, 2 H), 3.07–3.14 (m, 1 H), 2.98–3.06 (m, 1 H), 2.59–2.75 (m, 1 H), 1.65–1.82 (m, 5 H), 1.42–1.56 (m, 1 H), 1.03–1.40 (m, 7 H), 0.88–1.02 (m, 2 H), *m*/*z* calcd for C_22_H_32_N_2_O_4_S [M+H^+^] 418.2, found 419.4.

#### 6.5.66 Rac-(3*R*,3*aR*,9*bS*)-*N*-(cyclohexylmethyl)-1-(sulfonicacid)-3,3*a*,4,9*b*-tetrahydro-2*H*-chromeno­[4,3-*b*]­pyrrole-3-carboxamide (**66**)

Prepared according to method A. The residue was purified by prep HPLC to afford a white solid, yield 6 mg (29%). ^1^H NMR (400 MHz, DMSO) δ 8.26 (t, *J* = 5.8 Hz, 1H), 7.70 (dd, *J* = 7.9, 1.7 Hz, 1H), 7.28–7.19 (m, 1H), 7.05–6.95 (m, 1H), 6.84 (d, *J* = 8.2 Hz, 1H), 5.14 (d, *J* = 8.7 Hz, 1H), 4.14 (dd, *J* = 11.9, 2.6 Hz, 1H), 3.58 (dd, *J* = 11.0, 7.6 Hz, 1H), 3.47 (t, *J* = 11.0 Hz, 2H), 3.09–2.82 (m, 5H), 1.72–1.57 (m, 1H), 1.41 (ddp, *J* = 11.4, 7.4, 3.7 Hz, 4H), 1.27–1.07 (m, 1H), 0.87 (qd, *J* = 12.5, 3.9 Hz, 2H), *m*/*z* calcd for C_19_H_26_N_2_O_5_S [M+H^+^] 394.2, found 394.8.

#### 6.5.67 Rac-(3*R*,3*aR*,9*bS*)-*N*-(cyclohexylmethyl)-1-(sulfamoyl)-3,3*a*,4,9*b*-tetrahydro-2*H*-chromeno­[4,3-*b*]­pyrrole-3-carboxamide (**67**)

Prepared according to method A. The residue was purified by prep HPLC to afford a white solid, yield 6 mg (21%). ^1^H NMR (400 MHz, MeOD) δ 7.69 (dd, *J* = 7.8, 1.6 Hz, 1H), 7.16–7.09 (m, 1H), 6.96–6.87 (m, 1H), 6.76 (d, *J* = 8.2 Hz, 1H), 5.16 (d, *J* = 7.9 Hz, 1H), 4.24–4.04 (m, 2H), 3.60 (t, *J* = 9.7 Hz, 1H), 3.41 (t, *J* = 9.2 Hz, 1H), 3.28–3.16 (m, 1H), 3.07 (ddtd, *J* = 20.2, 13.4, 6.8, 4.5 Hz, 2H), 2.93 (ddt, *J* = 10.5, 8.1, 2.3 Hz, 1H) 1.80–1.65 (m, 7H), 1.49 (ddh, *J* = 10.7, 7.1, 3.5 Hz, 1H), 1.34–1.15 (m, 4H), 1.01–0.88 (m, 3H).1.80–1.65 (m, 6H), 1.56–1.43 (m, 1H), 1.34–1.15 (m, 3H), 1.01–0.88 (m, 3H), *m*/*z* calcd for C_20_H_29_N_3_O_4_S [M+H^+^] 407.5 found 408.1.

#### 6.5.68 Rac-(3*R*,3*aR*,9*bS*)-*N*-(cyclohexylmethyl)-1-(amino acetyl)-3,3*a*,4,9*b*-tetrahydro-2*H*-chromeno­[4,3-*b*]­pyrrole-3-carboxamide (**68**)

Prepared according to method C, using HATU as an alternative coupling agent. The residue was purified by prep HPLC to afford a white solid, yield 15 mg (40%). ^1^H NMR (400 MHz, MeOD) δ 8.40 (s, 1H), 7.58 (d, *J* = 7.1 Hz, 1H), 7.15 (t, *J* = 7.8 Hz, 1H), 6.87 (t, *J* = 7.1 Hz, 1H), 6.81 (d, *J* = 8.3 Hz, 1H), 5.69 (d, *J* = 7.9 Hz, 1H), 4.26–4.11 (m, 2H), 3.98 (d, *J* = 16.3 Hz, 1H), 3.82 (d, *J* = 16.4 Hz, 1H), 3.75–3.61 (m, 2H), 3.37–3.32 (m, 1H), 3.19–3.12 (m, 1H), 3.10–2.98 (m, 1H), 2.82 (ddt, *J* = 10.3, 7.9, 2.2 Hz, 1H), 1.82–1.66 (m, 6H), 1.57–1.45 (m, 1H), 1.35–1.16 (m, 4H), 1.02–0.93 (m, 1H), *m*/*z* calcd for C_21_H_29_N_3_O_3_ [M+H^+^] 371.2, found 373.0.

#### 6.5.69 Rac-(3*R*,3*aR*,9*bS*)-*N*-(cyclohexylmethyl)-1-(hydroxy acetyl)-3,3*a*,4,9*b*-tetrahydro-2*H*-chromeno­[4,3-*b*]­pyrrole-3-carboxamide (**69**)

Prepared according to method C, using HATU as an alternative coupling agent. The residue was purified by prep HPLC to afford a white solid, yield 6 mg (29%). ^1^H NMR (400 MHz, MeOD) δ 7.59 (d, *J* = 7.6 Hz, 1H), 7.18–7.06 (m, 1H), 6.86 (td, *J* = 7.5, 1.2 Hz, 1H), 6.79 (d, *J* = 1.2 Hz, 1H), 5.67 (d, *J* = 7.8 Hz, 1H), 4.33–4.01 (m, 4H), 3.62 (dd, *J* = 9.1, 2.3 Hz, 1H), 3.35–3.22 (m, 1H), 3.12 (dd, *J* = 13.3, 6.9 Hz, 1H), 3.01 (dd, *J* = 13.3, 6.8 Hz, 1H), 2.79 (ddt, *J* = 10.2, 7.7, 2.2 Hz, 0H), 1.84–1.61 (m, 2H), 1.49 (dtt, *J* = 10.7, 6.9, 3.6 Hz, 1H), 1.35–1.12 (m, 2H), 1.02–0.88 (m, 1H), *m*/*z* calcd for C_21_H_28_N_2_O_4_ [M+H^+^] 372.2, found 373.1.

#### 6.5.70 Rac-(3*R*,3*aR*,9*bS*)-*N*-(cyclohexylmethyl)-1-(2,2-diethoxyacetyl)-3,3*a*,4,9*b*-tetrahydro-2*H*-chromeno­[4,3-*b*]­pyrrole-3-carboxamide (**70**)

Prepared according to method C, using HATU as an alternative coupling agent. The residue was purified by prep HPLC to afford a white solid, yield 20 mg (77%). ^1^H NMR (400 MHz, MeOD) δ 7.52 (d, *J* = 7.5 Hz, 1H), 7.18–7.10 (m, 1H), 6.86 (td, *J* = 7.5, 1.2 Hz, 1H), 6.80 (d, *J* = 1.2 Hz, 1H), 5.68 (d, *J* = 7.8 Hz, 1H), 4.26–4.09 (m, 3H), 3.97 (dd, *J* = 11.1, 8.9 Hz, 1H), 3.93–3.54 (m, 8H), 3.31–3.22 (m, 1H), 3.17–2.98 (m, 3H), 2.80 (ddt, *J* = 10.2, 7.8, 2.1 Hz, 1H), 1.82–1.64 (m, 8H), 1.50 (tq, *J* = 10.2, 4.2 Hz, 1H), 1.35–1.12 (m, 14H), 1.00–0.90 (m, 3H), *m*/*z* calcd for C_25_H_36_N_2_O_5_ [M+H^+^] 444.3, found 445.1.

#### 6.5.71 Rac-(3*R*,3*aR*,9*bS*)-*N*-(cyclohexylmethyl)-1-(2,2-dihyrdoxyacetyl)-3,3*a*,4,9*b*-tetrahydro-2*H*-chromeno­[4,3-*b*]­pyrrole-3-carboxamide (**71**)

Prepared according to method C, using HATU as an alternative coupling agent. The residue was purified by prep HPLC to afford a white solid, yield 5 mg (46%). ^1^H NMR (400 MHz, MeOD) δ 7.63 (dd, *J* = 7.8, 1.6 Hz, 1H), 7.16–7.07 (m, 1H), 6.87 (td, *J* = 7.5, 1.2 Hz, 1H), 6.76 (dd, *J* = 8.2, 1.2 Hz, 1H), 5.48 (d, *J* = 7.8 Hz, 1H), 4.20 (dd, *J* = 12.0, 2.1 Hz, 1H), 4.11 (dd, *J* = 12.0, 2.0 Hz, 2H), 3.48 (dd, *J* = 9.2, 3.5 Hz, 1H), 3.29–3.19 (m, 2H), 3.17–2.94 (m, 1H), 2.85–2.74 (m, 1H), 1.f78–1.65 (m, 5H), 1.55–1.43 (m 1H), 1.34–1.13 (m, 1H), 0.98–0.87 (m, 2H), *m*/*z* calcd for C_21_H_28_N_2_O_5_ [M+H^+^] 388.2, found 388.1.

#### 6.5.72 Rac-(3*R*,3*aR*,9*bS*)-*N*-(cyclohexylmethyl)-1-(methylcarboxylate)-3,3*a*,4,9*b*-tetrahydro-2*H*-chromeno­[4,3-*b*]­pyrrole-3-carboxamide (**72**)

Prepared according to method A. The residue was purified by prep HPLC to afford a white solid, yield 14 mg (44%). ^1^H NMR (400 MHz, MeOD) δ 7.52 (d, *J* = 64.3 Hz, 1H), 7.17–7.09 (m, 1H), 6.78 (dd, *J* = 8.3, 1.2 Hz, 1H), 5.34 (d, *J* = 7.8 Hz, 1H), 4.19 (dd, *J* = 12.1, 2.2 Hz, 1H), 4.10 (dd, *J* = 12.1, 2.1 Hz, 1H), 3.80 (d, *J* = 26.8 Hz, 3H), 3.61 (d, *J* = 10.0 Hz, 1H), 3.50 (d, *J* = 12.0 Hz, 1H), 3.20 (d, *J* = 9.9 Hz, 1H), 3.14–2.97 (m, 2H), 2.86 (t, *J* = 9.6 Hz, 1H), 1.79–1.65 (m, 5H), 1.51–1.47 (m, 1H), 1.35–1.13 (m, 3H), 1.02–0.88 (m, 2H), *m*/*z* calcd for C_21_H_28_N_2_O_4_ [M+H^+^] 372.2, found 373.0.

#### 6.5.73 Rac-(3*R*,3*aR*,9*bS*)-*N*-(cyclohexylmethyl)-1-(ethylcarboxylate)-3,3*a*,4,9*b*-tetrahydro-2*H*-chromeno­[4,3-*b*]­pyrrole-3-carboxamide (**73**)

Prepared according to method A. The residue was purified by prep HPLC to afford a white solid, yield 5 mg (41%). ^1^H NMR (400 MHz, MeOD) δ 7.57 (d, *J* = 41.8 Hz, 1H), 7.20–7.11 (m, 1H), 6.91 (t, *J* = 7.6 Hz, 1H), 6.80 (dd, *J* = 8.2, 1.3 Hz, 1H), 5.36 (d, *J* = 7.8 Hz, 1H), 4.21 (dd, *J* = 12.0, 2.2 Hz, 1H), 4.12 (dd, *J* = 12.1, 2.2 Hz, 1H), 3.65 (t, *J* = 10.1 Hz, 2H), 3.57–3.48 (s, 1H), 3.22 (q, *J* = 9.8 Hz, 1H), 3.16–2.99 (m, 2H), 2.87 (q, *J* = 9.8 Hz, 1H), 1.82–1.66 (m, 4H), 1.53–1.48 (m, 1H), 1.42–1.16 (m, 6H), 1.05–0.83 (m, 1H), *m*/*z* calcd for C_22_H_30_N_2_O_4_ [M+H^+^] 386.2, found 387.1.

#### 6.5.74 Rac-(3*R*,3*aR*,9*bS*)-*N*-(cyclohexylmethyl)-1-(carbamide)-3,3*a*,4,9*b*-tetrahydro-2*H*-chromeno­[4,3-*b*]­pyrrole-3-carboxamide (**74**)

Prepared according to method D. The residue was purified by prep HPLC to afford a white solid, yield 10 mg (45%). ^1^H NMR (400 MHz, MeOD) δ 8.33–8.29 (m, 1H), 7.62 (dt, *J* = 7.7, 1.3 Hz, 1H), 7.11 (ddd, *J* = 8.5, 7.3, 1.7 Hz, 1H), 6.89 (dd, *J* = 7.6, 1.2 Hz, 1H), 6.76 (dd, *J* = 8.2, 1.2 Hz, 1H), 5.42 (d, *J* = 7.7 Hz, 1H), 4.21 (dd, *J* = 12.1, 2.1 Hz, 1H), 4.11 (dd, *J* = 12.0, 2.0 Hz, 2H), 3.30–3.21 (m, 1H), 3.17–2.97 (m, 2H), 2.83 (ddt, *J* = 11.5, 7.7, 2.1 Hz, 1H), 1.90–1.63 (m, 6H), 1.53–1.47 (m, 1H), 1.35–1.15 (m, 4H), 1.02–0.86 (m, 2H), *m*/*z* calcd for C_20_H_27_N_3_O_3_ [M+H^+^] 357.2, found 358.1.

#### 6.5.75 Rac-(3*R*,3*aR*,9*bS*)-*N*-(cyclohexylmethyl)-1-(*N*3-methylcarbamide)-3,3*a*,4,9*b*-tetrahydro-2*H*-chromeno­[4,3-*b*]­pyrrole-3-carboxamide (**75**)

Prepared according to method D. The residue was purified by prep HPLC to afford a white solid, yield 3 mg (57%). ^1^H NMR (400 MHz, MeOD) δ 7.60 (dd, *J* = 7.7, 1.3 Hz, 1H), 7.16–7.08 (m, 1H), 6.88 (td, *J* = 7.5, 1.2 Hz, 1H), 6.77 (dd, *J* = 8.3, 1.2 Hz, 1H), 5.45 (d, *J* = 7.8 Hz, 1H), 4.22 (dd, *J* = 12.1, 2.1 Hz, 1H), 4.12 (dd, *J* = 12.0, 2.0 Hz, 1H), 3.50 (p, *J* = 9.3 Hz, 2H), 3.27 (dt, *J* = 11.3, 9.6 Hz, 1H), 3.08 (qd, *J* = 13.3, 6.9 Hz, 2H), 2.81 (s, 3H), 1.82–1.65 (m, 5H), 1.54–1.46 (m, 1H), 1.37–1.13 (m, 4H), 1.03–0.88 (m, 3H), *m*/*z* calcd for C_21_H_29_N_3_O_3_ [M+H^+^] 371.2, found 372.1.

#### 6.5.76 Rac-(3*R*,3*aR*,9*bS*)-*N*-(cyclohexylmethyl)-1-(*N*3,*N*3-diemthylcarbamide)-3,3*a*,4,9*b*-tetrahydro-2*H*-chromeno­[4,3-*b*]­pyrrole-3-carboxamide (**76**)

Prepared according to method D. The residue was purified by prep HPLC to afford a white solid, yield 3 mg (28%). ^1^H NMR (400 MHz, MeOD) δ 0.29 (d, *J* = 7.8 Hz, 1H), 7.16–7.05 (m, 1H), 6.87 (t, *J* = 7.5 Hz, 1H), 6.78 (d, *J* = 8.2 Hz, 1H), 5.55 (d, *J* = 7.6 Hz, 1H), 4.24 (dd, *J* = 11.7, 2.9 Hz, 1H), 4.04 (dd, *J* = 11.7, 4.4 Hz, 1H), 3.67 (dd, *J* = 10.4, 8.5 Hz, 1H), 3.47 (dd, *J* = 10.4, 6.1 Hz, 1H), 3.14–2.96 (m, 4H), 2.89 (s, 6H), 2.82–2.73 (m, 1H), 1.81–1.62 (m, 5H), 1.54–1.43 (m, 1H), 1.34–1.15 (m, 5H), 1.05–0.88 (m, 3H), *m*/*z* calcd for C_22_H_31_N_3_O_3_ [M+H^+^] 385.2, found 386.0.

#### 6.5.77 Rac-(3*R*,3*aR*,9*bS*)-*N*-(cyclohexylmethyl)-1-(methoxycarbamide)-3,3*a*,4,9*b*-tetrahydro-2*H*-chromeno­[4,3-*b*]­pyrrole-3-carboxamide (**77**)

The chromenopyrrole (**77**) TFA salt (40 mg, 0.1 mmol, 1.0 equiv) was added to a solution of DMF (1.0 mL) and mixed. To the solution were added Et_3_N (51 μL, 0.4 mmol, 4.0 equiv), DMAP (1.2 mg, 0.01 mmol, 0.1 equiv), and *N*-methyl-*O*-benzoylhydroxylamine (62 mg, 0.4 mmol, 4.0 equiv) and stirred at r.t. overnight. The crude mixture was concentrated *in* *vacuo* and advanced to purification. The residue was prepared by reverse-phase flash column chromatography (5–95% MeCN in H_2_O) to give a white powder, yield 36 mg (47%). ^1^H NMR (400 MHz, MeOD) δ 7.60 (d, J = 7.7 Hz, 1H), 7.17–7.06 (m, 1H), 6.88 (t, J = 7.6 Hz, 1H), 6.77 (dd, J = 8.2, 1.2 Hz, 1H), 5.47 (d, J = 7.9 Hz, 1H), 4.89–4.85 (m, 1H), 4.19 (dd, J = 12.0, 2.1 Hz, 1H), 4.10 (dd, J = 12.1, 2.0 Hz, 1H), 3.70 (s, 3H), 3.32–3.20 (m, 1H), 3.10 (dd, J = 13.3, 6.9 Hz, 1H), 3.02 (dd, J = 13.3, 6.8 Hz, 1H), 2.84–2.74 (m, 1H), 1.80–1.62 (m, 6H), 1.57–1.44 (m, 1H), 1.34–1.12 (m, 3H), 1.01–0.85 (m, 2H), *m*/*z* calcd for C_21_H_29_N_3_O_4_ [M+H^+^] 387.2, found 388.7.

#### 6.5.78 Rac-(3*R*,3*aR*,9*bS*)-*N*-(cyclohexylmethyl)-1-(benzyloxycarbamide)-3,3*a*,4,9*b*-tetrahydro-2*H*-chromeno­[4,3-*b*]­pyrrole-3-carboxamide (**78**)

Carbodiimide (71 mg, 0.44 mmo, 5.5 equiv) was suspended in dry CH_2_Cl_2_ (1.2 mL) under argon gas. To this solution were added *N*-benzyloxamine (47 μL, 0.4 mmol, 5.0 equiv) and DIPEA (9 μL, 0.53 mmol) and left to stir at r.t. for 2 h. Chromenopyrrole (35 mg, 0.08 mmol, 1.0 equiv) was then added and left to stir under argon at r.t. overnight. The crude mixture was portioned between water (5 mL) and washed with CH_2_Cl_2_ (3 × 100 mL). The organic layer was dried over Na_2_SO_4_ and filtered *in* *vacuo*. The residue was prepared by flash column chromatography (0–10% MeOH in CH_2_Cl_2_) affording a yellow oil (38 mg, 56%); ^1^H NMR (400 MHz, CDCl_3_) δ 7.73 (dt, *J* = 7.8, 1.2 Hz, 1H), 7.44–7.28 (m, 5H), 7.13 (ddd, *J* = 8.5, 7.3, 1.7 Hz, 1H), 6.91 (td, *J* = 7.5, 1.3 Hz, 1H), 6.76 (dd, *J* = 8.3, 1.2 Hz, 1H), 5.83 (t, *J* = 6.0 Hz, 1H), 5.54 (d, *J* = 7.9 Hz, 1H), 4.94 (d, *J* = 11.4 Hz, 1H), 4.86 (d, *J* = 11.4 Hz, 1H), 4.18–4.07 (m, 2H), 3.58 (t, *J* = 9.4 Hz, 1H), 3.33 (t, *J* = 8.8 Hz, 1H), 3.21–3.02 (m, 2H), 2.78 (ddt, *J* = 11.5, 7.9, 2.0 Hz, 1H), 1.79–1.65, (m, 6H), 1.51–1.41 (m, 1H), 1.32–1.09 (m, 4H), 0.99–0.86 (m, 2H), *m*/*z* calcd for C_27_H_33_N_3_O_4_ [M+H^+^] 463.3, found 464.1.

#### 6.5.79 Rac-(3*R*,3*aR*,9*bS*)-*N*-(cyclohexylmethyl)-1-(hydroxycarbamide)-3,3*a*,4,9*b*-tetrahydro-2*H*-chromeno­[4,3-*b*]­pyrrole-3-carboxamide (**79**)

Chromenopyrrole **78** (40 mg, 0.08 mmol, 1.0 equiv) was suspended in MeOH (1 mL) and then flushed with argon. Pd­(OH)_2_/C (10 mg) was added, and H_2_ was bubbled through the system at r.t. for 3 h. The crude mixture was filtered through Celite and washed with MeOH (3 × 3 mL). The crude mixture was concentrated *in* *vacuo* and advanced to purification. The residue was prepared by reverse-phase flash column chromatography (595% MeCN in H_2_O) to give a white powder (35 mg, 40%). ^1^H NMR (400 MHz, MeOD) δ 7.62 (d, *J* = 7.0 Hz, 1H), 7.21–7.08 (m, 1H), 6.87 (td, *J* = 7.6, 1.2 Hz, 1H), 6.76 (d, *J* = 9.2 Hz, 1H), 5.48 (d, *J* = 7.8 Hz, 1H), 4.20 (dd, *J* = 12.1, 2.2 Hz, 1H), 4.11 (dd, *J* = 12.0, 2.0 Hz, 1H), 3.50–3.44 (m, 2H), 3.31–3.20 (m, 2H), 3.11 (dd, *J* = 13.3, 6.9 Hz, 1H), 3.02 (dd, *J* = 13.3, 6.8 Hz, 1H), 2.81 (tdt, *J* = 10.3, 7.9, 2.1 Hz, 1H), 1.81–1.64 (m, 6H), 1.54–1.44 (m, 1H), 1.39–1.12 (m, 3H), 1.02–0.88 (m, 2H), *m*/*z* calcd for C_20_H_27_N_3_O_4_ [M+H^+^] 373.2, found 373.2.

#### 6.5.80 Synthesis of Rac-(3*R*,3*aS*,9*bS*)-*N*-(cyclohexylmethyl)-1-methylsulfonyl-2,3,3*a*,4,5,9*b*-hexahydropyrrolo­[3,2-*c*]­quinoline-3-carboxamide (**80**)

Rac-(3*R*,3*aS*,9*bS*)-1-methylsulfonyl-2,3,3*a*,4,5,9*b*-hexahydropyrrolo­[3,2-*c*]­quinoline-3-carboxylic acid (126 mg, 0.25 mmol, 1.0 equiv) was mixed in CH_2_Cl_2_ (2.0 mL), the T3P (50.0%, 0.37 mmol, 1.5 equiv) was added, and the resulting solution was stirred for 30 min. The cylcohexylamine (36.4 mg, 0.32 mmol, 1.3 equiv) and DIPEA (0.17 mL, 0.99 mmol, 4.0 equiv) in (2.0 mL) were added, and the solution was stirred overnight. The mixture was partitioned between water (5 mL) and CH_2_Cl_2_ (2 × 5 mL) and passed through a hydrophobic frit. The residue was then purified by flash column chromatography (0–2% MeOH in CH_2_Cl_2_) to give an off-white solid (93.1 mg, 96%). ^1^H NMR (400 MHz, CDCl_3_) δ 7.65–7.73 (m, 1H), 7.03–7.16 (m, 1H), 6.79–6.92 (m, 1H), 6.51–6.66 (m, 1H), 5.80–6.03 (m, 1H), 5.04–5.15 (m, 1H), 3.65–3.79 (m, 1H), 3.45–3.62 (m, 2H), 3.16 (s, 4H), 3.05 (s, 3H), 2.80–2.93 (m, 1H), 1.63–1.81 (m, 5H), 1.41–1.56 (m, 1H), 1.10–1.34 (m, 3H), 0.86–1.06 (m, 2H), *m*/*z* calcd for C_20_H_29_N_3_O_3_S [M+H^+^] 391.2, found 392.2.

#### 6.5.81 Rac-(3*R*,3*aS*,9*bS*)-*N*-(cyclohexylmethyl)-1-methylsulfonyl-2,3,3*a*,4,5,9*b*-hexahydropyrrolo­[3,2-*c*]­quinoline-3-carboxamide (**81**)

Rac-(3*R*,3*aS*,9*bS*)-*N*-(cyclohexylmethyl)-1-methylsulfonyl-2,3,3*a*,4,5,9*b*-hexahydropyrrolo­[3,2-*c*]­quinoline-3-carboxamide (300 mg, 0.8 mmol, 1.0 equiv) was suspended in methanol (1 mL), and formaldehyde (37.0%, 288.0 μL, 3.83 mmol, 4.7 equiv) was added followed by acetic acid (10.0 μL). The solution was stirred at room temperature for 1 h. To this mixture, sodium triacetoxyborohydride (325 mg, 1.53 mmol, 2.0 equiv) was added portion wise. The mixture was concentrated *in* *vacuo.* Resuspension was performed in water and saturated with NaHCO_3_. The aqueous solution was washed with CH_2_Cl_2_ (2 × 10 mL). Combined organics were dried over Na_2_SO_4_, filtered, and concentrated *in* *vacuo.* The residue was then purified by flash column chromatography (0–2% MeOH in CH_2_Cl_2_) to give an off-white solid (14.1 mg, 45%). ^1^H NMR (400 MHz, CDCl_3_) δ 7.63–7.78 (m, 1H), 7.21 (s, 1H), 6.80–6.95 (m, 1H), 6.62–6.76 (m, 1H), 5.65–5.99 (m, 1H), 5.10 (d, J = 7.5 Hz, 1H), 3.65–3.75 (m, 1H), 3.51–3.62 (m, 1H), 3.35–3.46 (m, 1H), 3.16 (dd, J = 13.2, 6.7 Hz, 4H), 3.03 (s, 3H), 2.93 (s, 4H), 1.66–1.83 (m, 5H), 1.42–1.55 (m, 1H), 1.10–1.35 (m, 4H), 0.84–1.05 (m, 2H). *m*/*z* calcd for C_21_H_31_N_3_O_3_S [M+H^+^] 405.6, found 406.2.

#### 6.5.82 Rac-(3*R*,3*aS*,9*bS*)-*N*-(cyclohexylmethyl)-1-methylsulfonyl-2,3,3*a*,4,5,9*b*-hexahydropyrrolo­[3,2-*c*]­quinoline-3-carboxamide (**82**)

Rac-(3*R*,3*aS*,9*bS*)-*N*-(cyclohexylmethyl)-1-methylsulfonyl-2,3,3*a*,4,5,9*b*-hexahydropyrrolo­[3,2-*c*]­quinoline-3-carboxamide (30 mg, 0.08 mmol, 1.0 equiv) was dissolved in CH_2_Cl_2_ (0.5 mL) and stirred at ambient temperature under argon. DIPEA (2 6.7 μL, 0.153 mmol) was added in one portion followed by dropwise addition of acetyl chloride (6.0 μ L, 0.09 mmol, 1.1 equiv) in CH_2_Cl_2_ (0.5 mL). The resultant solution was stirred at room temperature overnight. The mixture was concentrated *in* *vacuo.* Resuspension was performed in water and saturated with NaHCO_3_. The aqueous solution was washed with CH_2_Cl_2_ (2 × 10 mL). Combined organics were dried over Na_2_SO_4_, filtered, and concentrated *in* *vacuo.* The residue was then purified by flash column chromatography (0–2% MeOH in CH_2_Cl_2_) to give an off-white solid (23.2 mg, 70%). ^1^H NMR (400 MHz, CDCl_3_) δ 7.87–7.94 (m, 1H), 7.30–7.37 (m, 2H), 7.14–7.23 (m, 1H), 6.59–6.71 (m, 1 H), 5.05–5.15 (m, 1H), 4.71–4.86 (m, 1H), 3.96–4.11 (m, 1H), 3.55–3.66 (m, 1H), 3.33–3.45 (m, 1H), 3.18–3.29 (m, 1H), 3.06–3.16 (m, 1H), 2.99 (s, 4H), 2.63–2.74 (m, 1H), 2.37 (s, 3H), 1.61–1.79 (m, 5H), 1.47–1.59 (m, 1H), 1.13–1.36 (m, 3H), 0.92–1.10 (m, 2H), *m*/*z* calcd for C_22_H_31_N_3_O_4_S [M+H^+^] 433.2, found 434.2.

## Supplementary Material









## Notes

Molecular docking of compound 1 (enantiomer S,S,R) to model of GPR84 (PDB)
Molecular docking of compound 1 (enantiomer R,R,S) to model of GPR84 (PDB)
Experimental procedures and characterization data for compounds **84**–**86** and **91**–**96**, compound spectral data (^1^H NMR and LC-MS), HRMS, HPLC traces, and SFC purification methods (PDF)
Molecular formula strings (CSV)

## Abbreviations

DMPK: drug metabolism and pharmacokinetics
FFAR: free fatty acid receptor
GPCR: G-protein-coupled receptor
MCFA: medium-chain fatty acid
SAR: structure–activity relationship
