# Supplementary material for: Discovery, Structure–Activity Relationship, and Functional Characterization of a Chromenopyrrole Series as Orthosteric Antagonists of GPR84
Source: J Med Chem. 2026 Mar 23;69(7):7945–65. doi: 10.1021/acs.jmedchem.5c03367 (PMC13071874; doi:10.1021/acs.jmedchem.5c03367)
Supplement: Supplementary file 3 [file jm5c03367_si_003.pdf]

Supporting Information

Discovery, Structure–Activity Relationship and Functional Characterization of a Chromenopyrrole Series as Orthosteric antagonists of GPR84

Michael A. Malone,[a] Ruijing Yin,[b] Yueming Li,[b] Laura Jenkins,[b] Abdul-Akim Guseinov,[b] Sara Marsango,[b] Mark Huggett,[d] Margaret Huggett,[d] Anna Boyle,[d] Angus Morrison,[d] Irina G. Tikhonova,[c] Graeme Milligan*,[b] Andrew G. Jamieson[a]*

[a]*School of Chemistry, The Advanced Research Centre, University of Glasgow, 11 Chapel Lane, Glasgow G11 6EW, UK.*

[b]*Centre for Translational Pharmacology, The Advanced Research Centre, University of Glasgow, 11 Chapel Lane, G11 6EW, UK.*

[c]*School of Pharmacy, Queen's University Belfast, Belfast BT9 7BL, U.K*

[d]BioAscent Discovery Ltd, Bo'Ness Road, Newhouse, Lanarkshire, ML1 5UH, U.K.

Corresponding Authors

Graeme Milligan – Email: graeme.milligan@glasgow.ac.uk

Andrew G. Jamieson – Email: andrew.jamieson.2@glasgow.ac.uk

Contents

General Information S8

Synthesis S9

Compound Spectral Data S13

^{1}H Spectra for Compound 1 ....S13

LC-MS Spectra for Compound 1 ....S13

^{1}H Spectra for Compound 2 ....S14

LC-MS Spectra for Compound 2 ....S14

^{1}H NMR Spectra for Compound 3 ....S14

LC-MS Spectra for Compound 3 ....S14

^{1}H Spectra for Compound 4 ....S15

LC-MS Spectra for Compound 4 ....S15

^{1}H Spectra for Compound 5 ....S16

LC-MS Spectra for Compound 5 ....S16

^{1}H Spectra for Compound 6 ....S16

LC-MS Spectra for Compound 6 ....S17

^{1}H NMR Spectra for Compound 7 ....S17

LC-MS Spectra for Compound 7 ....S18

^{1}H NMR Spectra for Compound 8 ....S18

LC-MS Spectra for Compound 8 ....S19

^{1}H NMR Spectra for Compound 9 ....S19

LC-MS Spectra for Compound 9 ....S20

^{1}H NMR Spectra for Compound 10 ....S20

LC-MS Spectra for Compound 10 ....S20

^{1}H NMR Spectra for Compound 11 ....S21

LC-MS Spectra for Compound 11 ....S21

^{1}H NMR Spectra for Compound 12 ....S21

LC-MS Spectra for Compound 12 ....S22

^{1}H NMR Spectra for Compound 13 ....S22

LC-MS Spectra for Compound 13 ....S22

^{1}H NMR Spectra for Compound 14 ....S23

LC-MS Spectra for Compound 14 ....S23

^{1}H NMR Spectra for Compound 15 ....S23

LC-MS Spectra for Compound 15 ....S24

^{1}H NMR Spectra for Compound 16 ....S24

LC-MS Spectra for Compound 16 ....S25

^{1}H NMR Spectra for Compound 17 ....S25

LC-MS Spectra for Compound 17 ....S26

^{1}H NMR Spectra for Compound 18 ....S26

LC-MS Spectra for Compound 18 ....S27

^{1}H NMR Spectra for Compound 19 ....S27

LC-MS Spectra for Compound 19 ....S28
^{1}H NMR Spectra for Compound 20 ....S28
LC-MS Spectra for Compound 20 ....S29
^{1}H NMR Spectra for Compound 21 ....S29
LC-MS Spectra for Compound 21 ....S30
^{1}H NMR Spectra for Compound 22 ....S30
LC-MS Spectra for Compound 22 ....S30
^{1}H NMR Spectra for Compound 23 ....S31
LC-MS Spectra for Compound 23 ....S31
^{1}H NMR Spectra for Compound 24 ....S32
LC-MS Spectra for Compound 24 ....S32
^{1}H NMR Spectra for Compound 25 ....S32
LC-MS Spectra for Compound 25 ....S33
^{1}H NMR Spectra Compound 26 ....S33
LC-MS Spectra Compound 26 ....S34
^{1}H NMR Spectra for Compound 27 ....S35
LC-MS Spectra for Compound 27 ....S35
^{1}H NMR Spectra for Compound 28 ....S35
LC-MS Spectra for Compound 28 ....S35
^{1}H NMR Spectra for Compound 29 ....S36
LC-MS Spectra for Compound 29 ....S36
^{1}H NMR Spectra for Compound 30 ....S36
LC-MS Spectra for Compound 30 ....S37
^{1}H NMR Spectra for Compound 31 ....S37
LC-MS Spectra for Compound 31 ....S38
^{1}H NMR Spectra for Compound 32 ....S38
LC-MS Spectra for Compound 32 ....S39
^{1}H NMR Spectra for Compound 33 ....S39
LC-MS Spectra for Compound 33 ....S40
^{1}H NMR Spectra for Compound 34 ....S40
LC-MS Spectra for Compound 34 ....S41
^{1}H NMR Spectra for Compound 35 ....S41
LC-MS Spectra for Compound 35 ....S42
^{1}H NMR Spectra for Compound 36 ....S42
LC-MS Spectra for Compound 36 ....S43
^{1}H NMR Spectra for Compound 37....s43
LC-MS Spectra for Compound 37 ....S44
^{1}H NMR Spectra for Compound 38 ....S44
LC-MS Spectra for Compound 38 ....S45
^{1}H NMR Spectra for Compound 39 ....S45
LC-MS Spectra for Compound 39 ....S45

^{1}H NMR Spectra for Compound 40 .... S46
LC-MS Spectra for Compound 40 .... S46
^{1}H NMR Spectra for Compound 41 .... S46
LC-MS Spectra for Compound 41 .... S47
^{1}H NMR Spectra for Compound 42 .... S47
LC-MS Spectra for Compound 42 .... S47
^{1}H NMR Spectra for Compound 43 .... S48
LC-MS Spectra for Compound 43 .... S48
^{1}H NMR Spectra for Compound 44 .... S48
LC-MS Spectra for Compound 44 .... S49
^{1}H NMR Spectra for Compound 45 .... S49
LC-MS Spectra for Compound 45 .... S50
^{1}H NMR Spectra for Compound 46 .... S50
LC-MS Spectra for Compound 46 .... S51
^{1}H NMR Spectra for Compound 47 .... S51
LC-MS Spectra for Compound 47 .... S52
^{1}H NMR Spectra for Compound 48 .... S52
LC-MS Spectra for Compound 48 .... S53
^{1}H NMR Spectra for Compound 49 .... S53
LC-MS Spectra for Compound 49 .... S54
^{1}H NMR Spectra for Compound 50 .... S54
LC-MS Spectra for Compound 50 .... S55
^{1}H NMR Spectra for Compound 51 .... S55
LC-MS Spectra for Compound 51 .... S56
^{1}H NMR Spectra for Compound 52 .... S56
LC-MS Spectra for Compound 52 .... S57
^{1}H NMR Spectra for Compound 53 .... S57
LC-MS Spectra for Compound 53 .... S58
^{1}H NMR Spectra for Compound 54 .... S58
LC-MS Spectra for Compound 54 .... S59
^{1}H NMR Spectra for Compound 55 .... S59
LC-MS Spectra for Compound 55 .... S60
^{1}H NMR Spectra for Compound 56 .... S60
LC-MS Spectra for Compound 56 .... S61
^{1}H NMR Spectra for Compound 57 .... S61
LC-MS Spectra for Compound 57 .... S62
^{1}H NMR Spectra for Compound 58 .... S63
LC-MS Spectra for Compound 58 .... S63
^{1}H NMR Spectra for Compound 59 .... S63
LC-MS Spectra for Compound 59 .... S64
^{1}H NMR Spectra for Compound 60 .... S64

LC-MS Spectra for Compound 60 ....S64
^{1}H NMR Spectra for Compound 61 ....S65
LC-MS Spectra for Compound 61 ....S65
^{1}H NMR Spectra for Compound 62 ....S66
LC-MS Spectra for Compound 62 ....S66
^{1}H NMR Spectra for Compound 63 ....S66
LC-MS Spectra for Compound 63 ....S67
^{1}H NMR Spectra for Compound 64 ....S67
LC-MS Spectra for Compound 64 ....S68
^{1}H NMR Spectra for Compound 65 ....S68
LC-MS Spectra for Compound 65 ....S69
^{1}H NMR Spectra for Compound 66 ....S69
LC-MS Spectra for Compound 66 ....S70
^{1}H NMR Spectra for Compound 67 ....S70
LC-MS Spectra for Compound 67 ....S71
^{1}H NMR Spectra for Compound 68 ....S71
LC-MS Spectra for Compound 68 ....S72
^{1}H NMR Spectra for Compound 69 ....S72
LC-MS Spectra for Compound 69 ....S73
^{1}H NMR Spectra for Compound 70 ....S73
LC-MS Spectra for Compound 70 ....S74
^{1}H NMR Spectra for Compound 71 ....S74
LC-MS Spectra for Compound 71 ....S75
^{1}H NMR Spectra for Compound 72 ....S75
LC-MS Spectra for Compound 72 ....S76
^{1}H NMR Spectra for Compound 73 ....S76
LC-MS Spectra for Compound 73 ....S77
^{1}H NMR Spectra for Compound 74 ....S77
LC-MS Spectra for Compound 74 ....S78
^{1}H NMR Spectra for Compound 75 ....S78
LC-MS Spectra for Compound 75 ....S79
^{1}H NMR Spectra for Compound 76 ....S79
LC-MS Spectra for Compound 76 ....S80
^{1}H NMR Spectra for Compound 77 ....S80
LC-MS Spectra for Compound 77 ....S81
^{1}H NMR Spectra for Compound 78 ....S81
LC-MS Spectra for Compound 78 ....S82
^{1}H NMR Spectra for Compound 79 ....S82
LC-MS Spectra for Compound 79 ....S83
^{1}H NMR Spectra for Compound 80 ....S83
LC-MS Spectra for Compound 80 ....S84

^{1}H NMR Spectra for Compound 81 .... S85
LC-MS Spectra for Compound 81 .... S85
^{1}H NMR Spectra for Compound 82 .... S85
LC-MS Spectra for Compound 82 .... S86
^{13}C spectra for Compound 42 .... S87
^{13}C spectra for Compound 58 .... S87
^{13}C spectra for Compound 62 .... S87
^{13}C spectra for Compound 63 .... S88
^{13}C spectra for Compound 72 .... S88
HRMS of lead compounds from SAR investigations S90
HRMS Table S90
HRMS spectra for compound 42 .... S90
HRMS spectra for compound 58 .... S91
HRMS spectra for compound 62 .... S92
HRMS spectra for compound 63 .... S93
HRMS spectra for compound 72 .... S94
HPLC traces S96
HPLC trace for compound 42 .... S96
HPLC trace for compound 58 .... S96
HPLC trace for compound 62 .... S96
HPLC trace for compound 63 .... S96
HPLC trace for compound 72 .... S97
SFC purification S98
Chiral separation of compound 42 .... S98
Chiral separation of compound 58 .... S99
Chiral separation of compound 62 .... S100
Chiral separation of compound 63 .... S101
Chiral separation of compound 72 .... S102
Correlation plot S103
Mutagenesis S104
Supplementary figure SI-1 .... S104
Additional figures S105
Supplementary figure SI-2 .... S105

General Information

All starting materials were purchased commercially and used without further purification unless stated otherwise. All solvents were reagent grade and used as received. Anhydrous solvents were obtained by passage through a solvent filtration system (Pure Solv) and were transferred via a syringe. Thin-layer chromatography was performed on silica gel 60 F254 aluminium plates from Merck. Readings of the TLC plates was obtained via the use of a UV lamp (254 nm) or treatment with potassium permanganate. Column chromatography was undertaken using 230 - 400 mesh silica gel from SiliCycle. Proton and carbon nuclear magnetic resonance spectra (^{1}H and ^{13}C) were recorded on AVANCE III 400 Brucker 400 MHz and 101 MHz respectively. Chemical shifts are expressed in parts per million (ppm) and are referenced to the deuterated NMR solvent ^{1}H ($CDCl_3$, δ 7.26, CD_3OD, δ 3.31) and ^{13}C ($CDCl_3$, δ 77.0, CD_3OD, δ 49.0). The following abbreviations were used to describe signal splitting from the recorded ^{1}H NMR spectra: s = singlet, d = doublet, t = triplet, q = quartet, m = multiplet. Coupling constants (J-values) were measured in hertz (Hz). Accurate mass measurements were obtained via high resolution mass spectrometry (HRMS) performed on a Bruker microTOF-Q II using positive mode electrospray ionisation (ESI^+). Analyses by liquid chromatography – mass spectrometry (LC-MS) were performed on a Thermo Scientific LCQ Fleet Ion Trap Mass Spectrometer (ESI^+), where buffer A = 0.1% TFA in H_2O and buffer B = 0.1% TFA in MeCN. HPLC analysis was performed on a Shimadzu RP-HPLC system equipped with Shimadzu LC-20AT pumps, a Shimadzu SIL20A autosampler and a SPS-20A UV-vis detector using a Phenomenex Aeris column (5 mm C18, 100 Å, 150 x 10 mm) at a flow rate of 1 mL/min.

Synthesis

Methyl(E)-4-(2-formylphenoxy)but-2-enoate (84)

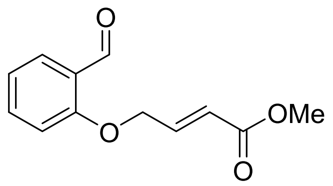

To a solution of salicylaldehyde (38.5 mmol, 1.0 equiv.) and K_2CO_3 (57.7 mmol, 1.5 equiv.) in acetone (100 mL) was added methyl (E)-4-bromobut-2-enoate (50.0 mmol, 1.3 equiv.). The resulting mixture was stirred at reflux for 3 hours. The crude material was concentrated under *vauco.* and a partitioning residue between water (200 mL) and washed with EtOAc (2 x 100 mL). The organic layer was dried over Na_2SO_4 and filtered under vacuo. Flash column chromatography (0 - 30% EtOAc in heptane) afforded the product (80%) as a white solid; ^{1}H NMR (400 MHz, $CDCl_3$) δ: 10.54 (s, 1H), 7.86 (dd, J = 8.0, 2.1 Hz, 1H), 7.54 (m, 1H), 6.93 (d, J = 8.0 Hz, 1H), 6.23 (dt, J = 16.0, 2.0 Hz, 1H), 4.83 (dd, 4.1, 2.1 Hz, 2H), 3.77 (s, 3H); m*/z calc.* for $C_{12}H_{14}O_4$ [M+H$^+$] 221.22 *found* 220.81.

Methyl-rac-(3R,3aR,9bS)-1-[(2,4-dimethoxyphenyl)methyl]-3,3a,4,9b-tetrahydro-2H-chromeno[4,3-b]pyrrole-3-carboxylate (85)

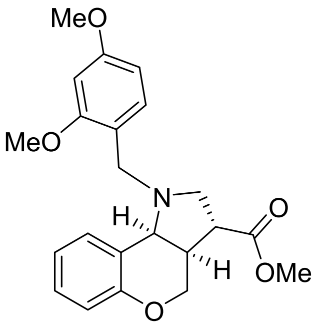

(±)

Methyl (E)-4-(2-formylphenoxy)but-2-enoate (13.6 mmol, 1.0 equiv.) and Ethyl-2-[(2,4-dimethoxyphenyl)methylamino]acetic acid (20.4 mmol, 1.5 equiv.) were suspended in toluene (100 mL). The mixture was heated at reflux for 6 hours and any water produced during the reaction was azeotroped using a Dean and Stark apparatus. The residue was cooled to room temperature and concentrated under *vauco.* The crude material was dissolved in EtOAc (100 mL) and washed with sat. NaHCO3 (2 x 50 mL). The aqueous layer was back extracted with EtOAc (50 mL) and the organic layers were combined. The combined organics were dried over Na_2SO_4, filtered and concentrated under *vacuo.* The residue was then purified by flash column chromatography (0-50% EtOAc in pet. ether) to a yellow oil (98 %): ^{1}H NMR (400 MHz, $CDCl_3$) δ: 7.29 (dd, J = 7.6, 2.5 Hz, 1H), 7.18 (m, 2H), 6.56 (m, 2H), 6.41 (m, 2H), 4.10 (m, 3H), 3.78 (s, 3H), 3.76 (s, 3H), 3.71 (s, 3H), 3.62 (m, 2H), 3.18 (m, 1H), 2.7 (m, 2H), 2.57 (m, 1H); m/z calcd for $C_{22}H_{26}NO_5$ [M+H$^+$] 384.18 found 384.18 (Δ = 2.67 ppm).

Methyl-rac-(3R,3aR,9bS)-1,2,3,3a,4,9b-hexahydrochromeno[4,3-b]pyrrole-3-carboxylate (86)

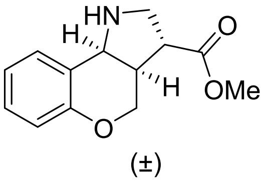

Methyl-rac-(3R,3aR,9bS)-1-[(2,4-dimethoxyphenyl)methyl]-3,3a,4,9b-tetrahydro-2H-chromeno[4,3-b]pyrrole-3-carboxylate (7.8 mmol, 1.0 equiv) was dissolved in TFA (12.0 mL) and subjected to microwave radiation at 100 °C for 80 minutes. The flask was placed in an ice bath and the mixture was diluted with water. Solid $NaHCO_3$ was added until pH 8. The aqueous solution was washed with EtOAc (3 x 50 mL). The combined organics were washed with brine (50 mL), dried over Na_2SO_4, filtered and concentrated down under *vacuo.* The residue was then purified by flash column chromatography (0 - 5% MeOH in CH_2Cl_2) to give a dark purple oil (66%); ^{1}H NMR (400 MHz, $CDCl_3$) δ: 7.35 (d, J = 7.4 Hz, 1H), 7.16 (m, 1H), 6.95 (m, 2H), 4.25 (d, J = 6.6 Hz, 1H), 4.18 (dd, J = 11.3, 3.8 Hz, 1H), 3.74 (s, 3H), 3.21 (m, 2H), 2.80 (m, 2H), 2.08 (s, 2H); *m/z calcd for* $C_{13}H_{16}NO_3$ [M+H$^+$] 234.27 *found* 233.95.

Synthesis of 2,2,2-trifluoro-N-(2-formylphenyl)acetamide (91)

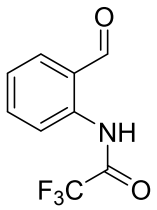

2-aminobenzaldehyde (16.5 mmol, 1 equiv.) was dissolved in CH_2Cl_2 and suspended in an ice bath under an argon atmosphere. Pyridine was then added (33.0 mmol, 2.0 equiv.). To the resulting solution was added TFAA (21.1 mmol, 1.3 equiv.) in a dropwise manner over 30 minutes. The ice bath was then removed and the reaction mixture was left to stir at r.t. for 4 hours. H_2O (50 mL) was added to the reaction mixture, extracting the aqueous layer with CH_2Cl_2 (2 x 50 mL). The organics were combined and washed with 1M HCl (70 mL) and then brine (70 mL), dried over Na_2SO_4, filtered and concentrated down under *vacuo.* The crude mixture was purified by flash column chromatography (0 – 20% EtOAc in heptane), affording an off white solid (1.66 g, 46%); ^{1}H NMR (400 MHz, $CDCl_3$) δ: 11.99 – 12.47 (m, 1H), 10.01 (s, 1H), 8.63 – 8.80 (m, 1H), 7.78 – 7.85 (m, 1H), 7.70 – 7.77 (m, 1H), 7.43 (d, J= 0.75 Hz, 1H).

Synthesis of methyl€-4-(2-formyl-N-(2,2,2-trifluoroacetyl)anilino)but-2-enoate (92)

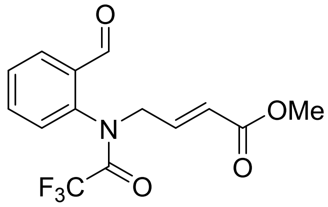

NaH (60%, 2.5 mmol, 1.1 equiv.) was suspended in DMF (4 mL) and stirred at r.t. under an argon atmosphere. A solution of 2,2,2-trifluoro-N-(2-formylphenyl)acetamide (2.3 mmol, 1.0 equiv.) in DMF (4 mL) was then added dropwise. The resulting reaction mixture was left to stir at r.t. for a further 30 mins. Over 20 minutes was then added a solution of methyl (E)-4-bromobut-2-enoate (618 mg, 3.5 mmol, 1.5 equiv.) in DMF (2 mL) dropwise. Upon completion a catalytic amount of 18-crown-6-ether in DMF was added and the corresponding reaction mixture was stirred at 60 °C for 4 hrs. The mixture was diluted in water (100 mL) and the aqueous solution was washed with EtOAc (2 x 50 mL). The

combined organics were washed with brine (50 mL), dried over Na_2SO_4, filtered and concentrated down under *vacuo.* The residue was then purified by flash column chromatography (0 - 40% EtOAc in Pet. ether) to give an orange gum (510 mg, 70%); ^{1}H NMR (400 MHz, $CDCl_3$) δ 10.09 (s, 1H), 7.92–8.03 (m, 1H), 7.71 (s, 2H), 7.29–7.37 (m, 1H), 6.83–6.98 (m, 1H), 5.85–5.95 (m, 1H), 4.96–5.07 (m, 1H), 3.87–3.97 (m, 1H), 7.60 (s, 3H).

Synthesis of rac-(3R,3aR,9bS)-1-[(2,4-dimethoxyphenyl)methyl]-3,3-a,4,9b-tetrahydro-2H-chromeno[4,3-b]pyrrole-3-carboxylate (93)

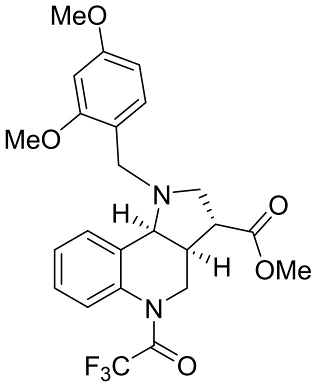

Methyl(E)-4-(2-formyl-N-(2,2,2-trifluoroacetyl)anilino)but-2-enoate (1.7 mmol, 1.0 equiv.) and 2-[(2,4-dimethoxyphenyl)methylamino]acetic acid (2.6 mmol, 1.5 equiv.) were suspended in toluene (15 mL). The mixture was heated at reflux for 6 hours and any water produced during the reaction was azeotroped using a Dean and Stark apparatus. The residue was cooled to room temperature and concentrated under vauco. The crude material was dissolved in EtOAc (10 mL) and washed with sat. NaHCO3 (2 x 5 mL). The aqueous layer was back extracted with EtOAc (10 mL) and the organic layers were combined. The combined organics were dried over Na_2SO_4, filtered and concentrated under vacuo. The residue was then purified by flash column chromatography (0-40% EtOAc in pet. ether) to give the product as a yellow oil (833 mg, 85%); ^{1}H NMR (400 MHz, $CDCl_3$) δ 10.09 (s, 1H), 7.92–8.03 (m, 1H), 7.71 (s, 2H), 7.60 (s, 3H), 7.29–7.37 (m, 1H), 6.83–6.98 (m, 1H), 5.85–5.95 (m, 1H), 4.96–5.07 (m, 1H), 3.87–3.97 (m, 1H), *m/z calcd for* $C_{24}H_{25}F_3N_2O_5$ [M+H$^+$] 478.5 *found* 479.2

Synthesis of methyl rac-(3R,3aS,9bS)-5-(2,2,2-trifluoroacetyl)-1,2,3,3a,4,9b-hexahydropyrrolo[3,2-c]quinoline-3-carboxylate (94)

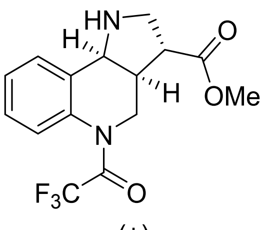

Methyl rac-(3R,3aR,9bS)-1-[(2,4-dimethoxyphenyl)methyl]-3,3a,4,9b-tetrahydro-2H-chromeno[4,3-b]pyrrole-3-carboxylate (1.3 mmol, 1.0 equiv.) was dissolved in TFA (1.96 mL) and subjected to microwave radiation at 100 °C for 80 minutes. The flask was placed in an ice bath and the mixture was diluted with water. Solid $NaHCO_3$ was added until pH 8. The aqueous solution was washed with EtOAc (3 x 25 mL). The organics were combined, dried over Na_2SO_4, filtered and concentrated down under *vacuo.* The residue was then purified by flash column chromatography (0-5%

MeOH in CH_2Cl_2) to give a dark purple oil (280 mg, 67%); ^{1}H NMR (400 MHz, $CDCl_3$) δ 7.60–7.83 (m, 1H), 7.54 (d, J = 8.8 Hz, 1H), 7.29–7.39 (m, 2H), 4.28–4.43 (m, 1H), 4.04–4.25 (m, 1H), 3.78 (s, 3H), 3.24–3.53 (m, 2H), 2.94–3.24 (m, 2H), 2.68–2.85 (m, 1H), 2.18–2.57 (m, 1H), *m/z calcd for* $C_{15}H_{15}F_3N_2O_3$ [M+H$^+$] 328.3 *found* 329.0

Synthesis of methyl rac-(3R,3aS,9bS)-1-methylsulfonyl-5-(2,2,2-trifluoroacetyl)-3,3a,4,9b-tetrahydro-2H-pyrrolo[3,2-c]quinoline-3-carboxylate (95)

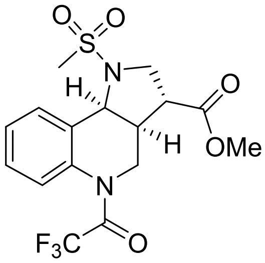

(±)

Methyl rac-(3R,3aS,9bS)-5-(2,2,2-trifluoroacetyl)-1,2,3,3a,4,9b-hexahydropyrrolo[3,2-c]quinoline-3-carboxylate (1.0 mmol, 1.0 equiv.) and DIPEA (16.0 mmol, 16 equiv.) were dissolved in dry CH_2Cl_2 (5.0 mL) under argon gas. The solution was cooled in an ice bath. Mesyl chloride (9.1 mmol, 9.0 equiv.) was added dropwise. The mixture was left stirring in the ice bath for 30 mins followed by 1 hour at room temp under argon gas. Was added CH_2Cl_2 (20.0 mL) to the reaction mixture and washed with $NaHCO_3$ (20 mL). The aqueous layer was back extracted with (10 mL) CH_2Cl_2. The organic layers were combined, dried over Na_2SO_4, filtered and concentrated down under *vacuo.* The residue was then purified by flash column chromatography (0-50% EtOAC in heptane) to give an off-white solid (322 mg, 76%); ^{1}H NMR (400 MHz, $CDCl_3$) δ 7.72–7.83 (m, 1H), 7.30–7.69 (m, 3H), 4.92–5.19 (m, 1H), 3.91–4.10 (m, 2H), 3.82–3.90 (m, 1H), 3.80 (s, 3H), 3.62–3.73 (m, 1H), 3.32–3.43 (m, 1H), 2.96–3.09 (m, 1H), 2.71–2.84 (m, 3H), *m/z calcd for* $C_{16}H_{17}F_3N_2O_5S$ [M+H$^+$] 406.4 *found* 407.4

Synthesis of rac-(3R,3aS,9bS)-1-methylsulfonyl-2,3,3a,4,5,9b-hexahydropyrrolo[3,2-c]quinoline-3-carboxylic acid (96)

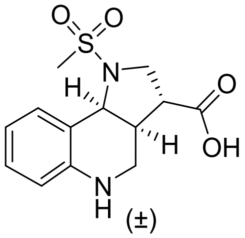

(±)

The relevant chromeno[4,3-b]pyrrole-3-carboxylate (0.3 mmol, 1.0 equiv.) was suspended in a solution of MeOH (1 mL) and 2M NaOH (0.9 mmol, 3 equiv.) and stirred at r.t. for 2 hours. The solution was neutralized with 5M HCl and concentrated down. Residue was partitioned between H_2O (5 mL) and EtOAc (3 x 5 mL). The organics were combined, dried over Na_2SO_4, filtered and concentrated down under *vacuo.* affording the crude product which was advanced to the next step.

Compound Spectral Data

[1]H Spectra for Compound 1

^{1}H NMR (400 MHz, CHLOROFORM-*d*) δ ppm 0.95 (s, 3 H) 1.61 (s, 4 H) 2.86 - 2.95 (m, 1 H) 3.05 (s, 3 H) 3.10 - 3.20 (m, 1 H) 3.43 (s, 2 H) 3.53 (s, 4 H) 3.56 - 3.63 (m, 1 H) 3.70 - 3.79 (m, 1 H) 4.15 - 4.28 (m, 2 H) 5.07 - 5.23 (m, 1 H) 6.00 - 6.23 (m, 1 H) 6.75 - 6.88 (m, 1 H) 6.96 - 7.08 (m, 1 H) 7.14 - 7.25 (m, 1 H) 7.69 - 7.79 (m, 1 H)

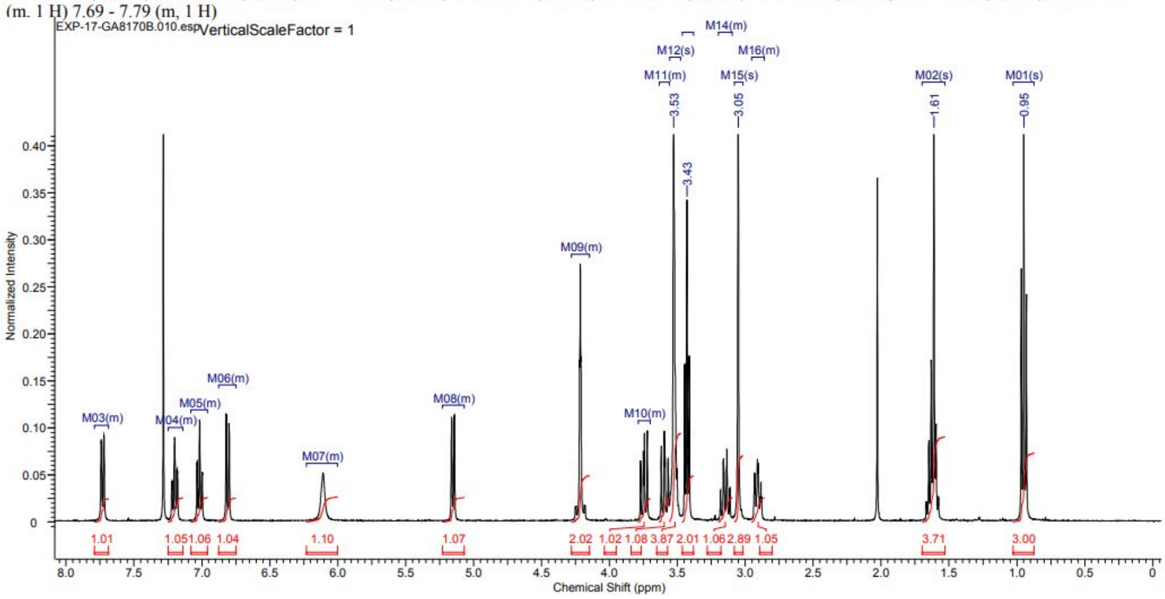

LC-MS Spectra for Compound 1

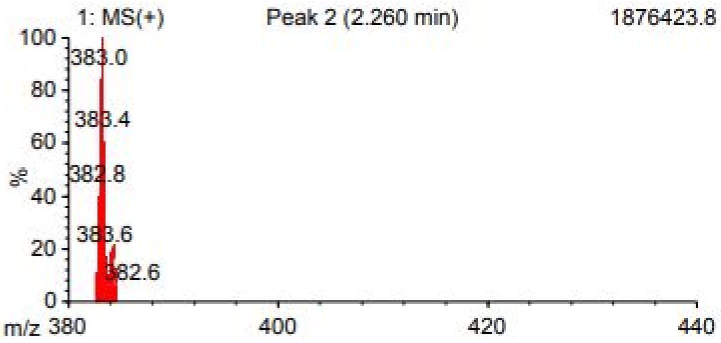

^{1}H Spectra for Compound 2

^{1}H NMR (400 MHz, DMSO-d_6) δ 8.18 - 8.33 (m, 1H), 7.50 - 7.62 (m, 1H), 7.10 - 7.23 (m, 1H), 6.91 - 7.05 (m, 1H), 6.75 - 6.85 (m, 1H), 5.08 - 5.20 (m, 1H), 4.01 - 4.23 (m, 2H), 3.36 - 3.50 (m, 2H), 3.10 (s, 6H), 2.76 - 2.87 (m, 1H), 0.97 - 1.08 (m, 5H), 0.67 - 0.80 (m, 1H), 0.40 (dd, J = 4.02, 8.53 Hz, 1H), 0.08 (t, J = 4.64 Hz, 1H)

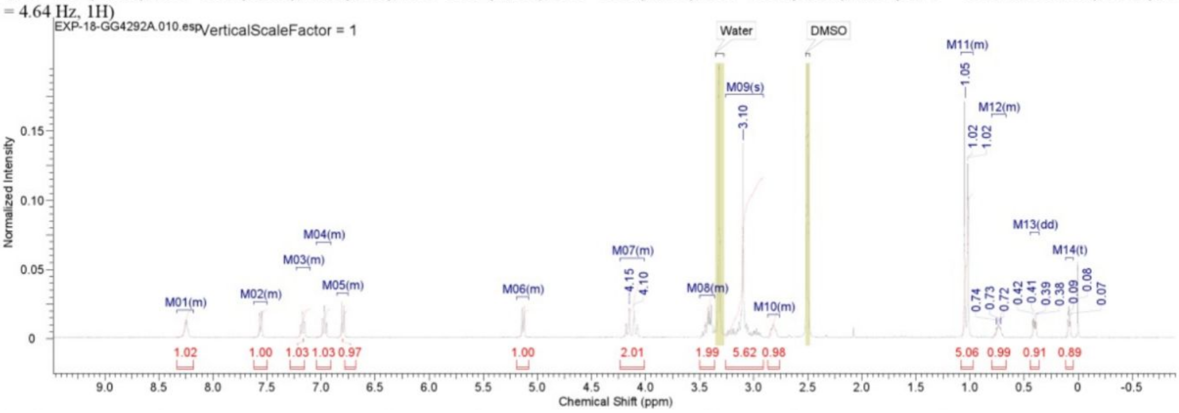

LC-MS Spectra for Compound 2

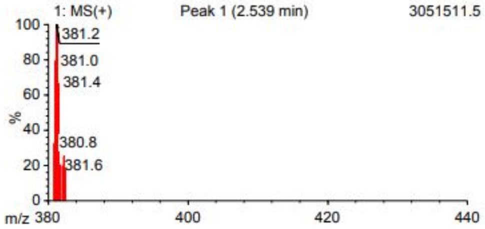

^{1}H NMR Spectra for Compound 3

^{1}H NMR (400 MHz, CHLOROFORM-d) δ ppm 1.85 (s, 2 H) 2.08 - 2.24 (m, 2 H) 2.84 - 2.95 (m, 1 H) 3.04 (s, 3 H) 3.08 - 3.19 (m, 1 H) 3.41 (d, J=6.78 Hz, 2 H) 3.51 - 3.61 (m, 1 H) 3.66 - 3.77 (m, 1 H) 4.11 - 4.27 (m, 2 H) 5.08 - 5.21 (m, 1 H) 5.79 - 6.04 (m, 1 H) 6.72 - 6.86 (m, 1 H) 6.95 - 7.08 (m, 1 H) 7.14 - 7.24 (m, 1 H) 7.63 - 7.78 (m, 1 H)

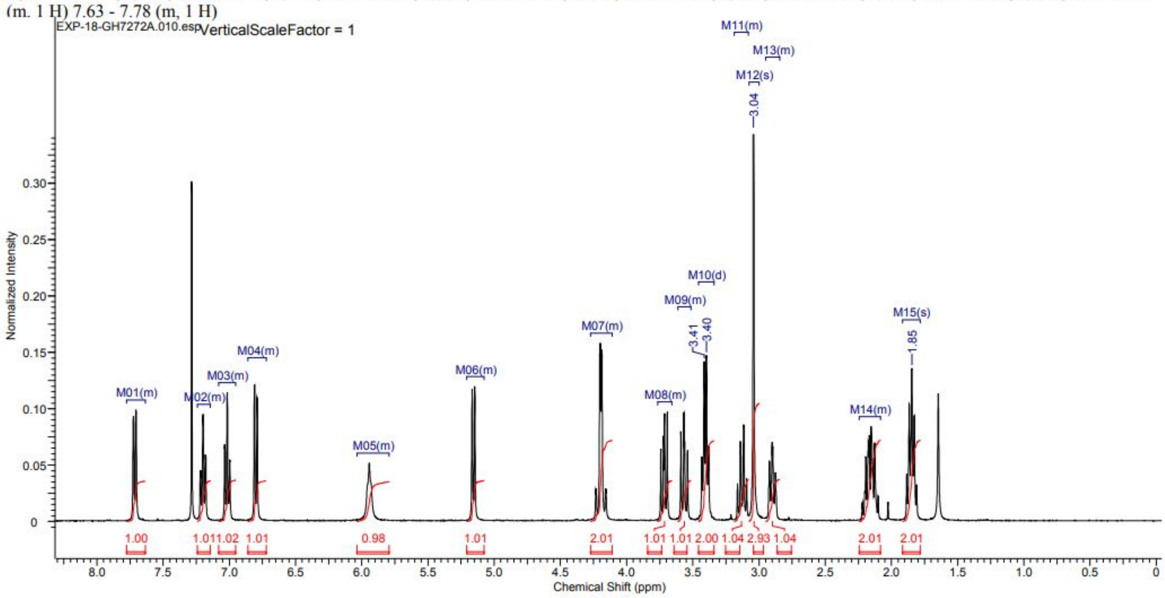

LC-MS Spectra for Compound 3

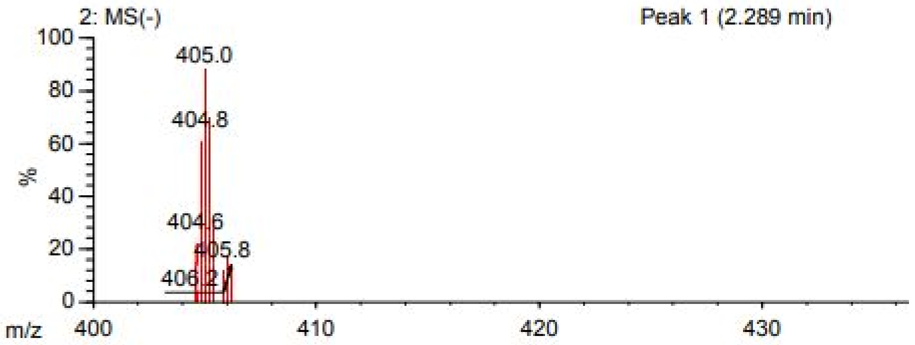

[1]H Spectra for Compound 4

^{1}H NMR (400 MHz, DMSO-d_6) δ 8.10 - 8.26 (m, 1H), 7.50 - 7.62 (m, 1H), 7.12 - 7.25 (m, 1H), 6.91 - 7.04 (m, 1H), 6.72 - 6.85 (m, 1H), 5.06 - 5.19 (m, 1H), 3.99 - 4.21 (m, 2H), 3.35 - 3.48 (m, 2H), 3.10 (s, 5H), 2.76 - 2.86 (m, 1H), 1.50 - 1.62 (m, 1H), 1.26 - 1.36 (m, 2H), 0.87 (d, J = 6.78 Hz, 4H)

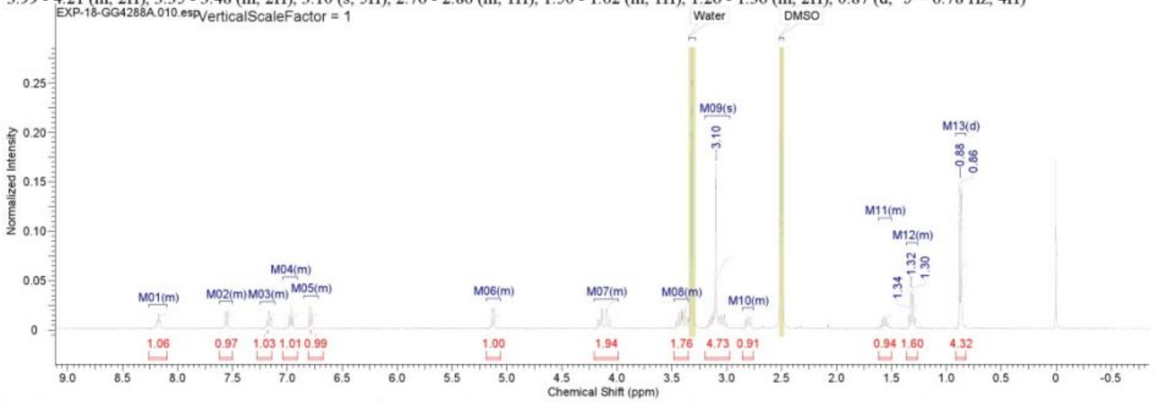

LC-MS Spectra for Compound 4

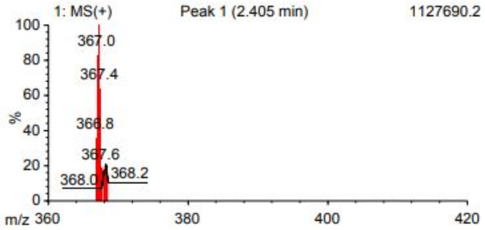

^{1}H Spectra for Compound 5

^{1}H NMR (400 MHz, CHLOROFORM-*d*) δ ppm 0.95 (d, *J*=6.78 Hz, 6 H) 1.74 - 1.93 (m, 1 H) 2.85 - 2.96 (m, 1 H) 3.05 (s, 3 H) 3.16 (s, 3 H) 3.52 - 3.63 (m, 1 H) 3.67 - 3.78 (m, 1 H) 4.21 (d, *J*=1.76 Hz, 2 H) 5.08 - 5.20 (m, 1 H) 5.74 - 5.89 (m, 1 H) 6.74 - 6.85 (m, 1 H) 6.96 - 7.07 (m, 1 H) 7.14 - 7.25 (m, 1 H) 7.68 - 7.78 (m, 1 H)

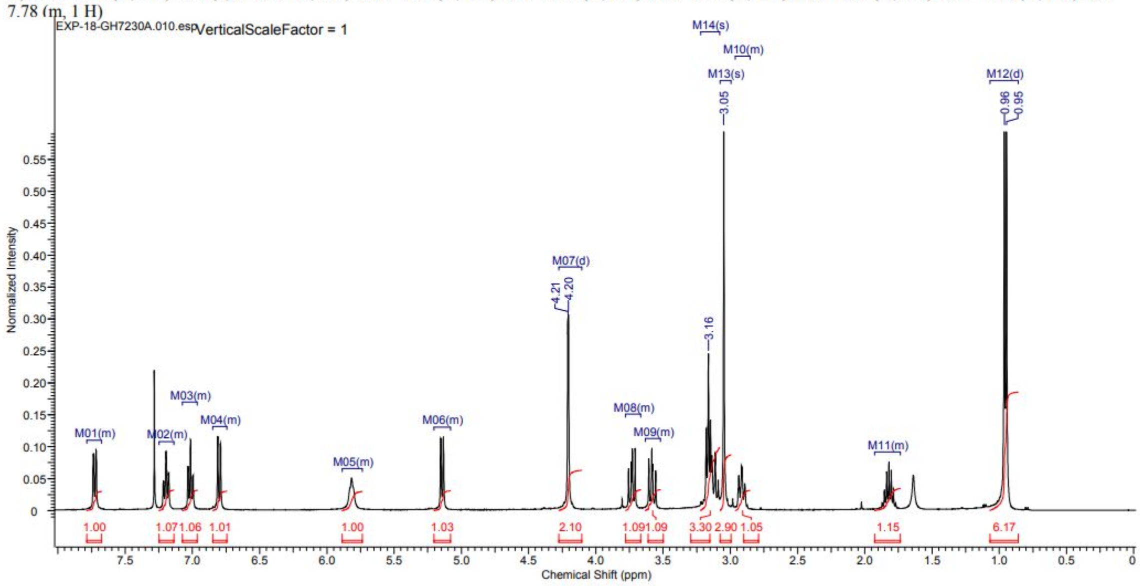

LC-MS Spectra for Compound 5

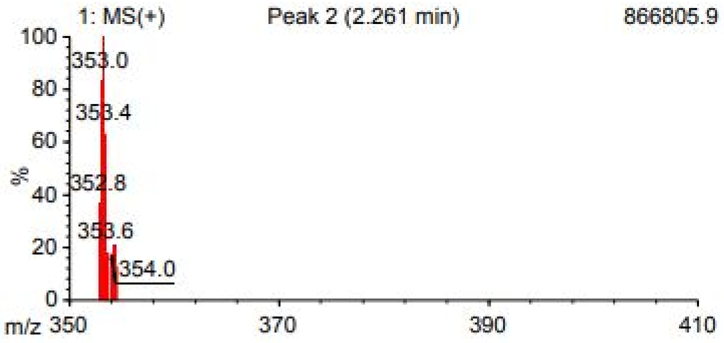

^{1}H Spectra for Compound 6

¹H NMR (400 MHz, CHLOROFORM-*d*) δ ppm 0.95 (s, 9 H) 2.86 - 2.99 (m, 1 H) 3.05 (s, 3 H) 3.15 (dd, *J*=6.53, 1.51 Hz, 3 H) 3.51 - 3.66 (m, 1 H) 3.69 - 3.80 (m, 1 H) 4.22 (d, *J*=1.76 Hz, 2 H) 5.10 - 5.20 (m, 1 H) 5.71 - 5.85 (m, 1 H) 6.74 - 6.88 (m, 1 H) 6.97 - 7.08 (m, 1 H) 7.16 - 7.24 (m, 1 H) 7.69 - 7.78 (m, 1 H)

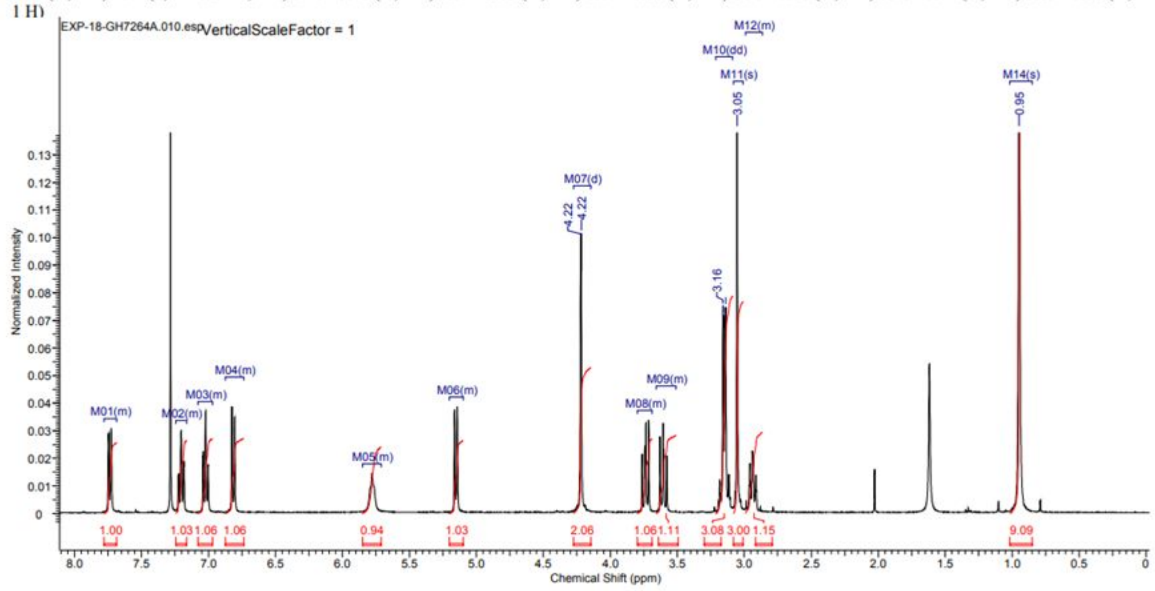

LC-MS Spectra for Compound 6

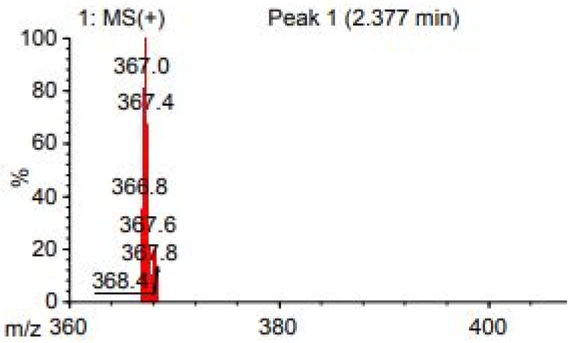

¹H NMR Spectra for Compound 7

^{1}H NMR (400 MHz, CHLOROFORM-*d*) δ ppm 0.93 (t, *J*=7.40 Hz, 6 H) 1.34 (s, 5 H) 2.85 - 2.98 (m, 1 H) 3.05 (s, 3 H) 3.08 - 3.17 (m, 1 H) 3.29 (s, 2 H) 3.54 - 3.64 (m, 1 H) 3.67 - 3.78 (m, 1 H) 4.15 - 4.26 (m, 2 H) 5.09 - 5.19 (m, 1 H) 5.62 - 5.79 (m, 1 H) 6.75 - 6.86 (m, 1 H) 6.95 - 7.07 (m, 1 H) 7.14 - 7.25 (m, 1 H) 7.68 - 7.79 (m, 1 H)

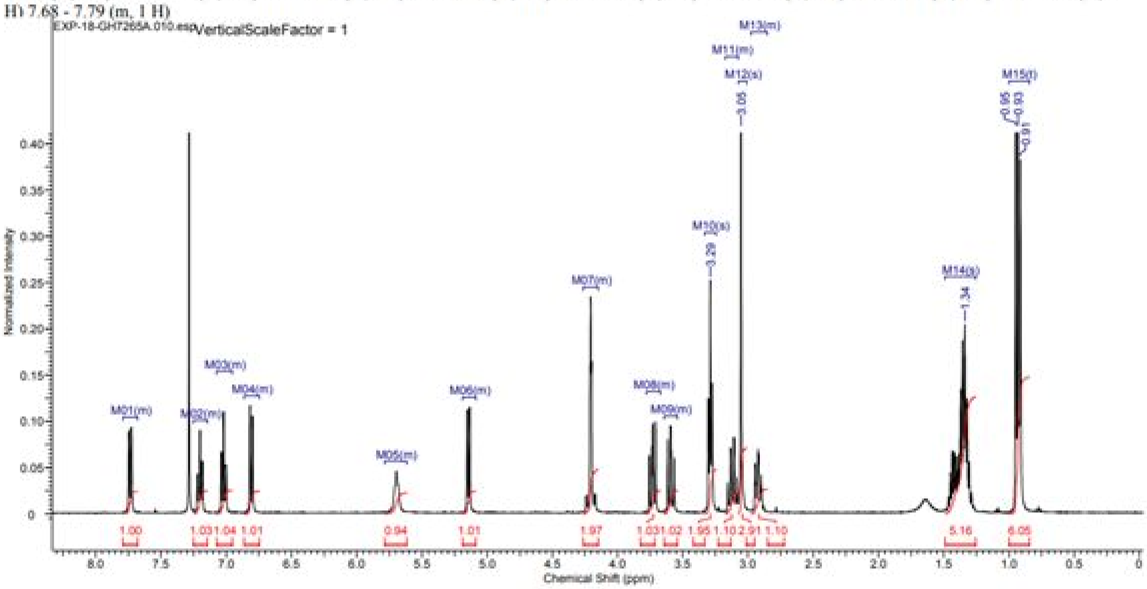

LC-MS Spectra for Compound 7

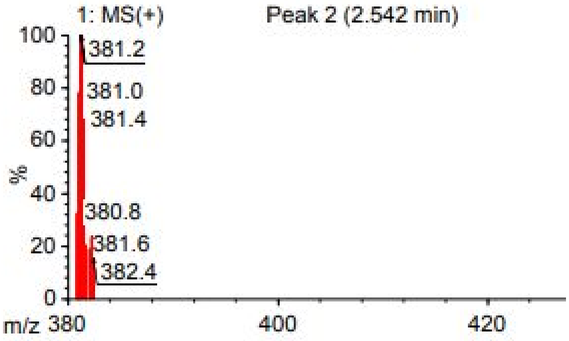

^{1}H NMR Spectra for Compound 8

^{1}H NMR (400 MHz, CHLOROFORM-*d*) δ ppm 0.18 - 0.27 (m, 2 H) 0.49 - 0.60 (m, 2 H) 0.91 - 1.04 (m, 1 H) 2.84 - 2.95 (m, 1 H) 3.04 (s, 3 H) 3.07 - 3.23 (m, 3 H) 3.51 - 3.63 (m, 1 H) 3.68 - 3.78 (m, 1 H) 4.15 - 4.26 (m, 2 H) 5.08 - 5.17 (m, 1 H) 5.78 - 5.92 (m, 1 H) 6.76 - 6.84 (m, 1 H) 6.96 - 7.06 (m, 1 H) 7.14 - 7.23 (m, 1 H) 7.68 - 7.77 (m, 1 H)

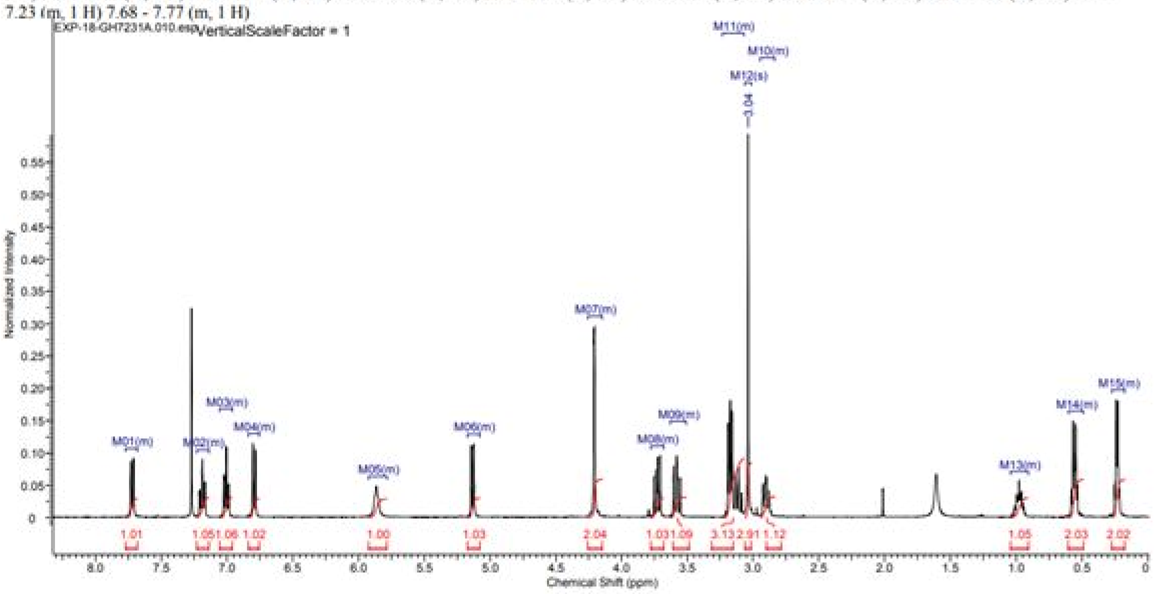

LC-MS Spectra for Compound 8

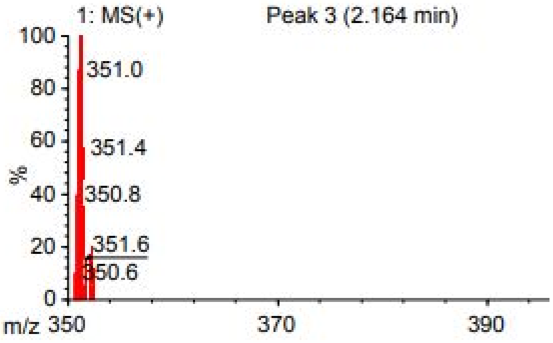

^{1}H NMR Spectra for Compound 9

^{1}H NMR (400 MHz, DMSO-d_6) δ 8.18 - 8.33 (m, 1H), 7.50 - 7.62 (m, 1H), 7.10 - 7.23 (m, 1H), 6.91 - 7.05 (m, 1H), 6.75 - 6.85 (m, 1H), 5.08 - 5.20 (m, 1H), 4.01 - 4.23 (m, 2H), 3.36 - 3.50 (m, 2H), 3.10 (s, 6H), 2.76 - 2.87 (m, 1H), 0.97 - 1.08 (m, 5H), 0.67 - 0.80 (m, 1H), 0.40 (dd, J = 4.02, 8.53 Hz, 1H), 0.08 (t, J = 4.64 Hz, 1H)

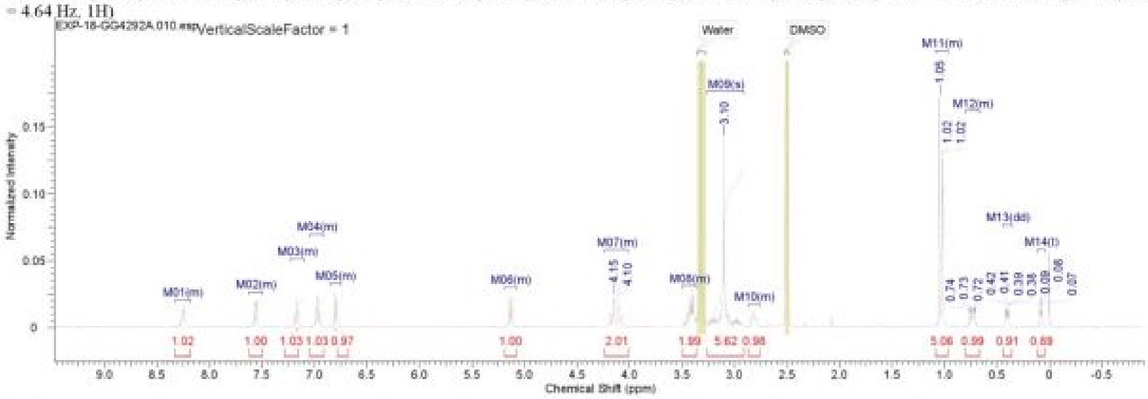

LC-MS Spectra for Compound 9

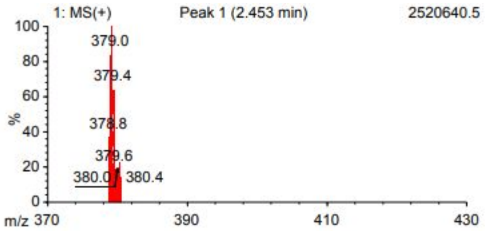

^{1}H NMR Spectra for Compound 10

^{1}H NMR (400 MHz, CHLOROFORM-d) δ ppm 1.24 - 1.39 (m, 1 H) 1.67 - 1.77 (m, 1 H) 1.86 - 2.07 (m, 1 H) 2.86 - 2.97 (m, 1 H) 3.06 (d, J=1.51 Hz, 5 H) 3.53 - 3.68 (m, 1 H) 3.71 - 3.83 (m, 1 H) 3.87 - 4.02 (m, 1 H) 4.22 (d, J=2.01 Hz, 2 H) 5.18 (d, J=7.78 Hz, 1 H) 6.07 - 6.23 (m, 1 H) 6.76 - 6.87 (m, 1 H) 7.02 (s, 1 H) 7.15 - 7.25 (m, 1 H) 7.64 - 7.79 (m, 1 H)

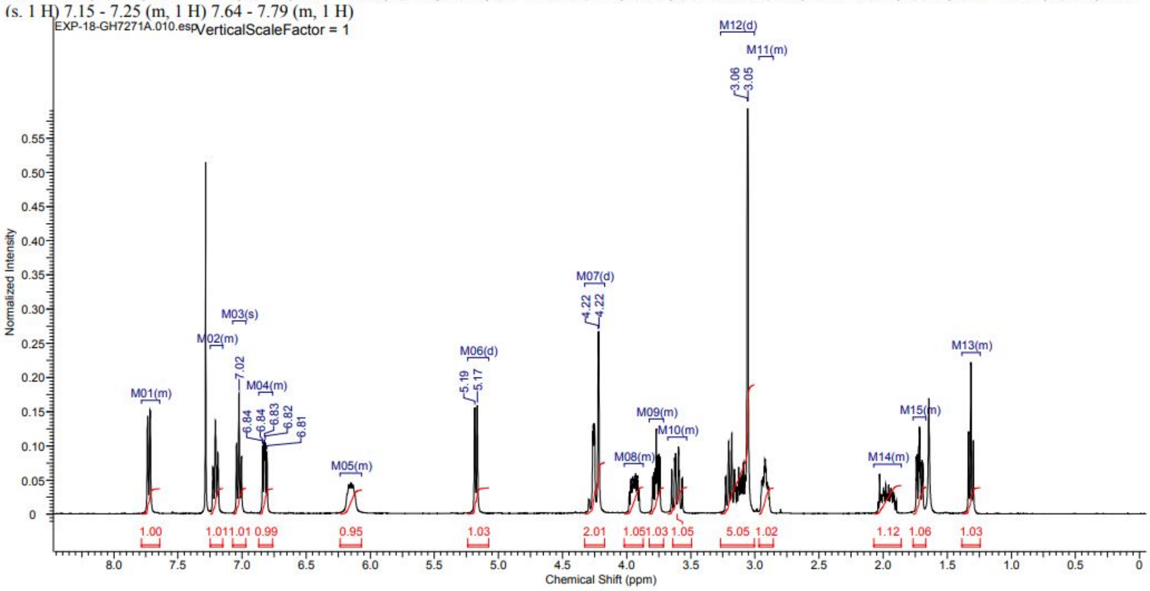

LC-MS Spectra for Compound 10

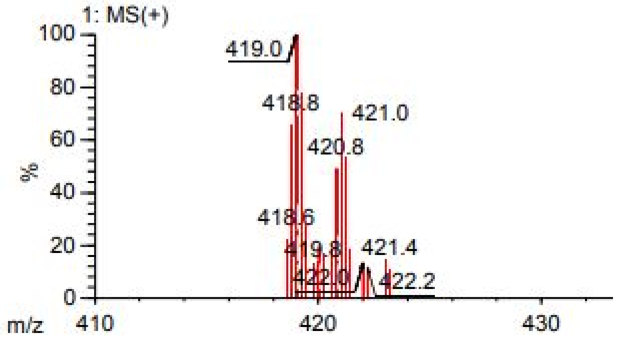

^{1}H NMR Spectra for Compound 11

^{1}H NMR (400 MHz, DMSO-d_6) δ 8.11 - 8.26 (m, 1H), 7.50 - 7.63 (m, 1H), 7.12 - 7.26 (m, 1H), 6.93 - 7.06 (m, 1H), 6.71 - 6.87 (m, 1H), 5.06 - 5.21 (m, 1H), 4.00 - 4.22 (m, 2H), 3.34 - 3.47 (m, 2H), 3.12 - 3.21 (m, 1H), 3.09 (s, 4H), 2.76 - 2.85 (m, 1H), 2.27 - 2.39 (m, 1H), 1.68 - 1.78 (m, 2H), 1.46 (t, J = 9.91 Hz, 2H), 1.10 (s, 3H), 1.02 (s, 3H)

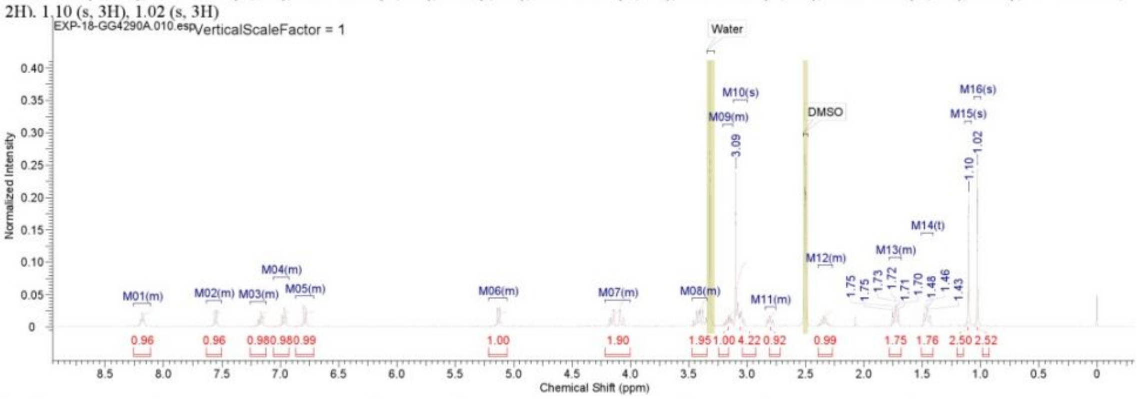

LC-MS Spectra for Compound 11

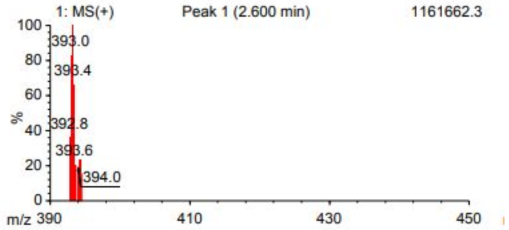

^{1}H NMR Spectra for Compound 12

^{1}H NMR (400 MHz, DMSO-d_6) δ 8.30 - 8.47 (m, 1H), 7.49 - 7.61 (m, 1H), 7.11 - 7.23 (m, 1H), 6.92 - 7.04 (m, 1H), 6.73 - 6.84 (m, 1H), 5.07 - 5.22 (m, 1H), 4.03 - 4.24 (m, 2H), 3.36 - 3.51 (m, 2H), 3.15 - 3.28 (m, 2H), 3.10 (s, 3H), 3.00 - 3.08 (m, 1H), 2.76 - 2.85 (m, 1H), 2.60 (ddd, J = 5.77, 8.53, 14.05 Hz, 2H), 2.22 - 2.36 (m, 3H)

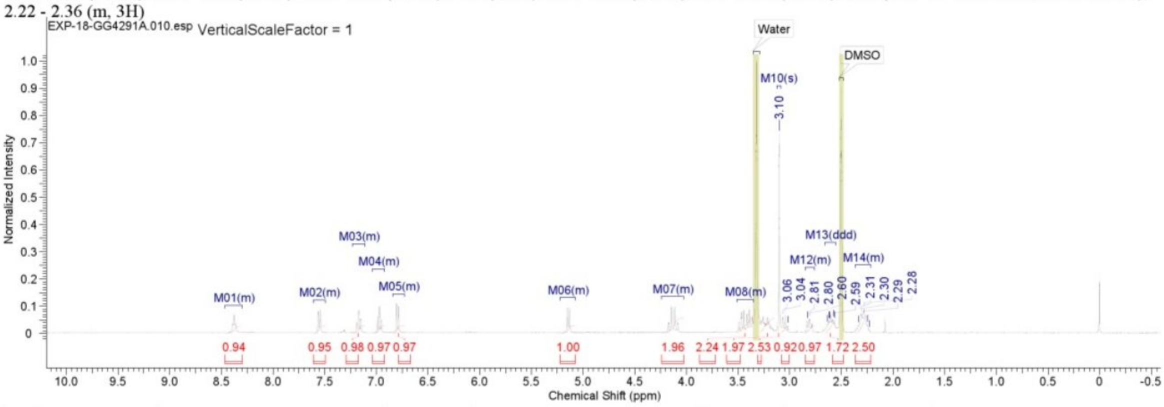

LC-MS Spectra for Compound 12

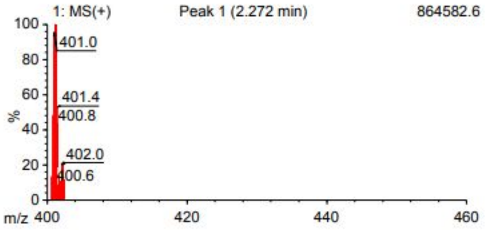

¹H NMR Spectra for Compound 13

¹H NMR (400 MHz, CHLOROFORM-*d*) δ ppm 1.12 - 1.30 (m, 2 H) 1.61 (s, 5 H) 1.72 - 1.84 (m, 2 H) 1.99 - 2.15 (m, 1 H) 2.86 - 2.95 (m, 1 H) 3.05 (s, 3 H) 3.07 - 3.17 (m, 1 H) 3.21 - 3.33 (m, 2 H) 3.53 - 3.65 (m, 1 H) 3.68 - 3.79 (m, 1 H) 4.21 (d, *J*=1.51 Hz, 2 H) 5.08 - 5.18 (m, 1 H) 5.72 - 5.88 (m, 1 H) 6.74 - 6.86 (m, 1 H) 6.96 - 7.06 (m, 1 H) 7.14 - 7.24 (m, 1 H) 7.67 - 7.77 (m, 1 H)

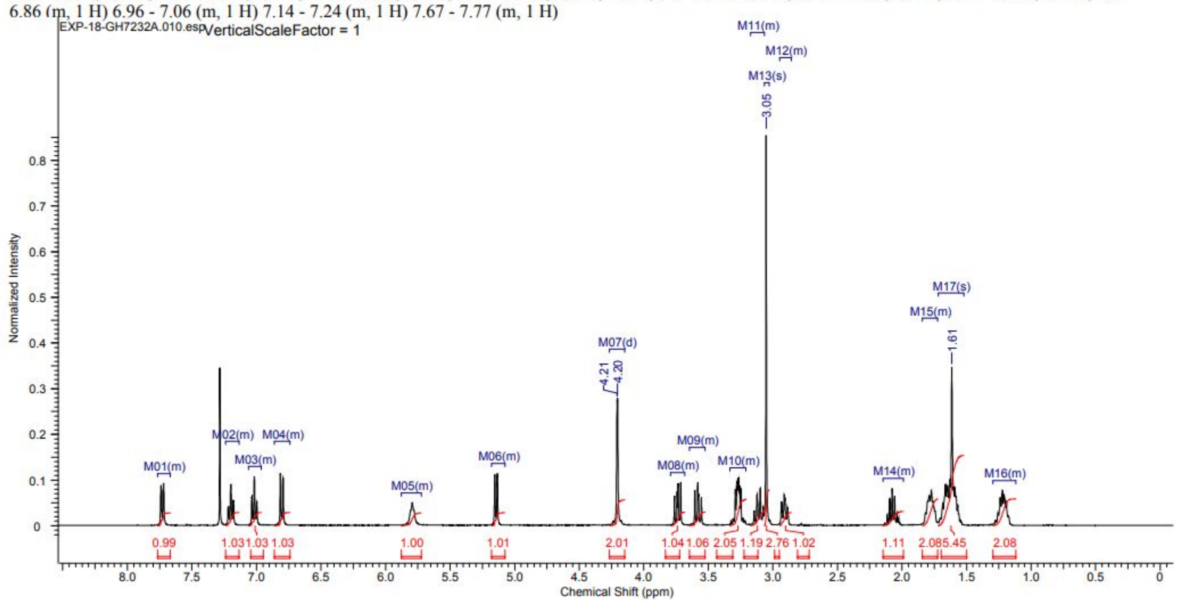

LC-MS Spectra for Compound 13

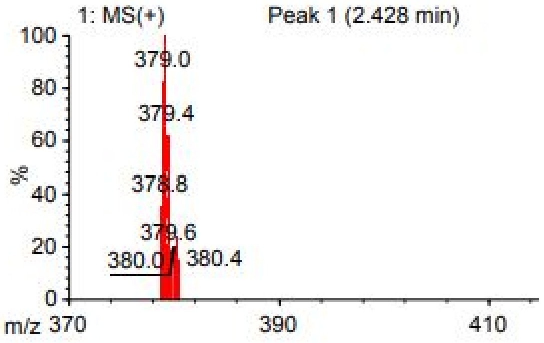

^{1}H NMR Spectra for Compound 14

^{1}H NMR (400 MHz, CHLOROFORM-*d*) δ ppm 0.83 - 1.03 (m, 2 H) 1.09 - 1.37 (m, 3 H) 1.38 - 1.54 (m, 1 H) 1.62 - 1.88 (m, 6 H) 2.31 - 2.41 (m, 1 H) 2.44 - 2.54 (m, 2 H) 2.57 - 2.70 (m, 1 H) 2.73 - 2.83 (m, 1 H) 2.84 - 2.92 (m, 1 H) 2.94 (s, 3 H) 3.29 - 3.43 (m, 1 H) 3.46 - 3.61 (m, 1 H) 4.11 - 4.22 (m, 1 H) 4.26 - 4.39 (m, 1 H) 5.10 - 5.21 (m, 1 H) 6.72 - 6.87 (m, 1 H) 6.92 - 7.05 (m, 1 H) 7.13 - 7.25 (m, 1 H) 7.65 - 7.77 (m, 1 H)

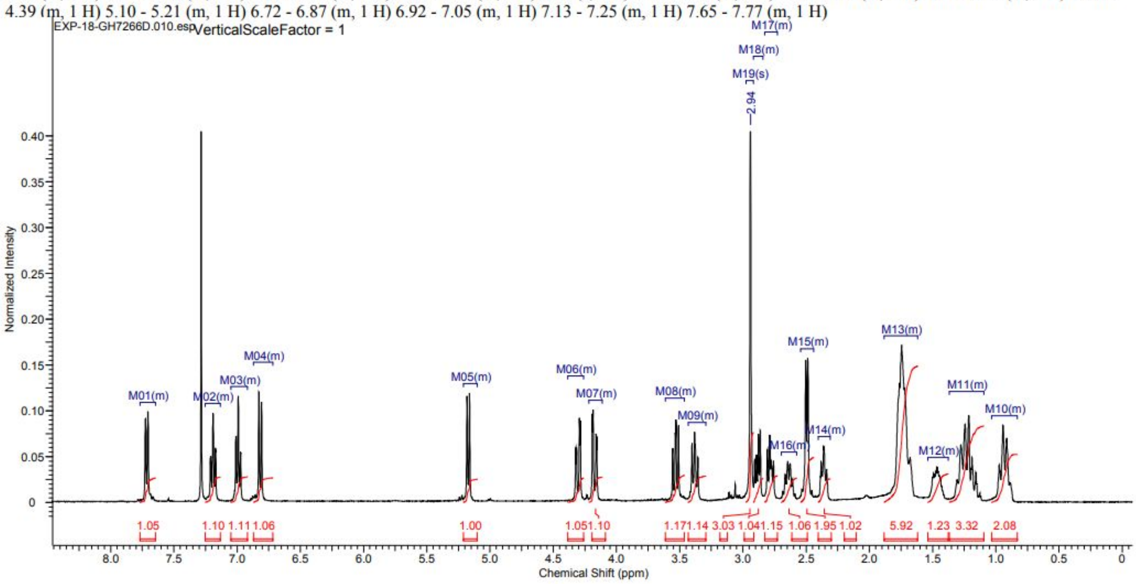

LC-MS Spectra for Compound 14

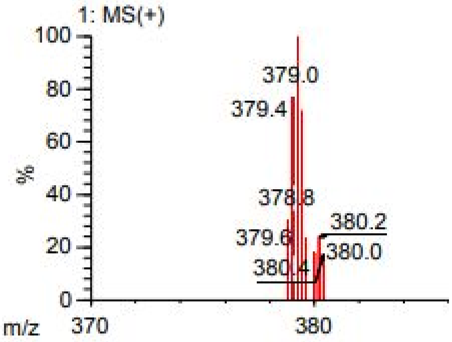

^{1}H NMR Spectra for Compound 15

1H NMR (400 MHz, CHLOROFORM-*d*) δ ppm 1.13 - 1.30 (m, 2 H) 1.35 - 1.79 (m, 13 H) 2.87 - 2.95 (m, 1 H) 3.05 (s, 4 H) 3.16 - 3.26 (m, 2 H) 3.51 - 3.64 (m, 1 H) 3.69 - 3.82 (m, 1 H) 4.21 (s, 2 H) 5.09 - 5.19 (m, 1 H) 5.71 - 5.84 (m, 1 H) 6.75 - 6.86 (m, 1 H) 6.94 - 7.07 (m, 1 H) 7.15 - 7.24 (m, 1 H) 7.69 - 7.76 (m, 1 H)

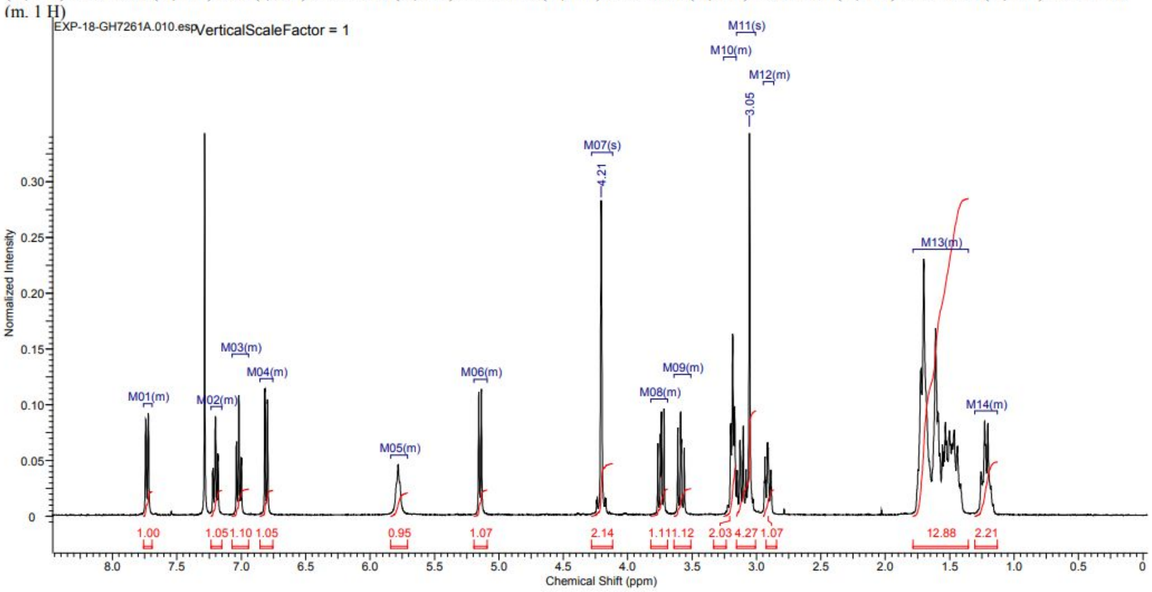

LC-MS Spectra for Compound 15

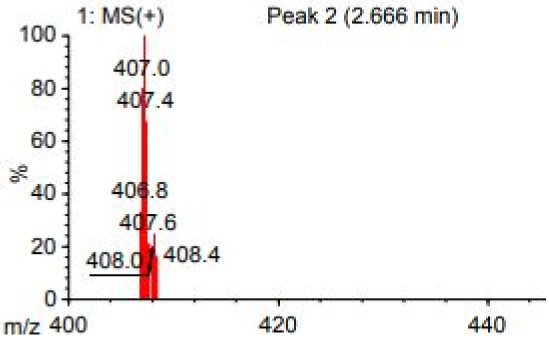

^{1}H NMR Spectra for Compound 16

1H NMR (400 MHz, CHLOROFORM-*d*) δ ppm 1.24 - 1.38 (m, 2 H) 1.40 - 1.80 (m, 14 H) 2.87 - 2.96 (m, 1 H) 3.05 (s, 3 H) 3.07 - 3.23 (m, 3 H) 3.51 - 3.64 (m, 1 H) 3.67 - 3.80 (m, 1 H) 4.21 (s, 2 H) 5.10 - 5.19 (m, 1 H) 5.72 - 5.84 (m, 1 H) 6.76 - 6.86 (m, 1 H) 6.98 - 7.07 (m, 1 H) 7.16 - 7.25 (m, 1 H) 7.69 - 7.78 (m, 1 H)

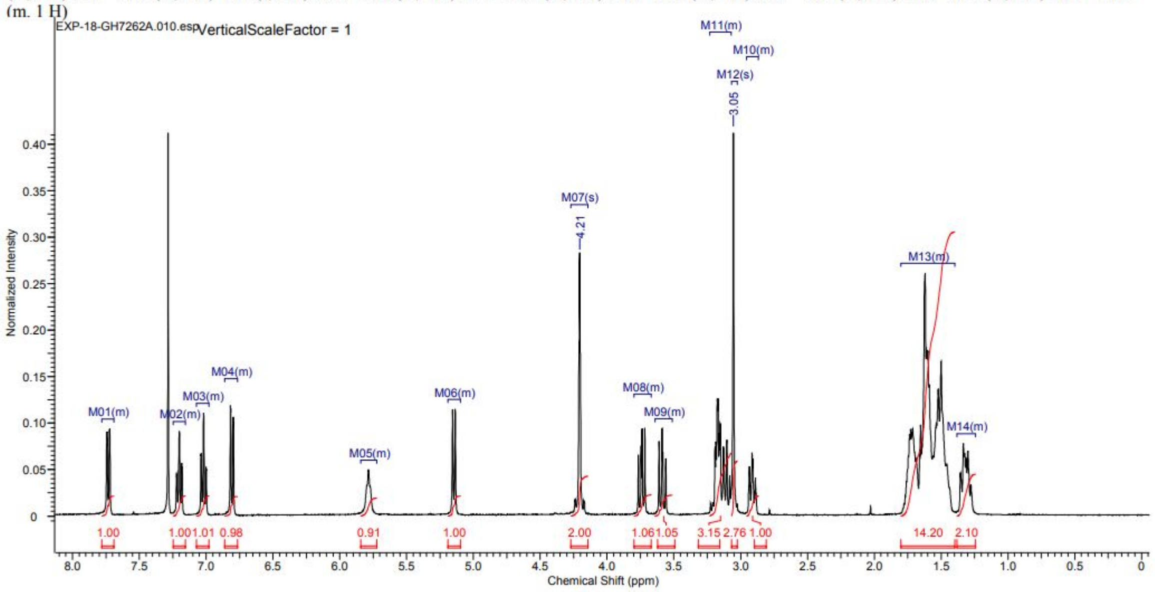

LC-MS Spectra for Compound 16

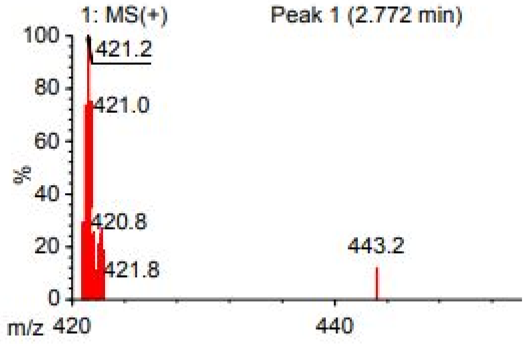

1H NMR Spectra for Compound 17

^{1}H NMR (400 MHz, CHLOROFORM-*d*) δ ppm 1.50 (br. s., 6 H) 1.60 - 1.81 (m, 7 H) 1.97 - 2.08 (m, 3 H) 2.88 - 2.97 (m, 1 H) 3.00 - 3.09 (m, 5 H) 3.10 - 3.21 (m, 1 H) 3.55 - 3.65 (m, 1 H) 3.68 - 3.81 (m, 1 H) 4.23 (d, *J*=1.51 Hz, 2 H) 5.08 - 5.20 (m, 1 H) 5.68 - 5.85 (m, 1 H) 6.82 (s, 1 H) 6.98 - 7.06 (m, 1 H) 7.16 - 7.24 (m, 1 H) 7.67 - 7.78 (m, 1 H)

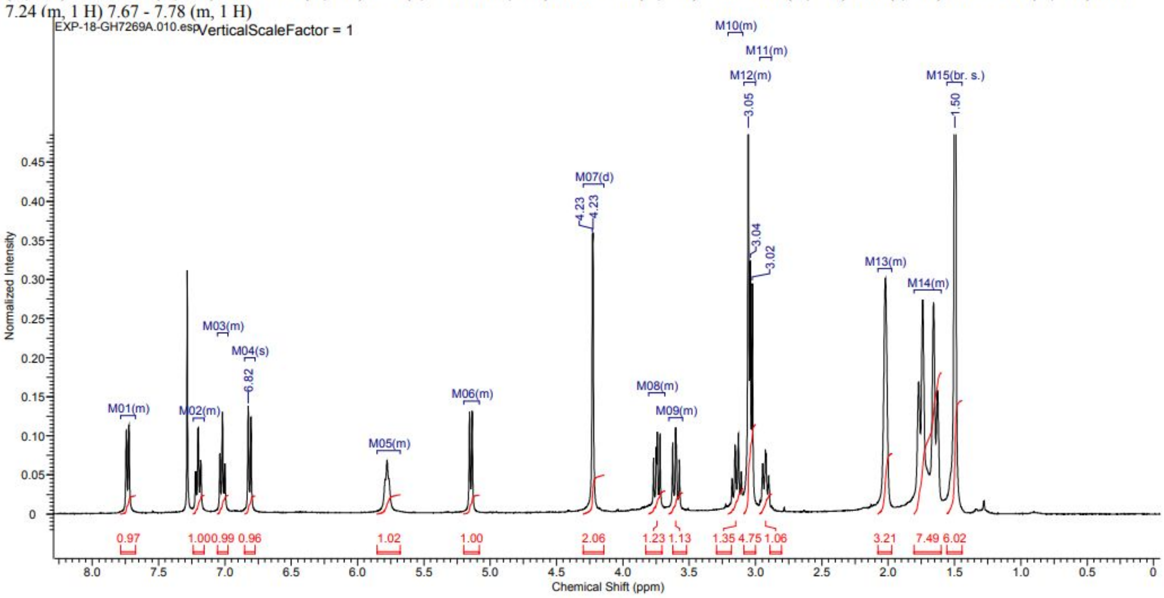

LC-MS Spectra for Compound 17

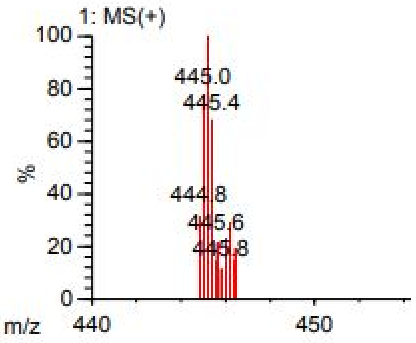

^{1}H NMR Spectra for Compound 18

1H NMR (400 MHz, CHLOROFORM-*d*) δ ppm 1.05 - 1.27 (m, 3 H) 1.28 - 1.49 (m, 2 H) 1.54 - 1.83 (m, 4 H) 1.87 - 2.01 (m, 2 H) 2.82 - 2.93 (m, 1 H) 3.05 (s, 4 H) 3.56 (dd, *J*=11.29, 9.03 Hz, 1 H) 3.73 (dd, *J*=11.29, 8.28 Hz, 2 H) 4.20 (s, 2 H) 5.07 - 5.19 (m, 1 H) 5.56 - 5.73 (m, 1 H) 6.77 - 6.84 (m, 1 H) 6.95 - 7.05 (m, 1 H) 7.11 - 7.24 (m, 1 H) 7.66 - 7.77 (m, 1 H)

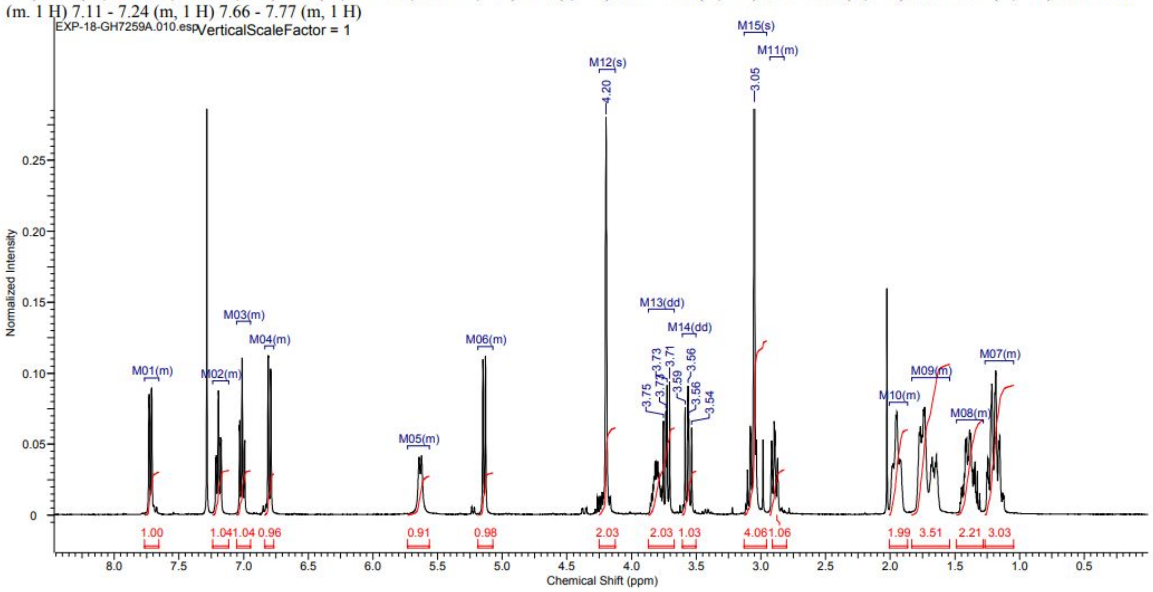

LC-MS Spectra for Compound 18

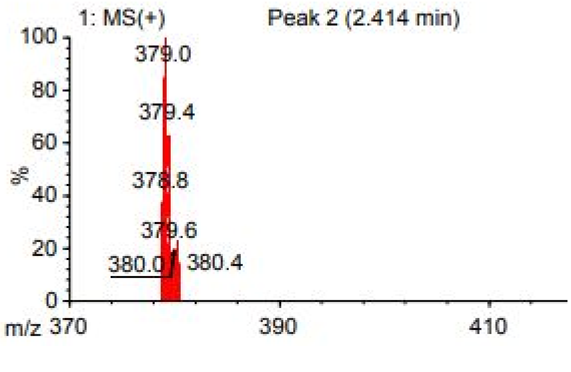

1H NMR Spectra for Compound 19

¹H NMR (400 MHz, CHLOROFORM-*d*) δ ppm 0.84 - 1.02 (m, 2 H) 1.07 - 1.34 (m, 4 H) 1.37 - 1.48 (m, 2 H) 1.57 - 1.77 (m, 6 H) 2.84 - 2.93 (m, 1 H) 3.04 (s, 4 H) 3.25 - 3.40 (m, 2 H) 3.49 - 3.62 (m, 1 H) 3.66 - 3.79 (m, 1 H) 4.18 (s, 2 H) 5.07 - 5.18 (m, 1 H) 5.63 - 5.77 (m, 1 H) 6.74 - 6.83 (m, 1 H) 6.95 - 7.05 (m, 1 H) 7.14 - 7.22 (m, 1 H) 7.65 - 7.76 (m, 1 H)

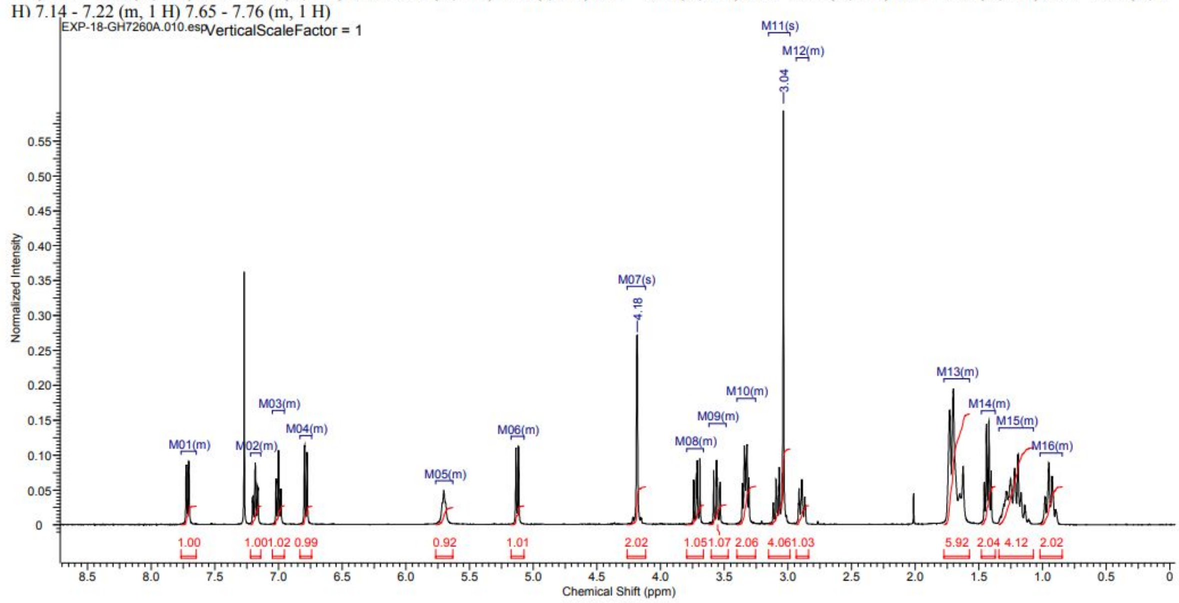

LC-MS Spectra for Compound 19

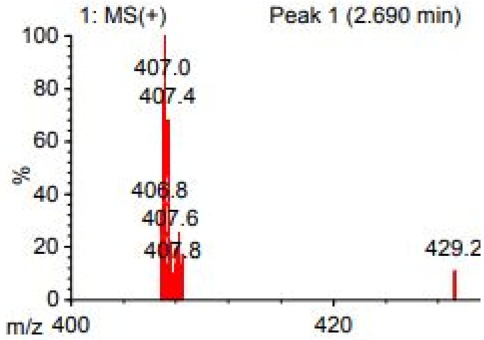

¹H NMR Spectra for Compound 20

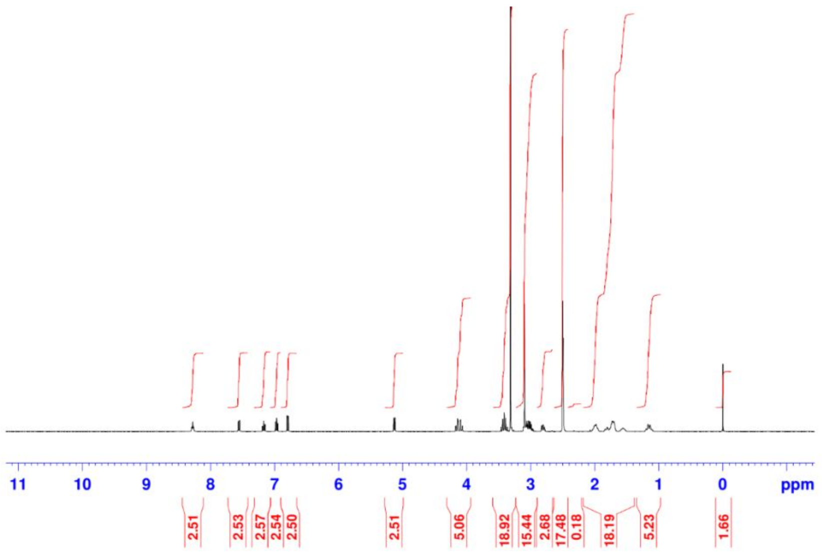

LC-MS Spectra for Compound 20

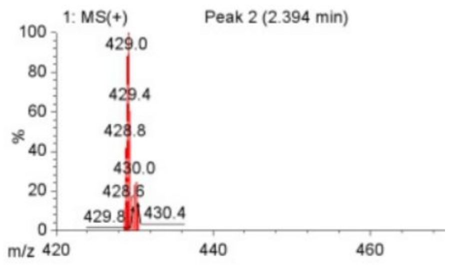

^{1}H NMR Spectra for Compound 21

^{1}H NMR (400 MHz, CHLOROFORM-*d*) δ ppm 0.83 - 1.06 (m, 5 H) 1.22 - 1.57 (m, 5 H) 1.62 - 1.79 (m, 3 H) 2.85 - 2.97 (m, 1 H) 3.05 (s, 3 H) 3.07 - 3.16 (m, 1 H) 3.16 - 3.21 (m, 1 H) 3.22 - 3.33 (m, 1 H) 3.52 - 3.65 (m, 1 H) 3.67 - 3.80 (m, 1 H) 4.21 (s, 2 H) 5.06 - 5.21 (m, 1 H) 5.66 - 5.84 (m, 1 H) 6.73 - 6.86 (m, 1 H) 6.95 - 7.07 (m, 1 H) 7.14 - 7.24 (m, 1 H) 7.68 - 7.79 (m, 1 H)

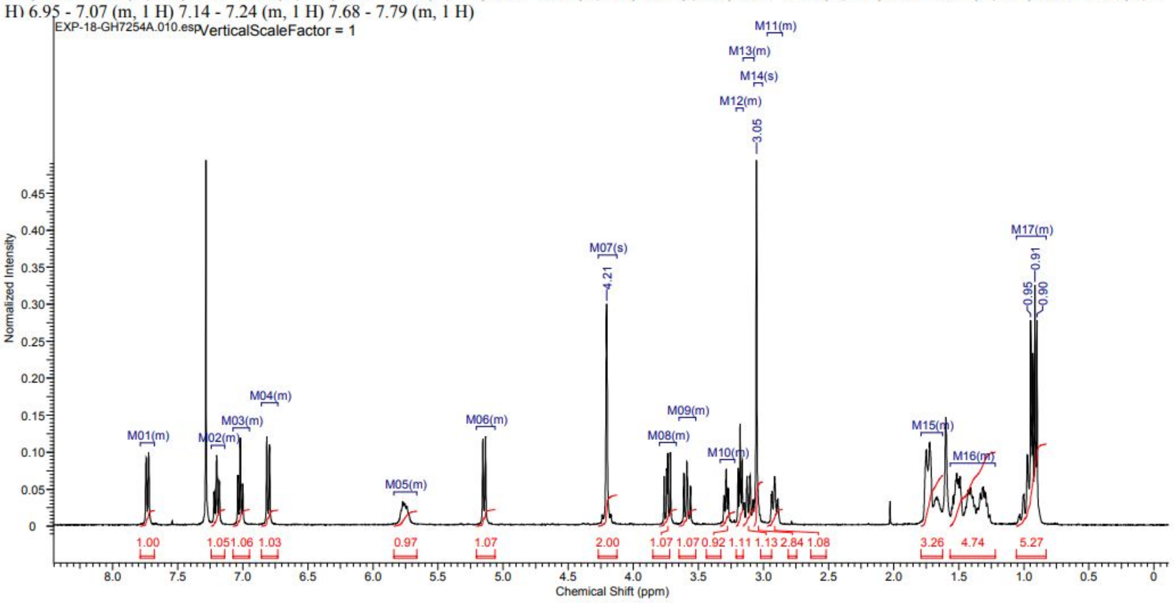

LC-MS Spectra for Compound 21

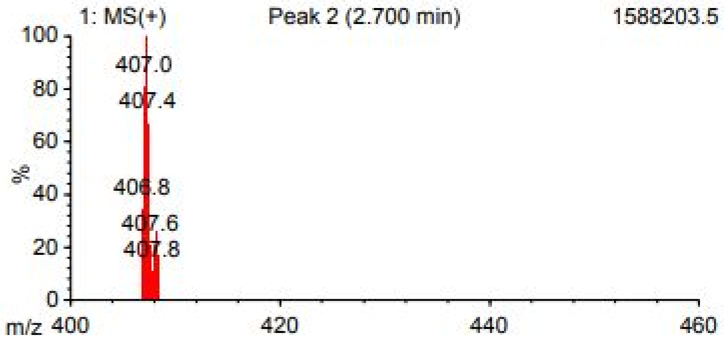

^{1}H NMR Spectra for Compound 22

^{1}H NMR (400 MHz, CHLOROFORM-*d*) δ ppm 1.29 - 1.45 (m, 2 H) 1.47 - 1.60 (m, 1 H) 1.65 - 1.75 (m, 2 H) 1.93 - 2.04 (m, 2 H) 2.31 (s, 3 H) 2.84 - 2.96 (m, 3 H) 3.04 (s, 3 H) 3.08 - 3.31 (m, 3 H) 3.52 - 3.63 (m, 1 H) 3.65 - 3.77 (m, 1 H) 4.13 - 4.25 (m, 2 H) 5.11 - 5.19 (m, 1 H) 5.91 - 6.07 (m, 1 H) 6.73 - 6.85 (m, 1 H) 6.96 - 7.06 (m, 1 H) 7.14 - 7.23 (m, 1 H) 7.65 - 7.77 (m, 1 H)

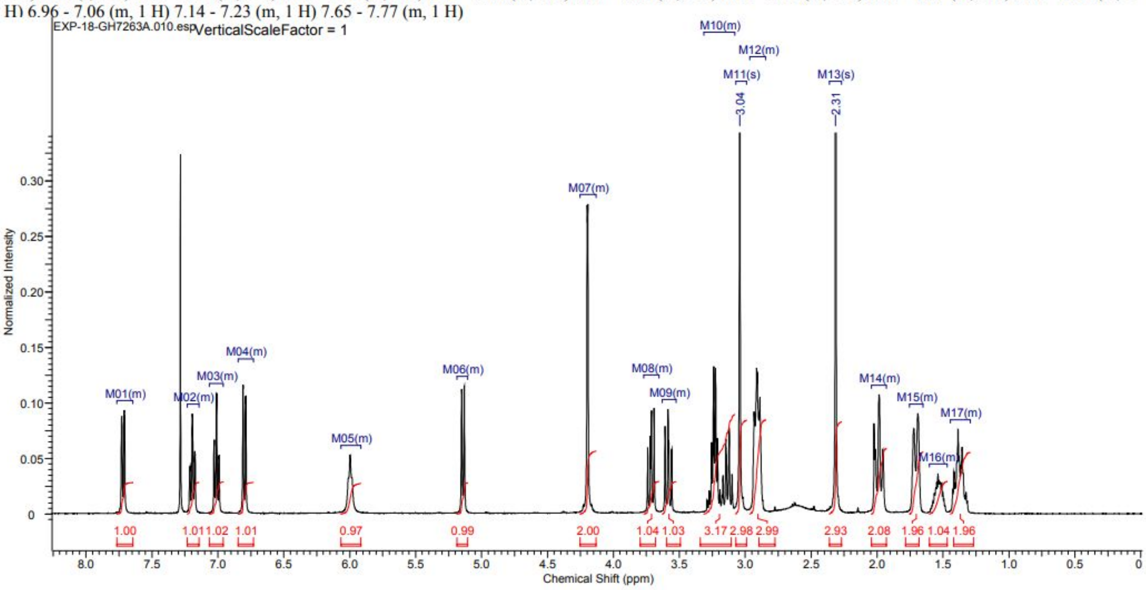

LC-MS Spectra for Compound 22

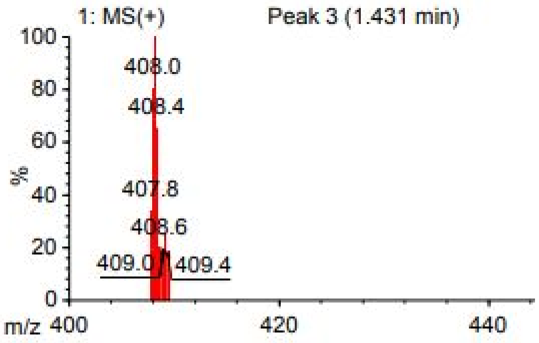

^{1}H NMR Spectra for Compound 23

^{1}H NMR (400 MHz, CHLOROFORM-*d*) δ ppm 1.36 (d, *J*=4.52 Hz, 2 H) 1.54 - 1.68 (m, 4 H) 1.70 - 1.87 (m, 1 H) 2.86 - 2.96 (m, 1 H) 3.05 (s, 3 H) 3.08 - 3.18 (m, 1 H) 3.24 (s, 2 H) 3.39 (d, *J*=1.25 Hz, 2 H) 3.53 - 3.64 (m, 1 H) 3.66 - 3.79 (m, 1 H) 3.93 - 4.07 (m, 2 H) 4.20 (dd, *J*=4.27, 1.76 Hz, 2 H) 5.15 (d, *J*=7.78 Hz, 1 H) 5.77 - 5.92 (m, 1 H) 6.76 - 6.84 (m, 1 H) 6.98 - 7.06 (m, 1 H) 7.15 - 7.24 (m, 1 H) 7.64 - 7.78 (m, 1 H)

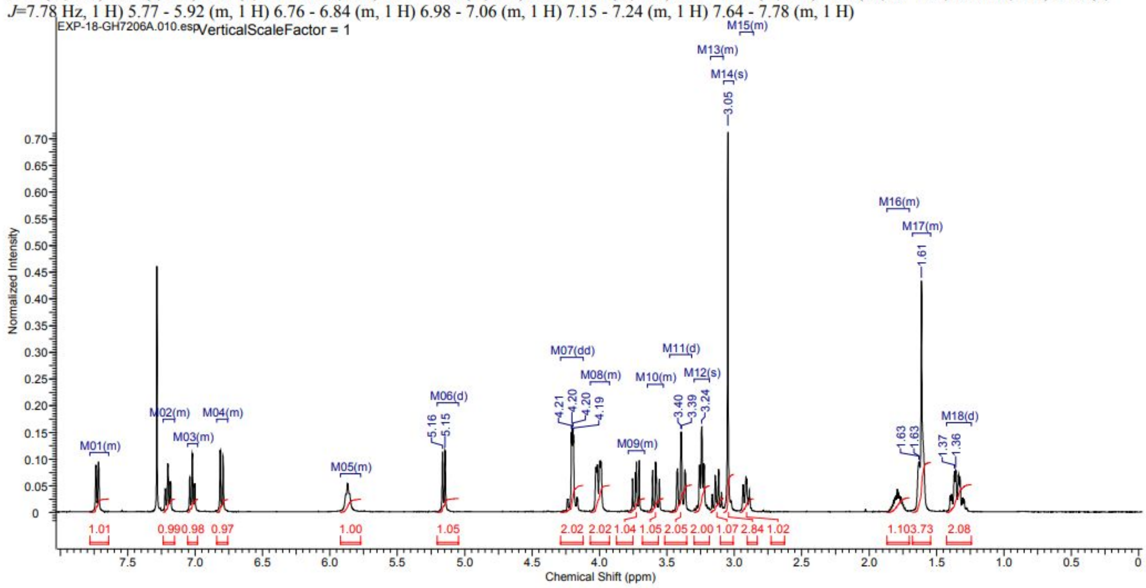

LC-MS Spectra for Compound 23

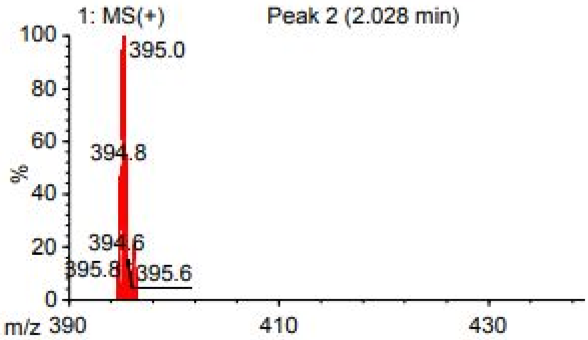

^{1}H NMR Spectra for Compound 24

^{1}H NMR (400 MHz, CHLOROFORM-*d*) δ ppm 0.94 - 1.09 (m, 1 H) 1.24 - 1.41 (m, 1 H) 1.60 (d, *J*=4.77 Hz, 4 H) 1.65 - 1.78 (m, 2 H) 1.78 - 1.93 (m, 1 H) 1.95 - 2.06 (m, 1 H) 2.07 - 2.21 (m, 1 H) 2.85 - 2.96 (m, 1 H) 3.05 (s, 3 H) 3.08 - 3.17 (m, 1 H) 3.18 - 3.24 (m, 1 H) 3.31 - 3.39 (m, 1 H) 3.54 - 3.63 (m, 1 H) 3.67 - 3.78 (m, 1 H) 4.13 - 4.28 (m, 2 H) 5.10 - 5.23 (m, 1 H) 5.73 - 5.92 (m, 1 H) 6.73 - 6.86 (m, 1 H) 6.96 - 7.08 (m, 1 H) 7.13 - 7.24 (m, 1 H) 7.66 - 7.79 (m, 1 H)

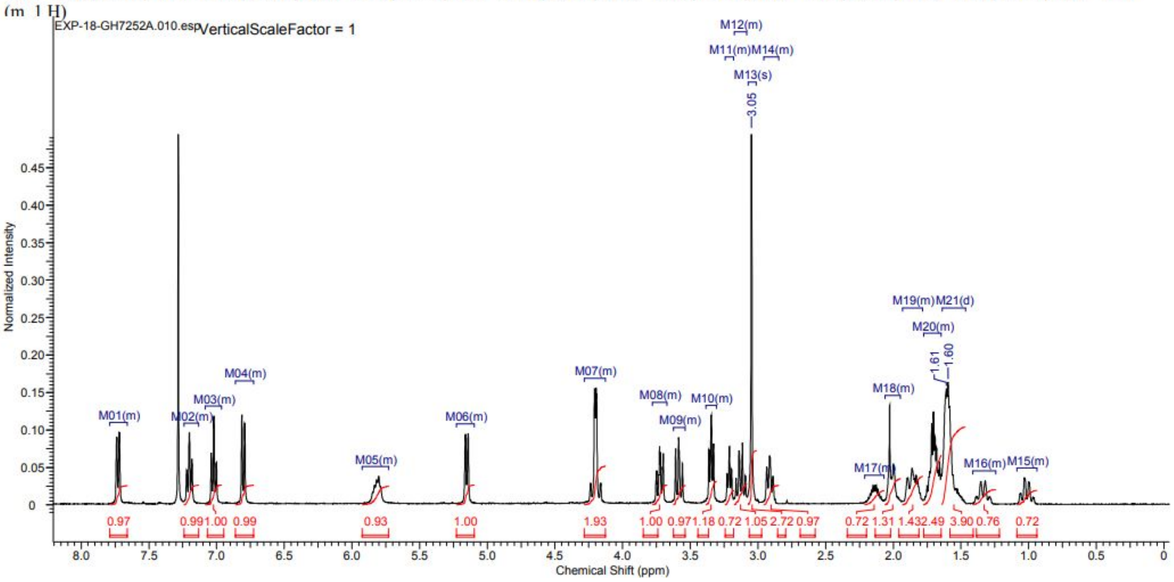

LC-MS Spectra for Compound 24

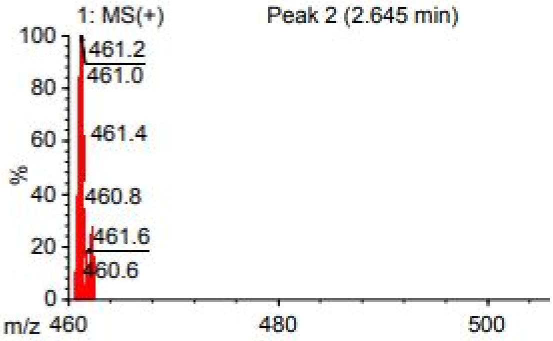

^{1}H NMR Spectra for Compound 25

^{1}H NMR (400 MHz, CHLOROFORM-*d*) δ ppm 0.91 (d, *J*=14.56 Hz, 6 H) 1.09 - 1.25 (m, 4 H) 1.34 - 1.47 (m, 3 H) 1.49 - 1.58 (m, 2 H) 2.86 - 2.96 (m, 1 H) 3.05 (s, 3 H) 3.07 - 3.16 (m, 1 H) 3.17 - 3.25 (m, 2 H) 3.49 - 3.64 (m, 1 H) 3.67 - 3.80 (m, 1 H) 4.14 - 4.28 (m, 2 H) 5.07 - 5.22 (m, 1 H) 5.71 - 5.89 (m, 1 H) 6.73 - 6.86 (m, 1 H) 6.96 - 7.09 (m, 1 H) 7.14 - 7.24 (m, 1 H) 7.67 - 7.79 (m, 1 H)

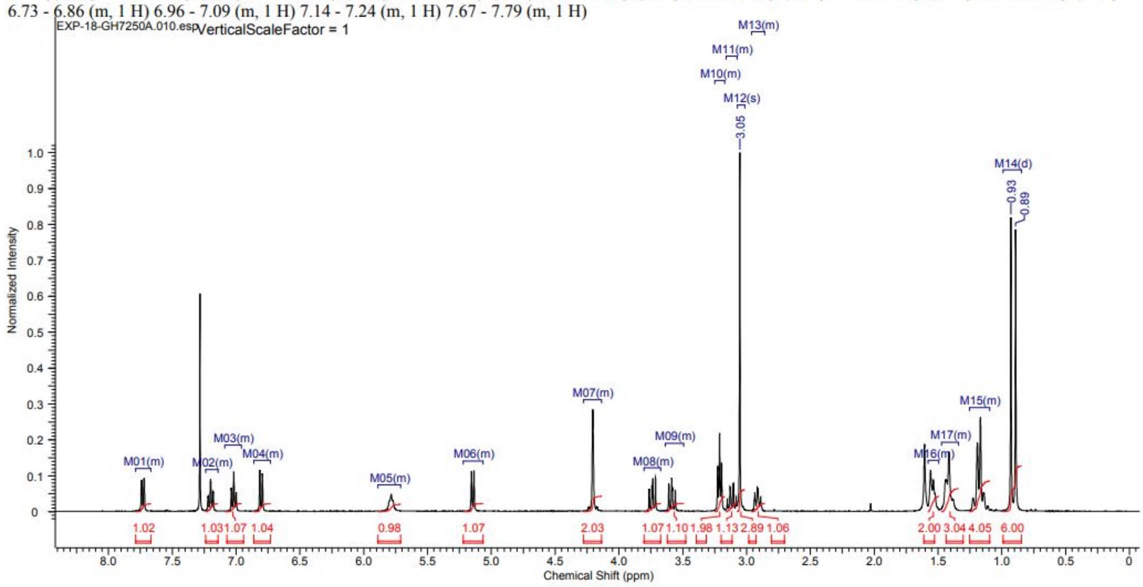

LC-MS Spectra for Compound 25

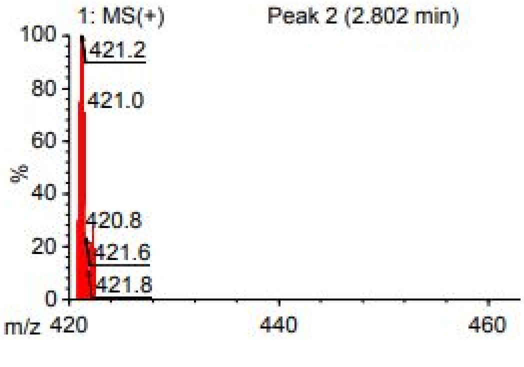

^{1}H NMR Spectra Compound 26

^{1}H NMR (400 MHz, CHLOROFORM-*d*) δ ppm 0.81 - 0.91 (m, 6 H) 0.92 - 1.19 (m, 3 H) 1.31 - 1.58 (m, 5 H) 1.68 - 1.85 (m, 3 H) 2.87 - 2.95 (m, 1 H) 3.05 (s, 3 H) 3.07 - 3.14 (m, 1 H) 3.14 - 3.21 (m, 1 H) 3.25 - 3.34 (m, 1 H) 3.52 - 3.64 (m, 1 H) 3.66 - 3.79 (m, 1 H) 4.13 - 4.26 (m, 2 H) 5.07 - 5.20 (m, 1 H) 5.69 - 5.88 (m, 1 H) 6.72 - 6.87 (m, 1 H) 6.96 - 7.08 (m, 1 H) 7.14 - 7.25 (m, 1 H) 7.67 - 7.79 (m, 1 H)

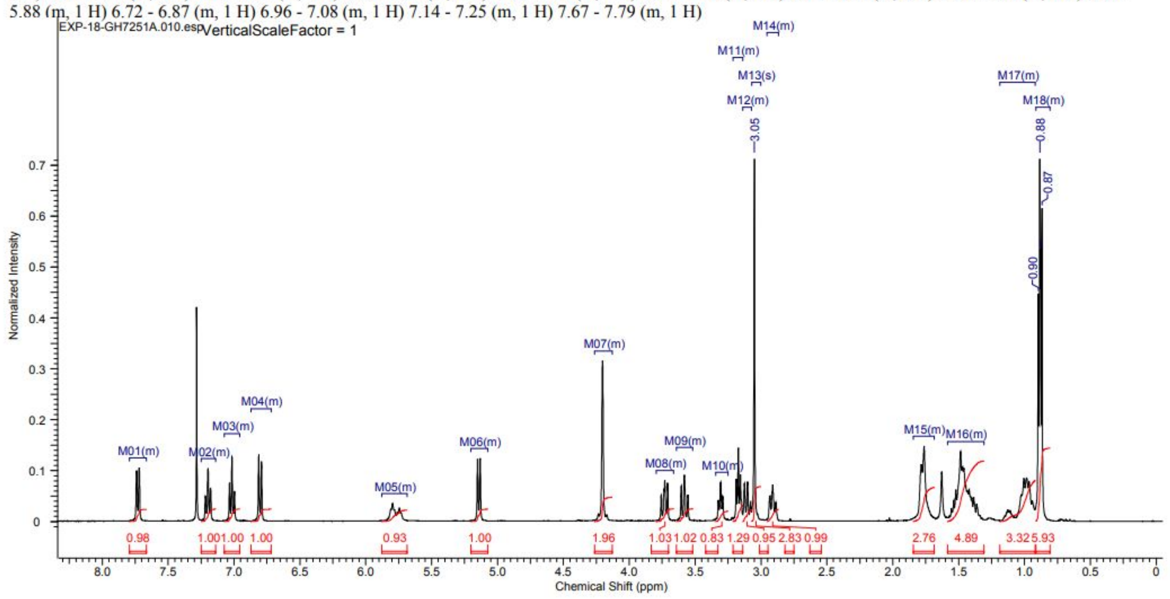

LC-MS Spectra Compound 26

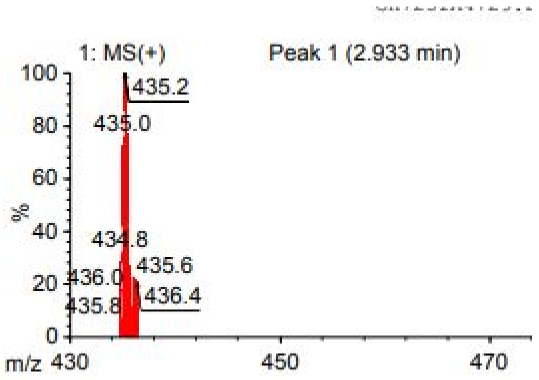

^{1}H NMR Spectra for Compound 27

^{1}H NMR (400 MHz, DMSO-d_6) δ 8.16 - 8.29 (m, 1H), 7.49 - 7.62 (m, 1H), 7.11 - 7.25 (m, 1H), 6.92 - 7.03 (m, 1H), 6.74 - 6.86 (m, 1H), 5.09 - 5.19 (m, 1H), 4.01 - 4.22 (m, 2H), 3.34 - 3.50 (m, 2H), 3.10 (s, 3H), 2.96 - 3.06 (m, 1H), 2.77 - 2.93 (m, 2H), 1.66 - 1.83 (m, 1H), 1.38 (d, J = 10.54 Hz, 2H), 1.21 (d, J = 13.55 Hz, 1H), 1.00 (d, J = 13.80 Hz, 1H), 0.95 (s, 5H), 0.87 (s, 5H), 0.67 (t, J = 12.67 Hz, 2H)

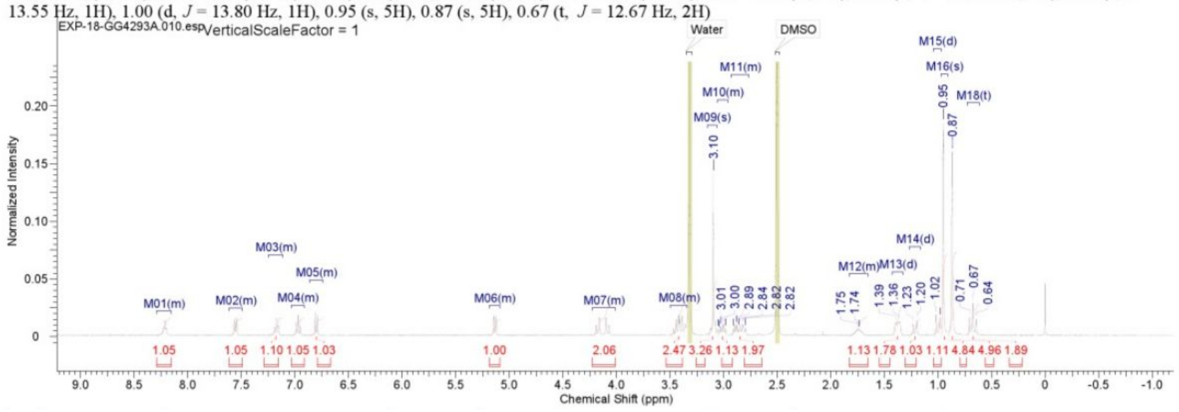

LC-MS Spectra for Compound 27

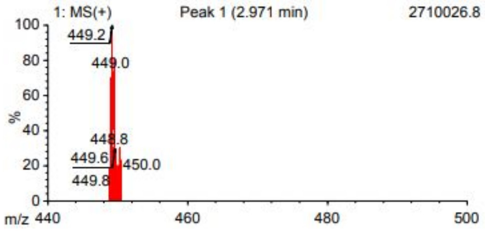

^{1}H NMR Spectra for Compound 28

^{1}H NMR (400 MHz, CHLOROFORM-d) δ ppm 0.89 - 1.08 (m, 1 H) 1.30 - 1.66 (m, 7 H) 1.68 - 1.91 (m, 2 H) 2.04 - 2.48 (m, 4 H) 2.59 - 2.65 (m, 1 H) 2.69 - 2.76 (m, 1 H) 2.86 - 2.95 (m, 1 H) 3.00 - 3.07 (m, 3 H) 3.08 - 3.23 (m, 2 H) 3.24 - 3.34 (m, 1 H) 3.53 - 3.63 (m, 1 H) 3.68 - 3.77 (m, 1 H) 4.20 (s, 2 H) 5.15 (d, J=8.03 Hz, 1 H) 5.90 - 6.03 (m, 1 H) 6.75 - 6.85 (m, 1 H) 6.97 - 7.07 (m, 1 H) 7.14 - 7.24 (m, 1 H) 7.67 - 7.77 (m, 1 H)

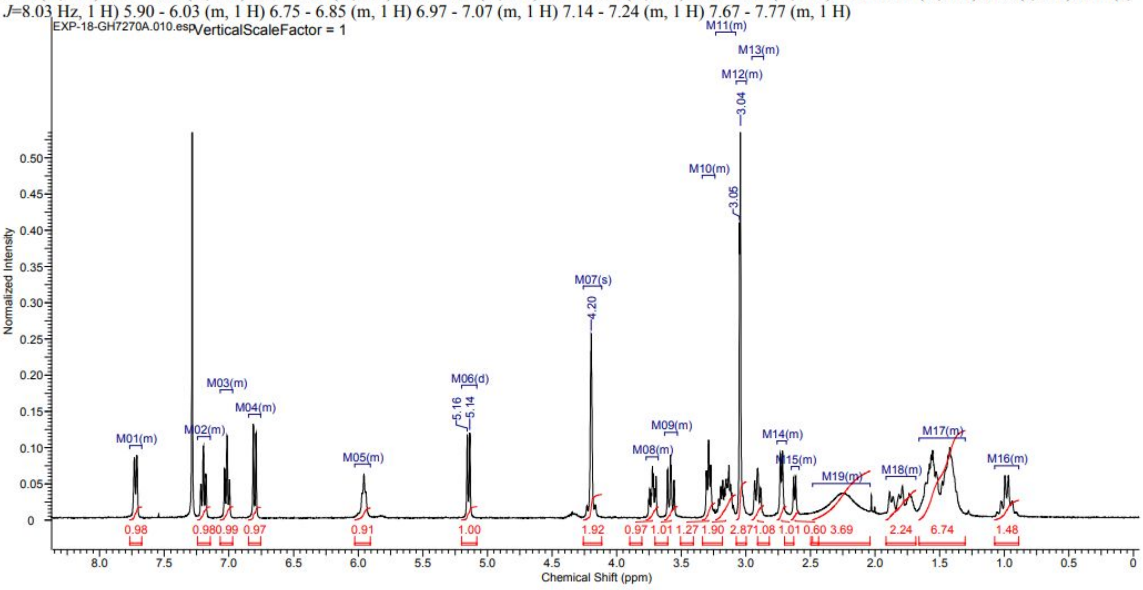

LC-MS Spectra for Compound 28

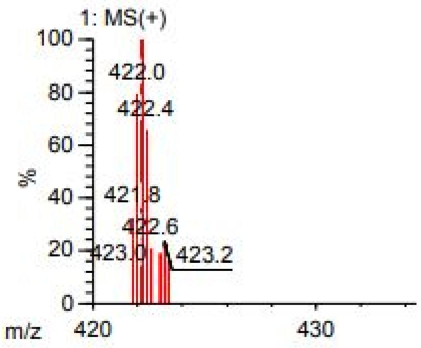

^{1}H NMR Spectra for Compound 29

^{1}H NMR (400 MHz, DMSO-d_6) δ 8.10 - 8.23 (m, 1H), 7.51 - 7.61 (m, 1H), 7.11 - 7.23 (m, 1H), 6.91 - 7.03 (m, 1H), 6.75 - 6.86 (m, 1H), 5.09 - 5.19 (m, 1H), 4.01 - 4.23 (m, 2H), 3.34 - 3.46 (m, 2H), 3.12 - 3.28 (m, 2H), 3.10 (s, 2H), 2.82 (d, J = 2.26 Hz, 1H), 2.14 (br. s., 1H), 1.49 - 1.61 (m, 2H), 1.32 - 1.42 (m, 2H), 1.21 - 1.30 (m, 2H), 1.08 - 1.20 (m, 3H)

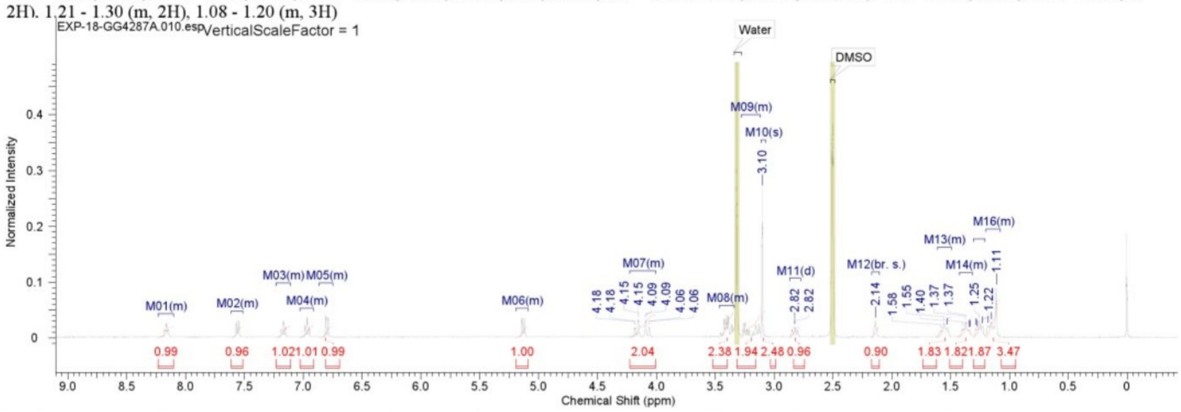

LC-MS Spectra for Compound 29

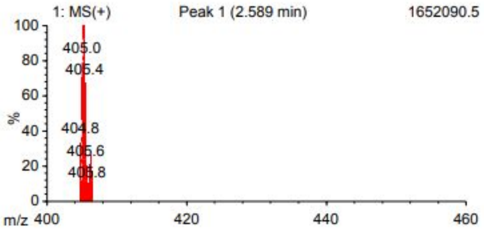

^{1}H NMR Spectra for Compound 30

^{1}H NMR (400 MHz, CHLOROFORM-*d*) δ ppm 1.06 - 1.23 (m, 1 H) 1.41 - 1.56 (m, 1 H) 1.56 - 1.63 (m, 2 H) 1.64 - 1.80 (m, 3 H) 1.83 - 2.02 (m, 3 H) 2.42 - 2.57 (m, 1 H) 2.59 - 2.70 (m, 1 H) 2.87 - 2.98 (m, 1 H) 3.06 (d, *J*=3.76 Hz, 3 H) 3.09 - 3.19 (m, 1 H) 3.20 - 3.29 (m, 1 H) 3.39 - 3.50 (m, 1 H) 3.54 - 3.66 (m, 1 H) 3.69 - 3.81 (m, 1 H) 4.14 - 4.29 (m, 2 H) 5.08 - 5.21 (m, 1 H) 5.70 - 5.89 (m, 1 H) 6.74 - 6.87 (m, 1 H) 6.96 - 7.08 (m, 1 H) 7.17 - 7.27 (m, 4 H) 7.29 - 7.35 (m, 2 H) 7.68 - 7.79 (m, 1 H)

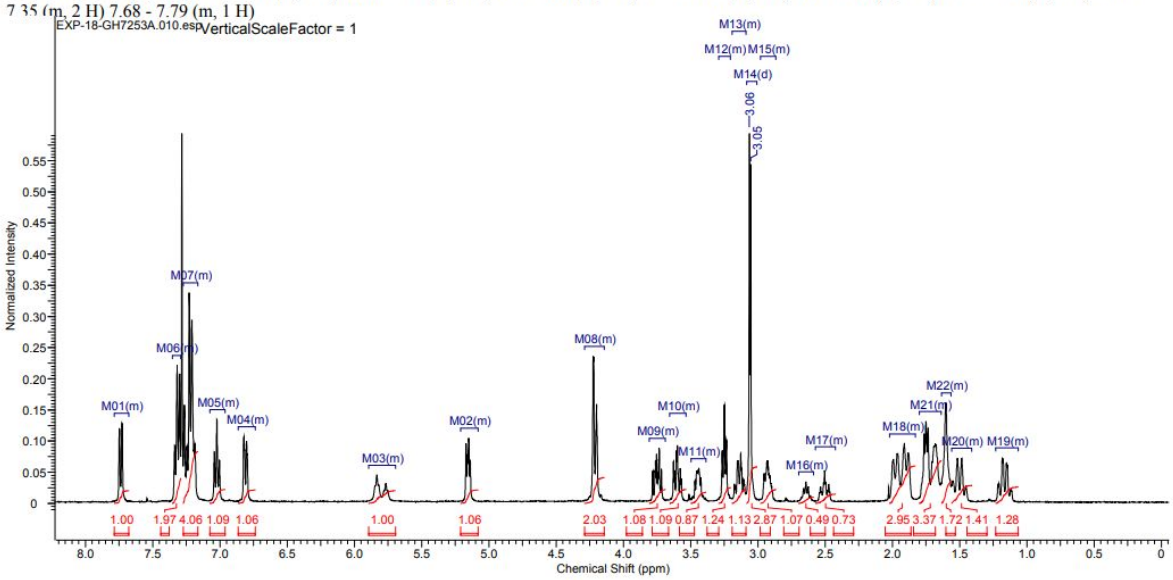

LC-MS Spectra for Compound 30

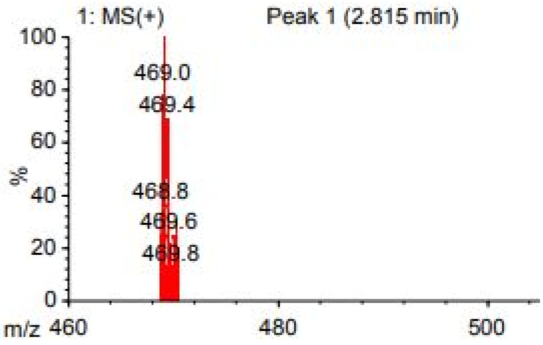

^{1}H NMR Spectra for Compound 31

¹H NMR (400 MHz, CHLOROFORM-*d*) δ ppm 1.39 - 1.55 (m, 1 H) 1.91 - 2.08 (m, 2 H) 2.45 - 2.59 (m, 1 H) 2.76 - 2.98 (m, 4 H) 3.06 (s, 3 H) 3.10 - 3.21 (m, 1 H) 3.28 - 3.48 (m, 2 H) 3.54 - 3.65 (m, 1 H) 3.70 - 3.81 (m, 1 H) 4.15 - 4.26 (m, 2 H) 5.11 - 5.20 (m, 1 H) 5.86 - 5.98 (m, 1 H) 6.77 - 6.84 (m, 1 H) 6.98 - 7.05 (m, 1 H) 7.06 - 7.17 (m, 4 H) 7.17 - 7.24 (m, 1 H) 7.67 - 7.79 (m, 1 H)

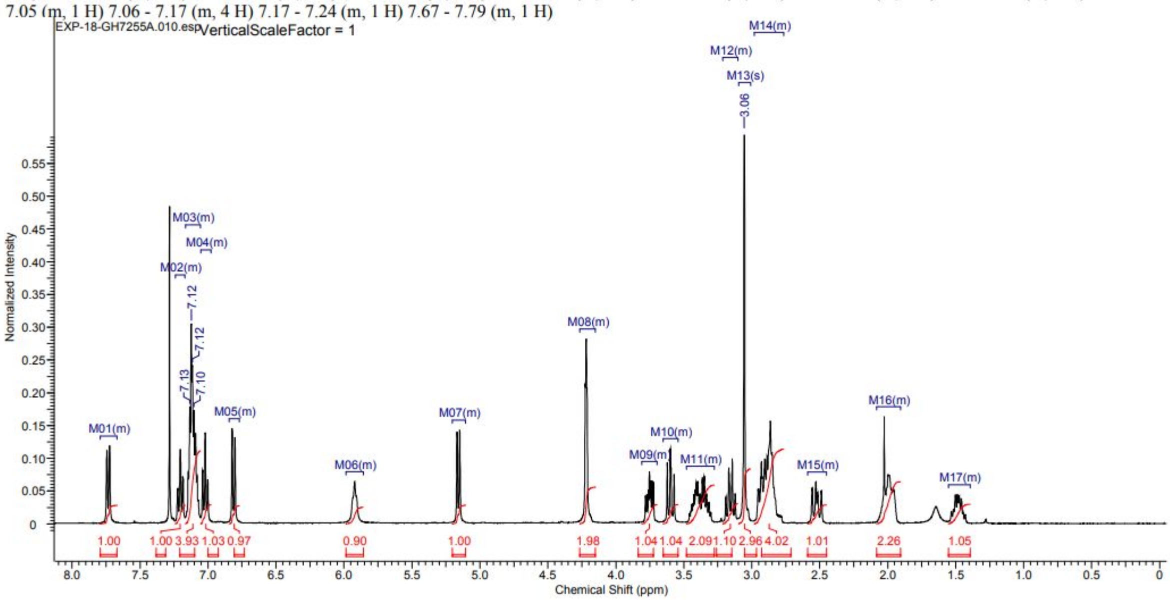

LC-MS Spectra for Compound 31

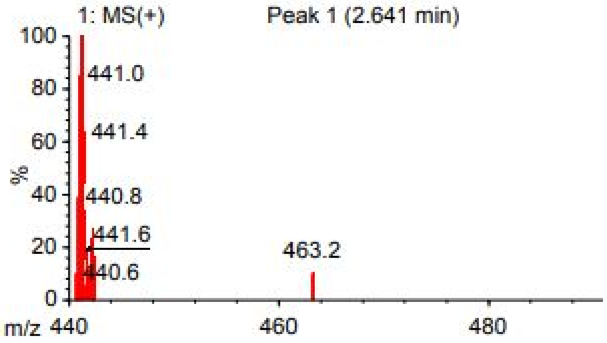

¹H NMR Spectra for Compound 32

^{1}H NMR (400 MHz, CHLOROFORM-*d*) δ ppm 1.66 - 1.84 (m, 2 H) 1.84 - 1.99 (m, 2 H) 2.80 (br. s., 2 H) 2.83 - 2.96 (m, 1 H) 2.99 - 3.17 (m, 5 H) 3.48 - 3.67 (m, 3 H) 3.67 - 3.77 (m, 1 H) 4.00 - 4.23 (m, 2 H) 5.08 - 5.18 (m, 1 H) 5.71 - 5.90 (m, 1 H) 6.74 - 6.83 (m, 1 H) 6.97 - 7.06 (m, 1 H) 7.08 - 7.26 (m, 5 H) 7.66 - 7.78 (m, 1 H)

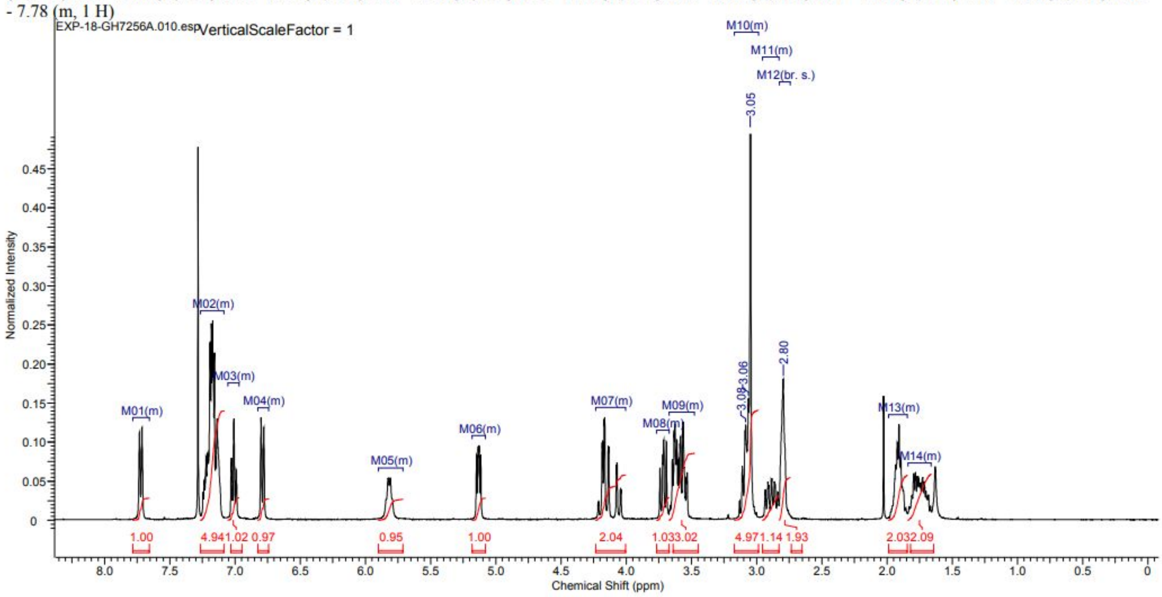

LC-MS Spectra for Compound 32

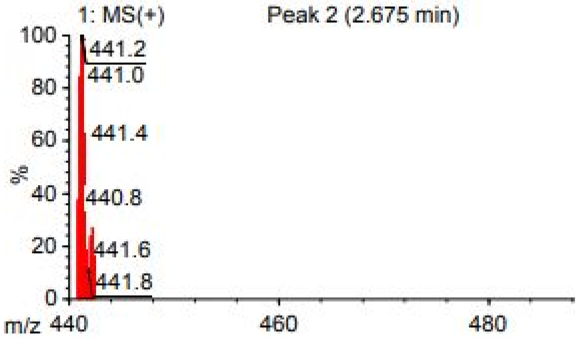

^{1}H NMR Spectra for Compound 33

1H NMR (400 MHz, CHLOROFORM-*d*) δ ppm 1.80 (br. s., 4 H) 2.68 - 2.81 (m, 4 H) 2.93 - 3.02 (m, 1 H) 3.06 (s, 3 H) 3.19 - 3.34 (m, 1 H) 3.56 - 3.69 (m, 1 H) 3.74 - 3.87 (m, 1 H) 4.15 - 4.33 (m, 2 H) 5.10 - 5.24 (m, 1 H) 6.74 - 6.89 (m, 1 H) 6.97 - 7.09 (m, 2 H) 7.14 - 7.25 (m, 2 H) 7.42 - 7.52 (m, 1 H) 7.69 - 7.78 (m, 1 H)

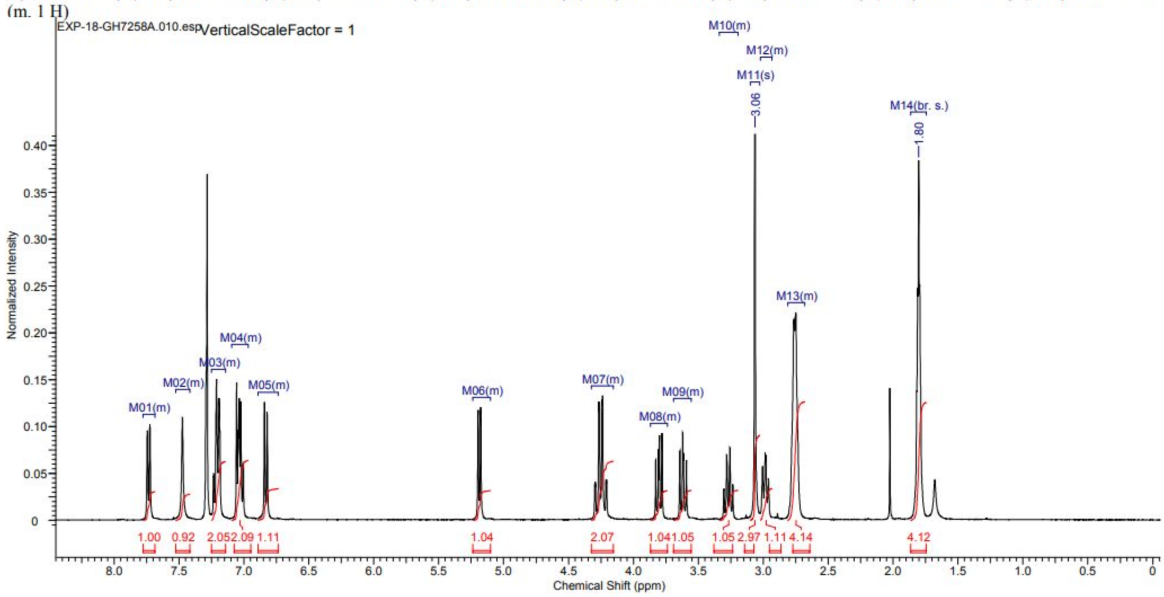

LC-MS Spectra for Compound 33

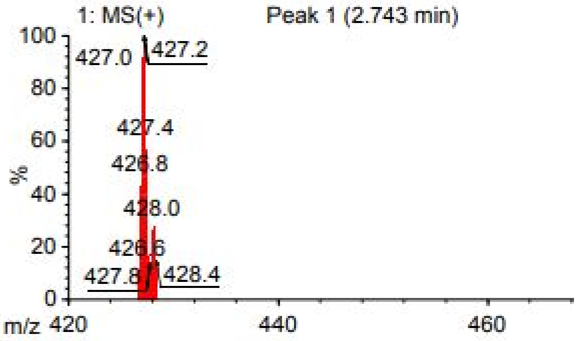

^{1}H NMR Spectra for Compound 34

^{1}H NMR (400 MHz, CHLOROFORM-*d*) δ ppm 2.73 (br. s., 3 H) 2.83 - 2.93 (m, 1 H) 3.05 (s, 3 H) 3.07 - 3.18 (m, 3 H) 3.45 (s, 2 H) 3.49 - 3.60 (m, 1 H) 3.66 - 3.78 (m, 1 H) 4.07 - 4.27 (m, 2 H) 5.09 - 5.21 (m, 1 H) 5.77 - 5.90 (m, 1 H) 6.77 - 6.84 (m, 1 H) 6.97 - 7.06 (m, 1 H) 7.13 - 7.25 (m, 5 H) 7.67 - 7.79 (m, 1 H)

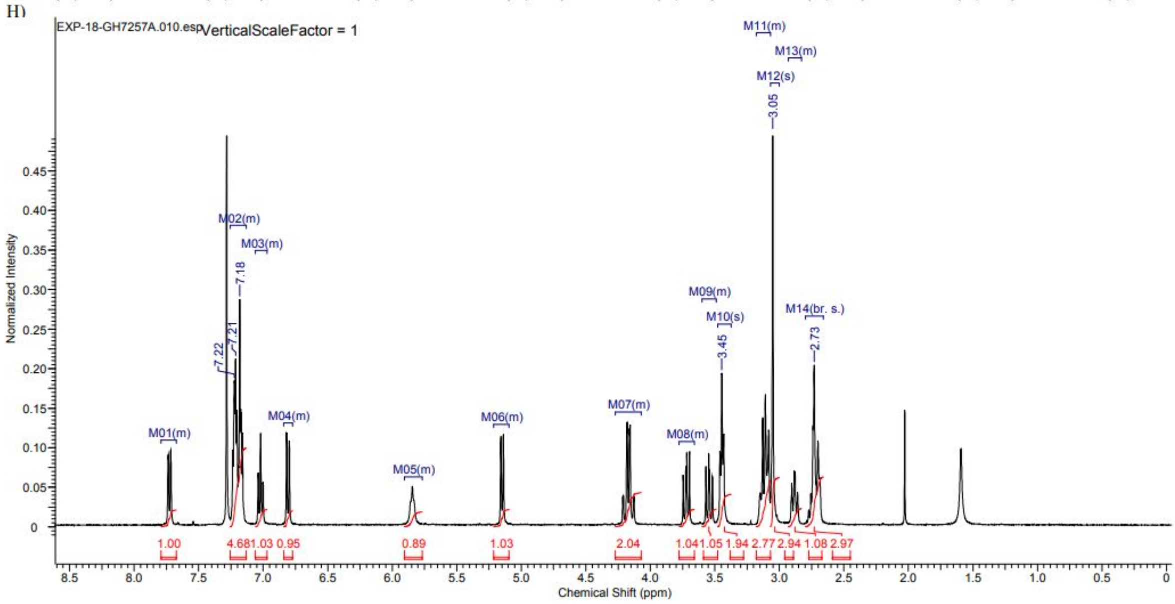

LC-MS Spectra for Compound 34

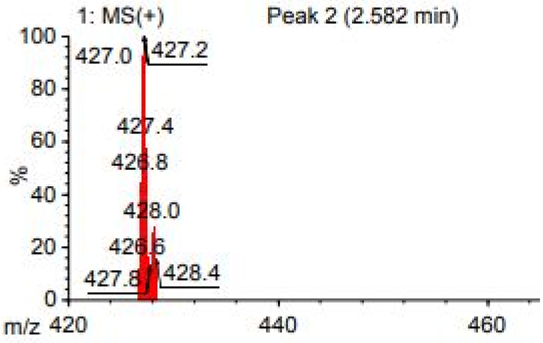

^{1}H NMR Spectra for Compound 35

^{1}H NMR (400 MHz, CHLOROFORM-*d*) δ ppm 2.88 - 2.98 (m, 1 H) 3.05 (s, 3 H) 3.09 - 3.23 (m, 1 H) 3.51 - 3.65 (m, 1 H) 3.69 - 3.83 (m, 1 H) 4.19 (d, *J*=1.76 Hz, 2 H) 4.42 - 4.59 (m, 2 H) 5.07 - 5.21 (m, 1 H) 6.01 - 6.23 (m, 1 H) 6.79 (d, *J*=0.50 Hz, 1 H) 6.95 - 7.06 (m, 1 H) 7.11 - 7.23 (m, 1 H) 7.24 - 7.43 (m, 5 H) 7.64 - 7.79 (m, 1 H)

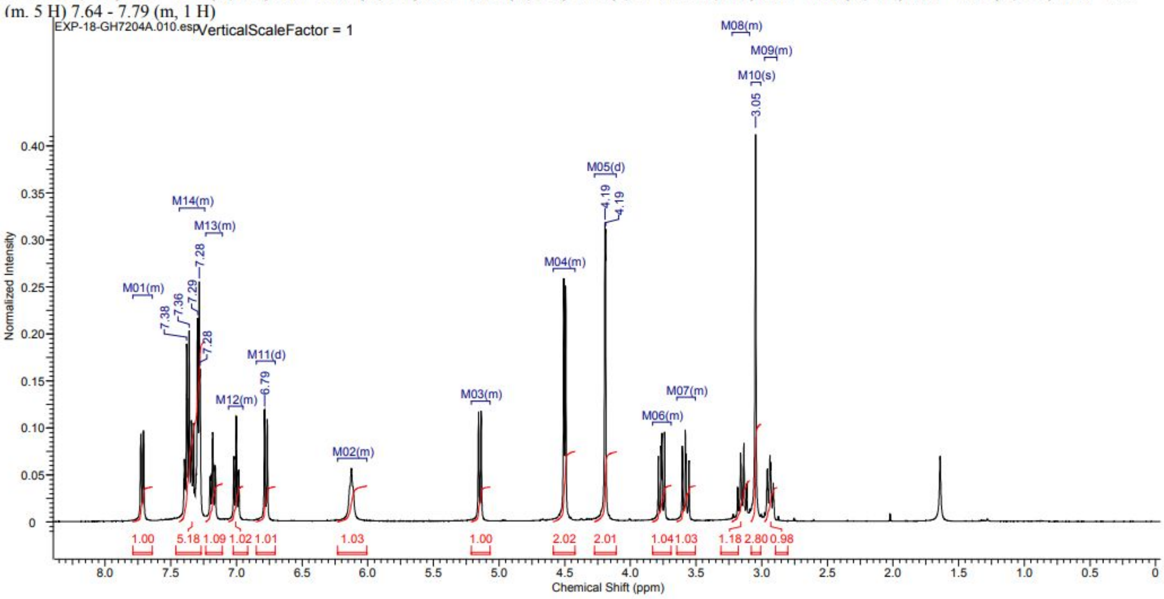

LC-MS Spectra for Compound 35

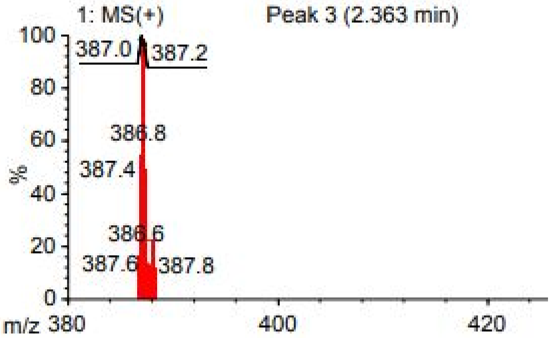

^{1}H NMR Spectra for Compound 36

^{1}H NMR (400 MHz, DMSO-d_6) δ 8.03 - 8.14 (m, 1H), 7.51 - 7.61 (m, 1H), 7.12 - 7.22 (m, 1H), 6.91 - 7.03 (m, 1H), 6.80 (d, J = 8.03 Hz, 1H), 5.13 (d, J = 8.03 Hz, 1H), 4.01 - 4.23 (m, 2H), 3.36 - 3.44 (m, 2H), 3.13 - 3.25 (m, 1H), 3.02 - 3.11 (m, 4H), 2.89 - 3.00 (m, 1H), 2.83 (dd, J = 8.16, 10.42 Hz, 1H), 1.07 - 1.54 (m, 9H), 0.81 (s, 3H)

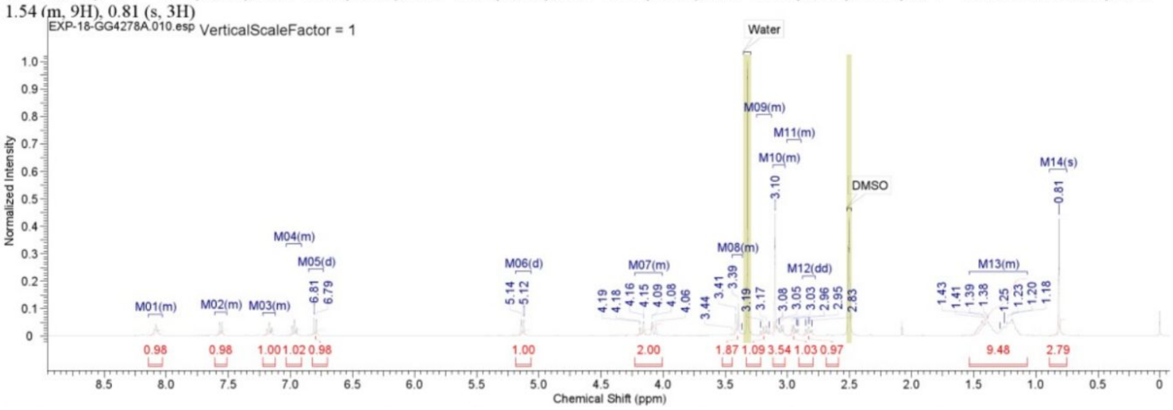

LC-MS Spectra for Compound 36

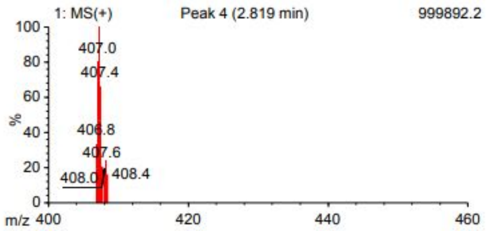

^{1}H NMR Spectra for Compound 37

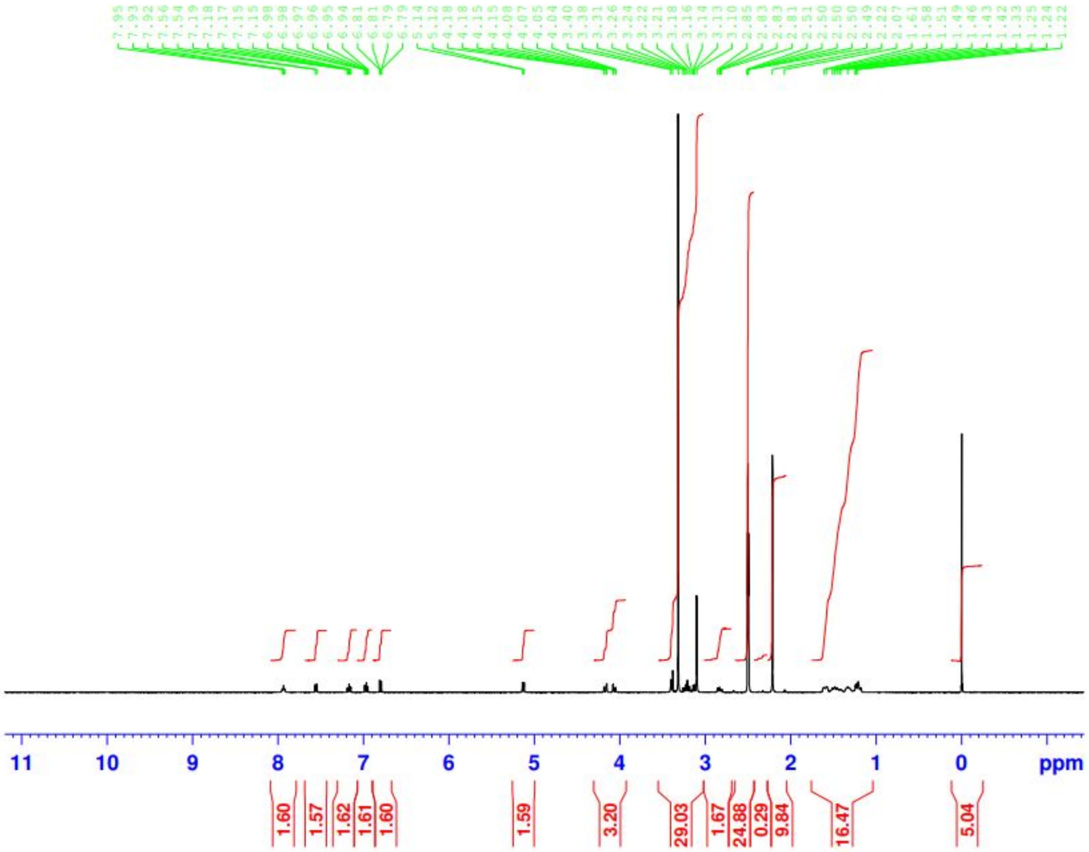

LC-MS Spectra for Compound 37

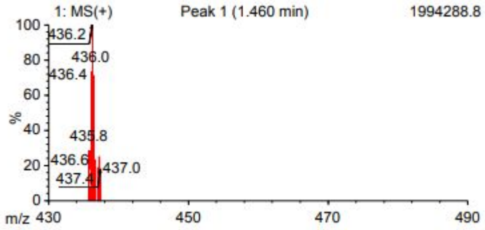

^{1}H NMR Spectra for Compound 38

1H NMR (400 MHz, DMSO-d6) δ 7.98 - 8.12 (m, 1H), 7.50 - 7.63 (m, 1H), 7.13 - 7.23 (m, 1H), 6.92 - 7.00 (m, 1H), 6.75 - 6.86 (m, 1H), 5.06 - 5.17 (m, 1H), 4.20 (s, 1H), 4.06 - 4.18 (m, 2H), 3.40 (s, 2H), 3.14 - 3.25 (m, 2H), 3.09 (s, 2H), 2.96 - 3.04 (m, 1H), 2.78 - 2.88 (m, 1H), 1.54 (d, J = 8.78 Hz, 2H), 1.39 (d, J = 9.54 Hz, 4H), 1.11 - 1.33 (m, 3H)

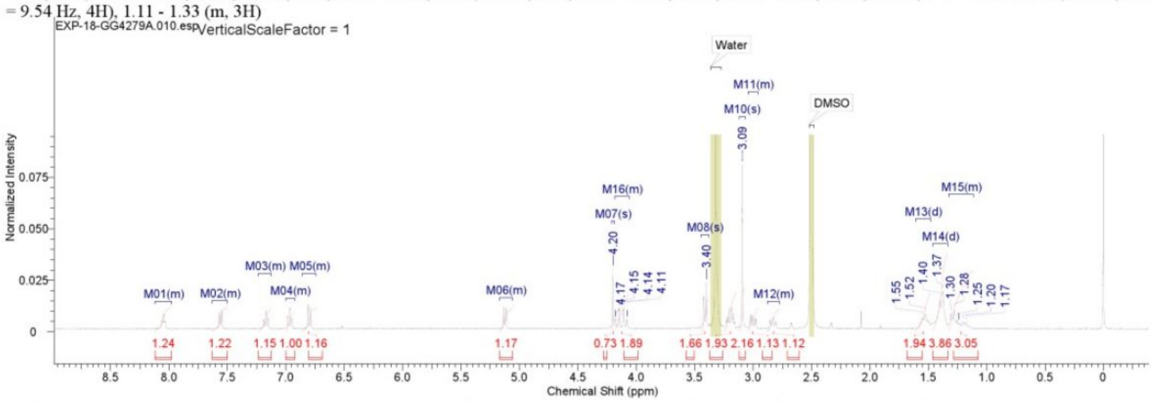

LC-MS Spectra for Compound 38

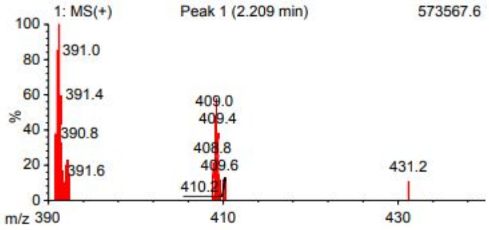

^{1}H NMR Spectra for Compound 39

1H NMR (400 MHz, DMSO-d6) δ 7.90 - 8.04 (m, 1H), 7.50 - 7.61 (m, 1H), 7.10 - 7.22 (m, 1H), 6.91 - 7.03 (m, 1H), 6.73 - 6.86 (m, 1H), 5.09 - 5.21 (m, 1H), 4.05 - 4.26 (m, 2H), 3.48 - 3.59 (m, 3H), 3.37 - 3.44 (m, 2H), 3.14 - 3.24 (m, 2H), 3.10 (s, 3H), 2.79 - 2.88 (m, 1H), 2.55 (br. s., 3H), 1.58 - 1.69 (m, 2H), 1.43 - 1.56 (m, 2H), 1.27 - 1.38 (m, 2H), 1.15 - 1.25 (m, 2H)

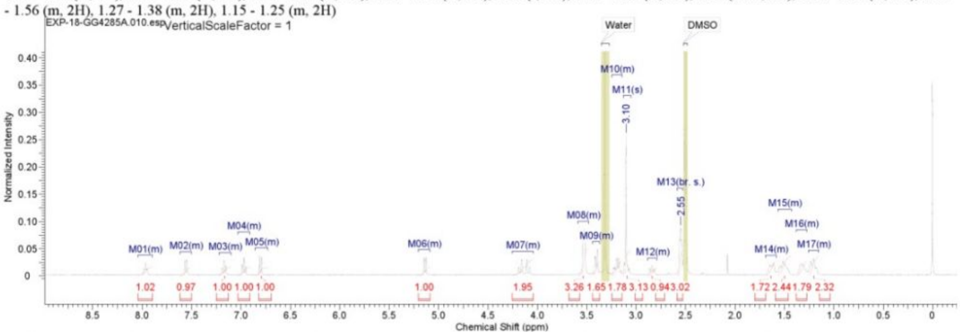

LC-MS Spectra for Compound 39

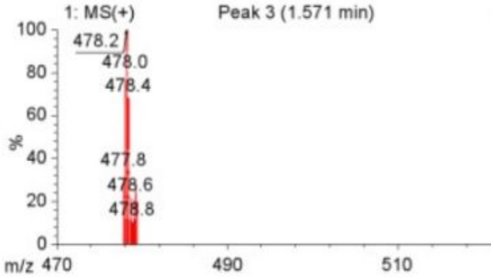

^{1}H NMR Spectra for Compound 40

^{1}H NMR (400 MHz, DMSO-d_6) δ 7.80 - 7.92 (m, 1H), 7.47 - 7.57 (m, 1H), 7.25 - 7.41 (m, 4H), 7.09 - 7.24 (m, 2H), 6.90 - 7.01 (m, 1H), 6.72 - 6.84 (m, 1H), 5.01 - 5.08 (m, 1H), 4.01 - 4.11 (m, 1H), 3.81 - 3.93 (m, 1H), 3.12 - 3.30 (m, 4H), 3.01 (s, 4H), 2.58 - 2.70 (m, 1H), 2.07 (s, 5H), 1.47 - 1.62 (m, 4H), 1.13 - 1.47 (m, 4H)

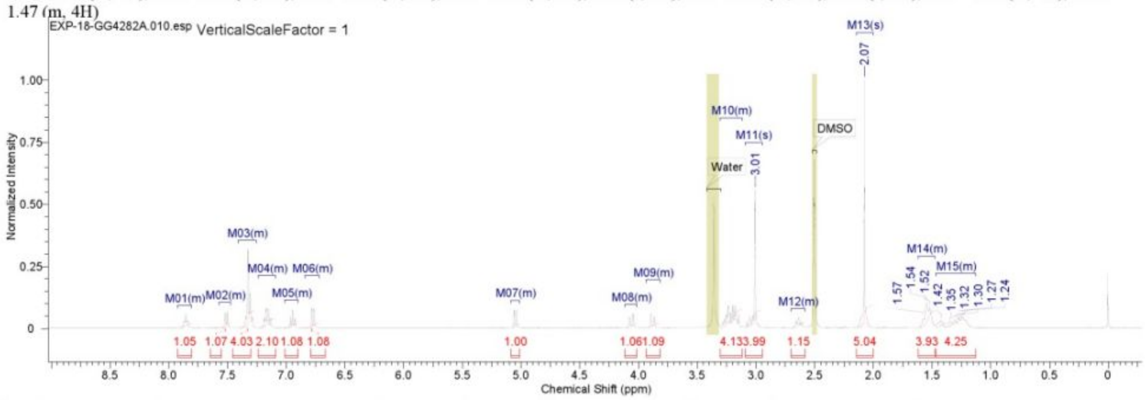

LC-MS Spectra for Compound 40

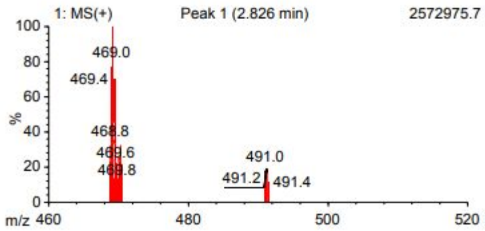

^{1}H NMR Spectra for Compound 41

^{1}H NMR (400 MHz, CHLOROFORM-d) δ ppm 0.73 - 1.06 (m, 2 H) 1.07 - 1.35 (m, 3 H) 1.50 - 1.85 (m, 7 H) 2.85 - 3.35 (m, 9 H) 3.49 - 3.76 (m, 3 H) 4.02 - 4.28 (m, 2 H) 5.13 (d, J=8.03 Hz, 1 H) 6.76 - 6.88 (m, 1 H) 7.03 (s, 1 H) 7.21 (s, 1 H) 7.70 - 7.83 (m, 1 H)

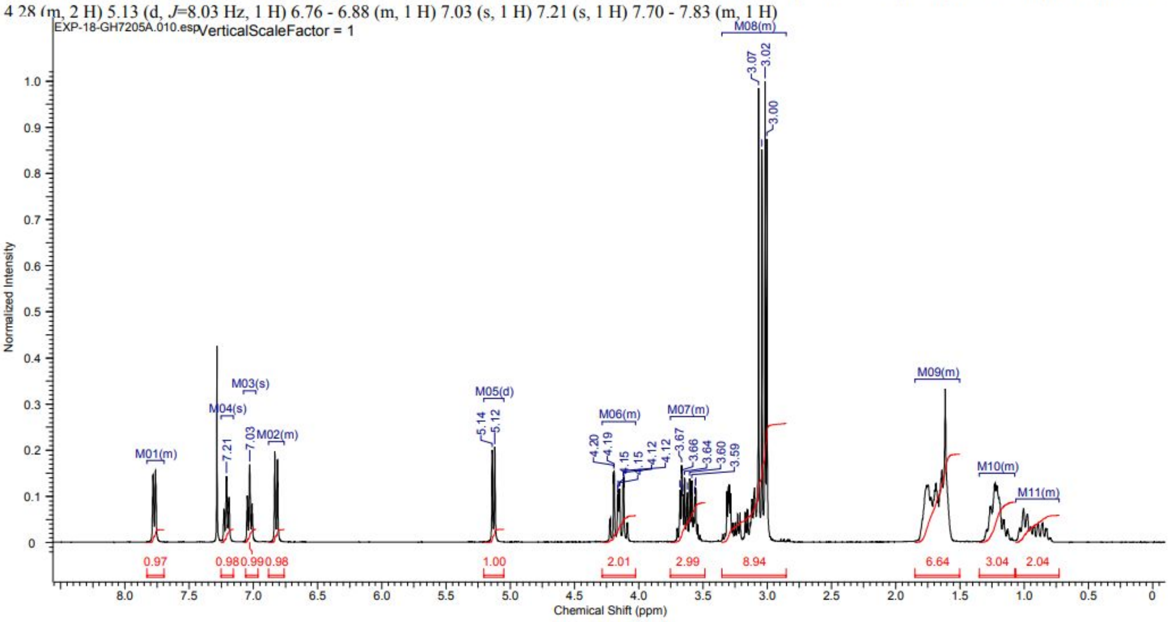

LC-MS Spectra for Compound 41

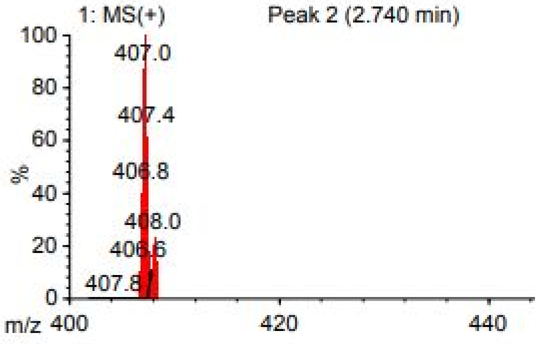

^{1}H NMR Spectra for Compound 42

^{1}H NMR (400 MHz, CHLOROFORM-*d*) δ ppm 0.85 - 1.06 (m, 2 H) 1.11 - 1.34 (m, 3 H) 1.38 - 1.59 (m, 1 H) 1.66 - 1.85 (m, 6 H) 2.12 (s, 3 H) 2.74 - 2.91 (m, 1 H) 3.08 - 3.29 (m, 3 H) 3.59 - 3.74 (m, 1 H) 3.81 - 3.93 (m, 1 H) 4.19 (d, *J*=1.51 Hz, 2 H) 5.66 - 5.73 (m, 1 H) 5.75 - 5.86 (m, 1 H) 6.73 - 6.83 (m, 1 H) 6.89 - 6.98 (m, 1 H) 7.11 - 7.21 (m, 1 H) 7.67 - 7.79 (m, 1 H)

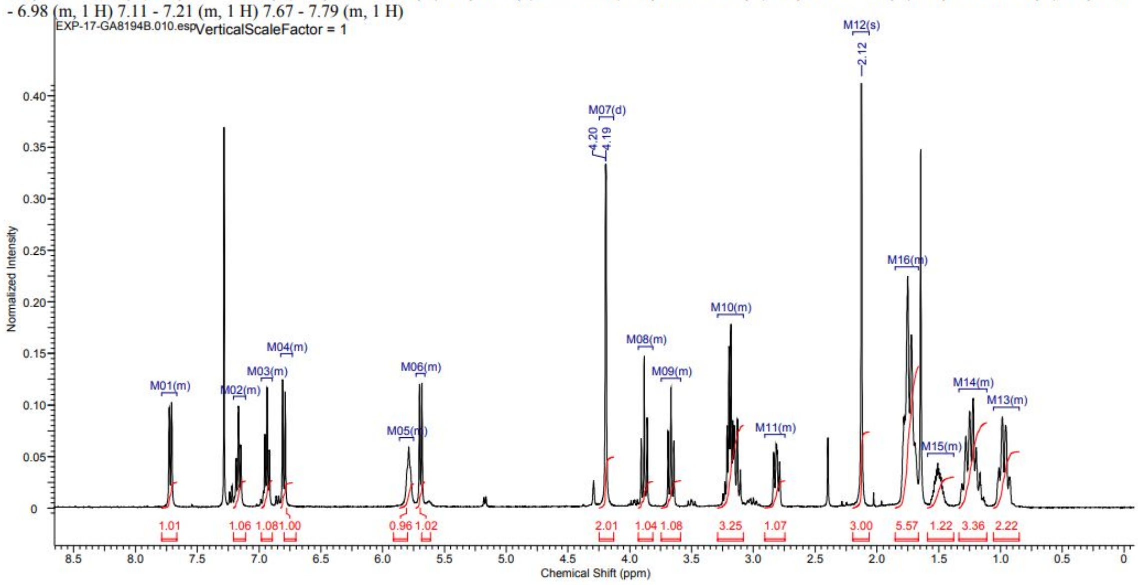

LC-MS Spectra for Compound 42

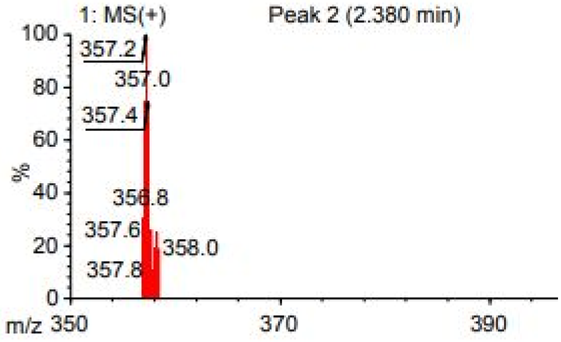

^{1}H NMR Spectra for Compound 43

^{1}H NMR (400 MHz, CHLOROFORM-*d*) δ ppm 1.46 - 1.61 (m, 1 H) 1.83 - 2.10 (m, 3 H) 2.85 - 2.96 (m, 1 H) 3.05 (s, 3 H) 3.16 (s, 2 H) 3.60 (d, *J*=9.03 Hz, 2 H) 3.70 - 3.82 (m, 2 H) 3.83 - 3.92 (m, 1 H) 3.93 - 4.04 (m, 1 H) 4.14 - 4.28 (m, 2 H) 5.04 - 5.21 (m, 1 H) 6.02 - 6.22 (m, 1 H) 6.74 - 6.85 (m, 1 H) 6.95 - 7.08 (m. 1 H) 7.14 - 7.24 (m, 1 H) 7.67 - 7.78 (m, 1 H)

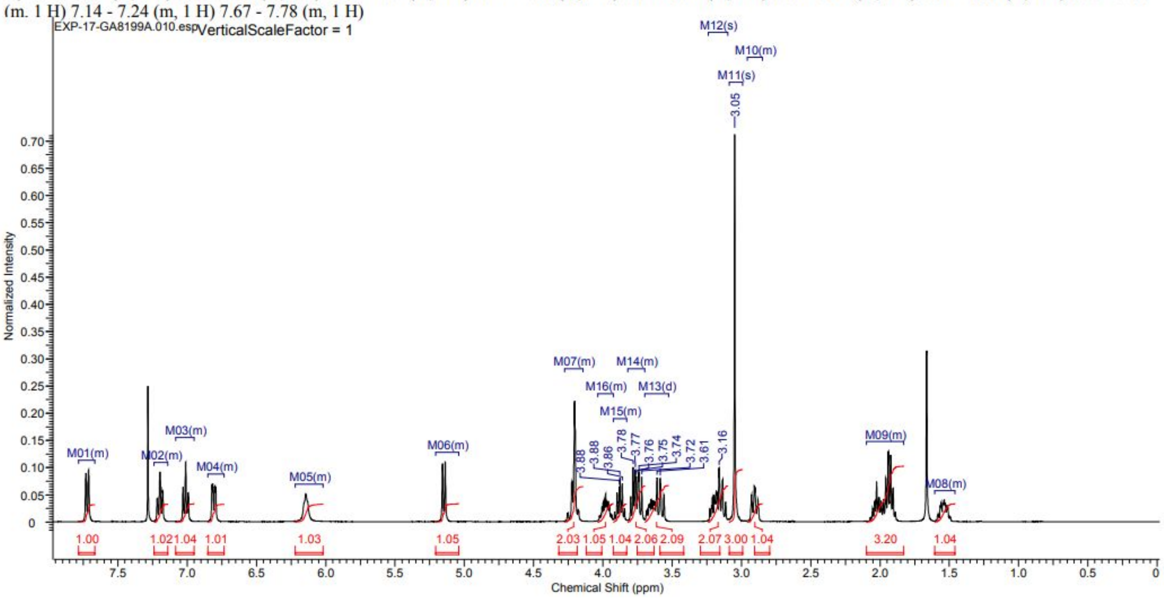

LC-MS Spectra for Compound 43

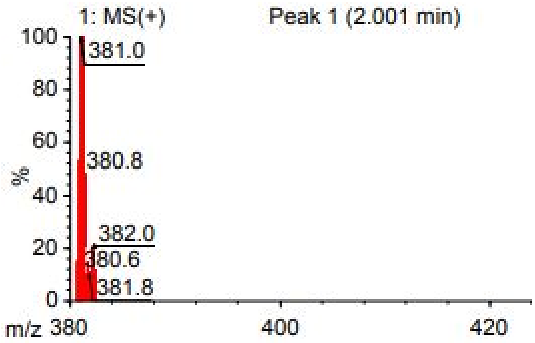

^{1}H NMR Spectra for Compound 44

1H NMR (400 MHz, CHLOROFORM-*d*) δ ppm 1.43 - 1.64 (m, 1 H) 1.84 - 2.08 (m, 3 H) 2.12 (s, 3 H) 2.71 - 2.87 (m, 1 H) 3.20 (s, 2 H) 3.62 - 3.73 (m, 2 H) 3.78 (d, *J*=7.03 Hz, 1 H) 3.82 - 3.93 (m, 2 H) 3.94 - 4.05 (m, 1 H) 4.19 (s, 2 H) 5.60 - 5.78 (m, 1 H) 6.09 - 6.26 (m, 1 H) 6.75 - 6.84 (m, 1 H) 6.89 - 6.97 (m, 1 H) 7.10 - 7.20 (m, 1 H) 7.64 - 7.76 (m, 1 H)

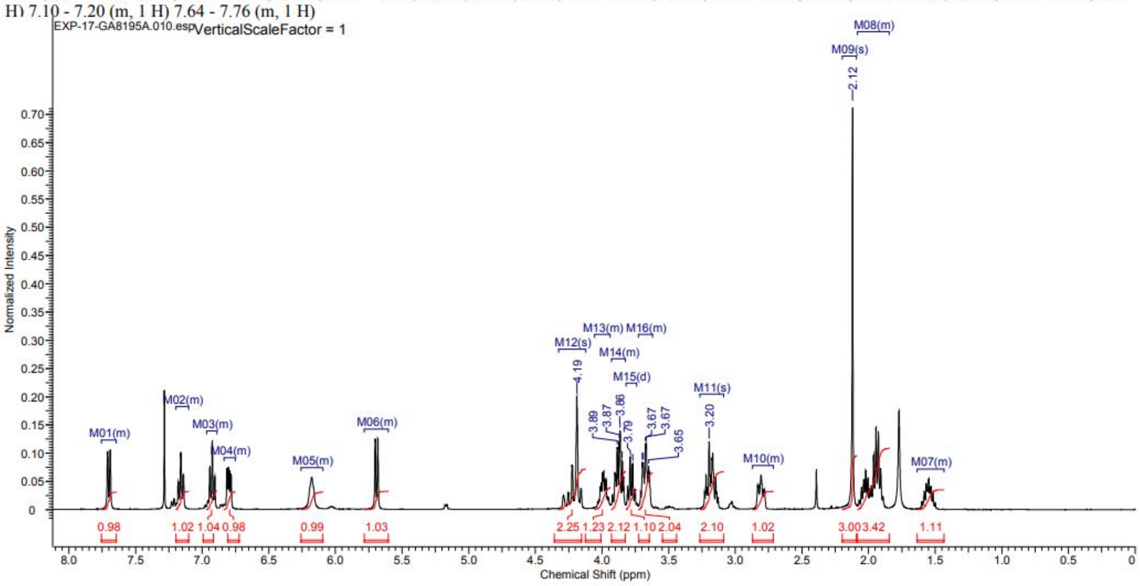

LC-MS Spectra for Compound 44

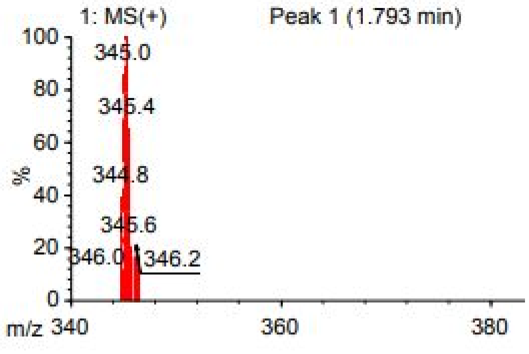

^{1}H NMR Spectra for Compound 45

1H NMR (400 MHz, CHLOROFORM-*d*) δ ppm 1.18 - 1.39 (m, 1 H) 1.41 - 1.64 (m, 4 H) 1.81 - 1.96 (m, 1 H) 2.86 - 2.97 (m, 1 H) 3.05 (d, *J*=2.01 Hz, 3 H) 3.08 - 3.23 (m, 2 H) 3.32 - 3.50 (m, 2 H) 3.52 - 3.66 (m, 2 H) 3.68 - 3.80 (m, 1 H) 3.92 - 4.05 (m, 1 H) 4.21 (d, *J*=1.51 Hz, 2 H) 5.08 - 5.19 (m, 1 H) 6.04 - 6.26 (m, 1 H) 6.72 - 6.86 (m, 1 H) 6.97 - 7.07 (m, 1 H) 7.14 - 7.24 (m, 1 H) 7.66 - 7.77 (m, 1 H)

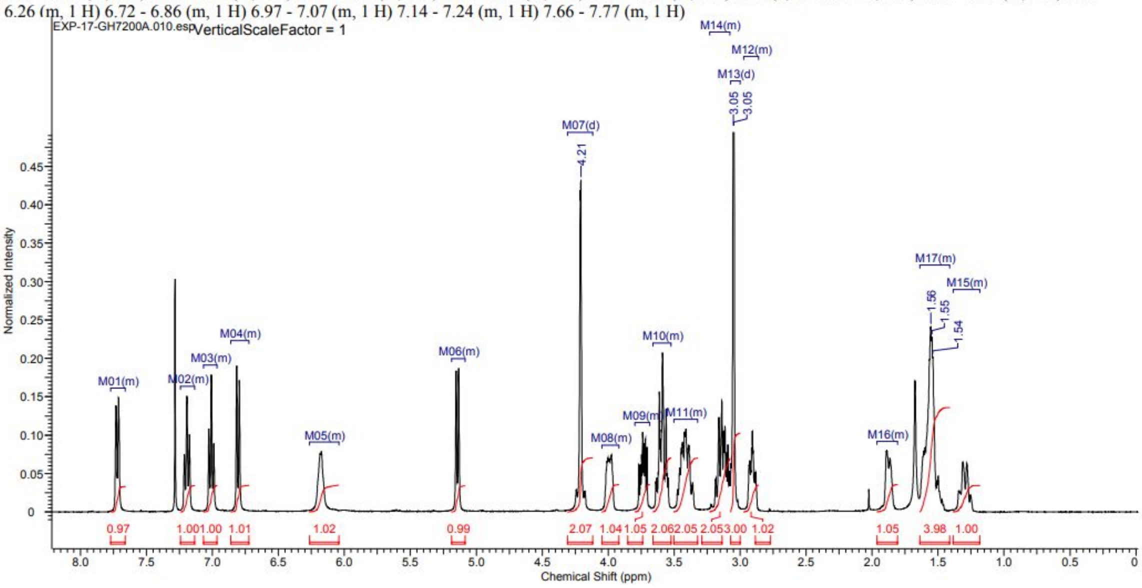

LC-MS Spectra for Compound 45

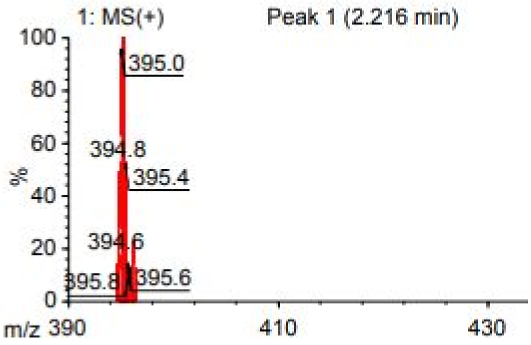

1H NMR Spectra for Compound 46

1H NMR (400 MHz, CHLOROFORM-*d*) δ ppm 1.19 - 1.41 (m, 1 H) 1.44 - 1.66 (m, 4 H) 1.82 - 1.93 (m, 1 H) 2.13 (d, *J*=1.00 Hz, 3 H) 2.75 - 2.86 (m, 1 H) 3.01 - 3.23 (m, 2 H) 3.34 - 3.51 (m, 2 H) 3.56 - 3.72 (m, 2 H) 3.82 - 3.92 (m, 1 H) 3.94 - 4.08 (m, 1 H) 4.20 (d, *J*=6.78 Hz, 2 H) 5.70 (d, *J*=7.78 Hz, 1 H) 6.17 (br. s., 1 H) 6.80 (d, *J*=8.28 Hz, 1 H) 6.93 (s, 1 H) 7.16 (s, 1 H) 7.66 - 7.75 (m, 1 H)

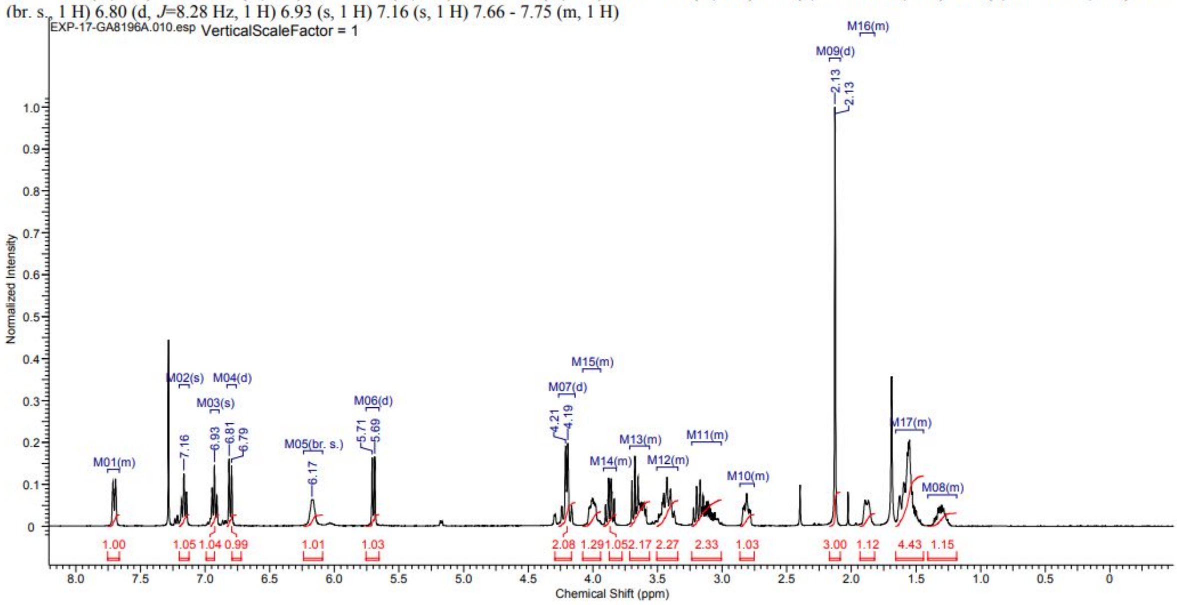

LC-MS Spectra for Compound 46

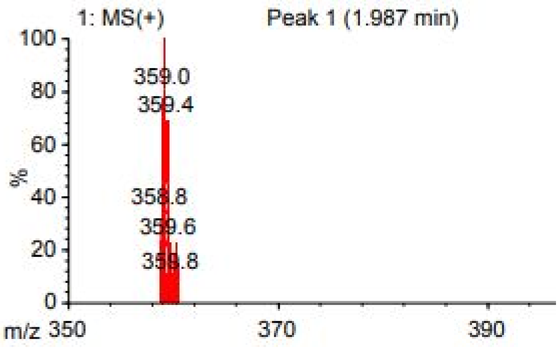

^{1}H NMR Spectra for Compound 47

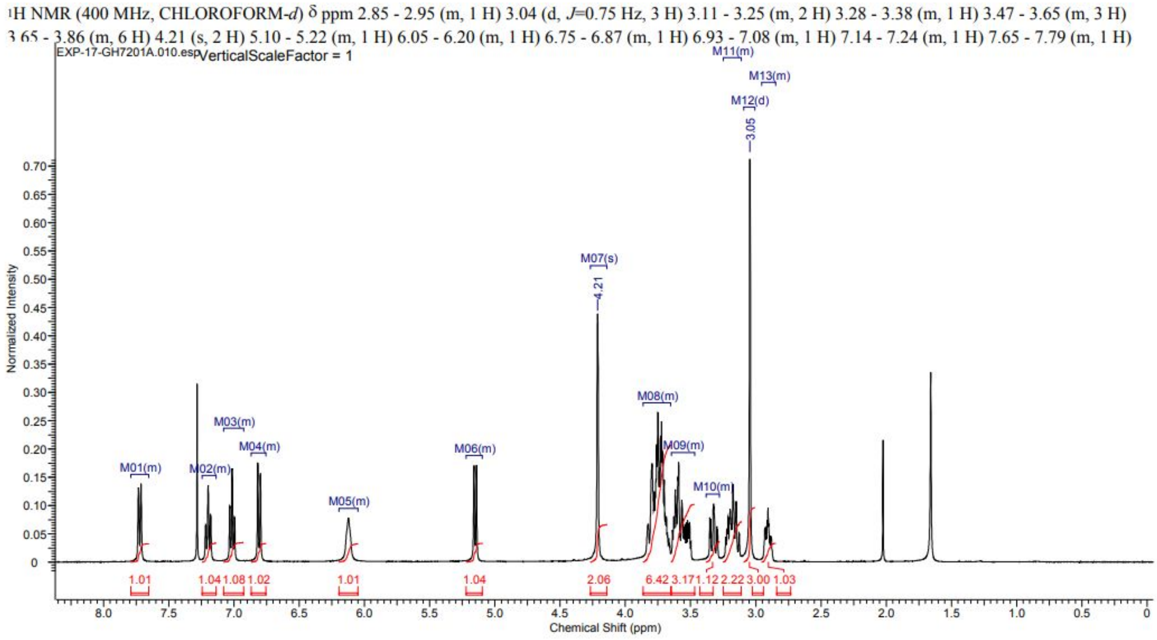

LC-MS Spectra for Compound 47

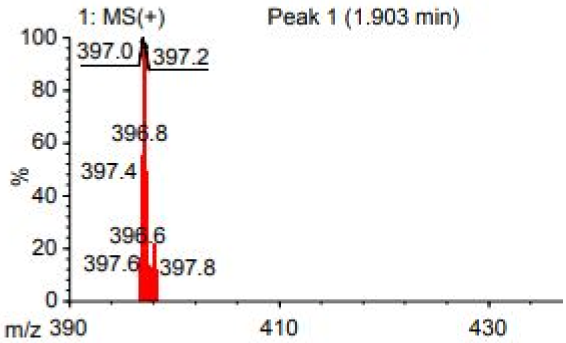

^{1}H NMR Spectra for Compound 48

^{1}H NMR (400 MHz, CHLOROFORM-*d*) δ ppm 2.13 (s, 3 H) 2.69 - 2.87 (m, 1 H) 3.13 - 3.26 (m, 2 H) 3.27 - 3.39 (m, 1 H) 3.47 - 3.92 (m, 10 H) 4.13 - 4.27 (m, 2 H) 5.60 - 5.77 (m, 1 H) 6.05 - 6.21 (m, 1 H) 6.77 - 6.83 (m, 1 H) 6.88 - 6.96 (m, 1 H) 7.12 - 7.20 (m, 1 H) 7.65 - 7.75 (m, 1 H)

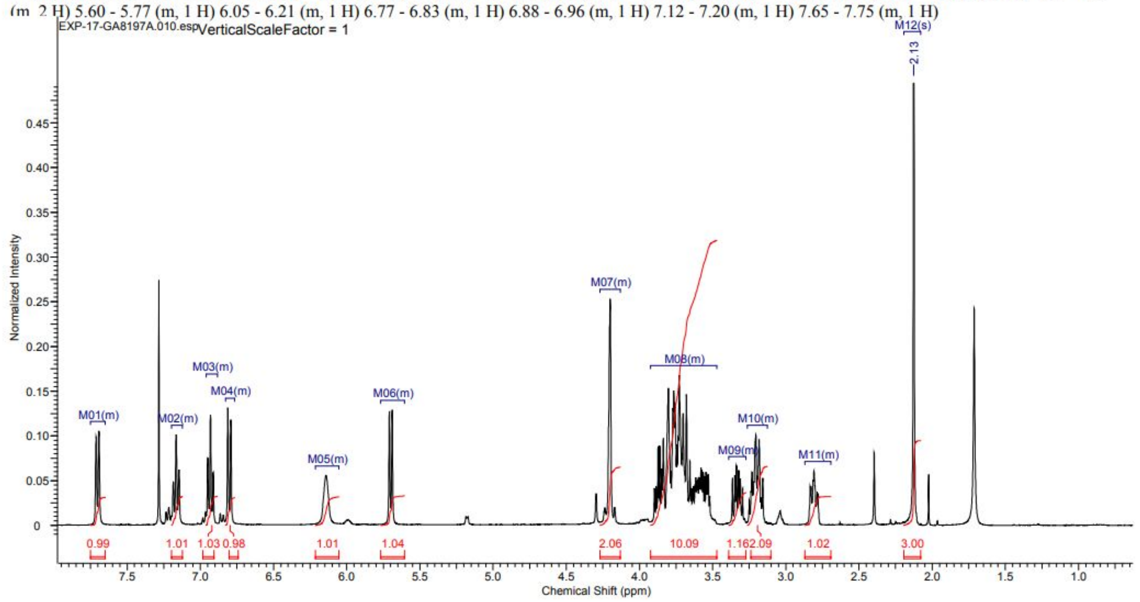

LC-MS Spectra for Compound 48

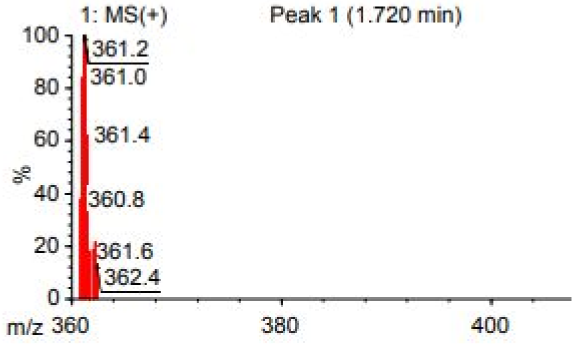

^{1}H NMR Spectra for Compound 49

1H NMR (400 MHz, CHLOROFORM-*d*) δ ppm 2.82 - 2.96 (m, 1 H) 3.04 (s, 3 H) 3.13 - 3.25 (m, 1 H) 3.44 - 3.63 (m, 2 H) 3.76 (s, 2 H) 3.90 - 4.04 (m, 1 H) 4.19 (d, *J*=1.76 Hz, 1 H) 4.30 (d, *J*=12.05 Hz, 3 H) 5.16 (d, *J*=7.78 Hz, 1 H) 6.17 - 6.29 (m, 1 H) 6.77 - 6.84 (m, 1 H) 6.90 (s, 4 H) 6.98 - 7.05 (m, 1 H) 7.16 - 7.24 (m, 1 H) 7.66 - 7.80 (m, 1 H)

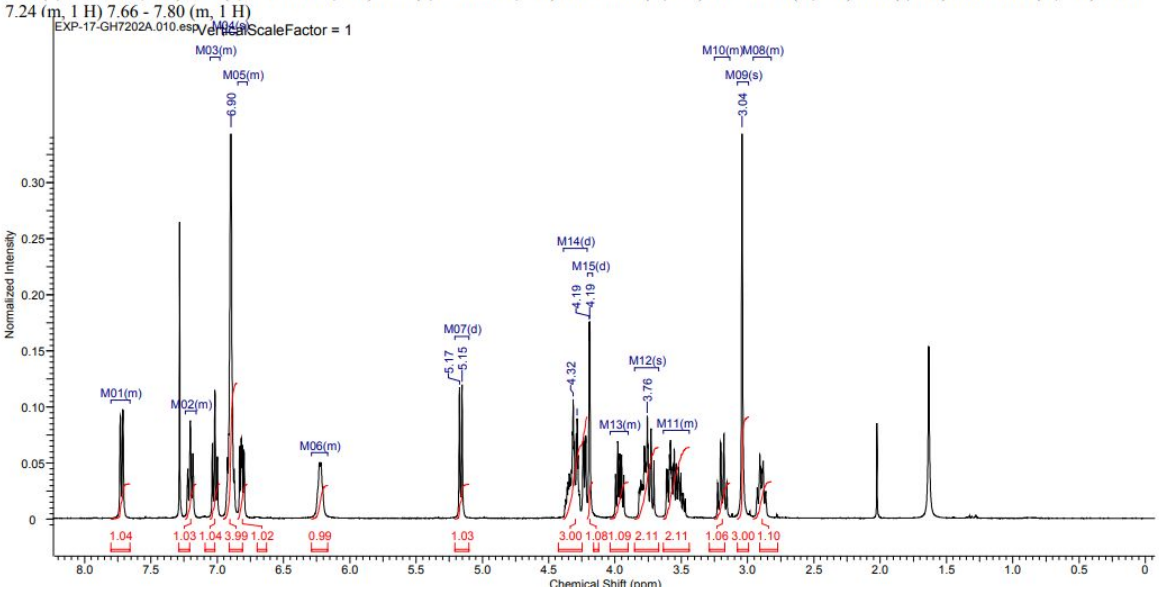

LC-MS Spectra for Compound 49

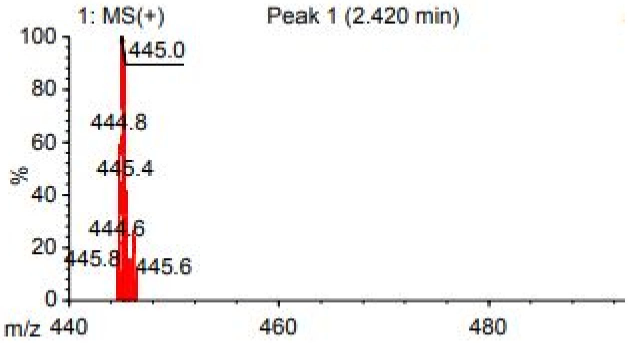

^{1}H NMR Spectra for Compound 50

^{1}H NMR (400 MHz, CHLOROFORM-*d*) δ ppm 2.12 (s, 3 H) 2.73 - 2.88 (m, 1 H) 3.15 - 3.32 (m, 1 H) 3.45 - 3.59 (m, 1 H) 3.62 - 3.72 (m, 1 H) 3.86 (d, *J*=10.04 Hz, 2 H) 3.92 - 4.03 (m, 1 H) 4.18 (d, *J*=1.25 Hz, 1 H) 4.33 (s, 3 H) 5.63 - 5.75 (m, 1 H) 6.22 - 6.34 (m, 1 H) 6.75 - 6.83 (m, 1 H) 6.85 - 6.98 (m, 5 H) 7.12 - 7.20 (m, 1 H) 7.64 - 7.75 (m, 1 H)

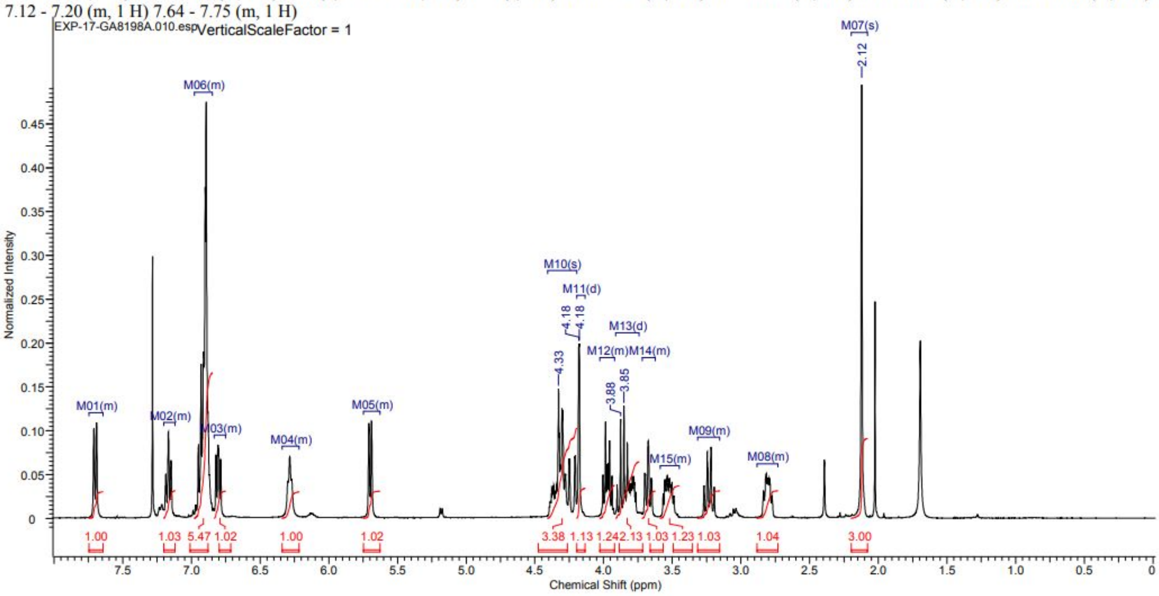

LC-MS Spectra for Compound 50

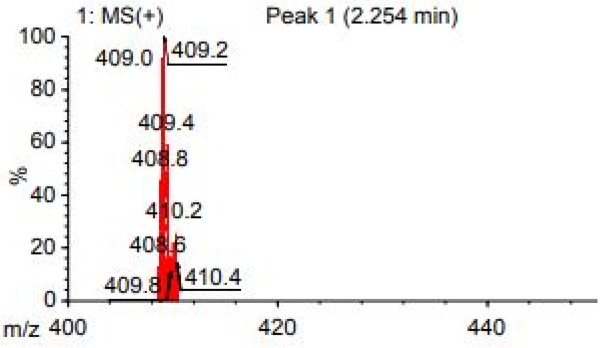

^{1}H NMR Spectra for Compound 51

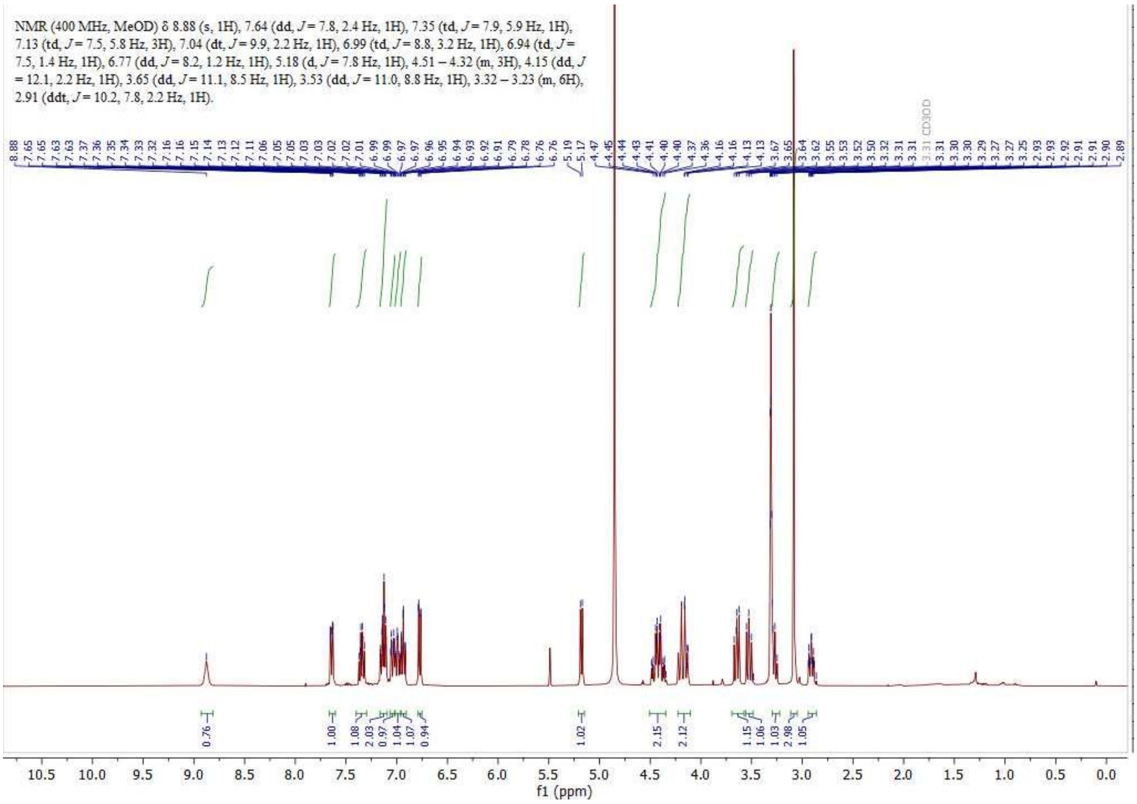

LC-MS Spectra for Compound 51

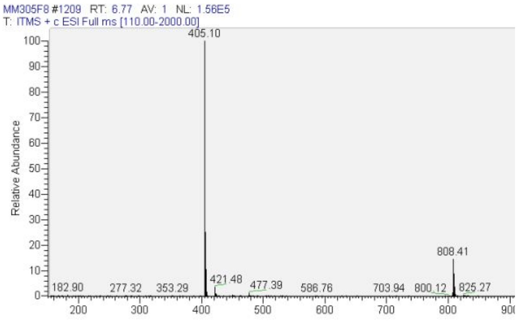

^{1}H NMR Spectra for Compound 52

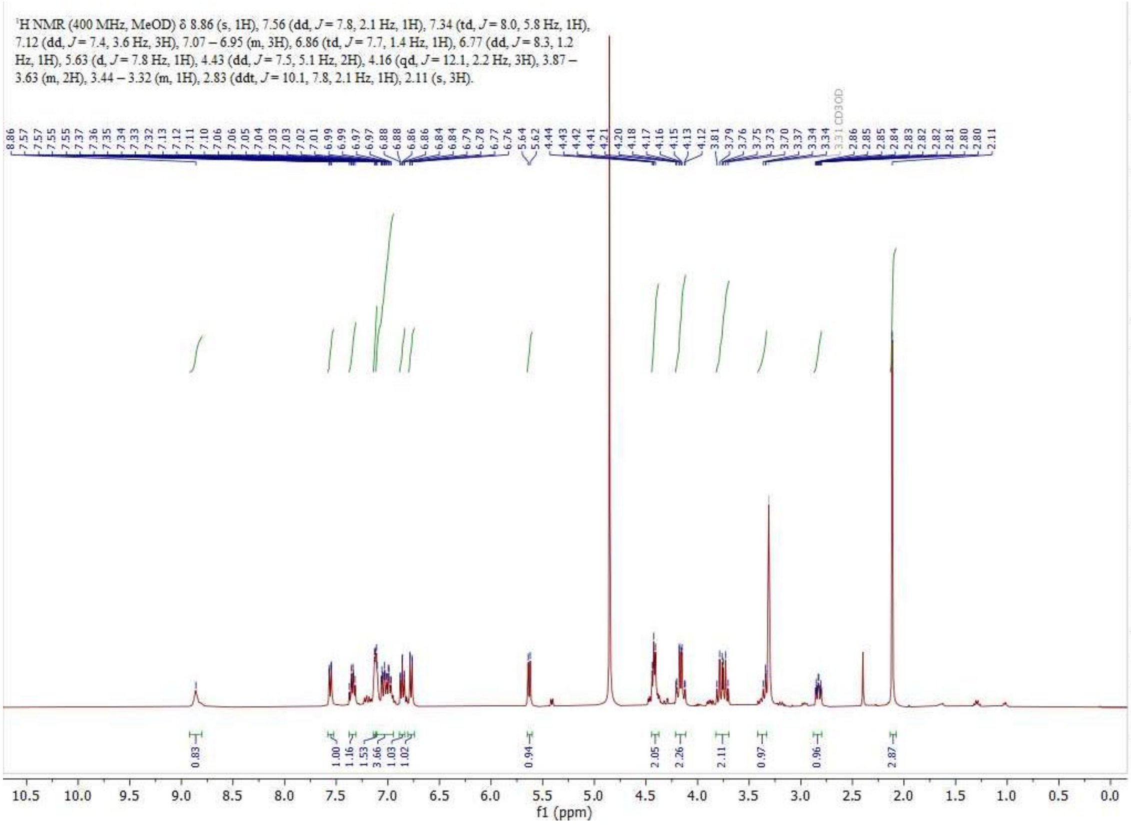

LC-MS Spectra for Compound 52

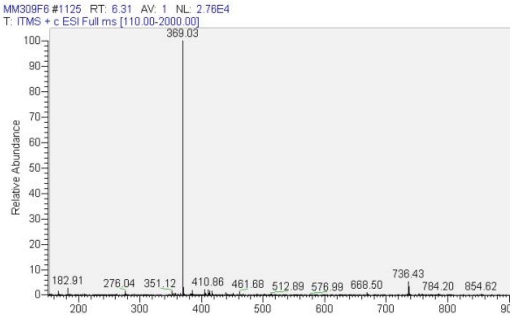

^{1}H NMR Spectra for Compound 53

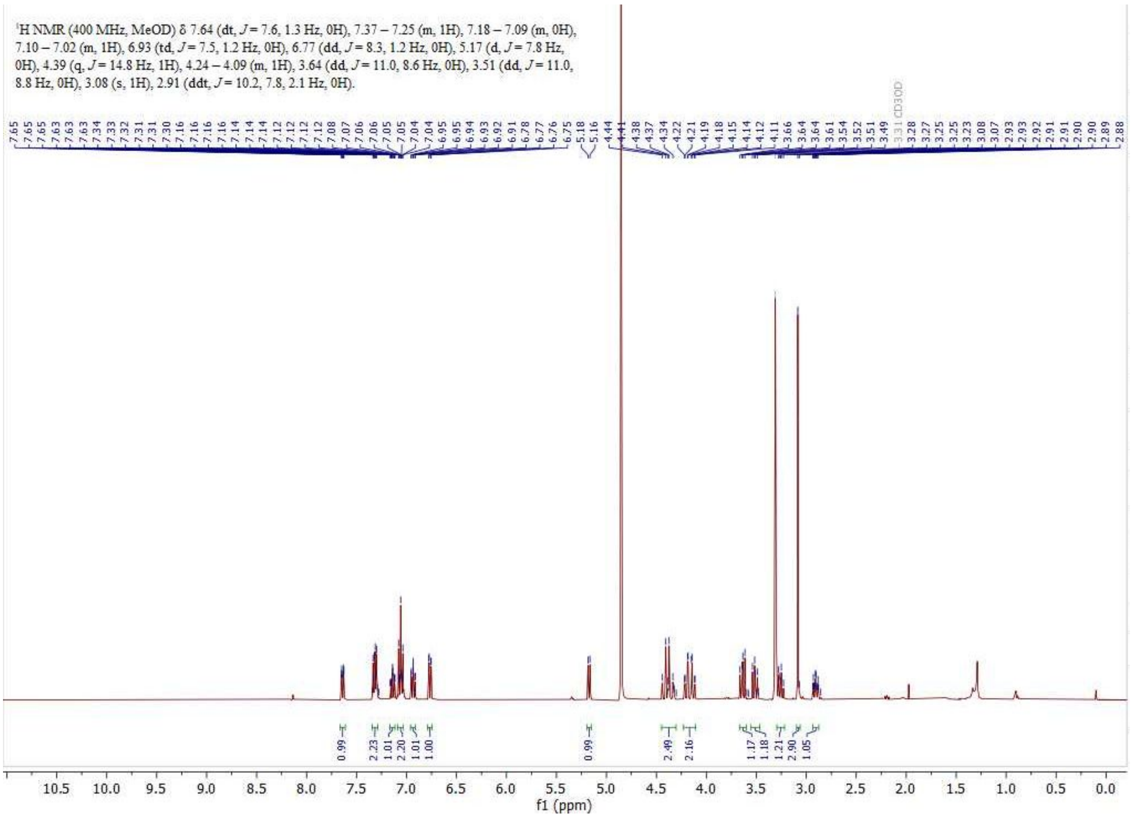

LC-MS Spectra for Compound 53

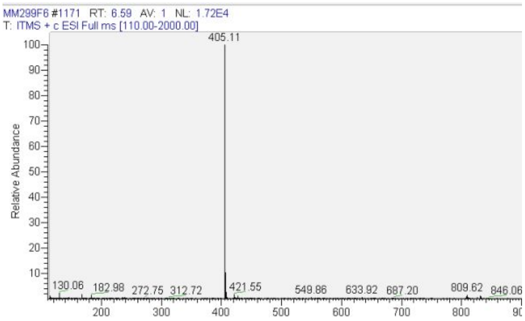

^{1}H NMR Spectra for Compound 54

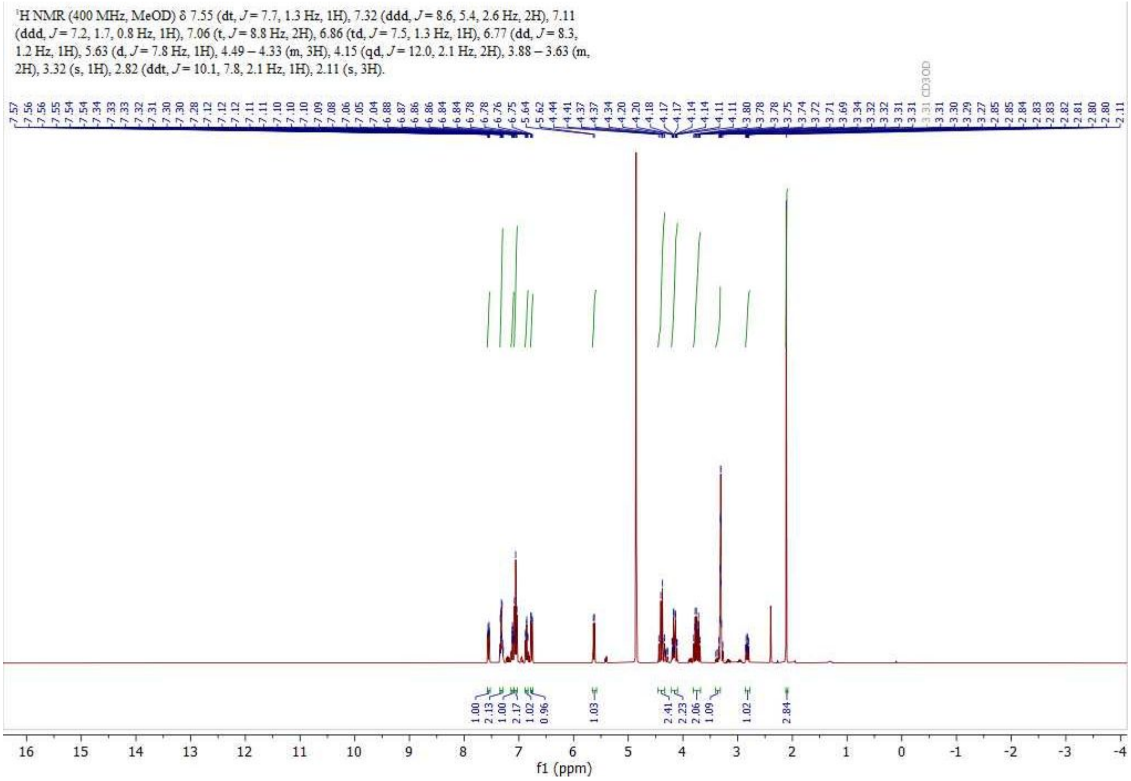

LC-MS Spectra for Compound 54

#

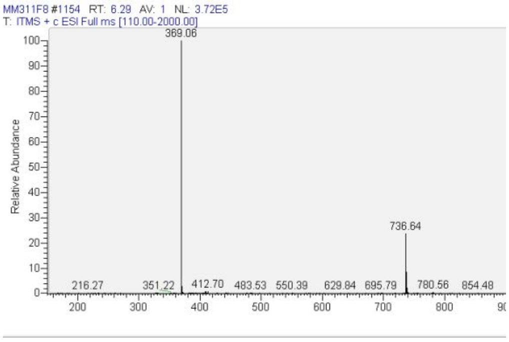

1H NMR Spectra for Compound 55

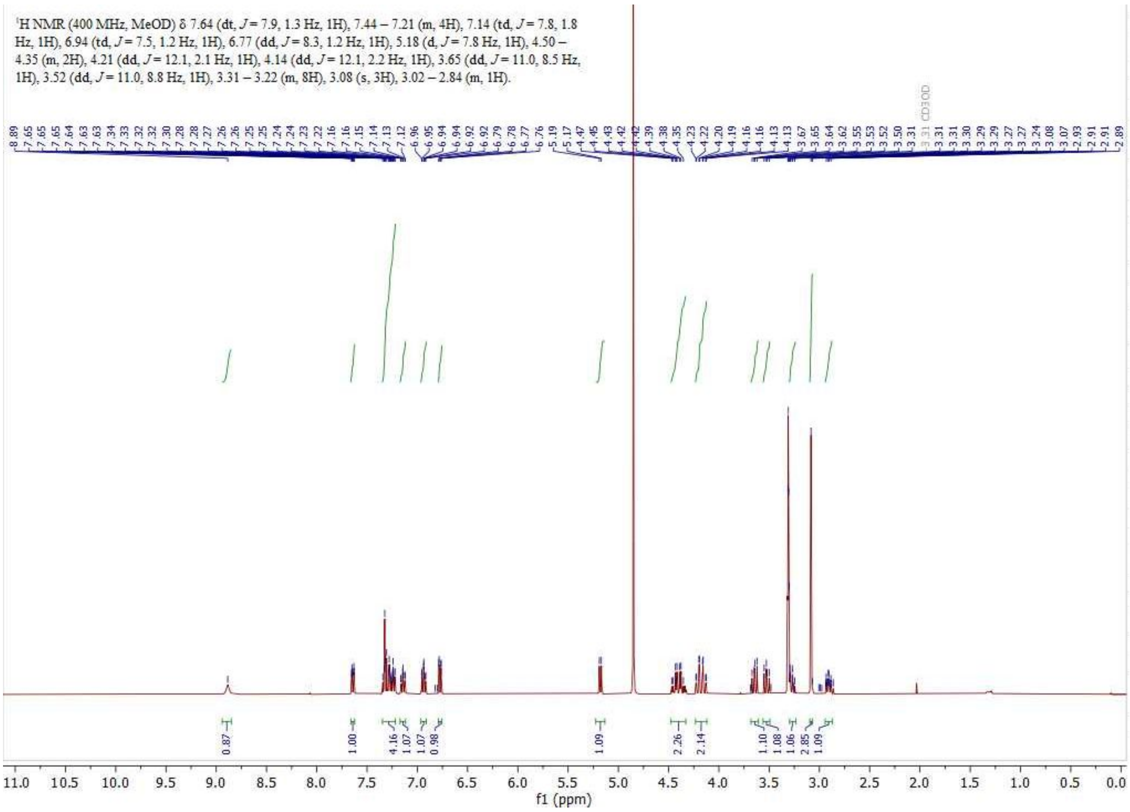

LC-MS Spectra for Compound 55

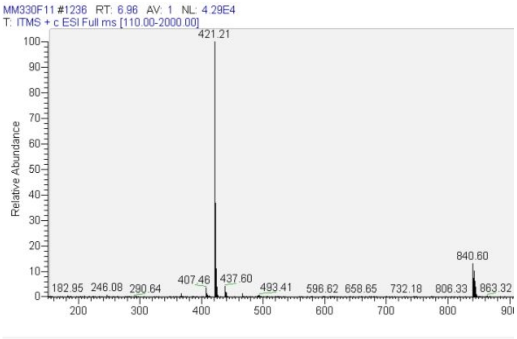

^{1}H NMR Spectra for Compound 56

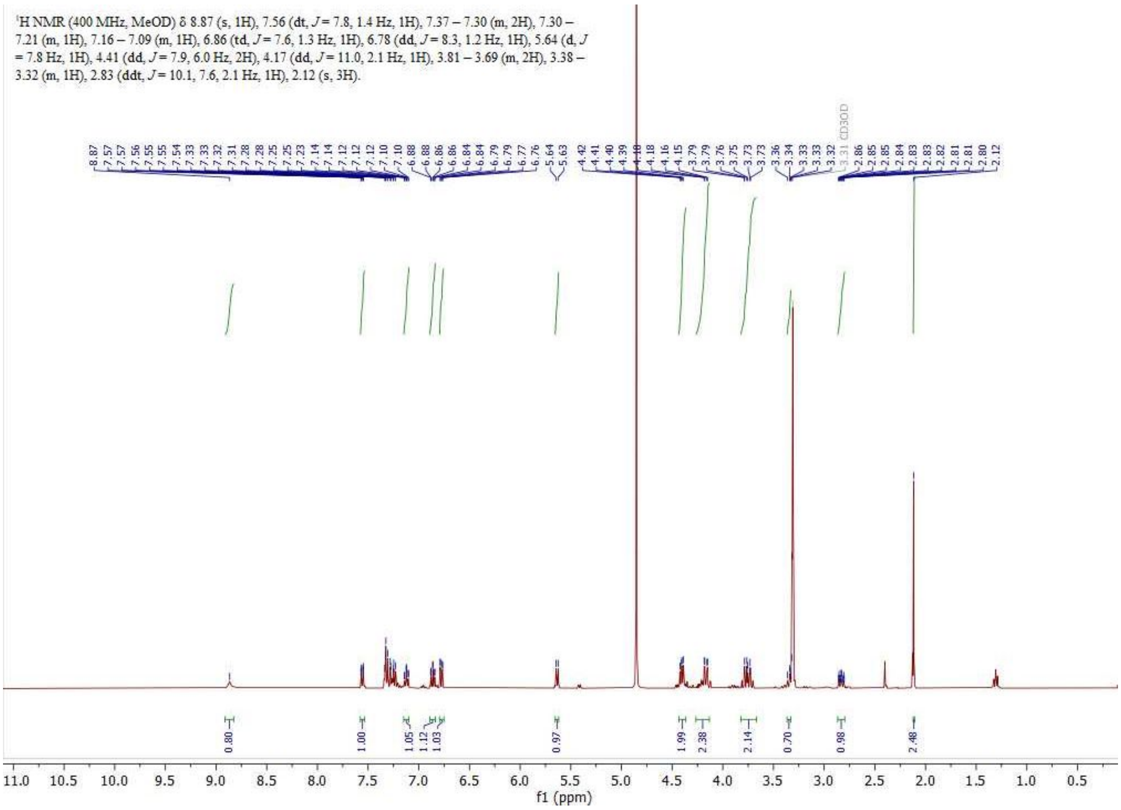

LC-MS Spectra for Compound 56

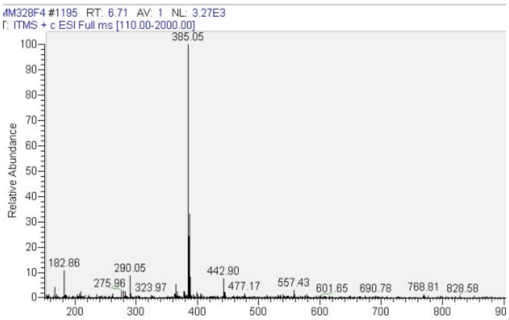

1H NMR Spectra for Compound 57

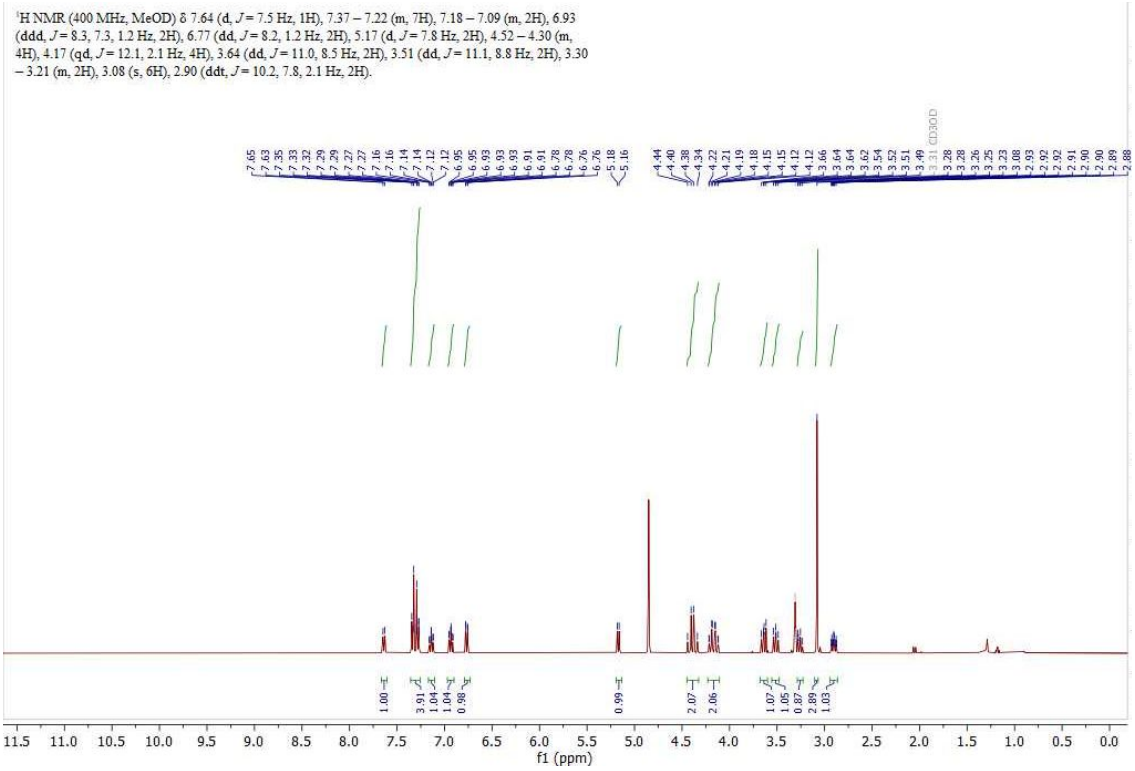

LC-MS Spectra for Compound 57

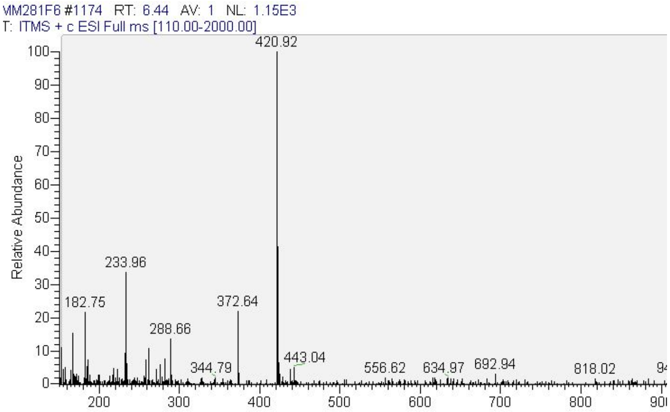

^{1}H NMR Spectra for Compound 58

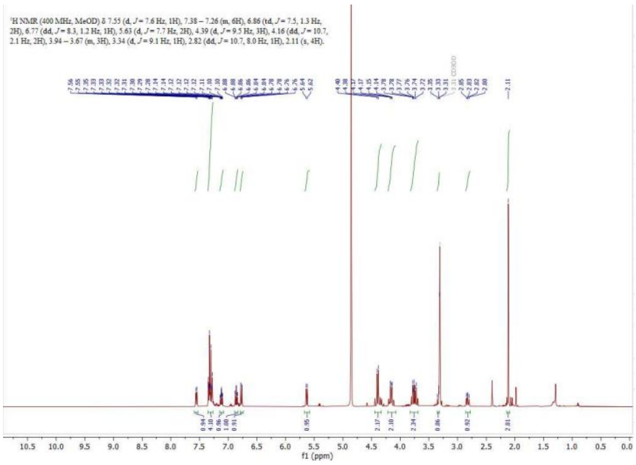

LC-MS Spectra for Compound 58

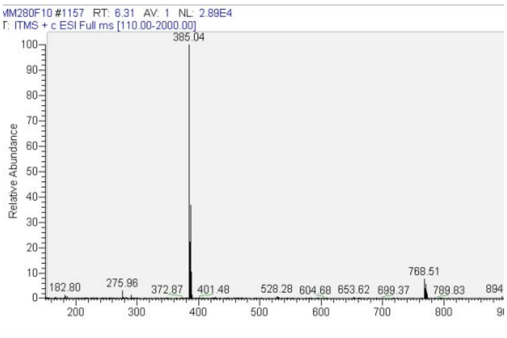

^{1}H NMR Spectra for Compound 59

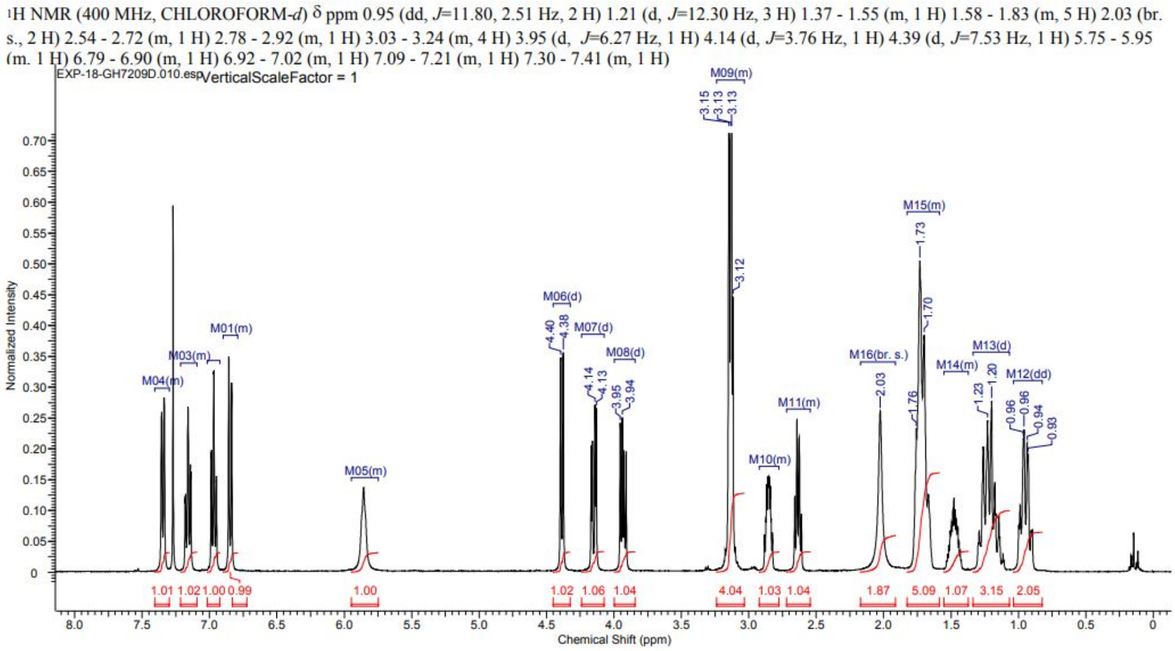

LC-MS Spectra for Compound 59

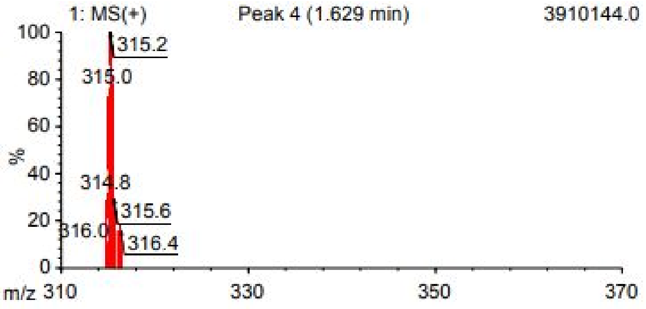

^{1}H NMR Spectra for Compound 60

^{1}H NMR (400 MHz, CHLOROFORM-*d*) δ ppm 0.86 - 1.05 (m, 2 H) 1.11 - 1.33 (m, 3 H) 1.41 - 1.57 (m, 1 H) 1.73 (d, *J*=11.29 Hz, 5 H) 2.49 (s, 3 H) 2.55 - 2.64 (m, 1 H) 2.65 - 2.73 (m, 1 H) 2.80 - 2.89 (m, 1 H) 3.15 (s, 2 H) 3.20 - 3.28 (m, 1 H) 3.36 - 3.48 (m, 1 H) 3.94 - 4.10 (m, 2 H) 5.58 - 5.81 (m, 1 H) 6.88 - 7.00 (m, 2 H) 7.17 - 7.25 (m, 2 H)

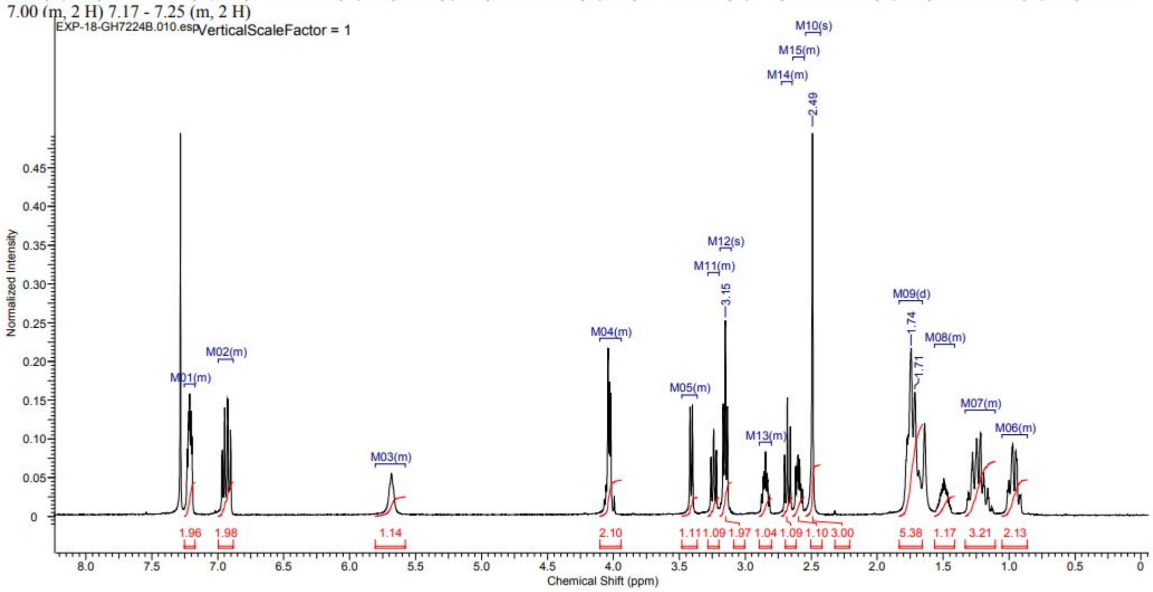

LC-MS Spectra for Compound 60

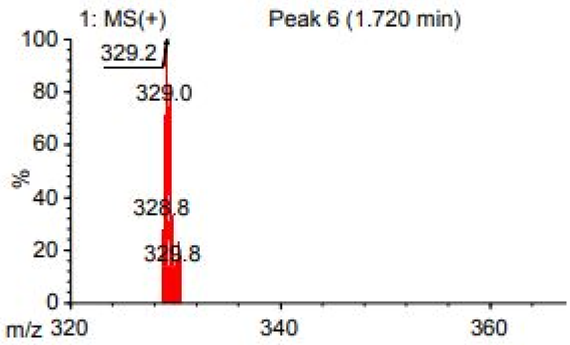

^{1}H NMR Spectra for Compound 61

^{1}H NMR (400 MHz, CHLOROFORM-*d*) δ ppm 0.81 - 1.01 (m, 2 H) 1.10 - 1.35 (m, 4 H) 1.38 - 1.53 (m, 1 H) 1.62 - 1.80 (m, 5 H) 2.51 - 2.63 (m, 2 H) 2.83 - 2.91 (m, 1 H) 2.92 - 3.02 (m, 1 H) 3.04 - 3.14 (m, 2 H) 3.42 - 3.52 (m, 1 H) 3.80 (s, 4 H) 4.01 - 4.09 (m, 1 H) 4.10 - 4.21 (m, 2 H) 5.70 - 5.89 (m, 1 H) 6.84 (d, *J*=8.53 Hz, 2 H) 6.88 - 6.99 (m, 2 H) 7.17 - 7.28 (m, 4 H)

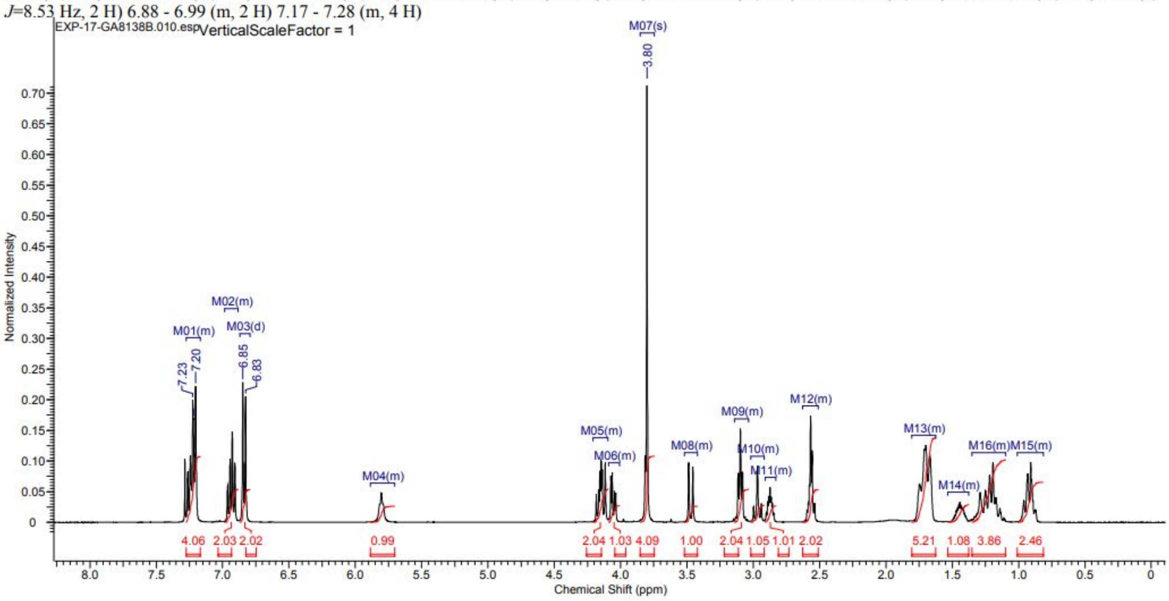

LC-MS Spectra for Compound 61

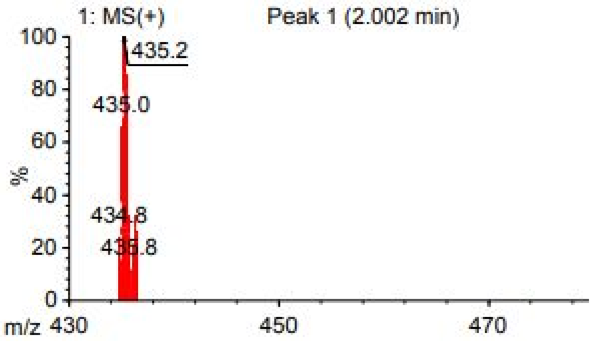

^{1}H NMR Spectra for Compound 62

^{1}H NMR (400 MHz, CHLOROFORM-*d*) δ ppm 0.87 - 1.04 (m, 2 H) 1.22 (s, 6 H) 1.40 - 1.56 (m, 1 H) 1.64 - 1.89 (m, 5 H) 2.15 - 2.33 (m, 1 H) 2.34 - 2.51 (m, 1 H) 2.73 - 2.87 (m, 1 H) 3.04 - 3.29 (m, 3 H) 3.55 - 3.72 (m, 1 H) 3.80 - 3.95 (m, 1 H) 4.20 (d, *J*=1.25 Hz, 2 H) 5.65 - 5.82 (m, 2 H) 6.75 - 6.87 (m, 1 H) 6.89 - 7.00 (m, 1 H) 7.12 - 7.24 (m, 1 H) 7.66 - 7.78 (m, 1 H)

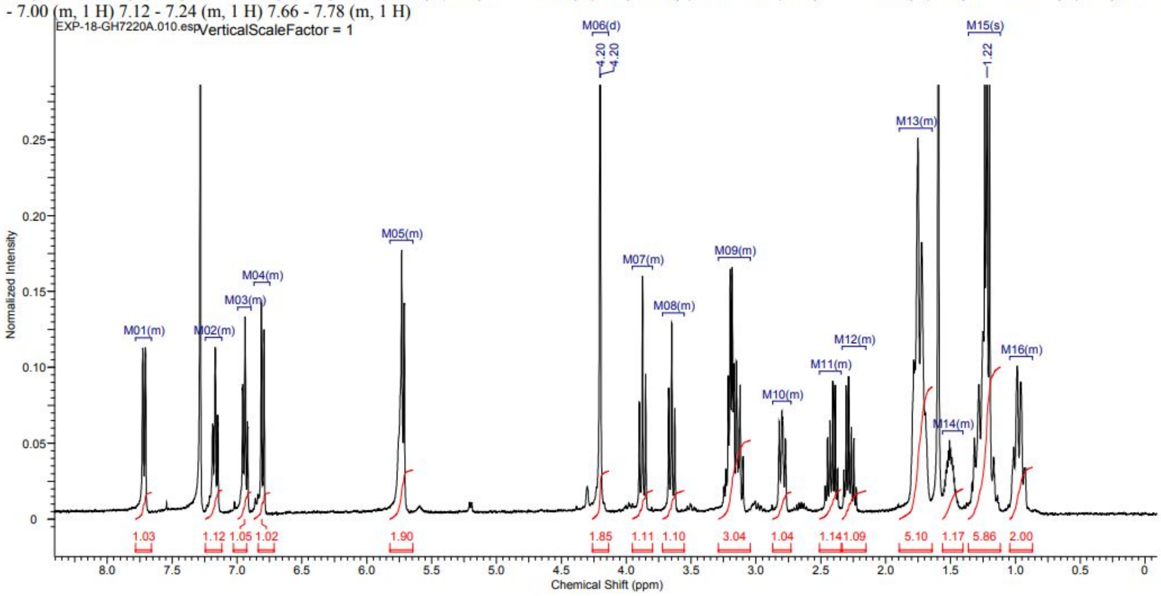

LC-MS Spectra for Compound 62

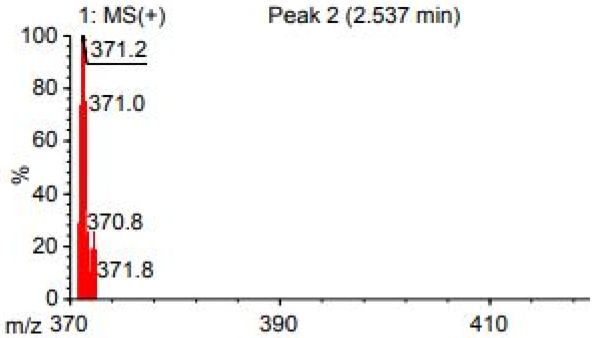

^{1}H NMR Spectra for Compound 63

^{1}H NMR (400 MHz, CHLOROFORM-*d*) δ ppm 0.74 - 1.37 (m, 9 H) 1.43 - 1.56 (m, 1 H) 1.62 - 1.84 (m, 5 H) 2.75 - 3.05 (m, 1 H) 3.21 (br. s., 3 H) 3.49 - 3.89 (m, 1 H) 3.90 - 4.14 (m, 1 H) 4.21 (d, *J*=1.51 Hz, 2 H) 5.41 - 5.86 (m, 2 H) 6.75 - 6.88 (m, 1 H) 6.89 - 7.02 (m, 1 H) 7.08 - 7.24 (m, 1 H) 7.67 (d, *J*=7.78 Hz, 1 H)

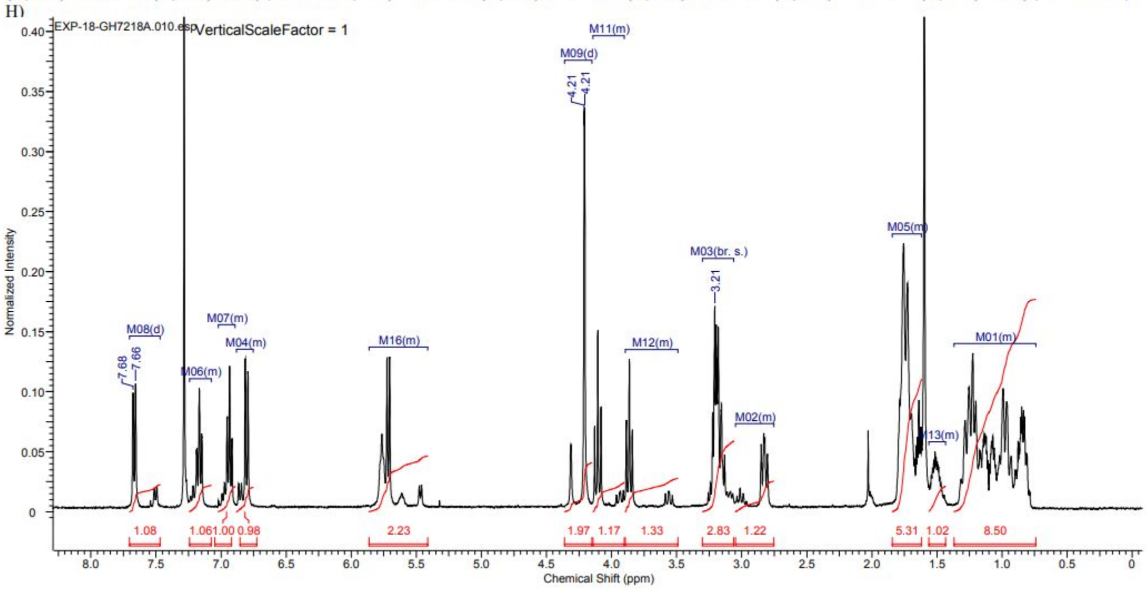

LC-MS Spectra for Compound 63

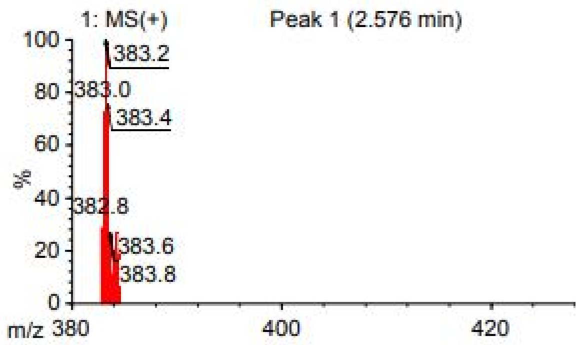

^{1}H NMR Spectra for Compound 64

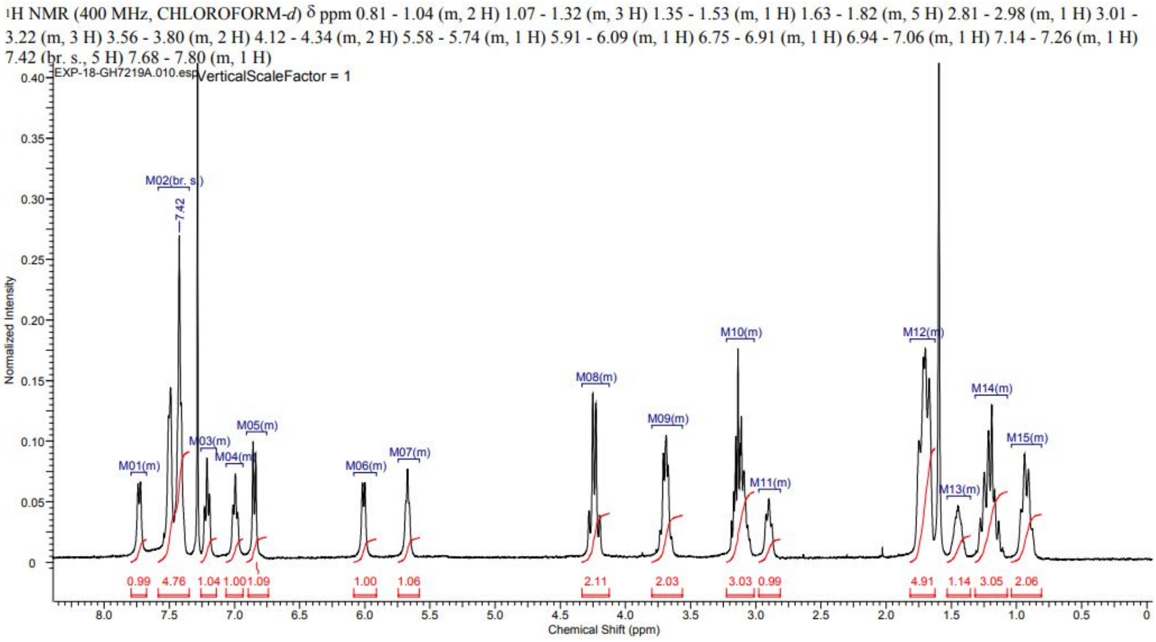

LC-MS Spectra for Compound 64

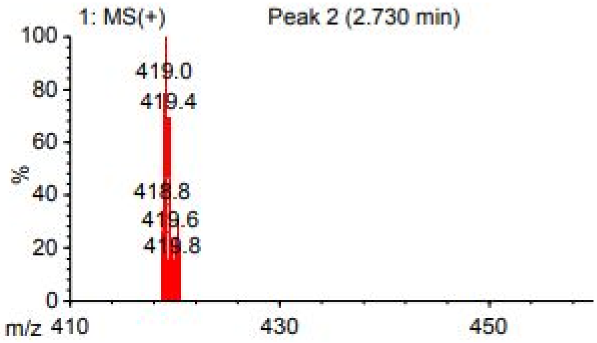

^{1}H NMR Spectra for Compound 65

¹H NMR (400 MHz, CHLOROFORM-*d*) δ ppm 0.88 - 1.02 (m, 2 H) 1.03 - 1.40 (m, 7 H) 1.42 - 1.56 (m, 1 H) 1.65 - 1.82 (m, 5 H) 2.59 - 2.75 (m, 1 H) 2.98 - 3.06 (m, 1 H) 3.07 - 3.14 (m, 1 H) 3.17 (t, *J*=6.40 Hz, 2 H) 3.48 - 3.64 (m, 1 H) 3.83 - 3.95 (m, 1 H) 4.23 (s, 2 H) 5.20 (d, *J*=7.53 Hz, 1 H) 5.64 - 5.85 (m, 1 H) 6.74 - 6.84 (m, 1 H) 6.97 - 7.05 (m, 1 H) 7.13 - 7.23 (m, 1 H) 7.65 - 7.78 (m, 1 H)

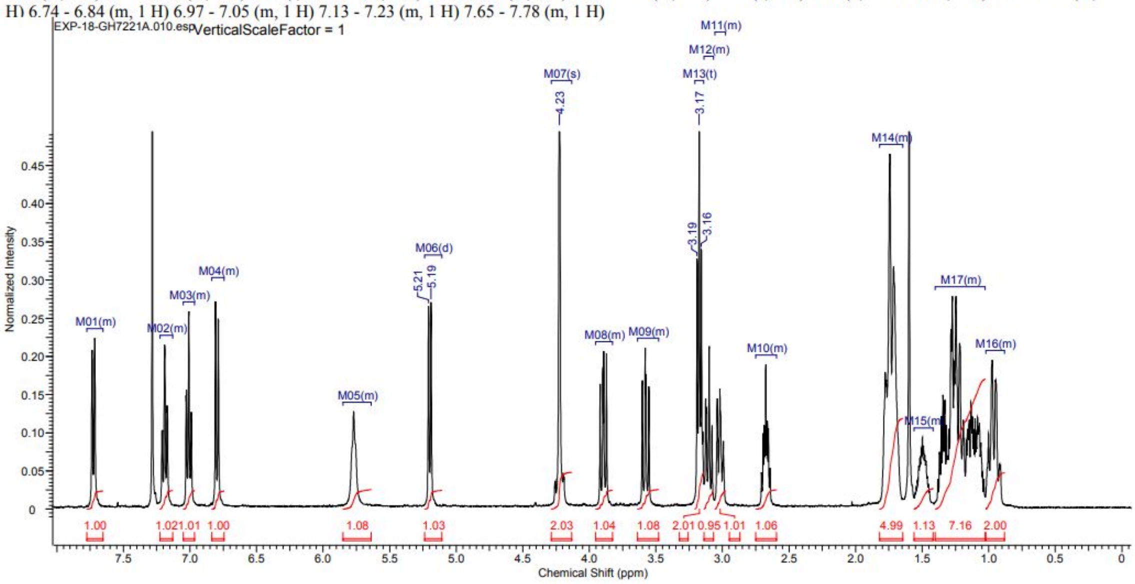

LC-MS Spectra for Compound 65

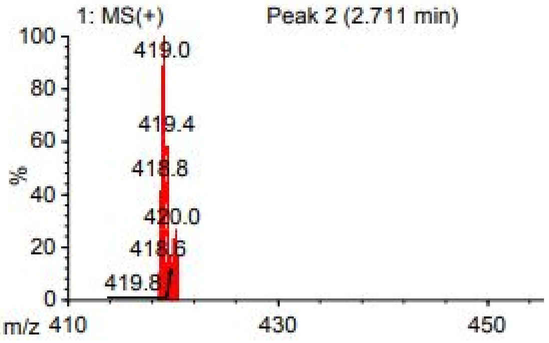

¹H NMR Spectra for Compound 66

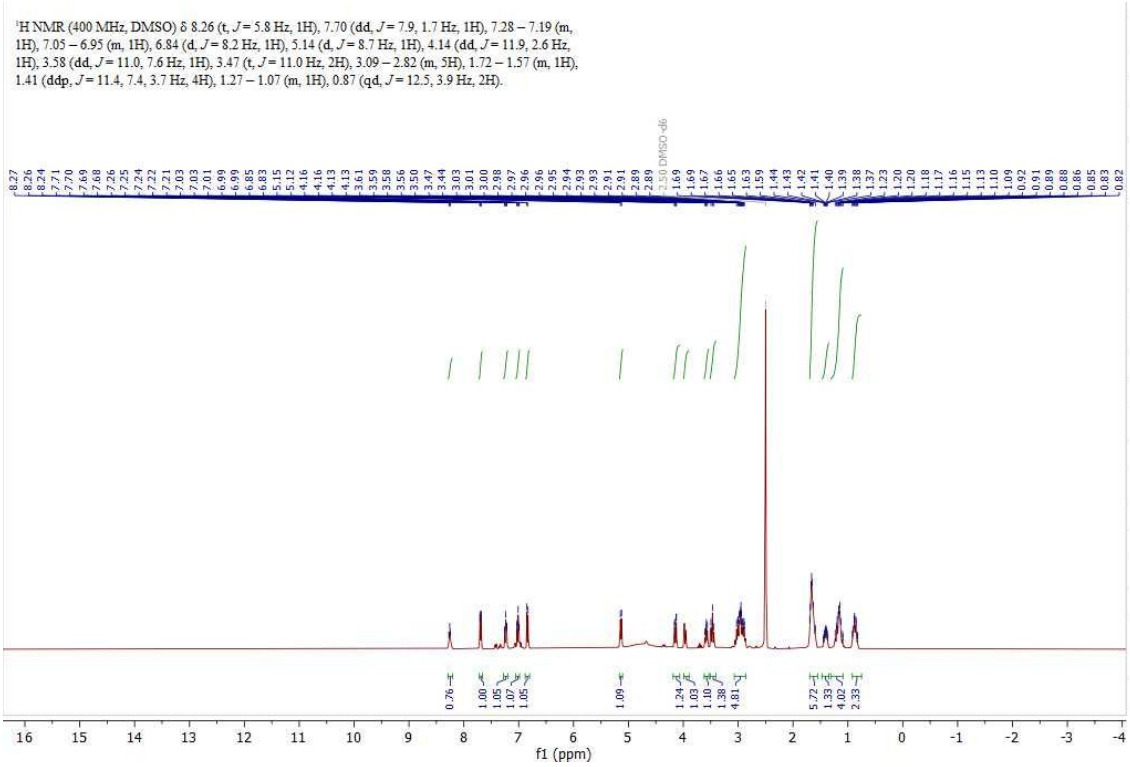

LC-MS Spectra for Compound 66

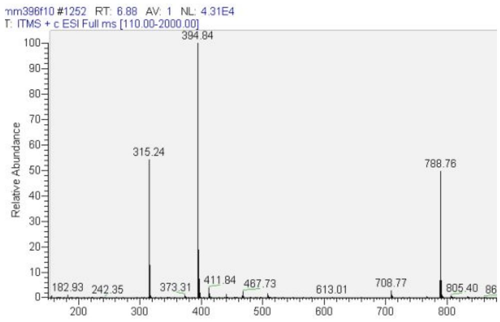

1H NMR Spectra for Compound 67

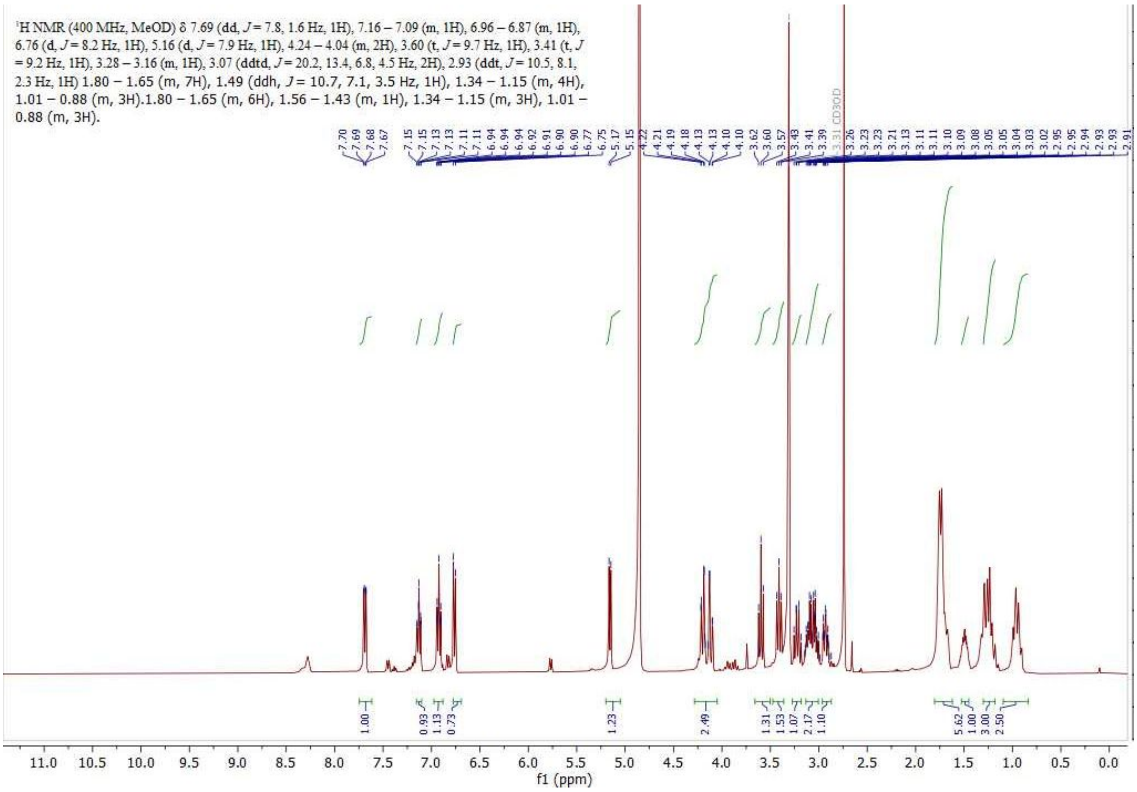

LC-MS Spectra for Compound 67

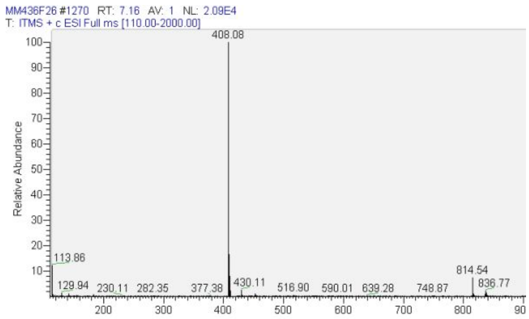

1H NMR Spectra for Compound 68

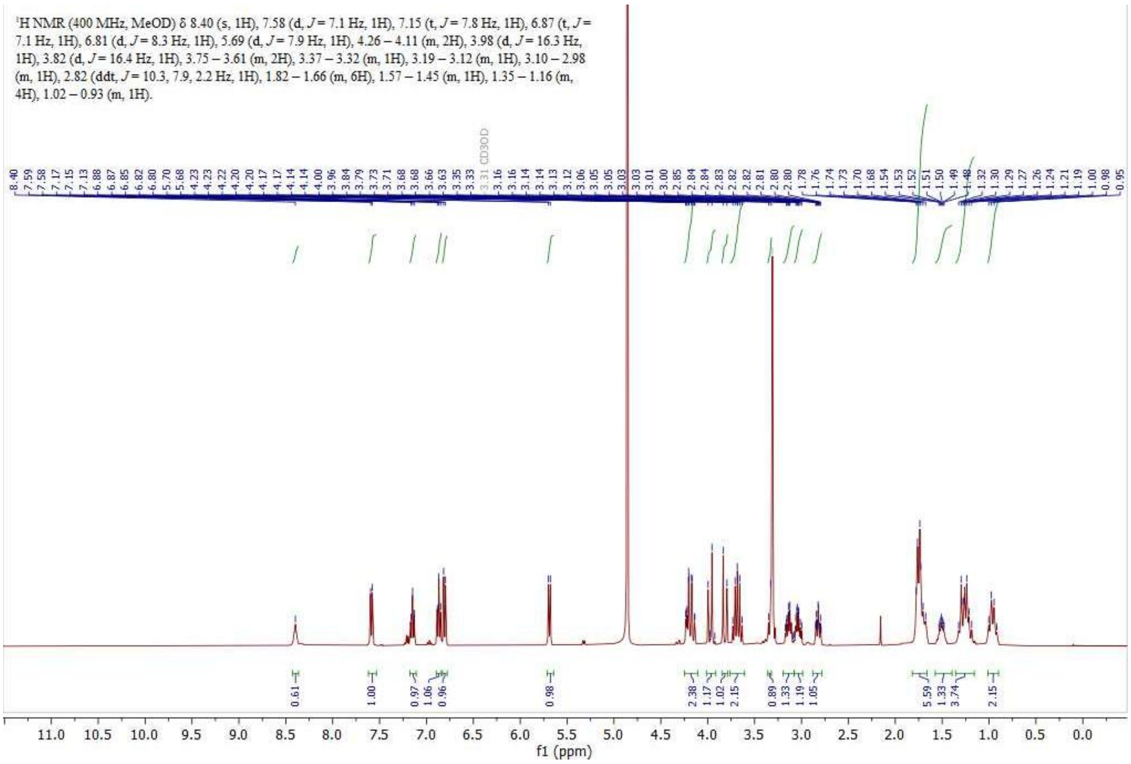

LC-MS Spectra for Compound 68

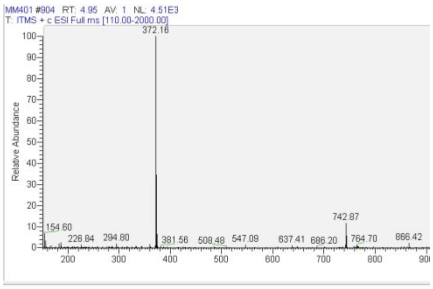

1H NMR Spectra for Compound 69

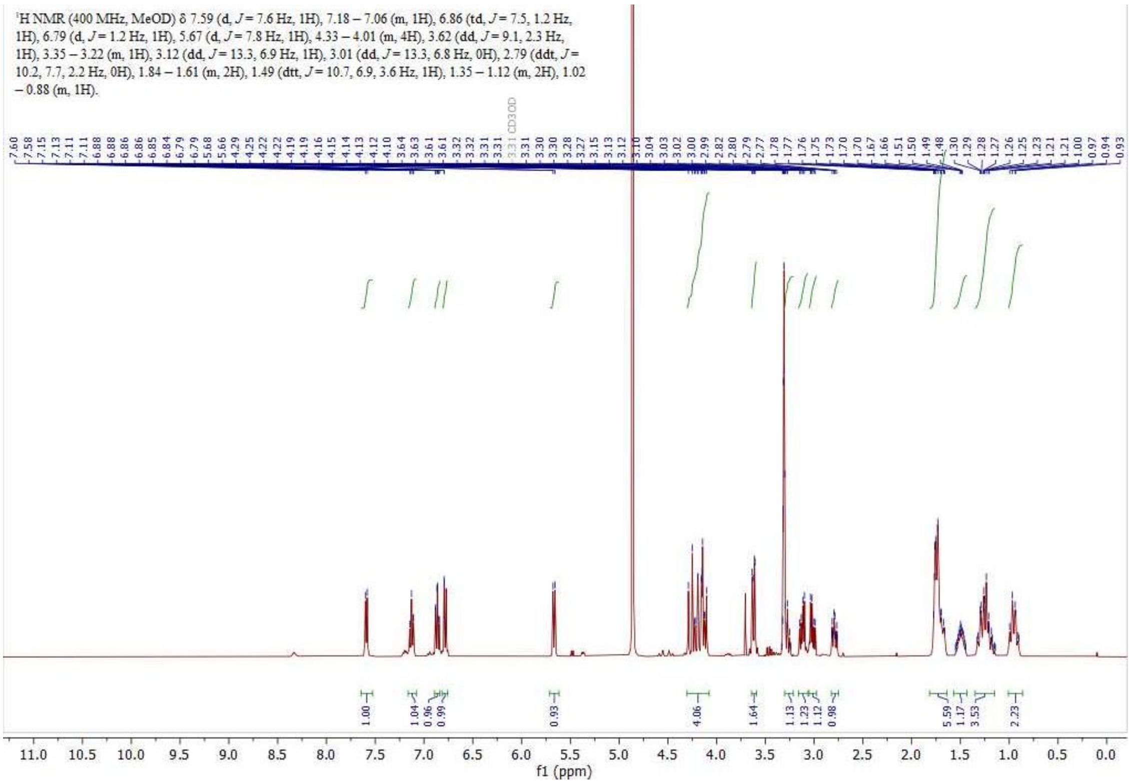

LC-MS Spectra for Compound 69

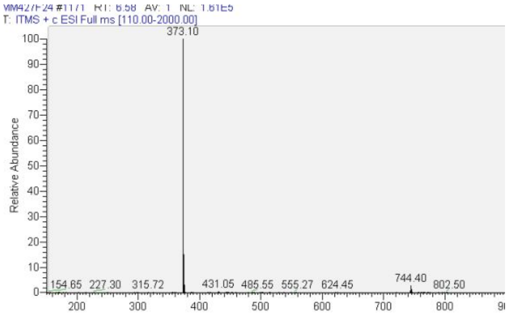

^{1}H NMR Spectra for Compound 70

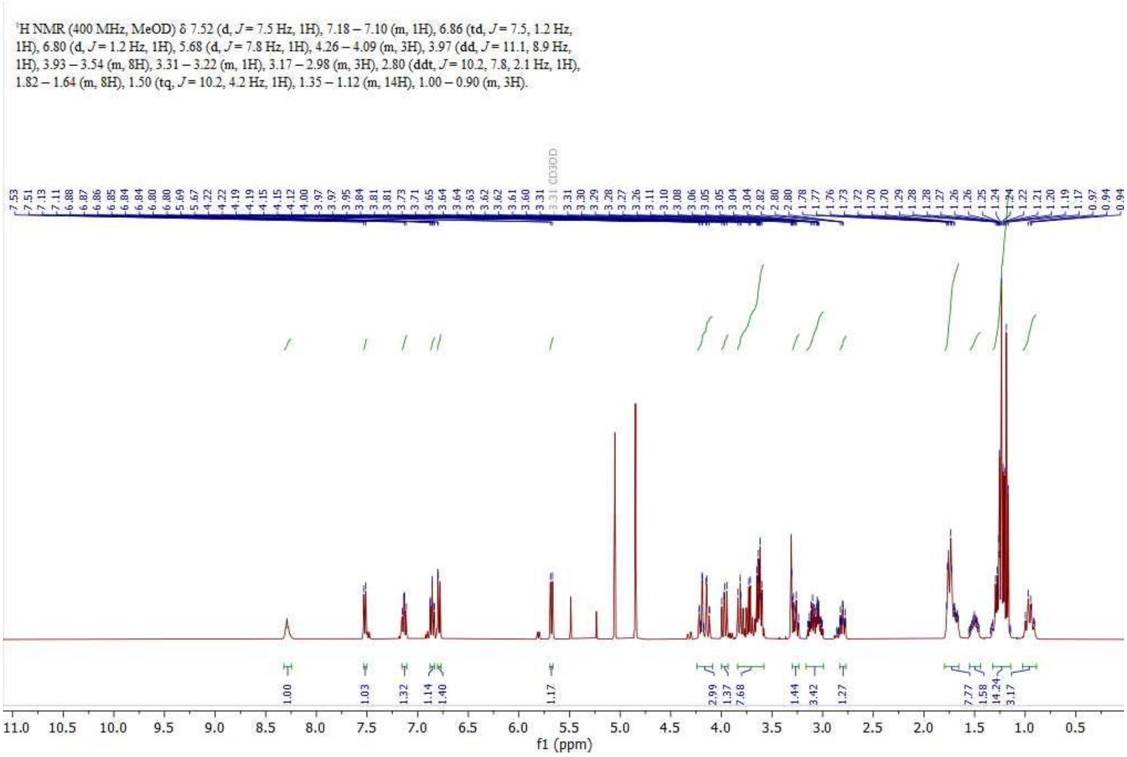

LC-MS Spectra for Compound 70

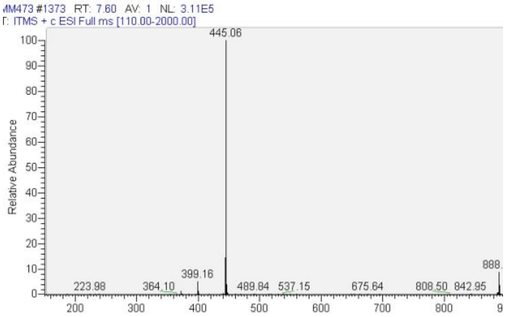

^{1}H NMR Spectra for Compound 71

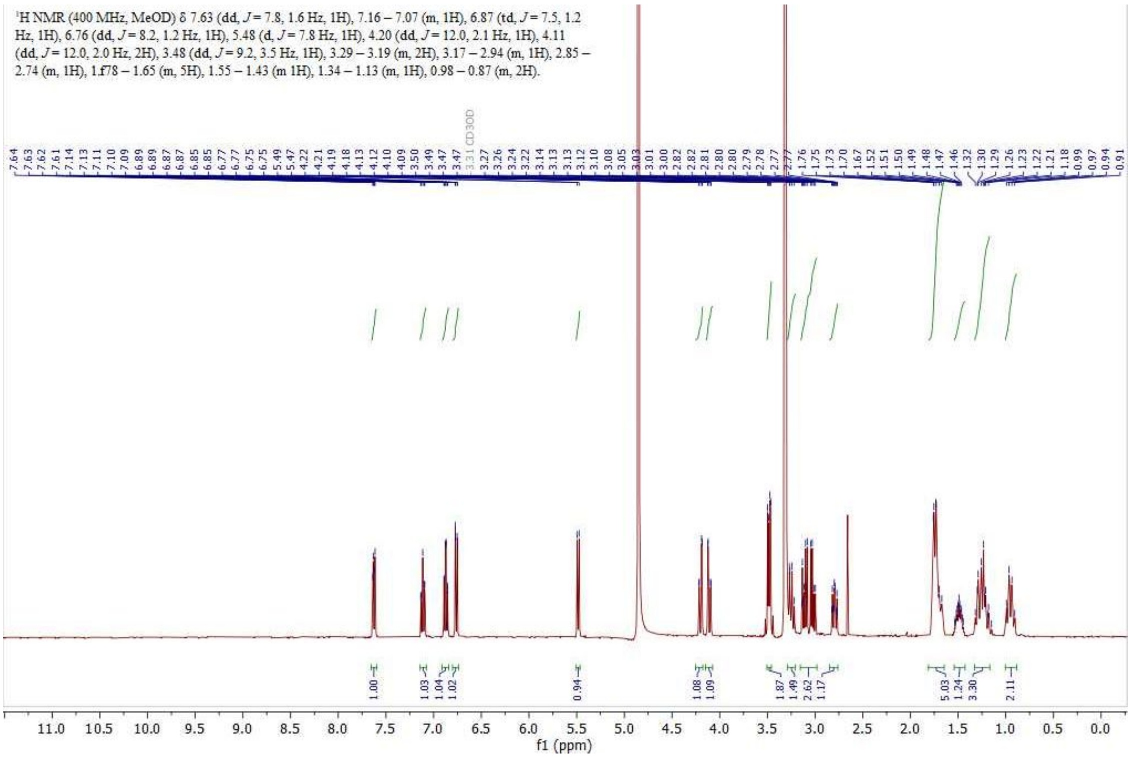

LC-MS Spectra for Compound 71

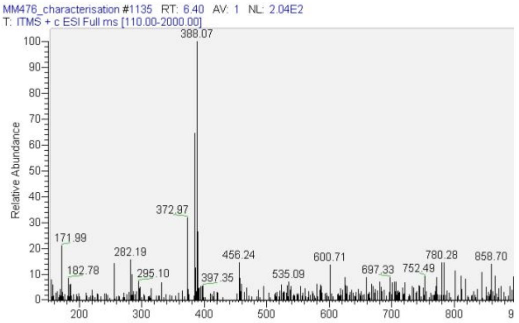

1H NMR Spectra for Compound 72

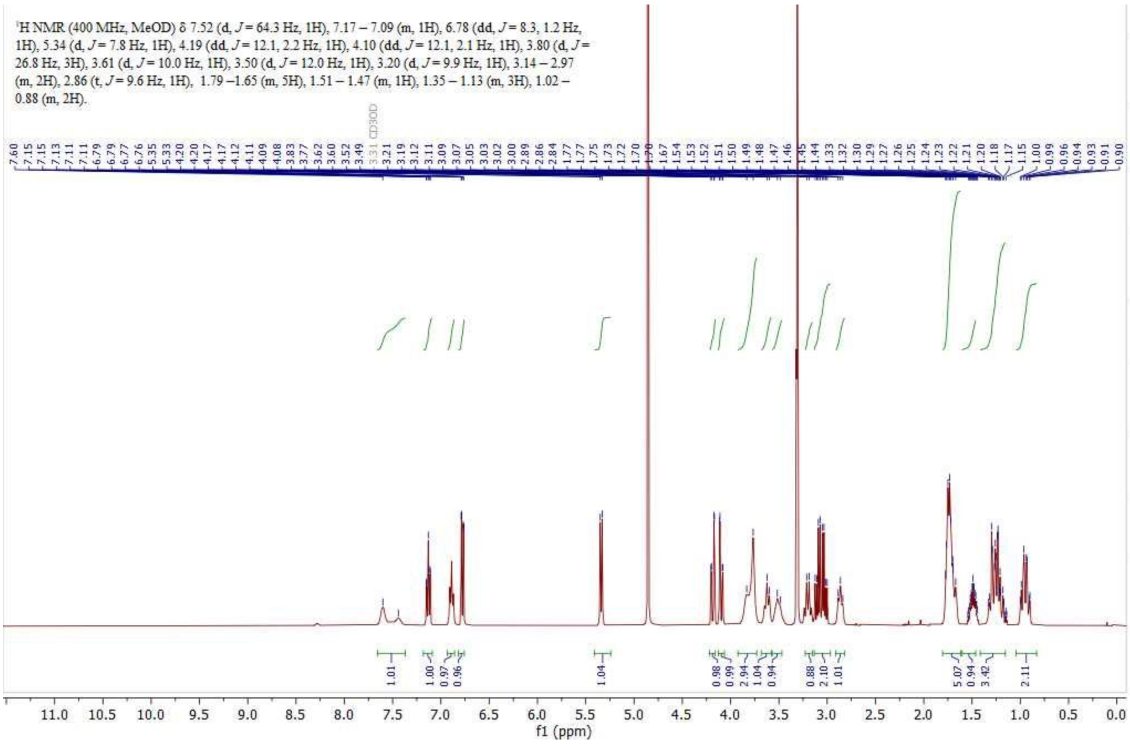

LC-MS Spectra for Compound 72

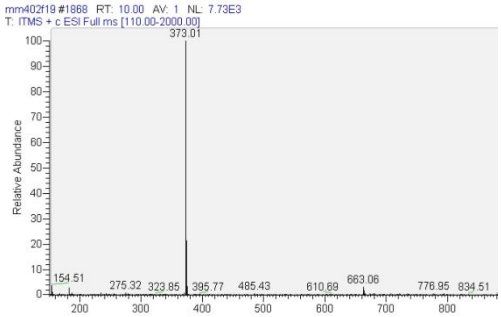

1H NMR Spectra for Compound 73

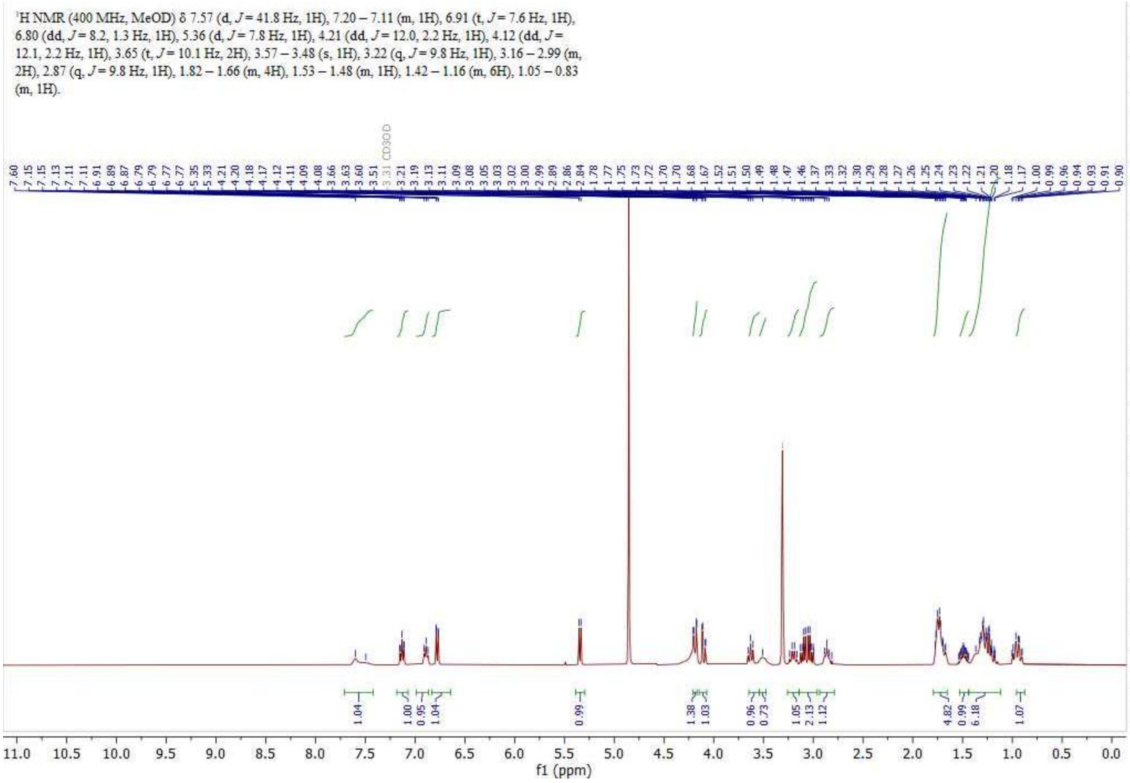

LC-MS Spectra for Compound 73

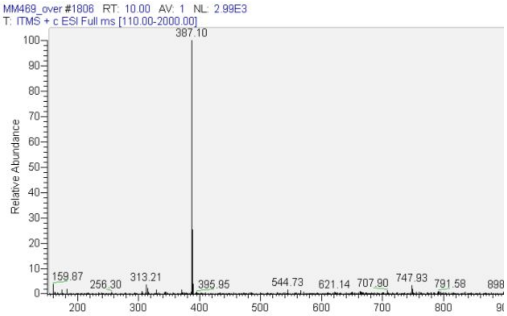

^{1}H NMR Spectra for Compound 74

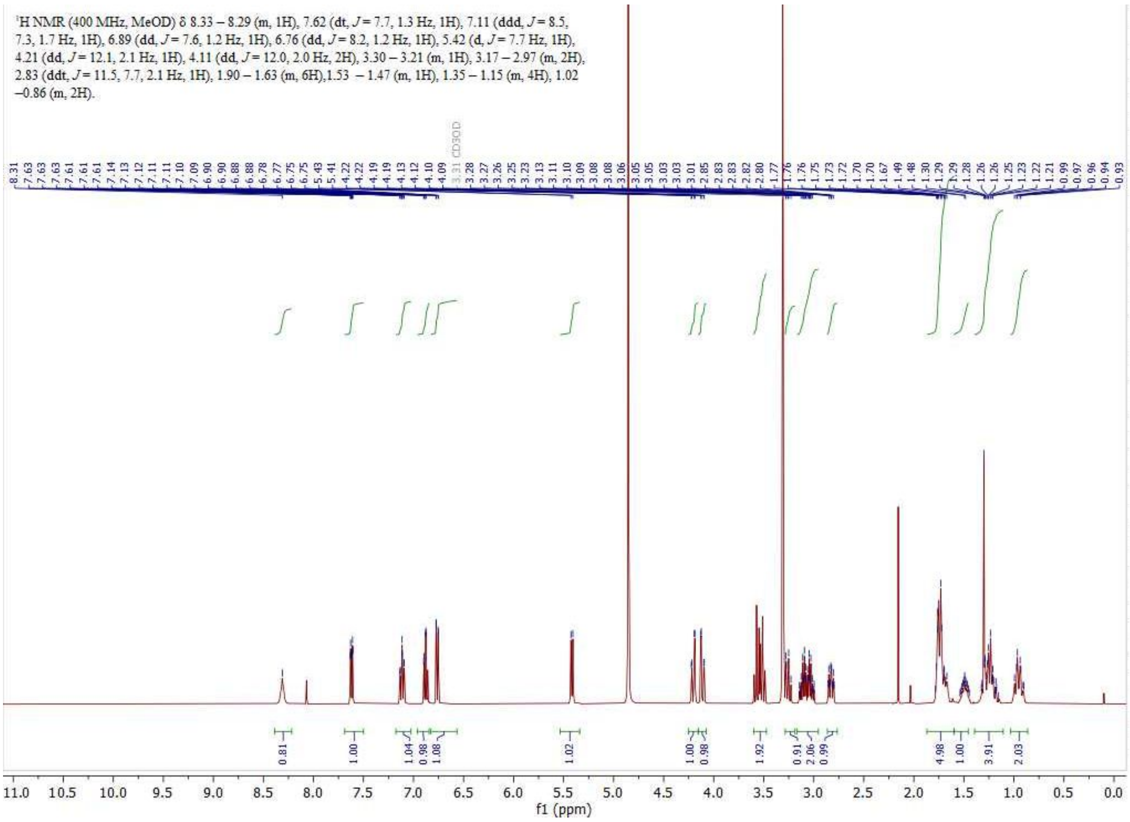

LC-MS Spectra for Compound 74

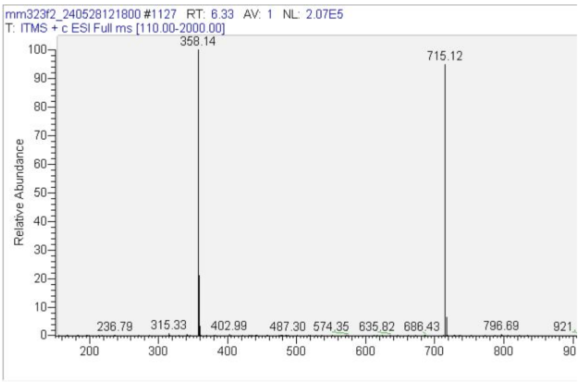

^{1}H NMR Spectra for Compound 75

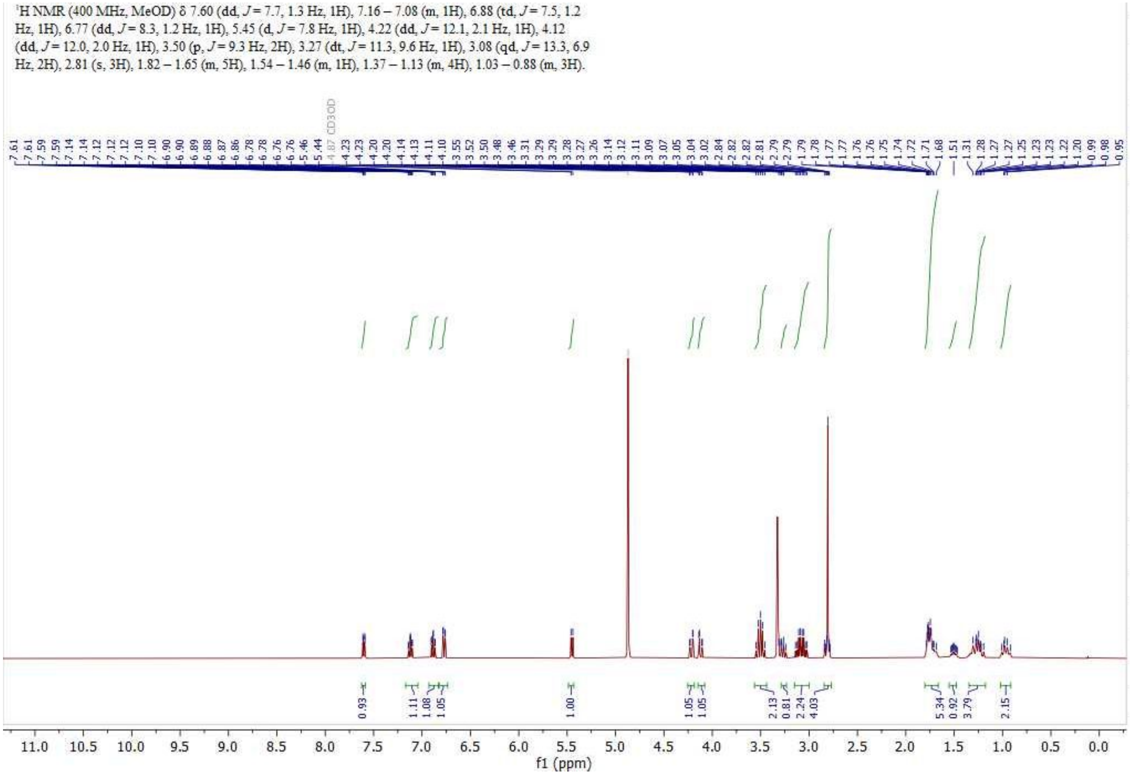

LC-MS Spectra for Compound 75

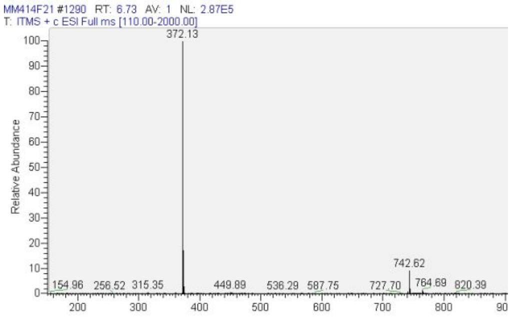

^{1}H NMR Spectra for Compound 76

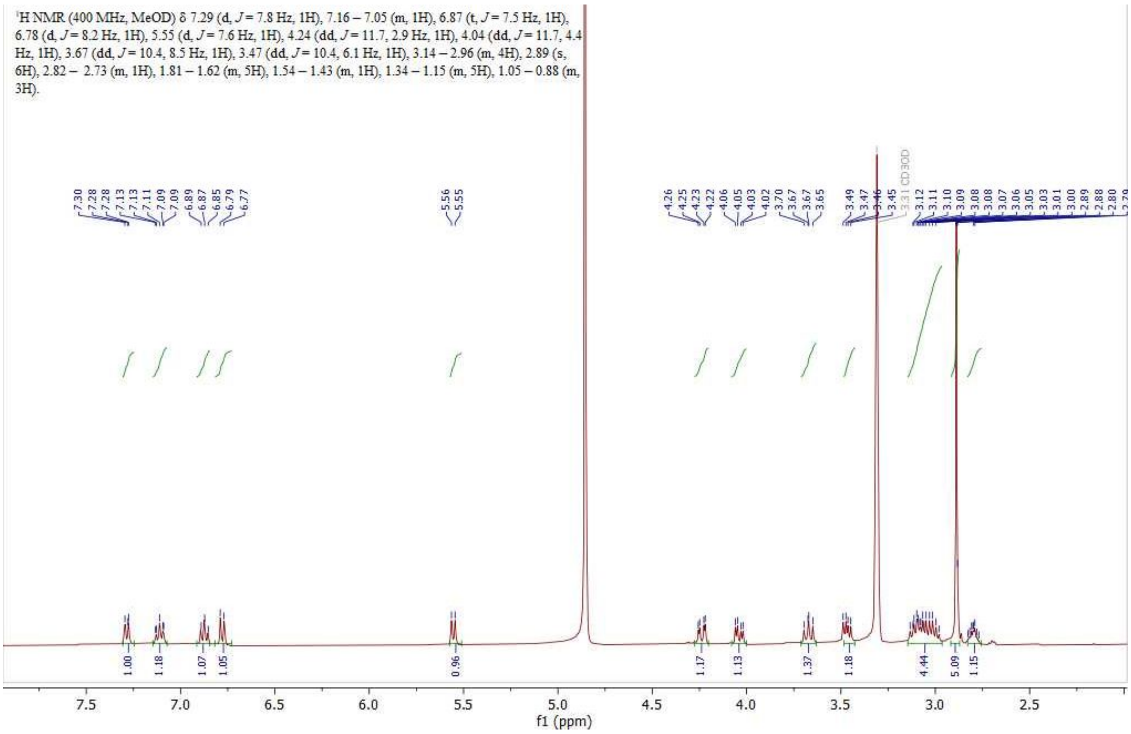

LC-MS Spectra for Compound 76

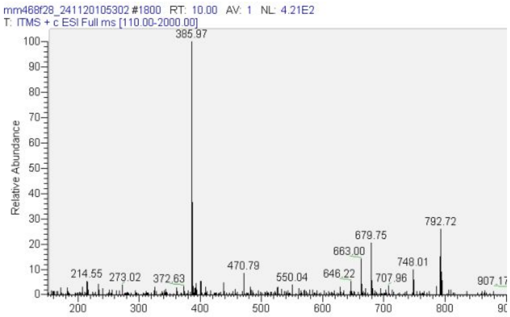

1H NMR Spectra for Compound 77

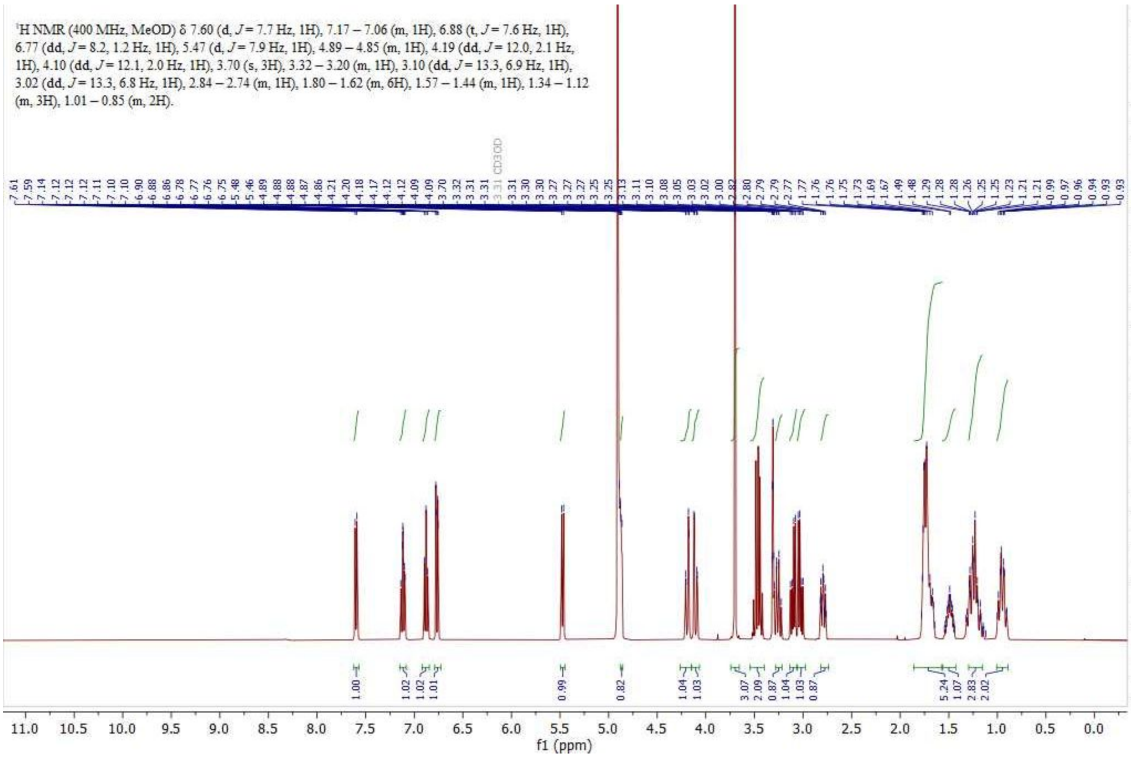

LC-MS Spectra for Compound 77

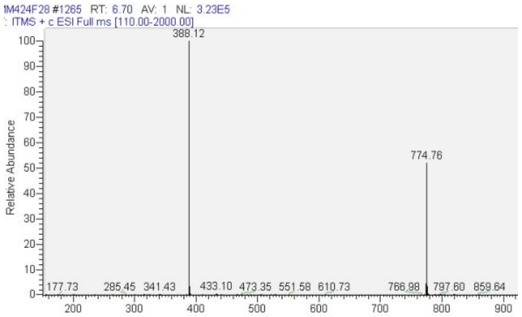

1H NMR Spectra for Compound 78

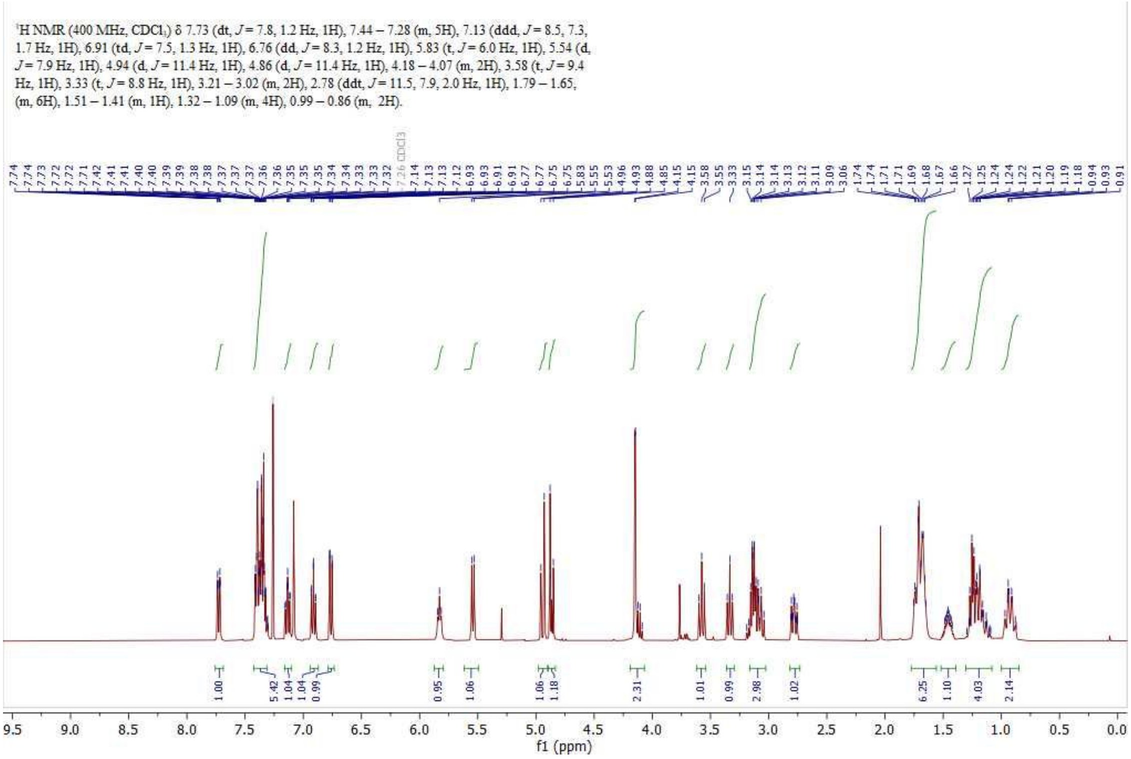

LC-MS Spectra for Compound 78

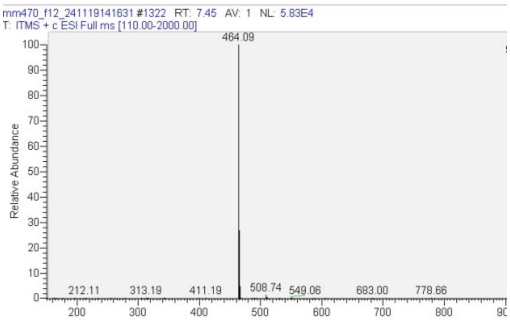

^{1}H NMR Spectra for Compound 79

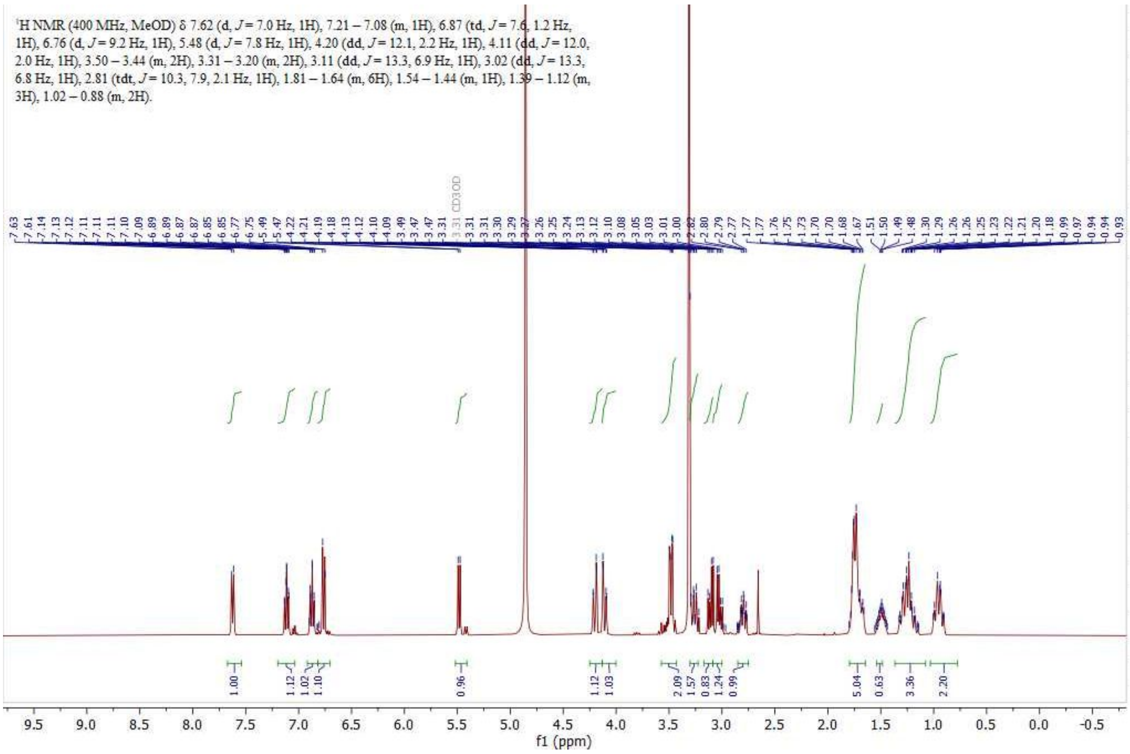

LC-MS Spectra for Compound 79

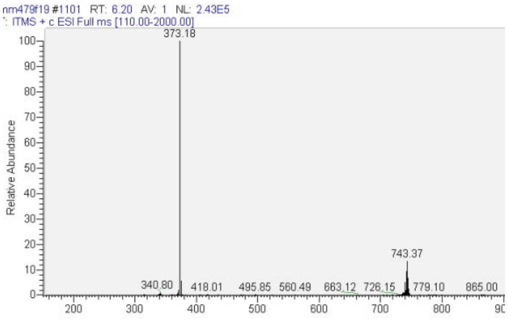

^{1}H NMR Spectra for Compound 80

¹H NMR (400 MHz, CHLOROFORM-*d*) δ ppm 0.86 - 1.06 (m, 2 H) 1.10 - 1.34 (m, 3 H) 1.41 - 1.56 (m, 1 H) 1.63 - 1.81 (m, 5 H) 2.80 - 2.93 (m, 1 H) 3.05 (s, 3 H) 3.16 (s, 4 H) 3.45 - 3.62 (m, 2 H) 3.65 - 3.79 (m, 1 H) 5.04 - 5.15 (m, 1 H) 5.80 - 6.03 (m, 1 H) 6.51 - 6.66 (m, 1 H) 6.79 - 6.92 (m, 1 H) 7.03 - 7.16 (m, 1 H) 7.65 - 7.73 (m, 1 H)

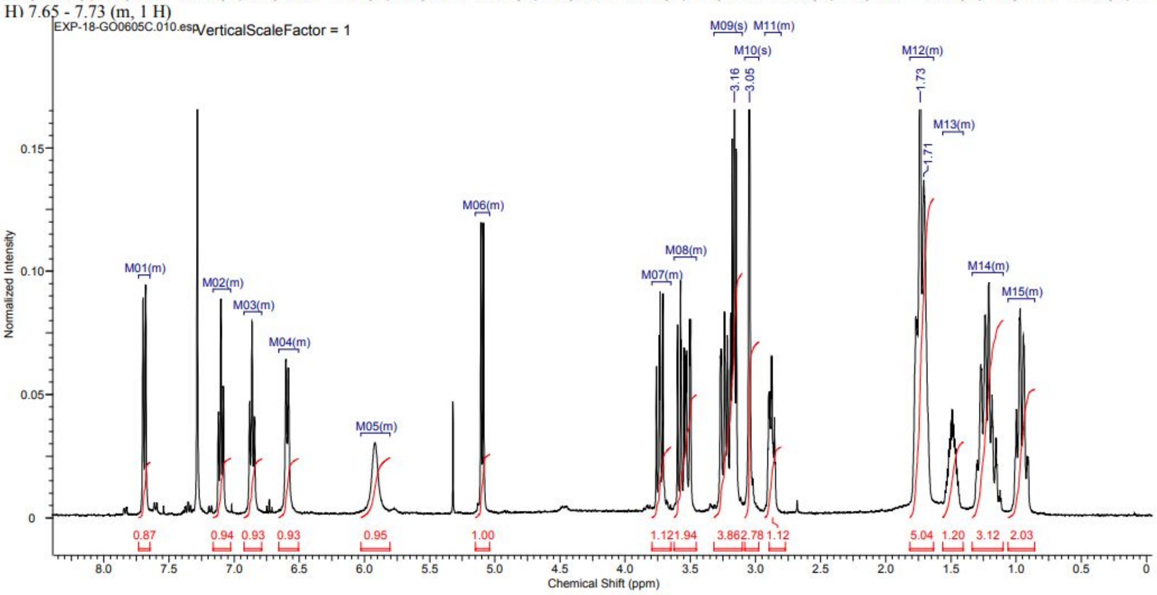

LC-MS Spectra for Compound 80

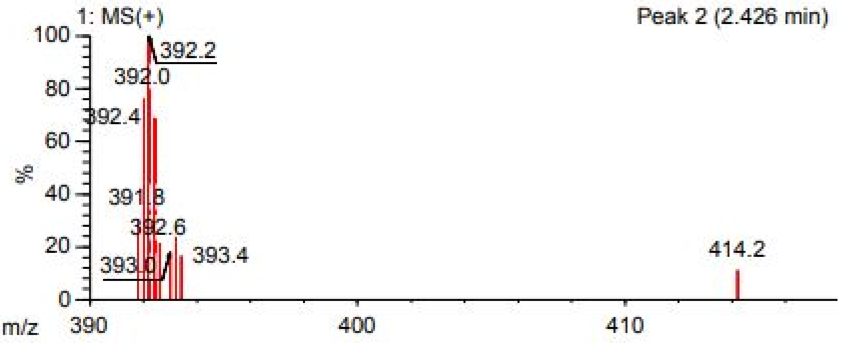

^{1}H NMR Spectra for Compound 81

^{1}H NMR (400 MHz, CHLOROFORM-*d*) δ ppm 0.84 - 1.05 (m, 2 H) 1.10 - 1.35 (m, 4 H) 1.42 - 1.55 (m, 1 H) 1.66 - 1.83 (m, 5 H) 2.93 (s, 4 H) 3.03 (s, 3 H) 3.16 (dd, *J*=13.18, 6.65 Hz, 4 H) 3.35 - 3.46 (m, 1 H) 3.51 - 3.62 (m, 1 H) 3.65 - 3.75 (m, 1 H) 5.10 (d, *J*=7.53 Hz, 1 H) 5.65 - 5.99 (m, 1 H) 6.62 - 6.76 (m, 1 H) 6.80 - 6.95 (m, 1 H) 7.21 (s, 1 H) 7.63 - 7.78 (m, 1 H)

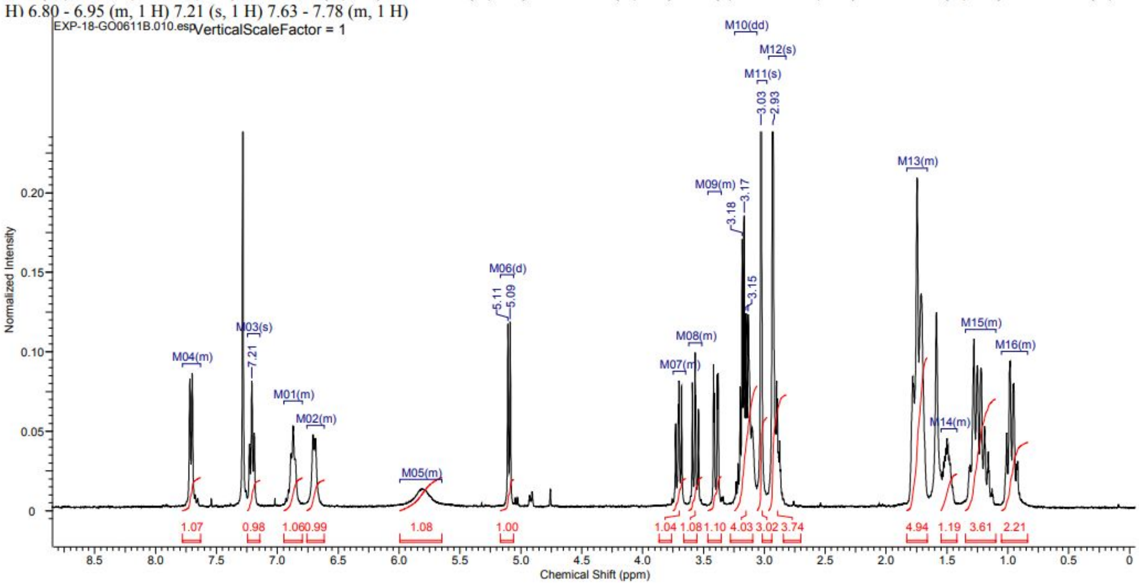

LC-MS Spectra for Compound 81

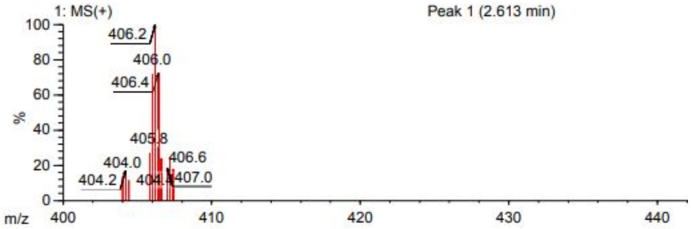

^{1}H NMR Spectra for Compound 82

^{1}H NMR (400 MHz, CHLOROFORM-*d*) δ ppm 0.92 - 1.10 (m, 2 H) 1.13 - 1.36 (m, 3 H) 1.47 - 1.59 (m, 1 H) 1.79 (br. s., 5 H) 2.37 (s, 3 H) 2.63 - 2.74 (m, 1 H) 2.99 (s, 4 H) 3.06 - 3.16 (m, 1 H) 3.18 - 3.29 (m, 1 H) 3.33 - 3.45 (m, 1 H) 3.55 - 3.66 (m, 1 H) 3.96 - 4.11 (m, 1 H) 4.71 - 4.86 (m, 1 H) 5.05 - 5.15 (m, 1 H) 6.59 - 6.71 (m, 1 H) 7.14 - 7.23 (m, 1 H) 7.30 - 7.37 (m, 2 H) 7.87 - 7.94 (m, 1 H)

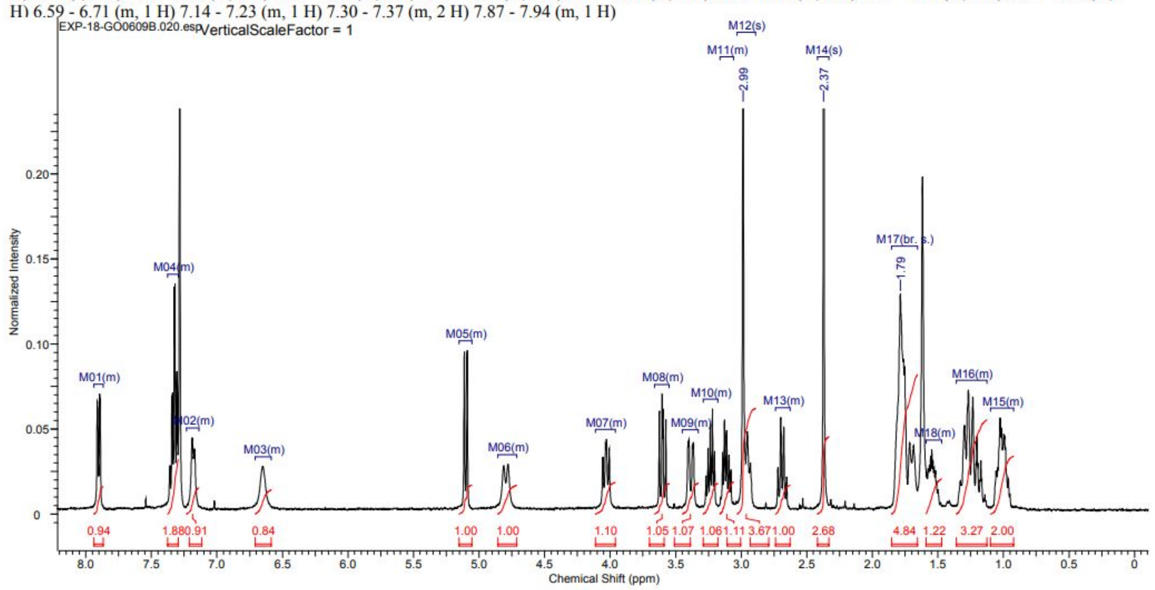

LC-MS Spectra for Compound 82

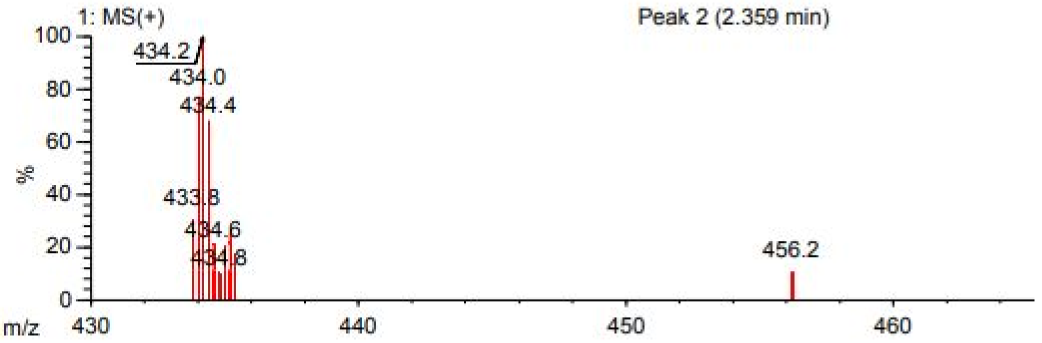

^{13}C spectra for Compound 42

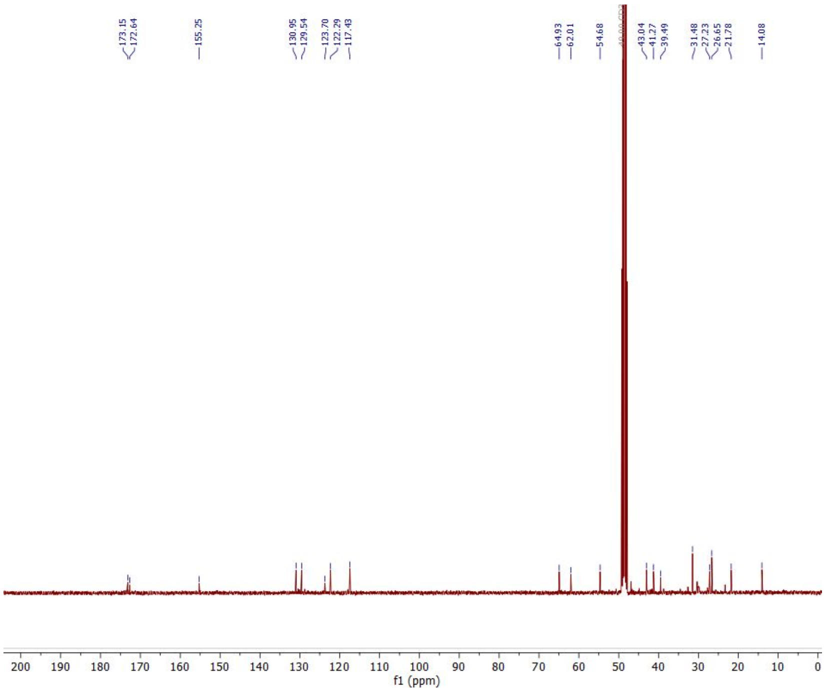

^{13}C spectra for Compound 58

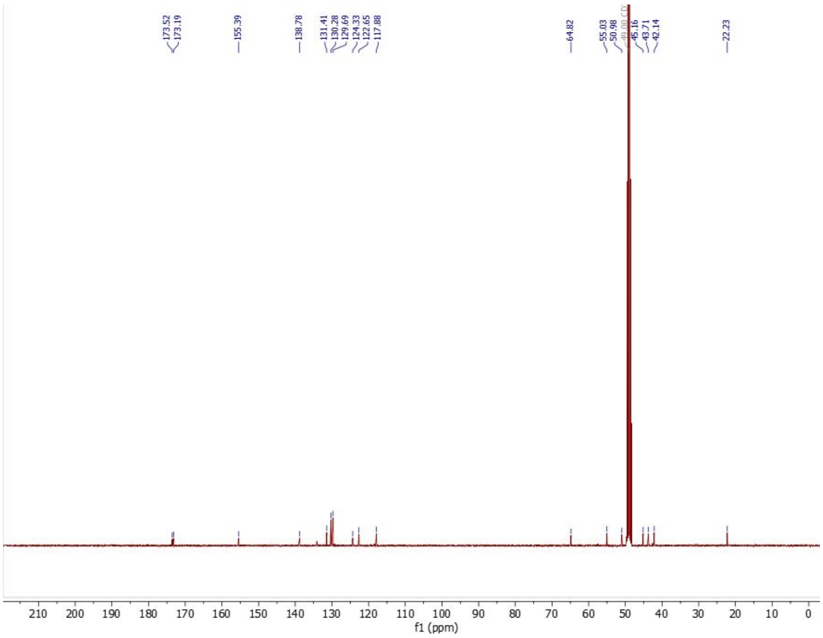

^{13}C spectra for Compound 62

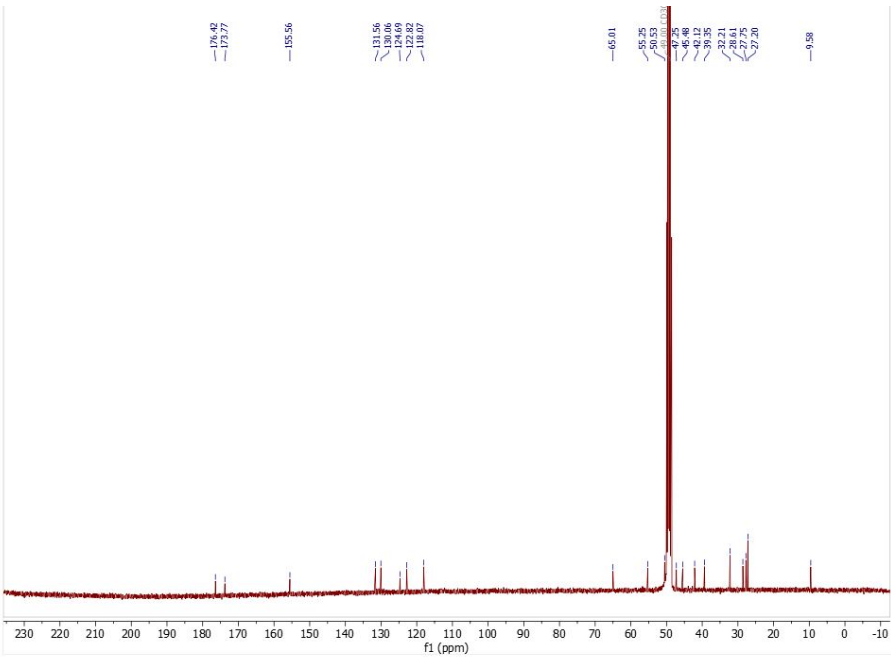

^{13}C spectra for Compound 63

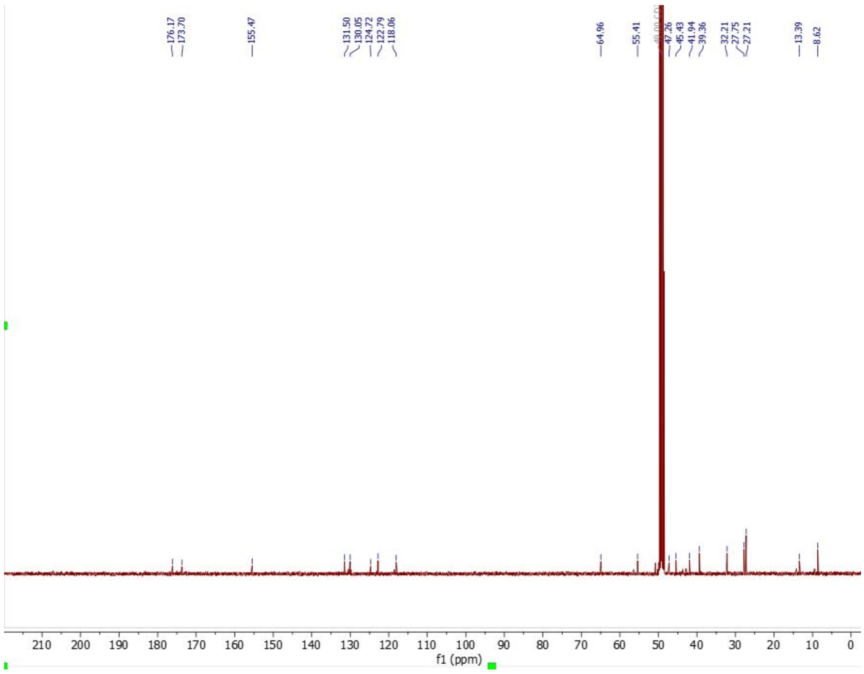

^{13}C spectra for Compound 72

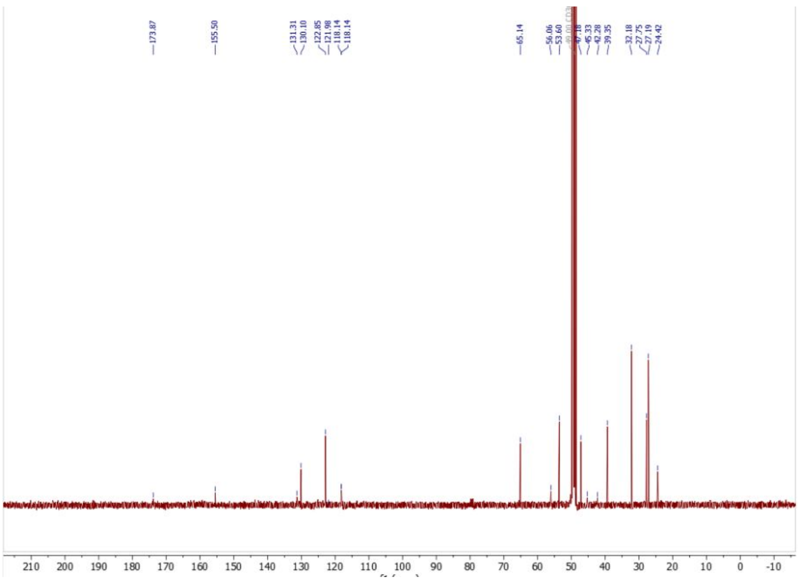
173.87
155.50
131.31
130.10
122.85
121.98
118.14
118.14
65.14
56.06
53.60
47.18
45.33
42.28
39.35
32.18
27.75
27.19
24.42
210
200
190
180
170
160
150
140
130
120
110
100
90
80
70
60
50
40
30
20
10
0
-10

HRMS of lead compounds from SAR investigations

HRMS Table

Compound	Chemical formula	Calculated mass	Observed mass
42	$C_{21}H_{28}N_2O_3Na$ [M+Na$^+$]	379.1992	379.1995
58	$C_{21}H_{21}ClN_2O_3Na$ [M+Na$^+$]	407.1133	407.1140
62	$C_{22}H_{30}N_2O_3Na$ [M+Na$^+$]	393.2149	393.2152
63	$C_{23}H_{30}N_2O_3Na$ [M+H$^+$]	405.2149	405.2156
72	$C_{21}H_{28}N_2O_4Na$ [M+Na$^+$]	395.1941	395.1944

HRMS spectra for compound 42

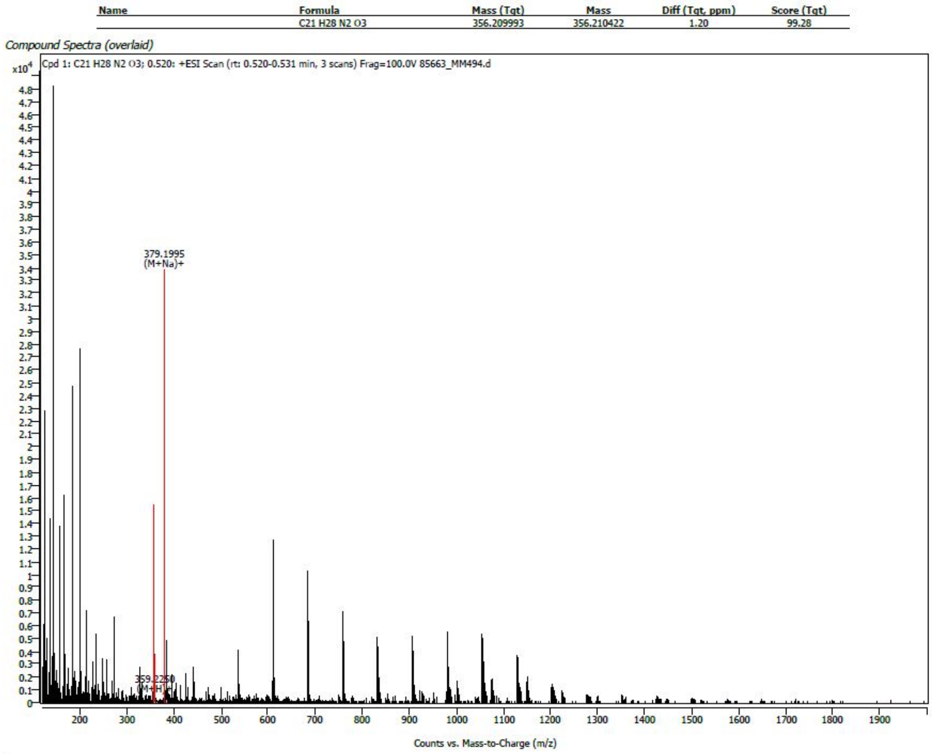

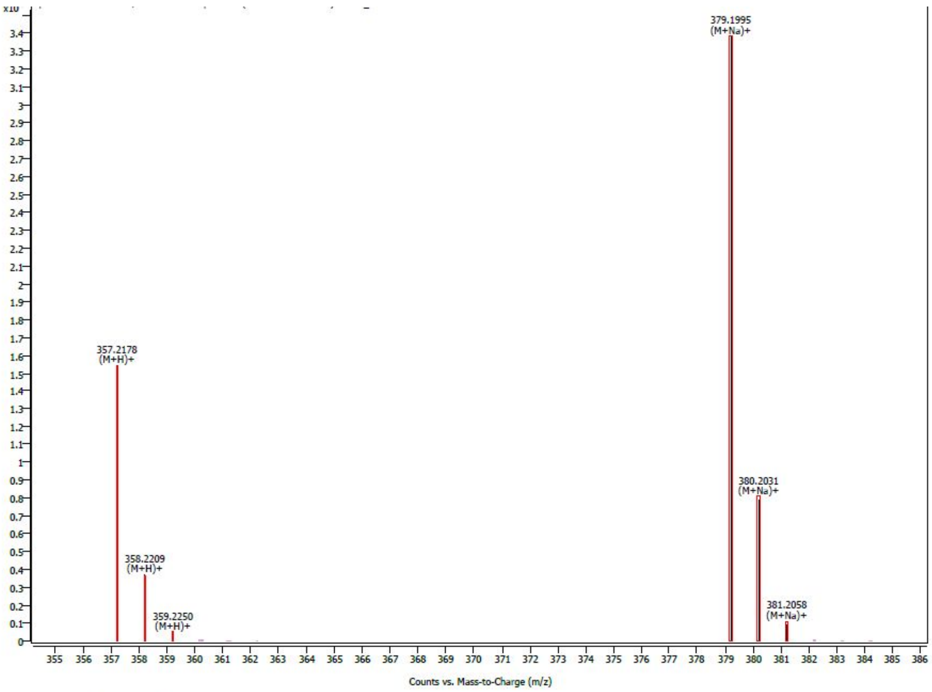

Spectrum Peaks

Ion Species	m/z	m/z (Calc)	Diff (ppm)	Abund	Height %	Height % (Calc)	Z
(M+H)+	357.217803	357.217269	1.49	15392	100.00	100.00	1
(M+H)+	358.220938	358.220476	1.29	3729	24.22	23.89	1
(M+H)+	359.224998	359.223277	4.79	493	3.20	3.35	1
(M+Na)+	379.199511	379.199214	0.78	33840	100.00	100.00	1
(M+Na)+	380.203096	380.202419	1.78	7892	23.32	23.88	1
(M+Na)+	381.205795	381.205219	1.51	925	2.73	3.34	1
	143.002432			48180			

HRMS spectra for compound 58

Cpd. 1: C21 H21 Cl N2 O3

Name	Formula	Mass (Tgt)	Mass	Diff (Tgt, ppm)	Score (Tgt)
	C21 H21 Cl N2 O3	384.124070	384.124641	1.49	98.13

Compound Spectra (overlaid)

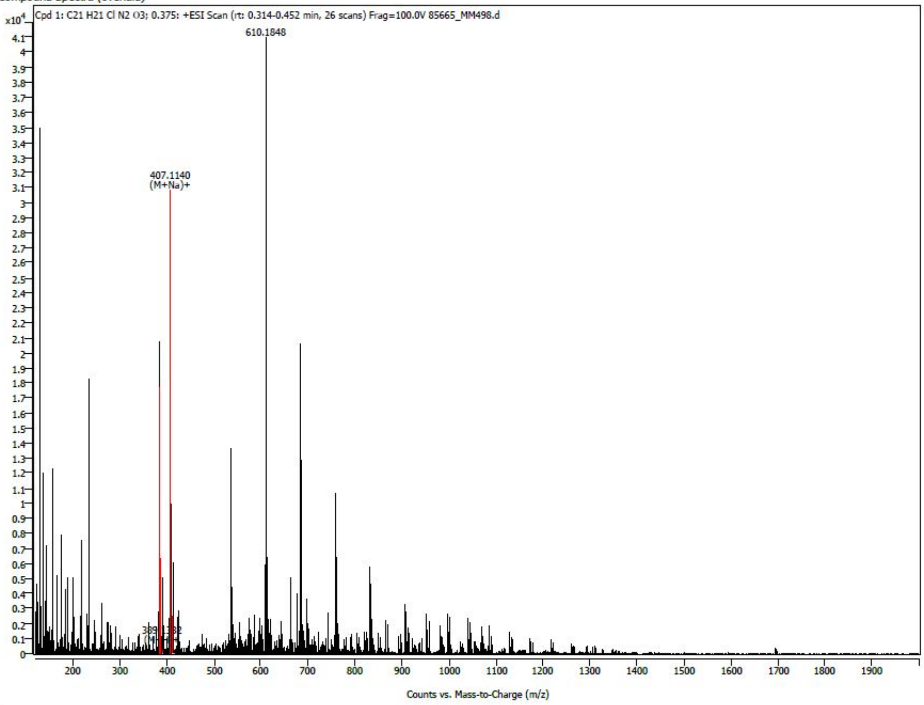

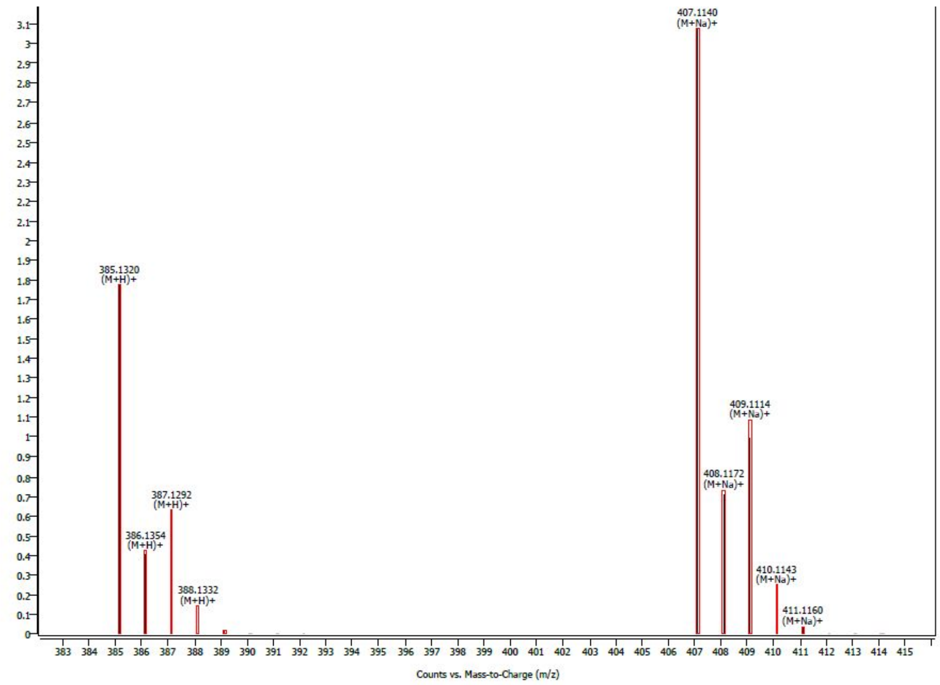

Spectrum Peaks

Ion Species	m/z	m/z (Calc)	Diff (ppm)	Abund	Height %	Height % (Calc)	Z
(M+H)+	385.131967	385.131347	1.61	17741	100.00	100.00	1
(M+H)+	386.135355	386.134543	2.10	4038	22.76	23.81	1
(M+H)+	387.129159	387.129238	-0.21	5718	32.23	35.32	1
(M+H)+	388.133221	388.131955	3.26	1115	6.28	7.96	1
(M+H)+	389.133244	389.134596	-3.47	173	0.97	1.09	1
(M+Na)+	407.114023	407.113291	1.80	30775	100.00	100.00	1
(M+Na)+	408.117197	408.116486	1.74	7083	23.02	23.80	1
(M+Na)+	409.111376	409.111182	0.47	9948	32.32	35.32	1
(M+Na)+	410.114279	410.113898	0.93	2303	7.48	7.96	1
(M+Na)+	411.116000	411.116538	-1.31	291	0.94	1.09	1
	610.184791			40962			

HRMS spectra for compound 62

Name	Formula	Mass (Tgt)	Mass	Diff (Tgt, ppm)	Score (Tgt)
	C22 H30 N2 O3	370.225643	370.225908	0.72	99.22

Compound Spectra (overlaid)

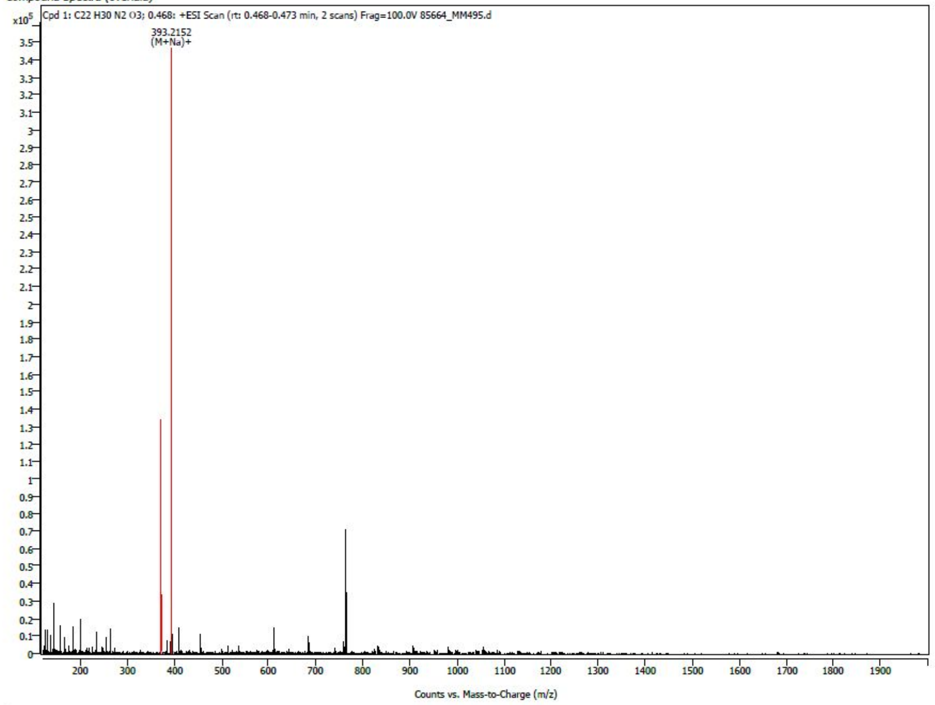

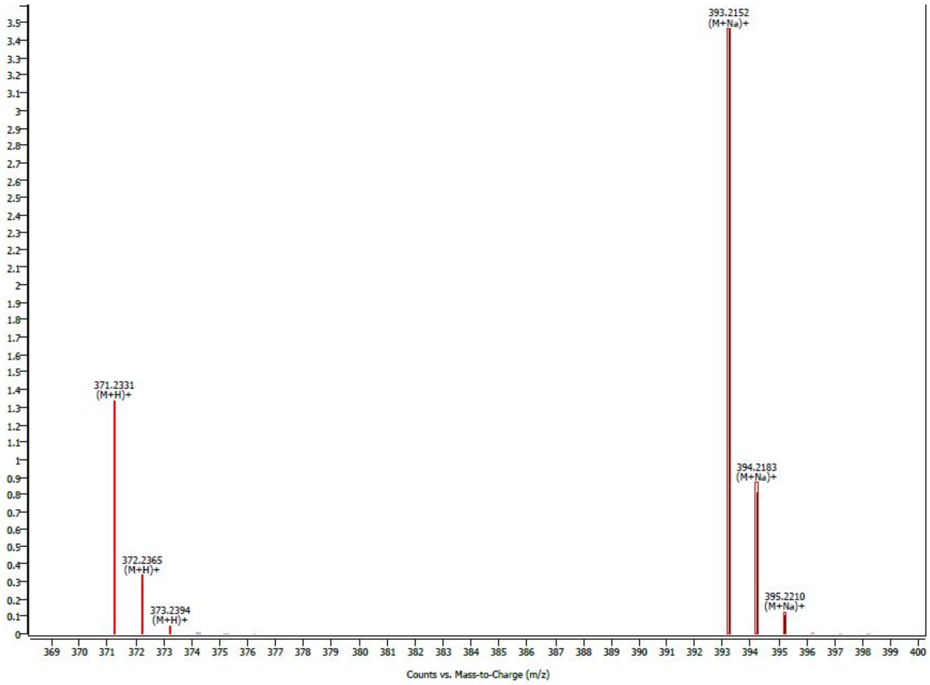

Spectrum Peaks

Ion Species	m/z	m/z (Calc)	Diff (ppm)	Abund	Height %	Height % (Calc)	Z
(M+H)+	371.233141	371.232919	0.60	133715	100.00	100.00	1
(M+H)+	372.236452	372.236135	0.85	28676	21.45	25.00	1
(M+H)+	373.239378	373.238972	1.09	4370	3.27	3.61	1
(M+Na)+	393.215162	393.214864	0.76	346623	100.00	100.00	1
(M+Na)+	394.218275	394.218078	0.50	80650	23.27	24.98	1
(M+Na)+	395.220970	395.220914	0.14	10701	3.09	3.61	1

HRMS spectra for compound 63

Name	Formula	Mass (Tgt)	Mass	Diff (Tgt, ppm)	Score (Tgt)
	C23 H30 N2 O3	382.225643	382.226378	1.92	98.33

Compound Spectra (overlaid)

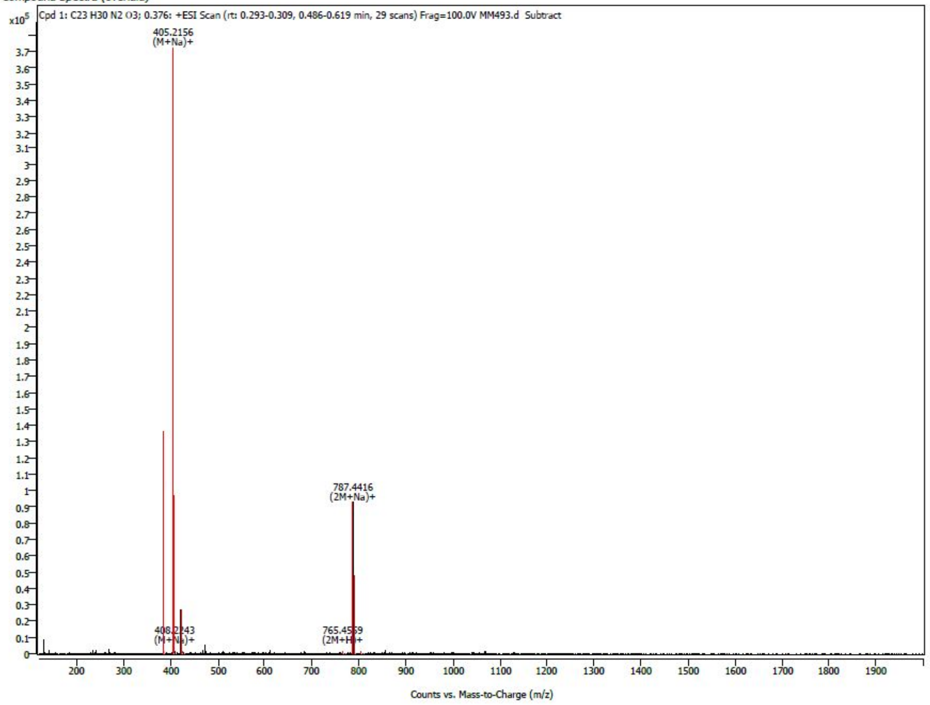

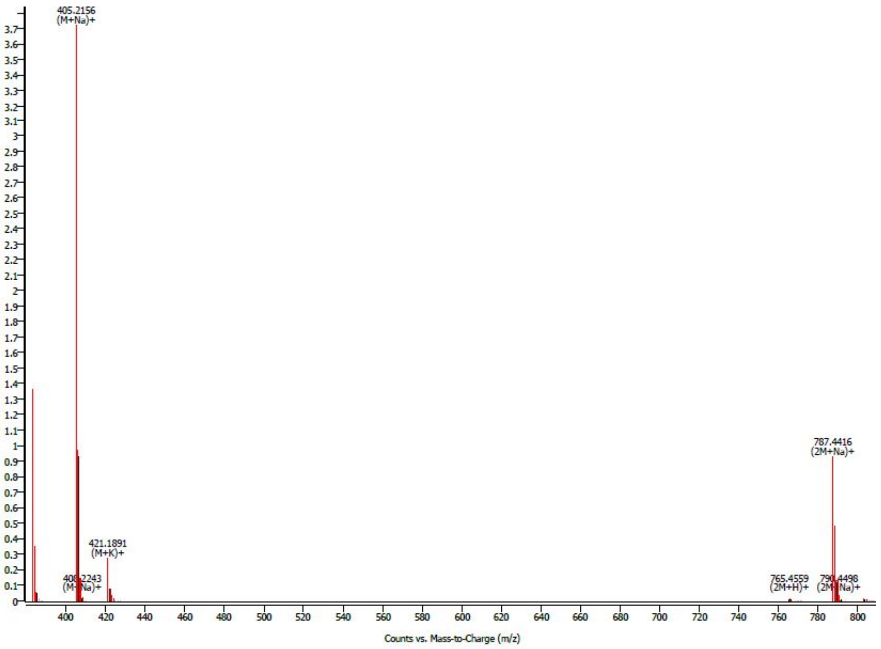

Spectrum Peaks

Ion Species	m/z	m/z (Calc)	Diff (ppm)	Abund	Height %	Height % (Calc)	Z
(M+H)+	383.233454	383.232919	1.40	136171	100.00	100.00	1
(M+H)+	384.236594	384.236141	1.18	31807	23.36	26.08	1
(M+H)+	385.239712	385.239008	1.83	4452	3.27	3.88	1
(M+Na)+	405.215630	405.214864	1.89	371660	100.00	100.00	1
(M+Na)+	406.218708	406.218084	1.54	92812	24.97	26.07	1
(M+Na)+	407.221457	407.220950	1.24	12154	3.27	3.88	1
(M+Na)+	408.224347	408.223676	1.64	1378	0.37	0.42	1
(M+K)+	421.189070	421.188801	0.64	27003	100.00	100.00	1
(M+K)+	422.192715	422.192020	1.65	7109	26.33	26.08	1
(M+K)+	423.188654	423.189706	-2.49	2279	8.44	11.10	1
(M+K)+	424.189154	424.191508	-5.55	411	1.52	2.30	1
(2M+H)+	765.455862	765.458562	-3.53	572	100.00	100.00	1
(2M+H)+	766.464094	766.461783	3.02	249	43.52	52.14	1
(2M+Na)+	787.441611	787.440507	1.40	92838	100.00	100.00	1
(2M+Na)+	788.444924	788.443727	1.52	46632	50.23	52.13	1
(2M+Na)+	789.447720	789.446758	1.22	11632	12.53	14.55	1
(2M+Na)+	790.449808	790.449668	0.18	2233	2.41	2.86	1
(2M+Na)+	791.450445	791.452498	-2.59	398	0.43	0.44	1

HRMS spectra for compound 72

Name	Formula	Mass (Tgt)	Mass	Diff (Tgt, ppm)	Score (Tgt)
	C21 H28 N2 O4	372.204907	372.205142	0.63	99.70

Compound Spectra (overlaid)

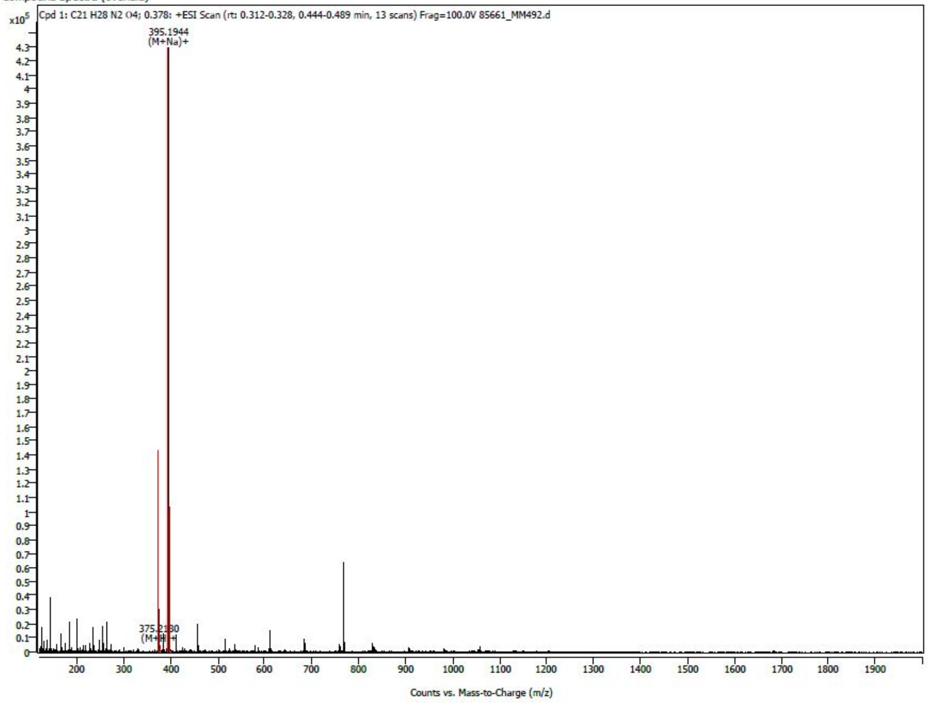

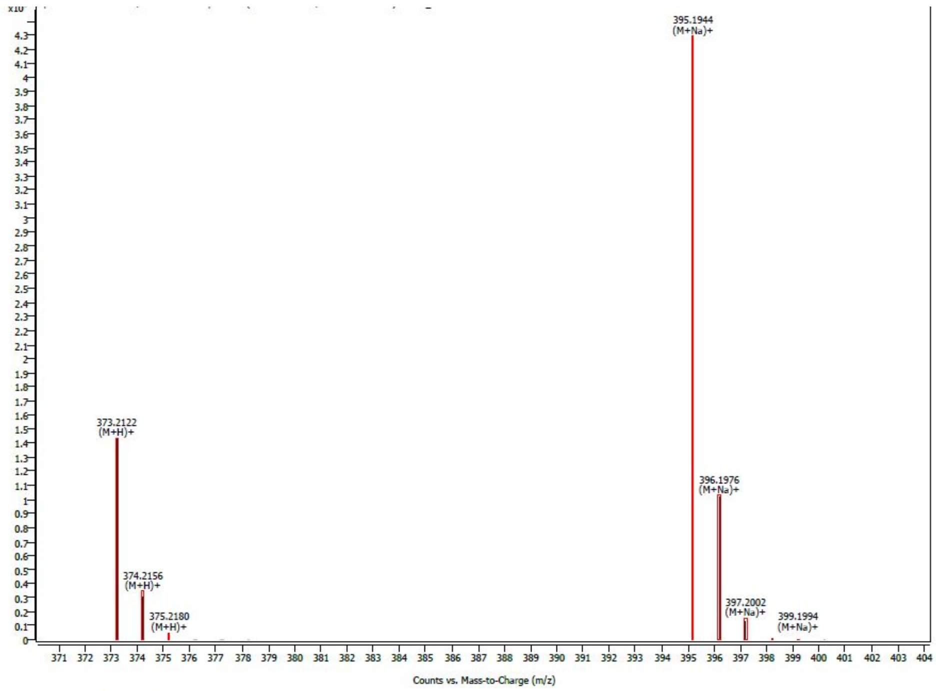

Spectrum Peaks

Ion Species	m/z	m/z (Calc)	Diff (ppm)	Abund	Height %	Height % (Calc)	Z
(M+H)+	373.212226	373.212184	0.11	143283	100.00	100.00	1
(M+H)+	374.215556	374.215392	0.44	30135	21.03	23.93	1
(M+H)+	375.218007	375.218093	-0.23	4307	3.01	3.56	1
(M+Na)+	395.194441	395.194128	0.79	429421	100.00	100.00	1
(M+Na)+	396.197553	396.197335	0.55	101736	23.69	23.92	1
(M+Na)+	397.200222	397.200035	0.47	13293	3.10	3.56	1
(M+Na)+	398.203526	398.202661	2.17	1220	0.28	0.40	1
(M+Na)+	399.199377	399.205248	-14.71	89	0.02	0.04	1

HPLC traces

HPLC trace for compound 42

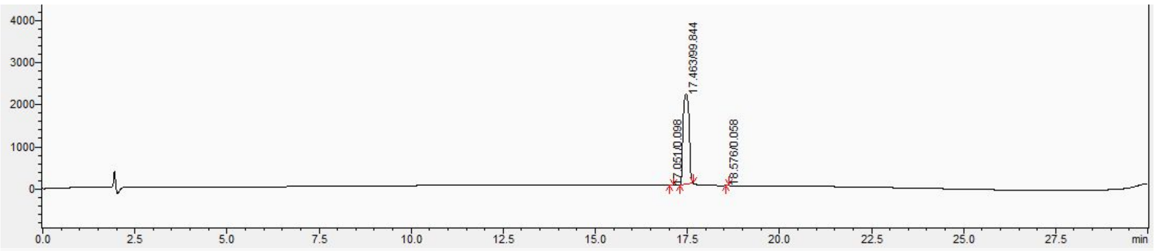

HPLC trace for compound 58

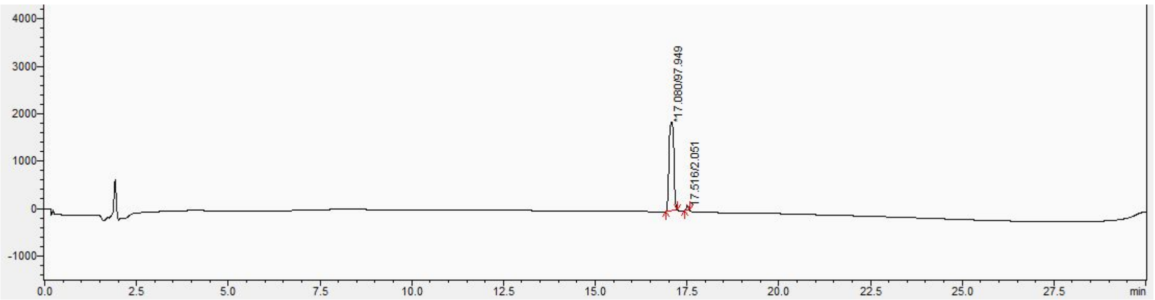

HPLC trace for compound 62

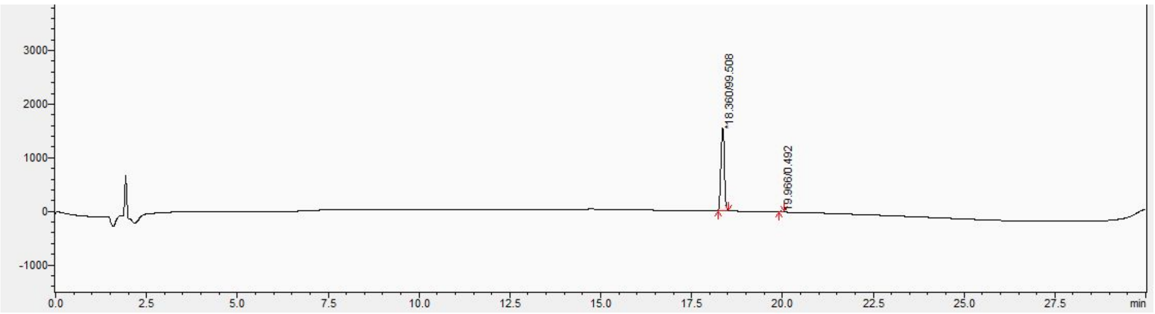

HPLC trace for compound 63

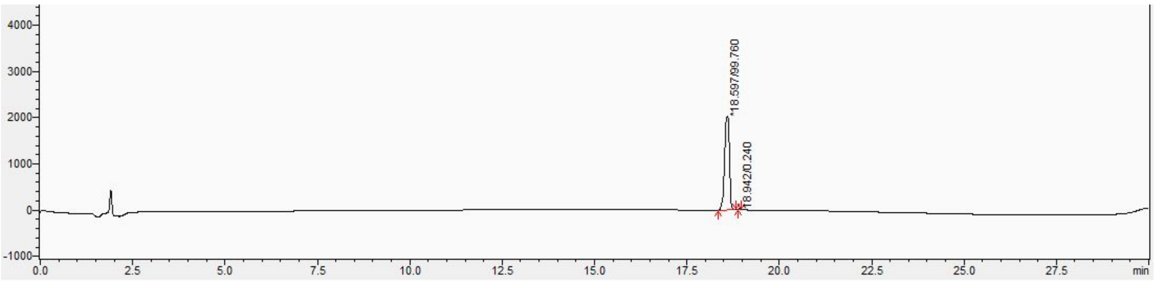

HPLC trace for compound 72

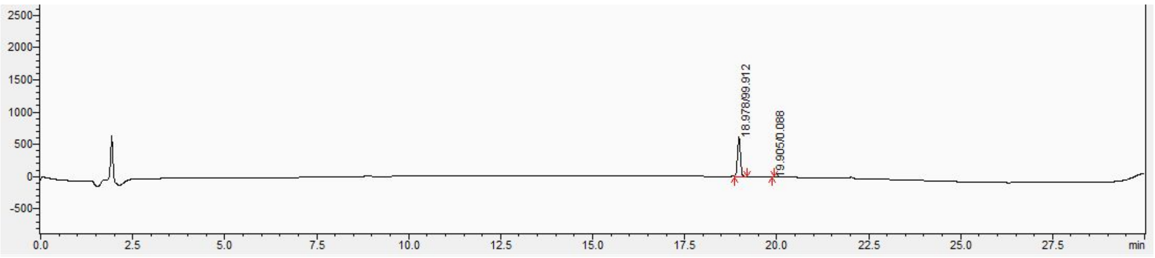

SFC purification

Chiral separation of compound 42

Well	Instrument Method	Inj. Vol. (uL)	Co-Solvent	Column	% Modifier	Temp. (oC)	Flow (mL/min)
11C	Isocratic_AS-H_prep	100	Methanol	AS-H 10x250mm	20	40	15

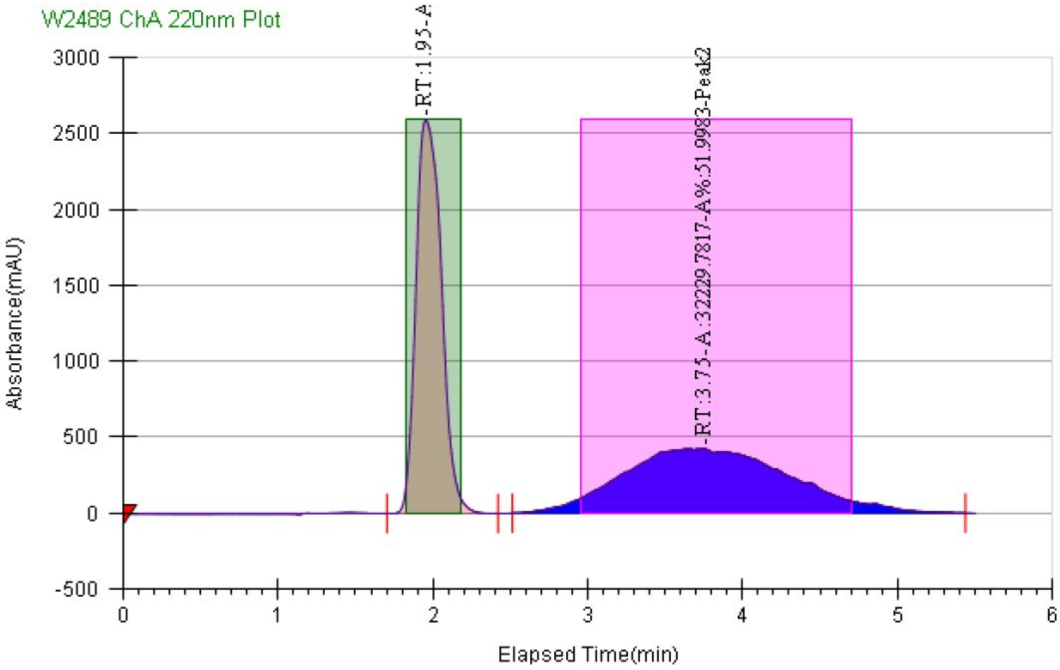

Chiral separation of compound 58

Well	Instrument Method	Inj. Vol. (uL)	Co-Solvent	Column	% Modifier	Temp. (oC)	Flow (mL/min)
11F	Isocratic_AS-H_prep	100	Methanol	AS-H 10x250mm	25	40	15

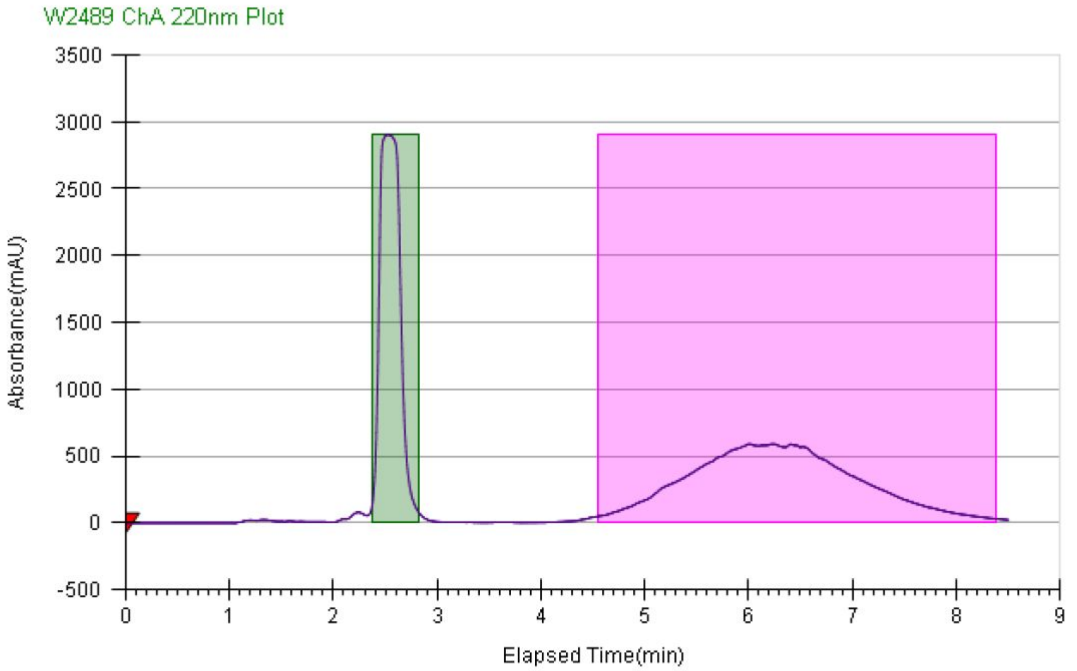

Chiral separation of compound 62

Well	Instrument Method	Inj. Vol. (uL)	Co-Solvent	Column	% Modifier	Temp. (oC)	Flow (mL/min)
11E	Isocratic_AS-H_prep	100	Methanol	AS-H 10x250mm	7.5	40	15

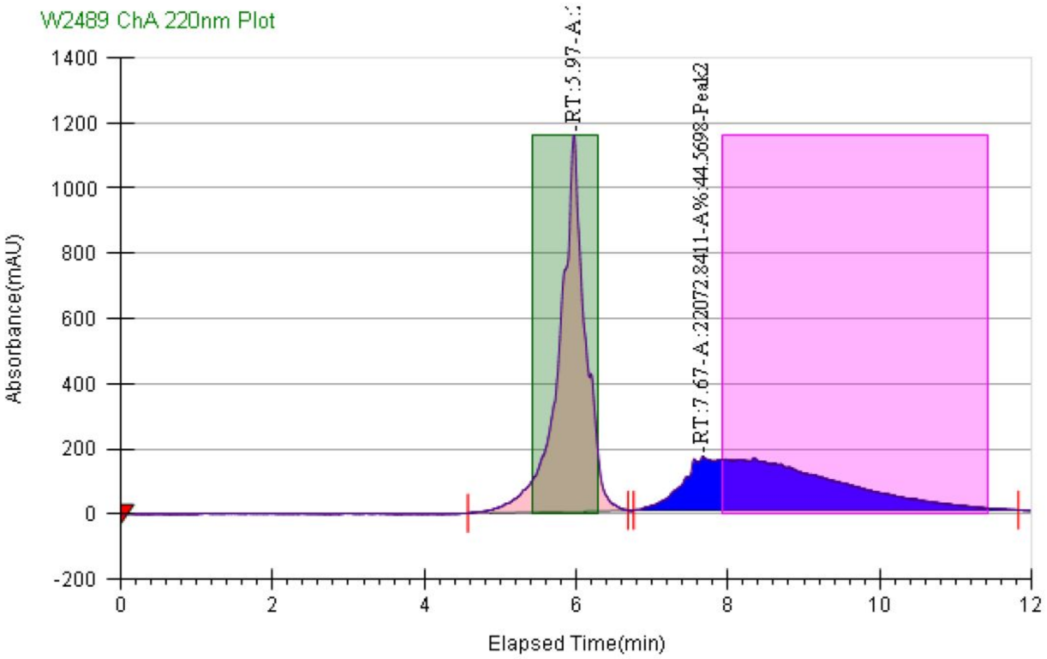

Chiral separation of compound 63

Well	Instrument Method	Inj. Vol. (uL)	Co-Solvent	Column	% Modifier	Temp. (oC)	Flow (mL/min)
11B	Isocratic_OD-H_prep	200	Methanol	OD-H 10x250mm	10	40	15

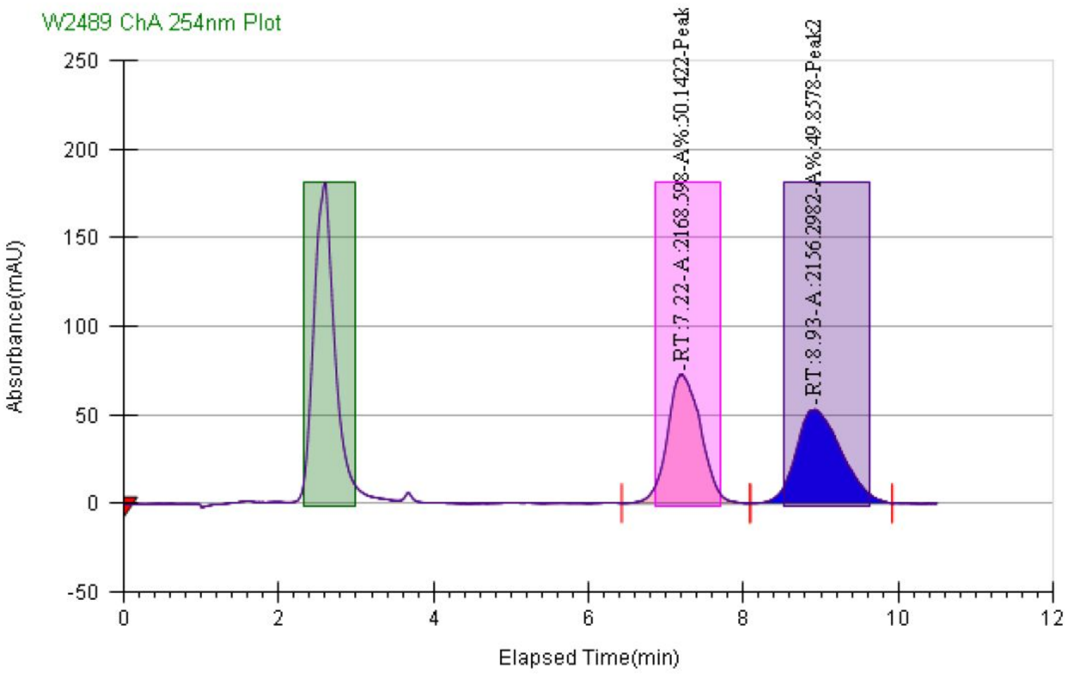

Chiral separation of compound 72

Well	Instrument Method	Inj. Vol. (uL)	Co-Solvent	Column	% Modifier	Temp. (oC)	Flow (mL/min)
11D	Isocratic_AD-H_prep	200	Methanol	AD-H 10x250mm	30	40	15

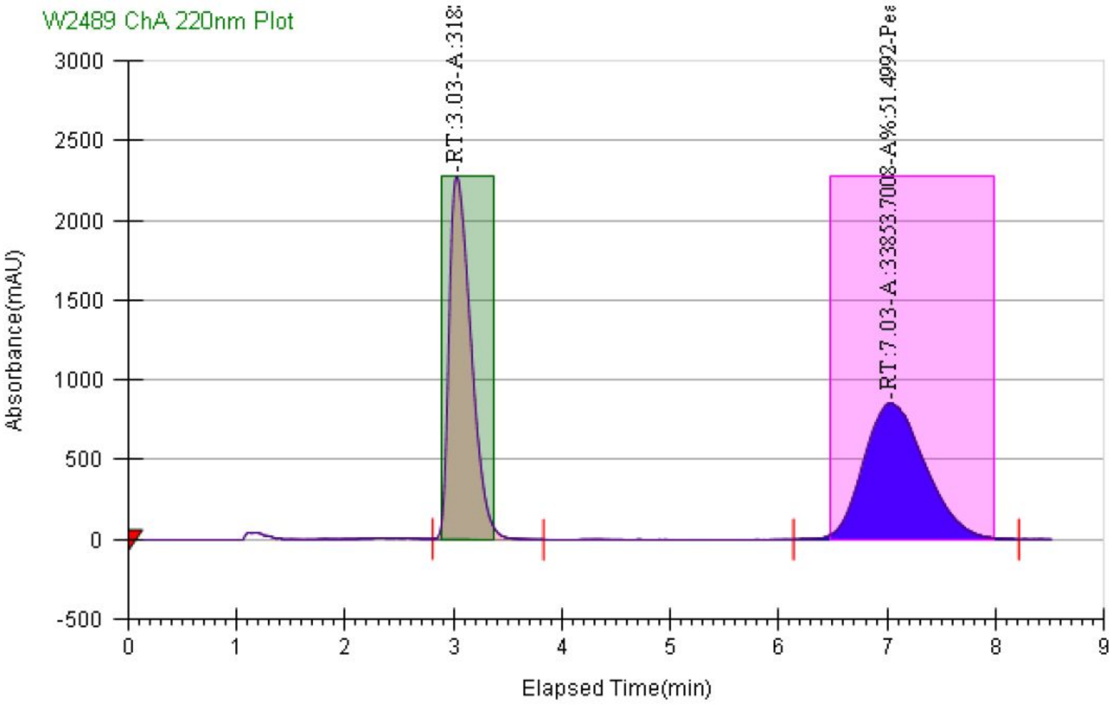

Correlation plot

Cmpnd Name	X = Arrestin-3 recruitmentp-IC50 (Mean $\pm$ SEM)	Y = Gi-protein incooperation-pIC50 (Mean $\pm$ SEM)
52	4.89 ± 0.19	5.41(n=2)
56	4.59 ± 0.04	4.98 ± 0.08
57	5.98 ± 0.09	5.76 ± 0.21
58	6.58 ± 0.06	6.63 ± 0.07
66	5.23 ± 0.18	5.08 ± 0.06
67	5.64 ± 0.03	5.51 ± 0.12
71	5.88 ± 0.14	5.82 ± 0.09
72	6.06 ± 0.11	6.41 ± 0.22
73	5.43 ± 0.11	5.49 ± 0.14
74	5.41 ± 0.16	5.57 ± 0.09
75	5.17 ± 0.05	5.64 ± 0.20
77	5.21 ± 0.17	5.35 ± 0.06

Mutagenesis

Supplementary figure SI-1

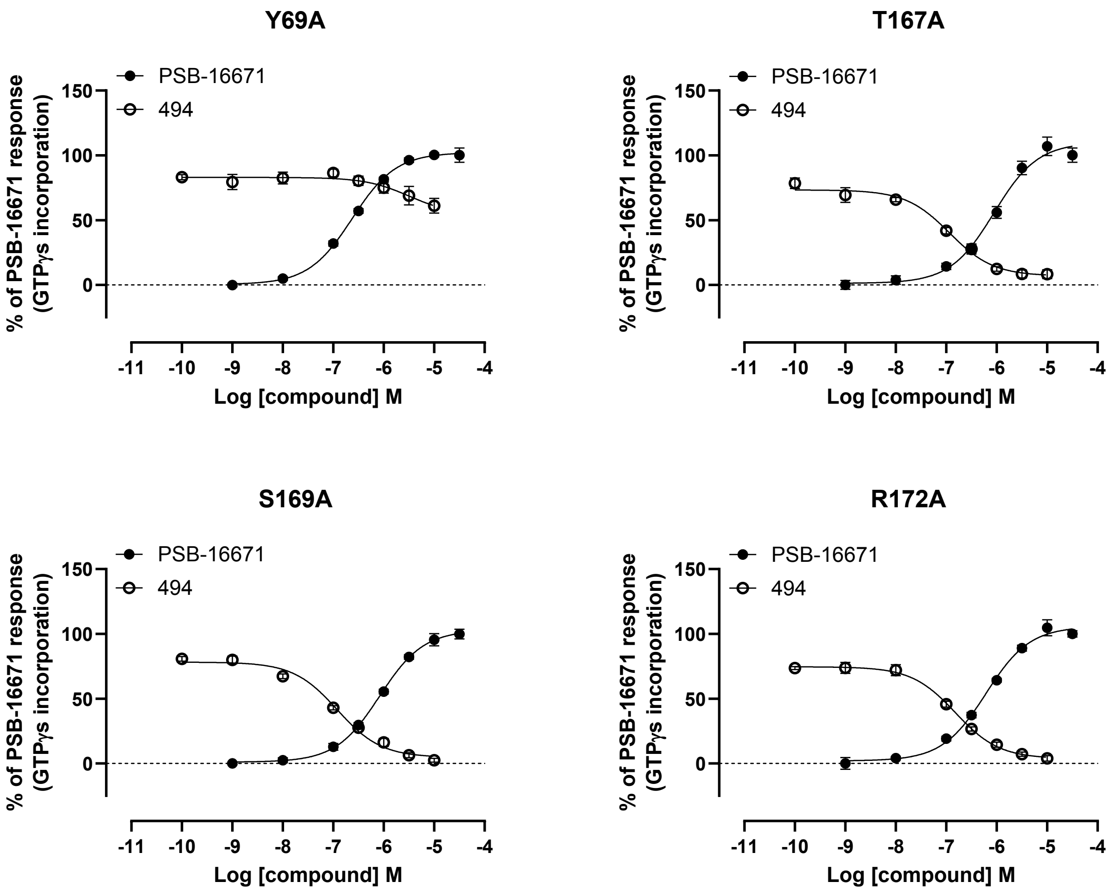

Additional figures

Supplementary figure SI-2

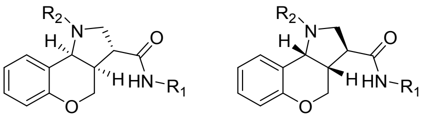

The enantiomers generated from the 1,3-dipolar cyclisation step, where the three contiguous chiral centres of the chromenpyrrole are formed. The concerted nature of this step, in addition to electron withdrawing groups on the dienophile preferring the endo transition state, leads to only to (isomers) the (*S,S,R*) and (*R,S,S*) enantiomers.
